# The hyper-diverse ant genus *Tetramorium* Mayr (Hymenoptera, Formicidae) in the Malagasy region taxonomic revision of the *T. naganum*, *T. plesiarum*, *T. schaufussii*, and *T. severini* species groups

**DOI:** 10.3897/zookeys.413.7172

**Published:** 2014-06-04

**Authors:** Francisco Hita Garcia, Brian L. Fisher

**Affiliations:** 1 Entomology, California Academy of Sciences, 55 Music Concourse Drive, San Francisco, CA 94118, U.S.A.

**Keywords:** Comoros, Madagascar, Reunion, taxonomy, Tetramoriini

## Abstract

The taxonomy of the *Tetramorium naganum*, *T. plesiarum*, *T. schaufussii*, and *T. severini* species groups are revised for the Malagasy region. A total of 31 species are treated, of which 22 are newly described and nine redescribed. This increases the richness of the hyper-diverse genus *Tetramorium* in the Malagasy region to 106 species, which makes it the most species-rich genus in the region. Twenty-nine of the treated species are endemic to Madagascar, one is endemic to the Comoros, and one species is found predominantly in Madagascar but also on the island of Reunion. The *T. naganum* species group contains five species, which are mainly distributed in the rainforests and montane rainforests of eastern and northern Madagascar: *T. alperti* **sp. n.**, *T. dalek* **sp. n.**, *T. enkidu* **sp. n.**, *T. gilgamesh* **sp. n.**, and *T. naganum* Bolton, 1979. The *T. plesiarum* species group holds five species: *T. bressleri* **sp. n.**, *T. hobbit* **sp. n.**, *T. gollum* **sp. n.**, *T. mars* **sp. n.**, and *T. plesiarum* Bolton, 1979. All five are arid-adapted species occurring in the southwest and west of Madagascar. The second-most species-rich group in the region is the *T. schaufussii* species group with 20 species, most of which inhabit rainforests or montane rainforests of eastern and northern Madagascar. This group includes two species complexes each containing ten species: the *T. cognatum* complex with the species *T. aspis* **sp. n.**, *T. camelliae* **sp. n.**, *T. cognatum* Bolton, 1979, *T. freya* **sp. n.**, *T. gladius* **sp. n.**, *T. karthala* **sp. n.**, *T. myrmidon* **sp. n.**, *T. proximum* Bolton, 1979, *T. rumo* **sp. n.**, and *T. tenuinode* **sp. n.**; and the *T. schaufussii* complex with the species *T. merina* **sp. n.**, *T. monticola* **sp. n.**, *T. nassonowii* Forel, 1892 **stat. n.**, *T. obiwan* **sp. n.**, *T. pseudogladius* **sp. n.**, *T. rala* **sp. n.**, *T. schaufussii* Forel, 1891, *T. sikorae* Forel, 1892 (= *T. latior* (Santschi, 1926)), *T. scutum* **sp. n.**, *T. xanthogaster* Santschi, 1911. The last group treated in this study is the *T. severini* species group, which contains only the species *T. severini* (Emery, 1895). This very conspicuous species is widely distributed in the rainforests and montane rainforests of eastern and northern Madagascar. All four groups are fully revised with group diagnoses, illustrated species-level identification keys, and detailed descriptions for all species that include multifocused montage images and distribution maps.

## Introduction

The hyper-diverse genus *Tetramorium* Mayr is widely distributed throughout all biogeographic regions, and with currently 520 described species (Bolton 2014) one of the most species-rich ant genera. *Tetramorium* is most diverse in the subtropics and tropics of the Old World, especially the Afrotropics and Madagascar, but is species-poor in the New World. Most regional faunas were revised by Bolton (1976, 1977, 1979, 1980), who provided a good taxonomic foundation on which later works could build (e.g. Csösz et al. 2007; Csösz and Schulz 2010; Hita Garcia et al. 2010; Hita Garcia and Fisher 2011; Bharti and Kumar 2012; Sharaf et al. 2012). The Malagasy *Tetramorium* fauna was also first revised by Bolton (1979), who treated eight species groups containing 36 species, of which 29 were endemic to Madagascar. The synonymisation of *Triglyphothrix* Forel (Bolton 1985) under *Tetramorium* added an additional species group with one tramp species; two additional tramp species were reported much later (Blard et al. 2003; Roberts and McGlynn 2004), for a total of 39 species known prior to 2011.

Recently, we started a large-scale taxonomic revision of the genus *Tetramorium* for the Malagasy region based initially on more than 160 morphospecies with more than 40,000 mounted specimens (Hita Garcia and Fisher 2011). We proposed 14 species groups as a foundation and provided a preliminary identification key to the Malagasy groups. In the same study we also revised the taxonomy of the *Tetramorium bicarinatum*, *Tetramorium obesum*, *Tetramorium sericeiventre*, and *Tetramorium tosii* species groups. One species was newly described while another was synonymised, leaving the species count for the region at 39. Based on that work, the *Tetramorium bessonii*, *Tetramorium bonibony*, *Tetramorium dysalum*, *Tetramorium kelleri*, *Tetramorium marginatum*, *Tetramorium tortuosum*, *Tetramorium tsingy*, and *Tetramorium tosii* species groups were revised shortly afterward (Hita Garcia and Fisher 2012a, 2012b). The latter two studies dealt with 58 species, of which 45 were described as new, and increased the species count for the Malagasy region to 84. We also proposed additional species groups for a total of 18 for the region (not 19 as noted in Hita Garcia and Fisher 2012b).

In this study we revise the taxonomy of four more Malagasy species groups: *Tetramorium naganum*, *Tetramorium plesiarum*, *Tetramorium schaufussii*, and *Tetramorium severini* species groups. All are fully revised with group diagnoses, illustrated species-level identification keys, multifocused montage images for all species, descriptions of 22 new species, redescriptions of nine previously known species, and distribution maps for all species. The species treated here increase the total species count for the genus *Tetramorium* in the Malagasy region to 106, which means that at present this genus holds the highest described species richness in the region. Furthermore, this study increases the number of recently revised Malagasy *Tetramorium* species groups to 16 (Hita Garcia and Fisher 2011, 2012a, 2012b), leaving only two species groups untreated: the species-rich *Tetramorium ranarum* group and the less diverse but abundant *Tetramorium simillimum* group. These groups will be revised in an upcoming study.

### Abbreviations of depositories

The collection abbreviations follow Evenhuis (2013). The material upon which this study is based is located and/or was examined at the following institutions:

BMNH The Natural History Museum (British Museum, Natural History), London, U.K.

CAS California Academy of Sciences, San Francisco, California, U.S.A.

MCSN Museo Civico di Storia Naturale Giacomo Doria, Genoa, Italy

MCZ Museum of Comparative Zoology, Cambridge, Massachusetts, U.S.A.

MHNG Muséum d’Histoire Naturelle de la Ville de Genève, Geneva, Switzerland

NHMB Naturhistorisches Museum, Basel, Switzerland

## Material and methods

The material examined in this study is based on ant inventories carried out in the Malagasy region from 1992 to 2012, which included more than 6,000 leaf litter samples, 4,000 pitfall traps, and 9,000 additional hand collecting events (see Fisher 2005 for additional details). All new type material and all imaged specimens can be uniquely identified with specimen-level codes affixed to each pin (e.g. CASENT0078328). In the presented descriptions we list all of the available specimen-level codes for the whole type series. It should be noted, however, that the number of stated paratype workers does not necessarily match the number of listed specimen-level codes because several pins hold more than one specimen.

Digital colour montage images were created using a JVC KY-F75 digital camera and Syncroscopy Auto-Montage software (version 5.0), or a Leica DFC 425 camera in combination with the Leica Application Suite software (version 3.8). All images presented are available online and can be seen on AntWeb (http://www.antweb.org). The distribution maps provided at the end of the study (Figs 61–66) were generated with R software (R Core Team 2014). The measurements were taken with a Leica MZ 12.5 stereomicroscope equipped with an orthogonal pair of micrometers at a magnification of 100×, rarely 80×. Measurements and indices are presented as minimum and maximum values with arithmetic means in parentheses. In addition, all measurements are expressed in mm to two decimal places. The measurements and indices used in this study follow Bolton (1975, 1979, 1980), Güsten et al. (2006), and Hita Garcia and Fisher (2011, 2012a, 2012b, 2013):

HL Head length: maximum distance from the midpoint of the anterior clypeal margin to the midpoint of the posterior margin of head, measured in full-face view. Impressions on anterior clypeal margin and posterior head margin reduce head length.

HW Head width: width of head directly behind the eyes measured in full-face view.

SL Scape length: maximum scape length excluding basal condyle and neck.

EL Eye length: maximum diameter of compound eye measured in oblique lateral view.

PW Pronotal width: maximum width of pronotum measured in dorsal view.

WL Weber’s length: diagonal length of mesosoma in lateral view from the posteroventral margin of propodeal lobe to the anteriormost point of pronotal slope, excluding the neck.

PSL Propodeal spine length: the tip of the measured spine, its base, and the centre of the propodeal concavity between the spines must all be in focus. Using a dual-axis micrometer the spine length is measured from the tip of the spine to a virtual point at its base where the spine axis meets orthogonally with a line leading to the median point of the concavity.

PTH Petiolar node height: maximum height of petiolar node measured in lateral view from the highest (median) point of the node to the ventral outline. The measuring line is placed at an orthogonal angle to the ventral outline of the node.

PTL Petiolar node length: maximum length of the dorsal face of the petiolar node from the anterodorsal to the posterodorsal angle, measured in dorsal view excluding the peduncle.

PTW Petiolar node width: maximum width of dorsal face of petiolar node measured in dorsal view.

PPH Postpetiole height: maximum height of the postpetiole measured in lateral view from the highest (median) point of the node to the ventral outline. The measuring line is placed at an orthogonal angle to the ventral outline of the node.

PPL Postpetiole length: maximum length of postpetiole measured in dorsal view.

PPW Postpetiole width: maximum width of postpetiole measured in dorsal view.

OI Ocular index: EL / HW × 100

CI Cephalic index: HW / HL × 100

SI Scape index: SL / HW × 100

DMI Dorsal mesosoma index: PW / WL × 100

LMI Lateral mesosoma index: PH / WL × 100

PSLI Propodeal spine index: PSL / HL × 100

PeNI Petiolar node index: PTW / PW × 100

LPeI Lateral petiole index: PTL / PTH × 100

DPeI Dorsal petiole index: PTW / PTL × 100

PpNI Postpetiolar node index: PPW / PW × 100

LPpI Lateral postpetiole index: PPL / PPH × 100

DPpI Dorsal postpetiole index: PPW / PPL × 100

PPI Postpetiole index: PPW / PTW × 100

Note that the petiole and postpetiole were measured differently. For the petiole, only the petiolar node was measured, excluding the peduncle, as the node has proved to be of high diagnostic value (Hita Garcia et al. 2010). Measurements of the whole petiole, peduncle plus node, would mask important differences between species. In contrast, we measured the whole postpetiole because it was rounded in most species and without a distinct peduncle-like structure. As a consequence, some information might be lost in the few species with a moderately or strongly anteroposteriorly compressed postpetiole. Even so, the postpetiole measurements as defined still permit better comparisons for most species.

Pubescence and pilosity are often of high diagnostic value within the genus *Tetramorium* (e.g. Bolton 1976, 1980, 1985; Hita Garcia et al. 2010, Hita Garcia and Fisher 2011, 2012a). The varying degree of inclination of pilosity is particularly important for the diagnosis of species, complexes, or groups. In this context we use the terms “erect”, “suberect”, “subdecumbent”, “decumbent”, and “appressed” following Wilson (1955).

## Synopsis of Malagasy *Tetramorium* species treated in this study

***Tetramorium naganum* species group**
  *Tetramorium alperti* sp. n.
  *Tetramorium dalek* sp. n.
  *Tetramorium enkidu* sp. n.
  *Tetramorium gilgamesh* sp. n.
  *Tetramorium naganum* Bolton, 1979
***Tetramorium plesiarum* species group**
  *Tetramorium bressleri* sp. n.
  *Tetramorium hobbit* sp. n.
  *Tetramorium gollum* sp. n.
  *Tetramorium mars* sp. n.
  *Tetramorium plesiarum* Bolton, 1979
***Tetramorium schaufussii* species group**
  ***Tetramorium cognatum* species complex**
    *Tetramorium aspis* sp. n.
    *Tetramorium camelliae* sp. n.
    *Tetramorium cognatum* Bolton, 1979
    *Tetramorium freya* sp. n.
    *Tetramorium gladius* sp. n.
    *Tetramorium karthala* sp. n.
    *Tetramorium myrmidon* sp. n.
    *Tetramorium proximum* Bolton, 1979
    *Tetramorium rumo* sp. n.
    *Tetramorium tenuinode* sp. n.
  ***Tetramorium schaufussii* species complex**
    *Tetramorium merina* sp. n.
    *Tetramorium monticola* sp. n.
    *Tetramorium nassonowii* Forel, 1892, stat. n.
    *Tetramorium obiwan* sp. n.
    *Tetramorium pseudogladius* sp. n.
    *Tetramorium rala* sp. n.
    *Tetramorium schaufussii* Forel, 1891
    *Tetramorium sikorae* Forel, 1892
    = *Tetramorium latior* (Santschi, 1926)
    *Tetramorium scutum* sp. n.
    *Tetramorium xanthogaster* Santschi, 1911
***Tetramorium severini* species group**
  *Tetramorium severini* (Emery, 1895)

### Revision of the *Tetramorium naganum* species group

#### *Tetramorium naganum* species group

**Diagnosis.** Eleven-segmented antennae; anterior clypeal margin medially impressed; frontal carinae always well developed, diverging posteriorly and usually approaching or reaching corners of posterior head margin; antennal scrobe present, weakly to very well developed; mesosoma moderately to strongly marginate from sides to dorsum; propodeal spines medium-sized to long, elongate-triangular to spinose; propodeal lobes triangular and short; petiolar node in profile high rounded nodiform to rectangular nodiform with moderately to well-rounded margins, in profile 1.5 to two times higher than long (LPeI 50–68), in dorsal view between 1.0 and 1.4 times wider than long (DPeI 103–139), anterior and posterior faces parallel, anterodorsal and posterodorsal margins usually at about same height (sometimes anterodorsal margin higher); postpetiole in profile always more or less globular; mandibles variably sculptured, but mostly unsculptured; cephalic dorsum and dorsal mesosoma with distinct longitudinally rugose sculpture; waist segments and gaster always unsculptured, smooth, and shiny; dorsal surfaces of head, mesosoma, and usually waist segments with few to abundant long, standing hairs; pilosity/pubescence on first gastral tergite variable, but with tendencies to more inclined pilosity and dense pubescence; sting appendage spatulate.

**Comments.** This small and compact group is restricted in its distribution to eastern and northern Madagascar. All five species are found only in rainforests or montane rainforests and seem to live mainly in leaf litter.

Within the 13 species groups with 11-segmented antennae, the *Tetramorium naganum* group shares the complete lack of sculpture on both waist segments with the majority of groups, but differs from the *Tetramorium kelleri*, *Tetramorium ranarum*, *Tetramorium tortuosum*, and parts of the *Tetramorium dysalum* groups. These groups contain only species in which either one or both waist segments are clearly sculptured. In addition, the very well developed sculpture on head and mesosoma distinguishes the *Tetramorium naganum* group from the groups with reduced sculpture: the *Tetramorium bessonii*, *Tetramorium marginatum*, and *Tetramorium tsingy* groups. Also, *Tetramorium severini*, the only member of the *Tetramorium severini* group, has a longer and lower mesosoma (LMI 35–38) with less margination from the sides to the dorsum, while the species of the *Tetramorium naganum* group have a higher (LMI 40–46), stouter, and more angled mesosoma. The *Tetramorium plesiarum* group is characterised by the presence of relatively deep and well-developed antennal scrobes with margins all around and a strongly developed median scrobal carina, a character always absent in the *Tetramorium naganum* group. The latter also cannot be mistaken for the *Tetramorium bonibony* group, in which some species possess a very conspicuous bump or protuberance on the pronotal dorsum while the remainder of the species have triangular or cuneiform petiolar nodes. The differentiation between the *Tetramorium naganum* group and the *Tetramorium dysalum* group can be more difficult however. The *Tetramorium dysalum* group is relatively heterogeneous and there are a few species that are morphologically close to some species in the *Tetramorium naganum* group. Nevertheless, the best method to discriminate between these two groups is to compare gastral pubescence and/or pilosity. In all species of the *Tetramorium dysalum* group appressed pubescence on the first gastral is scarce and inconspicuous, and pilosity consists of numerous long suberect to erect hairs. By contrast, pubescence and pilosity are very variable in the *Tetramorium naganum* group, but never with very reduced pubescence and long, standing pilosity as in the *Tetramorium dysalum* group.

The separation from the *Tetramorium schaufussii* group is likely the most difficult, and *Tetramorium naganum* was a member of that group until recently (Hita Garcia and Fisher 2012a). The five species of the *Tetramorium naganum* group have much stronger developed frontal carinae, a generally broader head (CI 92–99), a higher mesosoma (LMI 40–46), and usually longer propodeal spines (PSLI 25–37). In the *Tetramorium schaufussii* group most species (but not all) have weaker frontal carinae, a usually thinner head (CI 85–95), a lower mesosoma (LMI 35–42), and usually much shorter propodeal spines (PSLI 7–28). These values overlap in a few species. In fact, most members of the *Tetramorium schaufussii* group have relatively short spines with a PSLI below 20, a few species have slightly longer spines (PSLI 20–25), and only a few specimens of *Tetramorium gladius* and *Tetramorium rumo* have a higher PSLI of 26–28.

The five species of the group are morphologically very close to each other, and their delimitations are mainly based on different patterns of pilosity/pubescence on the waist segments and the first gastral tergite. *Tetramorium dalek* is easily separable from the other four species on the basis of the absence of standing hairs on the waist segments and shorter propodeal spines, but *Tetramorium alperti*, *Tetramorium enkidu*, *Tetramorium gilgamesh*, and *Tetramorium naganum* are morphologically very similar. Indeed, they are often difficult to discriminate and there are only very few reliable diagnostic characters, mainly gastral pilosity/pubescence. It is possible that two, three, or all four are conspecific and the abovementioned differences are just intraspecific variation. Nevertheless, we prefer to treat them as four distinct species since gastral pilosity/pubescence is usually very species-specific within the genus *Tetramorium* and other myrmicine genera and the material listed here can be moderately well distinguished by the diagnostics given in the key and the descriptions. However, we cannot rule out that gastral pilosity/pubescence is highly variable in this group, and if so, our hypotheses may not reflect real species boundaries.

##### Identification key to species of the *Tetramorium naganum* species group (workers)

**Table:** 

1	Propodeal spines relatively shorter (PSLI 25–27); waist segments without long standing hairs, instead with short, appressed to subdecumbent pubescence only, sometimes with one or two short erect to suberect hairs (Fig. 1A) [Madagascar]	*Tetramorium dalek*
–	Propodeal spines relatively longer (PSLI 27–37); waist segments always with long standing hairs and short appressed to subdecumbent pubescence (Fig. 1B)	2
2	In profile petiolar node relatively thicker, around 1.5 to 1.6 times higher than long (LPeI 60–68) (Fig. 2A, B)	3
–	In profile petiolar node relatively thinner, around 1.7 to 2.0 times higher than long (LPeI 50– 59) (Fig. 2C, D)	4
3	First gastral tergite with moderately long, relatively scattered, appressed to decumbent pubescence in combination with several much longer, fine, and erect hairs (Fig. 3A) [Madagascar]	*Tetramorium alperti*
–	First gastral tergite with moderately short, abundant, subdecumbent to suberect pilosity, and without short, dense, appressed to decumbent pubescence or long, fine erect hairs (Fig. 3B) [Madagascar]	*Tetramorium enkidu*
4	Eyes larger (OI 25–27); first gastral tergite covered by a mix of short to moderately long, abundant, decumbent to suberect pilosity, pilosity appearing disorganized due to varying degrees of inclination and hair length (Fig. 4A) [Madagascar]	*Tetramorium gilgamesh*
–	Eyes smaller (OI 21–23); first gastral tergite with short, dense, appressed to decumbent pubescence only, without any long, standing pilosity (Fig. 4B) [Madagascar]	*Tetramorium naganum*

**Figure 1.**
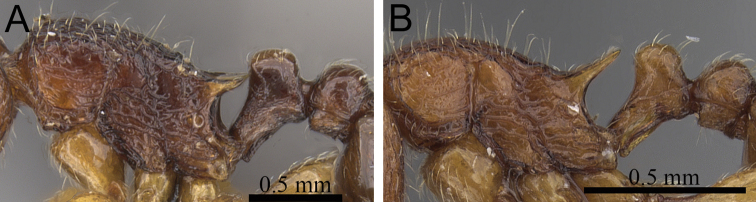
Propodeal spines and waist segments in profile. **A** *Tetramorium dalek* (CASENT0038402) **B** *Tetramorium naganum* (CASENT0280584).

**Figure 2.**
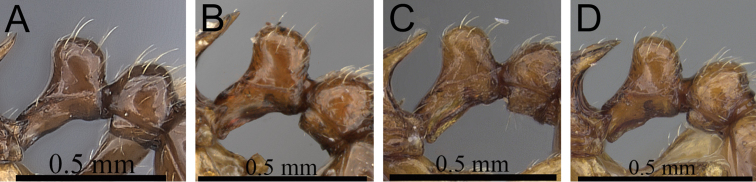
Petiole and postpetiole in profile. **A** *Tetramorium alperti* (CASENT0042547)**B** *Tetramorium enkidu* (CASENT0045673)**C** *Tetramorium naganum* (CASENT0280584)**D** *Tetramorium gilgamesh* (CASENT0247312).

**Figure 3.**
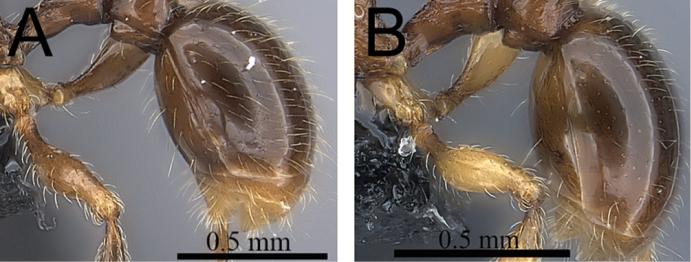
First gastral tergite in profile. **A** *Tetramorium alperti* (CASENT0042547) **B** *Tetramorium enkidu* (CASENT0039588).

**Figure 4.**
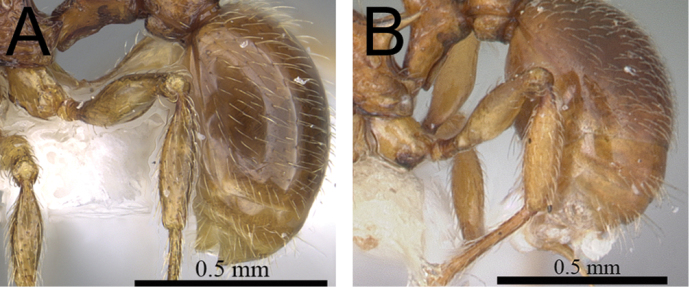
First gastral tergite in profile. **A** *Tetramorium gilgamesh* (CASENT0247312) **B** *Tetramorium naganum* (CASENT0102346).

##### Tetramorium alperti

Hita Garcia & Fisher sp. n.

http://zoobank.org/53D9615C-CF34-4216-B511-41081A7031B7

http://species-id.net/wiki/Tetramorium_alperti

**Figure 5.**
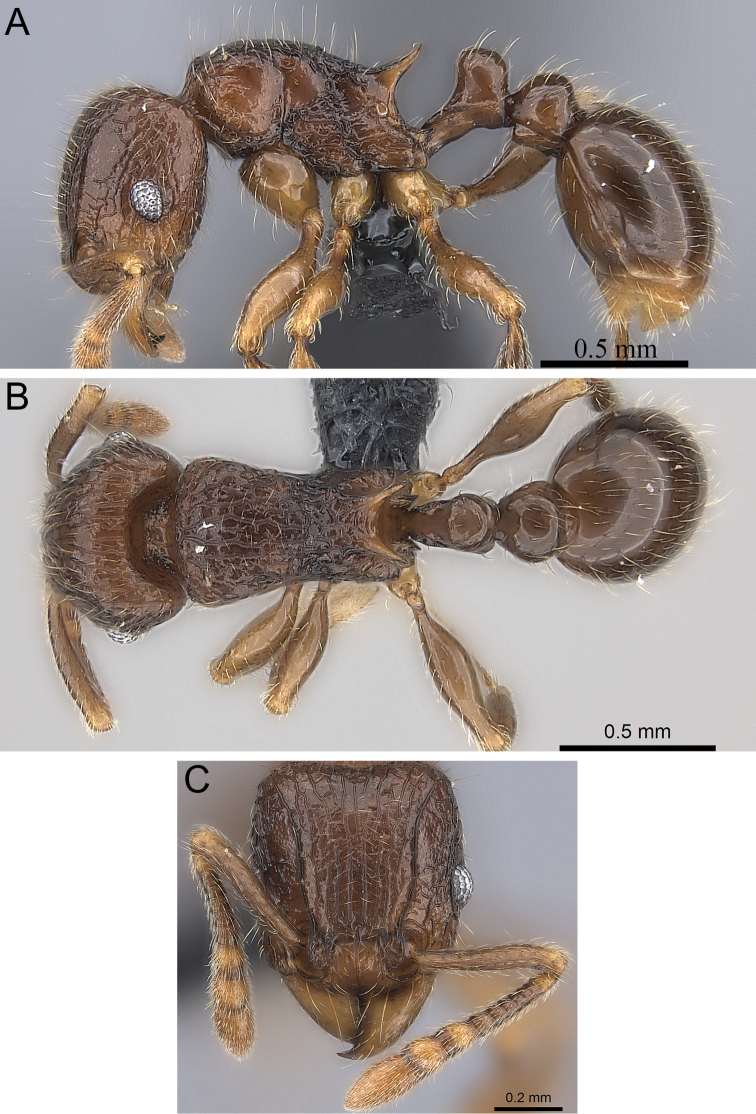
*Tetramorium alperti* holotype worker (CASENT0042547). **A** Body in profile **B** Body in dorsal view **C** Head in full-face view.

Figs 2A , 3A , 5 , 61

###### Type material.

**Holotype**, pinned worker, MADAGASCAR, Antsiranana, Parc National de Marojejy, Antranohofa, 26.6 km 31° NNE Andapa, 10.7 km 318° NW Manantenina, 14.44333°S, 49.74333°E, 1325 m, montane rainforest, sifted litter (leaf mould, rotten wood), collection code BLF09080, 18.XI.2003 (*B.L. Fisher*) (CAS: CASENT0042547). **Paratypes**, nine pinned workers with same data as holotype (BMNH: CASENT0042817; CAS: CASENT0042703; CASENT0042704; CASENT0042708; CASENT0042813; CASENT0042815; CASENT0042827; CASENT0042835; MCZ: CASENT0042821).

###### Non-type material.

MADAGASCAR: Antsiranana, Rés. Anjanaharibe-Sud, 9.2 km WSW Befingotra, 14.75°S, 49.46667°E, 1280 m, montane rainforest, 5.XI.1994 (*B.L. Fisher*); Fianarantsoa, Foret d’Ambalagoavy Nord, Ikongo, Ambatombe, 21.8275°S, 47.33889°E, 625 m, 1.XII.2000 (*R. Harin’Hala & M.E. Irwin*).

###### Diagnosis.

*Tetramorium alperti* differs from the other three species of the group by the following character combination: propodeal spines long to very long (PSLI 29–37); waist segments with several long erect hairs; first gastral tergite with moderately long, scattered, appressed to decumbent pubescence in combination with several much longer, erect standing hairs.

###### Worker measurements

**(N=10).** HL 0.56–0.68 (0.64); HW 0.54–0.65 (0.62); SL 0.40–0.47 (0.45); EL 0.13–0.15 (0.14); PH 0.31–0.38 (0.35); PW 0.40–0.50 (0.47); WL 0.70–0.84 (0.79); PSL 0.18–0.23 (0.21); PTL 0.15–0.18 (0.16); PTH 0.23–0.28 (0.26); PTW 0.16–0.19 (0.18); PPL 0.17–0.22 (0.20); PPH 0.23–0.27 (0.25); PPW 0.22–0.28 (0.26); CI 95–97 (96); SI 69–75 (73); OI 22–23 (23); DMI 57–62 (59); LMI 42–46 (44); PSLI 29–37 (33); PeNI 36–40 (38); LPeI 62–68 (64); DPeI 103–115 (108); PpNI 54–57 (55); LPpI 74–84 (80); DPpI 126–132 (128); PPI 139–156 (145).

###### Worker description.

Head longer than wide (CI 95–97); posterior head margin weakly concave. Anterior clypeal margin with distinct median impression. Frontal carinae strongly developed, diverging posteriorly and approaching or ending at posterior head margin; antennal scrobe present, but weak, shallow, and without defined posterior or ventral margins. Antennal scapes short, not reaching posterior head margin (SI 69–75). Eyes of moderate size (OI 22–23). Mesosomal outline in profile flat to weakly convex, relatively high (LMI 42–46), and moderately to strongly marginate from lateral to dorsal mesosoma; promesonotal suture and metanotal groove absent. Propodeal spines long to very long, spinose, acute, and often thick (PSLI 29–37); propodeal lobes short, triangular, and blunt or acute, always much shorter than propodeal spines. Petiolar node in profile high, rounded nodiform with moderately rounded antero- and posterodorsal margins, around 1.5 to 1.6 times higher than long (LPeI 62–68), anterior and posterior faces approximately parallel, anterodorsal and posterodorsal margins situated at about the same height and equally marginate, petiolar dorsum weakly convex; node in dorsal view around 1.0 to 1.1 times wider than long (DPeI 103–115), in dorsal view pronotum between 2.4 to 2.8 times wider than petiolar node (PeNI 36–40). Postpetiole in profile globular to subglobular, approximately 1.2 to 1.3 times higher than long (LPpI 74–84); in dorsal view around 1.2 to 1.3 times wider than long (DPpI 126–132), pronotum between 1.7 to 1.8 times wider than postpetiole (PpNI 54–57). Postpetiole in profile appearing less voluminous than petiolar node, postpetiole in dorsal view between 1.3 to 1.6 times wider than petiolar node (PPI 139–156). Mandibles usually mostly unsculptured and smooth with some weakly striate parts, generally very shiny; clypeus longitudinally rugose/rugulose, with three to five rugae/rugulae, median ruga always well developed and distinct, lateral rugae/rugulae sometimes weaker and interrupted; cephalic dorsum between frontal carinae with seven to nine longitudinal rugae, rugae running from posterior clypeal margin to posterior head margin, often interrupted or with cross-meshes, especially posteriorly; scrobal area mostly unsculptured; lateral head reticulate-rugose to longitudinally rugose. Ground sculpture on head absent to weakly punctate. Dorsum of mesosoma mostly irregularly longitudinally rugose; lateral mesosoma variably sculptured, lateral pronotum and anepisternum mostly unsculptured, smooth and shining with very little sculpture, usually only traces of rugulae present, katepisternum, metapleuron, and lateral propodeum noticeably irregularly longitudinally rugose. Forecoxae unsculptured, smooth and shining. Ground sculpture on mesosoma weak to absent. Waist segments and gaster completely unsculptured, smooth and shining. Whole body with numerous, long, and fine standing hairs; first gastral tergite with moderately long, relatively scattered, appressed to decumbent pubescence in combination with several much longer, fine, and erect hairs. Anterior edges of antennal scapes and dorsal (outer) surfaces of hind tibiae usually with decumbent to suberect hairs. Head, mesosoma, waist segments, and gaster orange-brown to chestnut brown, mandibles, antennae, and legs always lighter, usually yellowish brown.

###### Etymology.

The name of the new species is a patronym dedicated to Gary D. Alpert from Cambridge, Massachusetts, U.S.A., to honour his numerous ant collecting activities in Madagascar.

###### Distribution and biology.

This new species is only known from three localities (Fig. 61). Two are located in the northeast of Madagascar (Anjanaharibe-Sud and Marorejy) while the third is found much further south (Ambalagoavy). Anjanaharibe-Sud and Marorejy are montane forests ranging from 1280 to 1325 m elevation, whereas Ambalagoavy is located at 525 m. This distributional and elevational pattern suggests that *Tetramorium alperti* might have been distributed throughout most of the eastern Madagascar humid forests, but is now only found in a few montane forests and one lower rainforest site. Based on the available collection data, *Tetramorium alperti* seems to be a leaf litter inhabitant.

###### Discussion.

*Tetramorium alperti* cannot be confused with *Tetramorium dalek* since the latter has no long standing hairs on the waist segments and the first gastral tergite, while these are present in *Tetramorium alperti*. Differentiation from the other three species of the group, however, is more challenging. The primary diagnostic characters distinguishing *Tetramorium alperti* are the pilosity/pubescence pattern on the first gastral tergite and the shape of the petiolar node in profile. *Tetramorium alperti* possesses moderately long, relatively scattered, appressed to decumbent pubescence in combination with several much longer and erect hairs. This pattern on the first gastral tergite is not found in *Tetramorium enkidu*, *Tetramorium gilgamesh* or *Tetramorium naganum* since they either lack long and erect hairs or appressed to decumbent pubescence entirely. In addition, *Tetramorium alperti* also has a thicker petiolar node which is around 1.5 to 1.6 times higher than long (LPeI 62–68), compared to *Tetramorium gilgamesh* and *Tetramorium naganum*, in which the petiolar nodes are around 1.7 to 2.0 times higher than long (LPeI 50–59). The species probably most easily confused with *Tetramorium alperti* is *Tetramorium enkidu* since both share the same general habitus and the same morphometric range, and the only difference is the pattern of pilosity/pubescence on the first gastral tergite outlined above. Possibly they are conspecific and the differences in gastral pilosity/pubescence represent intraspecific variation, however, there are some good arguments to separate them as two distinct species. First, both species are found in sympatry in Marojejy, and they are easily identified by the differences in pilosity/pubescence mentioned above without any intermediate forms. Second, patterns of pilosity/pubescence are usually very species-specific within the genus *Tetramorium* (e.g. Bolton 1976, 1980; Hita Garcia et al. 2010; Hita Garcia and Fisher 2012a, 2012b). We were able to reveal several consistent patterns of pilosity/pubescence in the *Tetramorium naganum* species group, and the pattern of *Tetramorium alperti* is unique to this group, although it resembles the one seen in *Tetramorium ryanphelanae* Hita Garcia & Fisher from the *Tetramorium bessonii* species group, and a few species from the *Tetramorium schaufussii* species complex.

To our knowledge, there is no significant intraspecific variation in the material treated as *Tetramorium alperti*.

##### Tetramorium dalek

Hita Garcia & Fisher sp. n.

http://zoobank.org/F10C4841-A175-4D98-B3DD-19FBCC9F5E03

http://species-id.net/wiki/Tetramorium_dalek

Figs 1A , 6 , 61

###### Type material.

**Holotype**, MADAGASCAR, Toamasina, Montagne d’Anjanaharibe, 19.5 km 27° NNE Ambinanitelo, 15.1783°S, 49.635°E, 1100 m, montane rainforest, sifted litter (leaf mold, rotten wood), collection code BLF08150, 12.–16.III.2003 (*B.L. Fisher et al.*) (CAS: CASENT0038402). **Paratypes**, 17 pinned workers with same data as holotype (BMNH: CASENT0038394; CAS: CASENT0038369; CASENT0038372; CASENT0038376; CASENT0038390; CASENT0038408; CASENT0038413; CASENT0038416; CASENT0038425; CASENT0038429; CASENT0038443; CASENT0038445; CASENT0038450; CASENT0038456; CASENT0038465; MCZ: CASENT0038397; CASENT0038424); and seven workers from MADAGASCAR, Toamasina, Montagne d’Anjanaharibe, 18.0 km 21° NNE Ambinanitelo, 15.1883°S, 49.615°E, 470 m, rainforest, sifted litter (leaf mold, rotten wood), collection code BLF08802, 8.–12.III.2003 (*B.L. Fisher et al.*) (CAS: CASENT0037833; CASENT0037869; CASENT0037903; CASENT0037909; CASENT0037916; CASENT0037933; CASENT0037936).

**Figure 6.**
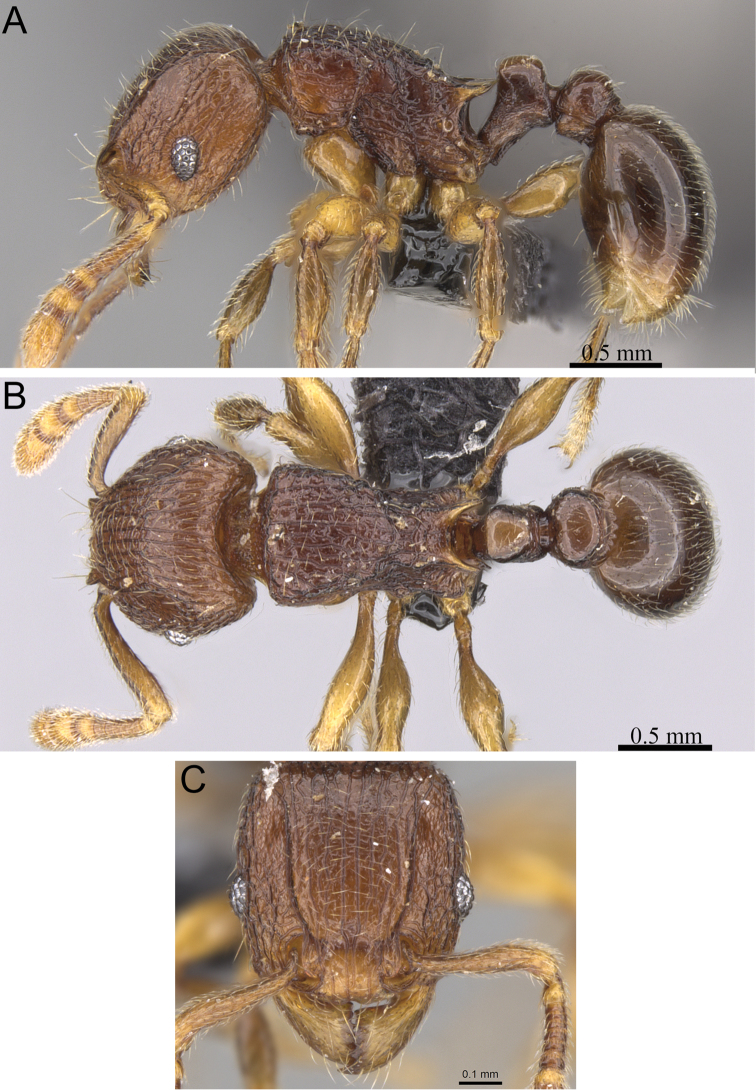
*Tetramorium dalek* holotype worker (CASENT0038402). **A** Body in profile **B** Body in dorsal view **C** Head in full-face view.

###### Non-type material.

MADAGASCAR: Antsiranana, 1 km W Andampibe, Cap Masoala, 15.69361°S, 50.18139°E, 125 m, lowland rainforest, 29.XI.1993 (*G.D. Alpert*); Antsiranana, Res. Anjanaharibe-Sud, 9.2 km WSW Befingotra, 14.75°S, 49.4667°E, 1280 m, 4.–9.XI.1994 (*B.L. Fisher*); Toamasina, Ambohitsitondroina, 6.9 km NE Ambanizana, 15.5851°S, 50.0095°E, 825 m, rainforest, 2.XII.1993 (*B.L. Fisher*); Toamasina, Andranobe, 6.3 km S Ambanizana, 15.6813°S, 49.958°E, 25 m, rainforest, 13.–14.XI.1993 (*B.L. Fisher*); Toamasina, Andranobe, 5.3 km SSE Ambanizana, 15.6713°S, 49.9739°E, 425 m, rainforest, 21.XI.1993 (*B.L. Fisher*); Toamasina, Montagne d’Anjanaharibe, 18.0 km 21° NNE Ambinanitelo, 15.1883°S, 49.615°E, 470 m, rainforest, 8.–12.III.2003 (*B.L. Fisher et al.*); Toamasina, Montagne d’Anjanaharibe, 19.5 km 27° NNE Ambinanitelo, 15.1783°S, 49.635°E, 1100 m, montane rainforest, 12.–16.III.2003 (*B.L. Fisher et al.*); Toamasina, Reserve Betampona, Camp Vohitsivalana, 37.1 km 338° Toamasina, 17.8867°S, 49.2025°E, 520 m, rainforest, 1.–3.XII.2005 (*B.L. Fisher et al.*); Toamasina, 19 km ESE Maroantsetra, 15.4833°S, 49.9°E, 350 m, rainforest, 22.IV.1989 (*P.S. Ward*); Toamasina, Montagne d’Akirindro 7.6 km 341° NNW Ambinanitelo, 15.2883°S, 49.5483°E, 600 m, rainforest, 17.–21.III.2003 (*B.L. Fisher et al.*); Toamasina, Nosy Mangabe, 15.5°S, 49.766667°E, 300 m, rainforest, 18.IV.1989 (*P.S. Ward*); Toamasina, Ile Sainte Marie, Forêt Ambohidena, 22.8 km 44° Ambodifotatra, 16.8243°S, 49.9642°E, 20 m, littoral rainforest, 21.XI.2005 (*B.L. Fisher et al.*); Toamasina, F.C. Sandranantitra, 18.0483°S, 49.0917°E, 450 m, rainforest, 21.–24.I.1999 (*H.J. Ratsirarson*); Toamasina, Parc National de Zahamena, Tetezambatana forest, near junction of Nosivola and Manakambahiny Rivers, 17.743°S, 48.7294°E, 860 m, rainforest, 18.–19.II.2009 (*B.L. Fisher et al.*); Toamasina, Parc National de Zahamena, Onibe River, 17.7591°S, 48.8547°E, 780 m, rainforest, 21.–23.II.2009 (*B.L. Fisher et al.*).

###### Diagnosis.

*Tetramorium dalek* is easily distinguishable by the following combination of characters: waist segments without long standing hairs, instead with short, appressed to subdecumbent pubescence only, sometimes with one or two short erect to suberect hairs; propodeal spines moderately long to long (PSLI 25–27); first gastral tergite with short, relatively dense, appressed to subdecumbent pubescence and without any standing hairs at all.

###### Worker measurements

**(N=12).** HL 0.47–0.56 (0.51); HW 0.45–0.54 (0.49); SL 0.31–0.35 (0.33); EL 0.11–0.13 (0.12); PH 0.23–0.30 (0.26); PW 0.34–0.39 (0.36); WL 0.54–0.68 (0.61); PSL 0.12–0.15 (0.13); PTL 0.12–0.14 (0.12); PTH 0.19–0.22 (0.20); PTW 0.13–0.17 (0.15); PPL 0.13–0.16 (0.15); PPH 0.19–0.21 (0.19); PPW 0.18–0.22 (0.20); CI 95–97 (96); SI 63–70 (67); OI 23–24 (24); DMI 54–63 (59); LMI 42–46 (43); PSLI 25–27 (26); PeNI 38–46 (42); LPeI 55–68 (61); DPeI 108–139 (123); PpNI 53–59 (56); LPpI 70–80 (76); DPpI 131–147 (139); PPI 126–138 (133).

###### Worker description.

Head longer than wide (CI 95–97); posterior head margin weakly concave. Anterior clypeal margin with distinct median impression. Frontal carinae strongly developed, diverging posteriorly, and usually approaching or ending at posterior head margin; antennal scrobe present, but weak, shallow, and without defined posterior or ventral margins. Antennal scapes short, not reaching posterior head margin (SI 63–70). Eyes of moderate size (OI 23–24). Mesosomal outline in profile flat to very weakly convex, relatively high (LMI 42–46), and moderately to strongly marginate from lateral to dorsal mesosoma; promesonotal suture and metanotal groove absent. Propodeal spines elongate-triangular to spinose, moderately long to long, and acute (PSLI 25–27); propodeal lobes short, triangular, and blunt or acute, always much shorter than propodeal spines. Petiolar node in profile high, rounded nodiform to weakly rectangular nodiform, with moderately rounded antero- and posterodorsal margins, around 1.5 to 1.8 times higher than long (LPeI 55–68), anterior and posterior faces approximately parallel, anterodorsal and posterodorsal margins situated at about the same height and equally marginate (very rarely posterodorsal margin more rounded and lower than anterodorsal margin), petiolar dorsum flat to very weakly convex; node in dorsal view around 1.1 to 1.4 times wider than long (DPeI 108–139), in dorsal view pronotum between 2.2 to 2.6 times wider than petiolar node (PeNI 38–46). Postpetiole in profile globular, approximately 1.2 to 1.4 times higher than long (LPpI 70–80); in dorsal view around 1.3 to 1.5 times wider than long (DPpI 131–147), pronotum between 1.7 to 1.9 times wider than postpetiole (PpNI 53–59). Postpetiole in profile appearing slightly more voluminous than petiolar node, postpetiole in dorsal view around 1.3 to 1.4 times wider than petiolar node (PPI 126–138). Mandibles distinctly striate; clypeus longitudinally rugose/rugulose, with three to five rugae/rugulae, median ruga always well developed and distinct, lateral rugae/rugulae sometimes weaker and interrupted; cephalic dorsum between frontal carinae with seven to ten longitudinal rugae, rugae running from posterior clypeal margin to posterior head margin, often interrupted or with cross-meshes, especially posteriorly; scrobal area mostly unsculptured; lateral head longitudinally rugose to reticulate-rugose. Ground sculpture on head absent to weakly punctate. Mesosoma laterally and dorsally mostly irregularly longitudinally rugose. Forecoxae unsculptured, smooth and shining. Ground sculpture on mesosoma weak to absent. Waist segments and gaster completely unsculptured, smooth and shining. Head and mesosoma with numerous, moderately long and fine standing hairs; waist segments and first gastral tergite with short, comparatively dense, appressed to subdecumbent pubescence, sometimes several of these short hairs suberect to erect. Anterior edges of antennal scapes and dorsal (outer) surfaces of hind tibiae usually with decumbent to suberect hairs. Head, mesosoma, waist segments, and gaster generally orange-brown to chestnut brown, mandibles, antennae, and legs always lighter, usually yellowish brown.

###### Etymology.

The name of the new species is taken from the popular British TV show “Dr. Who” and refers to a fictional, extra-terrestrial race of evil mutants. During different stages of the revision we considered placing the material listed here as *Tetramorium dalek* in at least three to four different groups, which caused a significant amount of nuisance, especially to the first author. Naming this species after an evil, extra-terrestrial, and often annoying race was a logical consequence. The species epithet is an arbitrary combination of letters, thus invariant.

###### Distribution and biology.

The new species is found in the lowland and montane rainforests of eastern Madagascar from the southernmost localities Sandranantitra, Betampona, and Zahamena, north to Anjanaharibe-Sud (Fig. 61). In addition, *Tetramorium dalek* has an elevational range from 20 to 1280 m, and seems to live in leaf litter.

###### Discussion.

*Tetramorium dalek* is easily distinguishable within the *Tetramorium naganum* species group since it is the only species without long, standing hairs on the waist segments and the first gastral tergite, and in addition, it also has generally shorter propodeal spines (PSLI 25–27) than the other four species (PSLI 27–37). But it is possible to mistake *Tetramorium dalek* with species from other groups. Its general habitus and in particular its lack of standing pilosity on the first gastral tergite could lead to misplacement in the *Tetramorium schaufussii* complex of the *Tetramorium schaufussii* species group or the *Tetramorium ibycterum* complex in the *Tetramorium ranarum* group. Indeed, a misidentification with one species from the latter complex is likely. *Tetramorium ibycterum* superficially shares many characters with *Tetramorium dalek*, and even most morphometric ranges. However, *Tetramorium ibycterum* has very well developed antennal scrobes with clearly defined margins all around, whereas *Tetramorium dalek* has weaker antennal scrobes without clearly defined margins all around. In addition, *Tetramorium dalek* differs from the species of the *Tetramorium schaufussii* complex in having a broader head (CI 95–97) and higher and stouter mesosoma (LMI 42–46). Consequently, despite morphological similarities to other species groups, we consider *Tetramorium dalek* best placed in the *Tetramorium naganum* group.

##### Tetramorium enkidu

Hita Garcia & Fisher sp. n.

http://zoobank.org/CEF594E4-5B03-42F9-B250-4295A137AAC6

http://species-id.net/wiki/Tetramorium_enkidu

**Figure 7.**
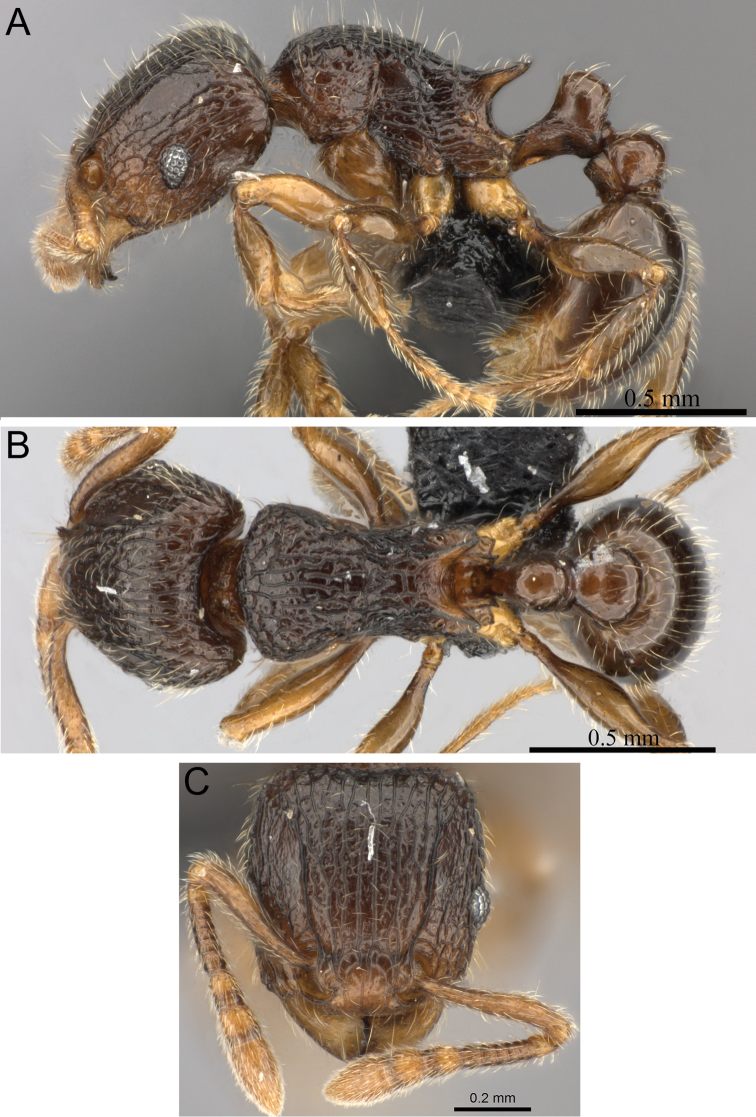
*Tetramorium enkidu* holotype worker (CASENT0056450). **A** Body in profile **B** Body in dorsal view **C** Head in full-face view.

Figs 2B , 3B , 7 , 61

###### Type material.

**Holotype**, pinned worker, MADAGASCAR, Antsiranana, Forêt Ambanitaza, 26.1 km 347° Antalaha, 14.67933°S, 50.18367°E, 240 m, rainforest, sifted litter (leaf mold, rotten wood), collection code BLF10997, 26.XI.2004 (*B.L. Fisher*) (CAS: CASENT0056450). **Paratypes**, ten pinned workers with same data as holotype (BMNH: CASENT0056445; CAS: CASENT0056435; CASENT0056436; CASENT0056437; CASENT0056441; CASENT0056448; CASENT0056449; CASENT0056456; CASENT0056467; MCZ: CASENT0056461).

###### Non-type material.

Antsiranana, Forêt Ambanitaza, 26.1 km 347° Antalaha, 14.6793°S, 50.1837°E, 240 m, rainforest, 26.XI.2004 (*B.L. Fisher*); Antsiranana, 1 km W Andampibe, Cap Masoala, 15.6936°S, 50.1814°E, 125 m, lowland rainforest, 1.XII.1993 (*G.D. Alpert*); Antsiranana, Forêt de Binara, 9.4km 235° SW Daraina, 13.2633°S, 49.6°E, 1100 m, montane rainforest, 5.XII.2003 (*B.L. Fisher*); Antsiranana, Parc National Montagne d’Ambre, 3.6 km 235° SW Joffreville, 12.5344°S, 49.1795°E, 925 m, montane rainforest, 20.–26.I.2001 (*B.L. Fisher et al.*); Antsiranana, Parc National de Marojejy, Manantenina River, 27.6 km 35° NE Andapa, 9.6 km 327° NNW Manantenina, 14.435°S, 49.76°E, 775 m, 15.–18.XI.2003 (*B.L. Fisher et al.*); Toamasina, Montagne d’Akirindro, 7.6 km 341° NNW Ambinanitelo, 15.2883°S, 49.5483°E, 600 m, rainforest, 17.–21.III.2003 (*B.L. Fisher et al.*); Toamasina, 19 km ESE Maroantsetra, 15.4833°S, 49.9°E, 350 m, 22.IV.1989 (*P.S. Ward*).

###### Diagnosis.

*Tetramorium enkidu* is distinguishable from the other species of the group by the following combination of characters: eyes small to moderate in size (OI 22–24); waist segments with several long erect hairs; propodeal spines long to very long (PSLI 29–36); in profile petiolar node relatively thick, between 1.5 and 1.7 times higher than long (LPeI 60–65); first gastral tergite with moderately short, abundant, subdecumbent to suberect pilosity, and without short, dense, appressed to decumbent pubescence.

###### Worker measurements

**(N=12).** HL 0.53–0.62 (0.58); HW 0.50–0.60 (0.56); SL 0.33–0.43 (0.39); EL 0.11–0.14 (0.13); PH 0.25–0.34 (0.31); PW 0.36–0.46 (0.43); WL 0.59–0.77 (0.71); PSL 0.16–0.21 (0.19); PTL 0.13–0.16 (0.15); PTH 0.21–0.26 (0.24); PTW 0.14–0.18 (0.17); PPL 0.15–0.21 (0.19); PPH 0.20–0.26 (0.24); PPW 0.21–0.26 (0.24); CI 93–98 (96); SI 64–72 (69); OI 22–24 (23); DMI 58–62 (60); LMI 41–45 (43); PSLI 29–36 (32); PeNI 35–42 (39); LPeI 60–65 (63); DPeI 104–113 (109); PpNI 54–60 (57); LPpI 73–85 (79); DPpI 120–140 (131); PPI 142–155 (147).

###### Worker description.

Head weakly to distinctly longer than wide (CI 93–98); posterior head margin weakly concave. Anterior clypeal margin with distinct median impression. Frontal carinae strongly developed, diverging posteriorly, and usually approaching or ending at posterior head margin; antennal scrobe present, but weak, shallow, and without defined posterior or ventral margins. Antennal scapes very short, not reaching posterior head margin (SI 64–72). Eyes short to moderate (OI 22–24). Mesosomal outline in profile weakly convex, relatively high (LMI 41–45), and moderately to strongly marginate from lateral to dorsal mesosoma; promesonotal suture and metanotal groove absent. Propodeal spines spinose, long to very long, and acute (PSLI 29–36); propodeal lobes short, triangular, and blunt or acute, always much shorter than propodeal spines. Petiolar node in profile high, rounded nodiform, with well-rounded antero- and posterodorsal margins, around 1.5 to 1.7 times higher than long (LPeI 60–65), anterior and posterior faces approximately parallel, anterodorsal and posterodorsal margins situated at about the same height and equally marginate, petiolar dorsum always distinctly convex; node in dorsal view slightly wider than long (DPeI 104–113), in dorsal view pronotum between 2.4 to 2.8 times wider than petiolar node (PeNI 36–42). Postpetiole in profile globular, approximately 1.2 to 1.4 times higher than long (LPpI 73–85); in dorsal view between 1.2 to 1.4 times wider than long (DPpI 120–140), pronotum around 1.7 to 1.8 times wider than postpetiole (PpNI 54–60). Postpetiole in profile appearing slightly lower and thicker than petiolar node, postpetiole in dorsal view around 1.4 to 1.5 times wider than petiolar node (PPI 142–155). Mandibles usually unsculptured, smooth, and shining, sometimes weakly partially striate (especially basally), rarely fully covered in fine striations; clypeus longitudinally rugose/rugulose, with three to six rugae/rugulae, median ruga always well developed and distinct, lateral rugae/rugulae usually weaker and/or interrupted; cephalic dorsum between frontal carinae with seven to nine longitudinal rugae, rugae running from posterior clypeal margin to posterior head margin, often irregular, interrupted or with cross-meshes, especially posteriorly; scrobal area mostly unsculptured; lateral head longitudinally rugose to reticulate-rugose. Ground sculpture on head weak to absent. Mesosoma laterally and dorsally irregularly longitudinally rugose, rarely lateral mesosoma with few unsculptured areas. Forecoxae unsculptured, smooth and shining. Ground sculpture on mesosoma very weak to absent. Waist segments and gaster completely unsculptured, smooth and shining. Head, mesosoma, and waist segments with numerous, long, and fine standing hairs; first gastral tergite with moderately short, abundant, subdecumbent to suberect pilosity, and without short, dense, appressed to decumbent pubescence; pilosity appearing disorganized due to varying degrees of inclination. Anterior edges of antennal scapes and dorsal (outer) surfaces of hind tibiae with decumbent to suberect hairs. Head, mesosoma, waist segments, and gaster light to dark brown, mandibles, antennae, and legs always of lighter brown.

###### Etymology.

The new species is named after the fictional character “Enkidu” who is a central figure in the ancient Mesopotamian poem “Epic of Gilgamesh”, one of the oldest written stories in human history. The species epithet is an arbitrary combination of letters, thus invariant.

###### Distribution and biology.

The new species is restricted to the northern part of Madagascar. Its distribution ranges from Montagne d’Akirindro, the area around Maroantsetra, and Cap Masoala north through Ambanitaza, and Marojejy to Binara and Montagne d’Ambre (Fig. 61). The localities are rainforests or montane rainforests situated at altitudes from 125 to 1100. In addition, *Tetramorium enkidu* appears to live in leaf litter or the ground.

###### Discussion.

*Tetramorium enkidu* is easily identifiable within the species group. The presence of several long, erect hairs on the waist segments and much longer propodeal spines (PSLI 29–36) separate *Tetramorium enkidu* from *Tetramorium dalek* (PSLI 25–27). The latter species and *Tetramorium naganum* both lack standing pilosity on the first gastral tergite, which is present in *Tetramorium enkidu*. *Tetramorium naganum* also has a thinner petiolar node, which is between 1.7 to 1.9 times higher than long (LPeI 54–58), contrasting with the thicker node of *Tetramorium enkidu*, which is between 1.5 and 1.7 times higher than long (LPeI 60–65). Also distinguishable from *Tetramorium enkidu* by a relatively thin petiolar node is *Tetramorium gilgamesh* (LPeI 50–58). The latter also possesses larger eyes (OI 25–27) than *Tetramorium enkidu* (OI 22–24), but this can be difficult to see without measuring. The last species of the group, *Tetramorium alperti*, shares the thicker petiolar node shape with *Tetramorium enkidu*, as well as most other characters except gastral pilosity. Indeed, as outlined in the description of *Tetramorium alperti*, both could be easily combined into one species since their separation is based only on differences in gastral pilosity/pubescence. However, we prefer to describe them as distinct because their distribution ranges overlap and both maintain a species-specific pattern of gastral pilosity/pubescence in sympatry.

##### Tetramorium gilgamesh

Hita Garcia & Fisher sp. n.

http://zoobank.org/24769905-BB87-467D-8785-93586E3E8E65

http://species-id.net/wiki/Tetramorium_gilgamesh

**Figure 8.**
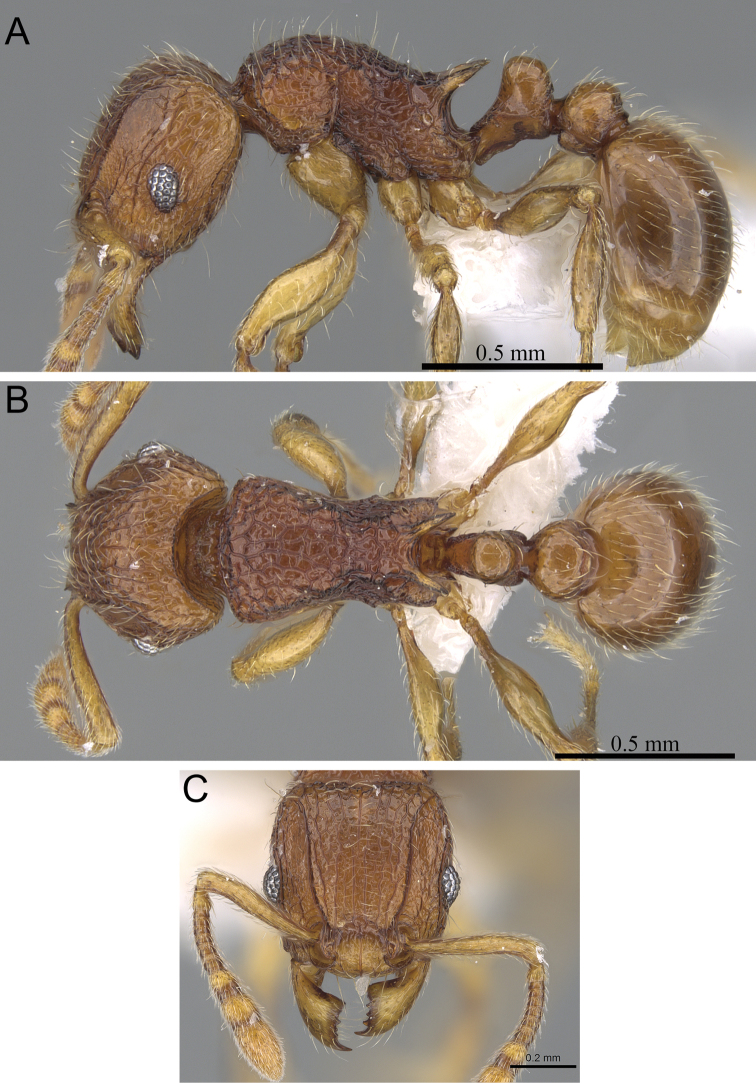
*Tetramorium gilgamesh* holotype worker (CASENT0247312). **A** Body in profile **B** Body in dorsal view **C** Head in full-face view.

Figs 2D , 4A , 8 , 61

###### Type material.

**Holotype**, pinned worker, MADAGASCAR, Toamasina, F.C. Sandranantitra, 18.0483°S, 49.0917°E, 450 m, rainforest, sifted litter (leaf mold, rotten wood), collection code HJR101, 18.–21.I.1999 (*H.J. Ratsirarson*) (CAS: CASENT0247312). **Paratypes**, four pinned workers with same data as holotype (CAS: CASENT0189097; CASENT0218015); and three pinned workers with same data as holotype except collected from the 21.–24.I.1999 and collection code HJR102 (CAS: CASENT0189096; CASENT0218016).

###### Non-type material.

MADAGASCAR: Antsiranana, 1 km W Andampibe, Cap Masoala, 15.6936°S, 50.1814°E, 125 m, lowland rainforest, 1.XII.1993 (*G.D. Alpert*); Toamasina, Montagne d’Akirindro 7.6 km 341° NNW Ambinanitelo, 15.2883°S, 49.5483°E, 600 m, rainforest, 17.–21.III.2003 (*B.L. Fisher et al.*); Toamasina, Andranobe, 5.3 km SSE Ambanizana, 15.6713°S, 49.9739°E, 425 m, rainforest, 21.XI.1993 (*B.L. Fisher*); Toamasina, Réserve Spéciale Ambatovaky, Sandrangato river, 16.7727°S, 49.2655°E, 450 m, rainforest, 20.–22.II.2010 (*B.L. Fisher et al.*); Toamasina, Réserve Spéciale Ambatovaky, Sandrangato river, 16.7633°S, 49.2669°E, 520 m, rainforest, 22.II.2010 (*B.L. Fisher et al.*); Toamasina, Réserve Spéciale Ambatovaky, Sandrangato river, 16.8175°S, 49.295°E, 360 m, rainforest, 25.–27.II.2010 (*B.L. Fisher et al.*); Toamasina, F.C. Andriantantely, 18.695°S, 48.8133°E, 530 m, rainforest, 7.–10.XII.1998 (*H.J. Ratsirarson*); Toamasina, Montagne d’Anjanaharibe, 18.0 km 21° NNE Ambinanitelo, 15.1883°S, 49.615°E, 470 m, rainforest, 8.–12.III.2003 (*B.L. Fisher et al.*); Toamasina, Reserve Betampona, Camp Vohitsivalana, 37.1 km 338° Toamasina, 17.8867°S, 49.2025°E, 520 m, rainforest, 1.–3.XII.2005 (*B.L. Fisher et al.*); Toamasina, Parc National Mananara-Nord, 7.1 km 261° Antanambe, 16.455°S, 49.7875°E, 225 m, rainforest, 14.XI.2005 (*B.L. Fisher et al.*); Toamasina, F.C. Sandranantitra, 18.0483°S, 49.0917°E, 450 m, rainforest, 18.–24.I.1999 (*H.J. Ratsirarson*).

###### Diagnosis.

The following character combination distinguishes *Tetramorium gilgamesh* from the other members of the *Tetramorium naganum* group: relatively large eyes (OI 25–27); propodeal spines long (PSLI 27–30); petiolar node relatively high and thin, around 1.7 to 2.0 times higher than long (LPeI 50–59); waist segments with several long erect hairs; first gastral tergite with short to moderately long, abundant, decumbent to suberect pilosity, and without short, dense, appressed to subdecumbent pubescence; pilosity appearing disorganized due to varying degrees of inclination and hair length.

###### Worker measurements

**(N=12).** HL 0.52–0.56 (0.54); HW 0.49–0.55 (0.51); SL 0.36–0.38 (0.37); EL 0.13–0.14 (0.13); PH 0.26–0.30 (0.27); PW 0.36–0.41 (0.38); WL 0.61–0.66 (0.64); PSL 0.14–0.17 (0.15); PTL 0.11–0.14 (0.12); PTH 0.21–0.24 (0.22); PTW 0.13–0.17 (0.14); PPL 0.15–0.18 (0.16); PPH 0.19–0.22 (0.20); PPW 0.20–0.24 (0.22); CI 92–98 (94); SI 68–76 (73); OI 25–27 (26); DMI 58–62 (59); LMI 40–45 (42); PSLI 27–30 (28); PeNI 33–40 (37); LPeI 50–59 (55); DPeI 112–127 (118); PpNI 54–59 (56); LPpI 74–85 (80); DPpI 125–142 (132); PPI 143–169 (153).

###### Worker description.

Head weakly to distinctly longer than wide (CI 92–98); posterior head margin weakly concave. Anterior clypeal margin with distinct median impression. Frontal carinae strongly developed, diverging posteriorly, and usually approaching or ending at posterior head margin; antennal scrobe distinct but relatively shallow, usually with defined margins all around, but ventral margin sometimes poorly defined and merging with surrounding sculpture, median scrobal carina generally not well developed, if present relatively weak and reaching eye level only. Antennal scapes short, not reaching posterior head margin (SI 68–76). Eyes relatively large (OI 25–27). Mesosomal outline in profile weakly convex, relatively high (LMI 40–45), and moderately to strongly marginate from lateral to dorsal mesosoma; promesonotal suture and metanotal groove absent. Propodeal spines spinose, long, and acute (PSLI 27–30); propodeal lobes short, triangular, and blunt or acute, always much shorter than propodeal spines. Petiolar node in profile high, rounded nodiform, with well-rounded antero- and posterodorsal margins, around 1.7 to 2.0 times higher than long (LPeI 50–59), anterior and posterior faces not parallel, node slightly narrowing from base to apex, usually anterodorsal and posterodorsal margins situated at about same height (very rarely anterodorsal margin weakly higher than posterodorsal margin), petiolar dorsum weakly to moderately convex; node in dorsal view around 1.1 to 1.3 times wider than long (DPeI 112–127), in dorsal view pronotum between 2.5 to 3.0 times wider than petiolar node (PeNI 33–40). Postpetiole in profile globular, approximately 1.2 to 1.3 times higher than long (LPpI 74–85); in dorsal view between 1.2 to 1.4 times wider than long (DPpI 125–142), pronotum between 1.7 to 1.9 times wider than postpetiole (PpNI 54–59). Postpetiole in profile appearing thicker and lower than petiolar node, postpetiole in dorsal view around 1.4 to 1.7 times wider than petiolar node (PPI 143–169). Mandibles usually unsculptured, smooth, and shining, rarely with traces of longitudinal rugulae; clypeus longitudinally rugose/rugulose, with three to six rugae/rugulae, median ruga always well developed and distinct, lateral rugae/rugulae usually weaker and/or interrupted; cephalic dorsum between frontal carinae with eight to nine longitudinal rugae, rugae running from posterior clypeal margin to posterior head margin, some rugae irregular, interrupted or with cross-meshes, especially posteriorly; scrobal area mostly unsculptured, smooth and shining; lateral head mostly longitudinally rugose to reticulate-rugose, but with extensive unsculptured areas. Ground sculpture on head absent to weakly punctate. Mesosoma laterally and dorsally irregularly longitudinally rugose, sometimes lateral mesosoma with a few unsculptured areas medially. Forecoxae unsculptured, smooth and shining. Ground sculpture on mesosoma usually weak to absent. Waist segments and gaster completely unsculptured, smooth and shining. Head, mesosoma, and waist segments with numerous, long, and fine standing hairs; first gastral tergite with short to moderately long, abundant, decumbent to suberect pilosity, without short, dense, appressed to subdecumbent pubescence; pilosity appearing disorganized due to varying degrees of inclination and hair length. Anterior edges of antennal scapes and dorsal (outer) surfaces of hind tibiae with decumbent to subdecumbent hairs. Head, mesosoma, waist segments, and gaster usually orange to light brown, rarely of darker brown, mandibles, antennae, and legs usually lighter, yellowish brown.

###### Etymology.

The new species is named after the fictional character “Gilgamesh”, the main figure in the ancient Mesopotamian poem “Epic of Gilgamesh”, one of the earliest surviving works of literature. The species epithet is an arbitrary combination of letters, thus invariant.

###### Distribution and biology.

*Tetramorium gilgamesh* is distributed in eastern Madagascar (Fig. 61). Its distribution ranges from the southernmost known locality Andriantantely north to Montagne d’Anjanaharibe and Montagne d’Akirindro, and northeast to Andranobe and Andampibe on the Masoala Peninsula. All localities are lowland rainforests situated at elevations of 125 to 600 m. The preferred microhabitat of *Tetramorium gilgamesh* seems to be leaf litter.

###### Discussion.

The identification of *Tetramorium gilgamesh* within the *Tetramorium naganum* species group is fairly straightforward. The best diagnostic character is eye size since *Tetramorium gilgamesh* has the largest eyes of the group with an OI 25–27 (vs. OI 21–24 in the other four species). Beyond this, it cannot be mistaken for *Tetramorium dalek* since the waist segments and a first gastral tergite of the latter species are covered with short, comparatively dense, appressed to subdecumbent pubescence without standing pilosity. By contrast, *Tetramorium gilgamesh* has long, erect hairs on the waist segments and the first gastral tergite is covered with short to moderately long, abundant, decumbent to suberect pilosity, but without short, dense, appressed to subdecumbent pubescence. Additionally, the pilosity on the first gastral tergite of *Tetramorium gilgamesh* appears disorganized due to varying degrees of inclination and hair length. The gastral pilosity separates it also from *Tetramorium alperti*, *Tetramorium enkidu*, and *Tetramorium naganum*, which have very different patterns of pilosity/pubescence. Furthermore, *Tetramorium alperti* and *Tetramorium enkidu* have thicker petiolar nodes (LPeI 60–68) than *Tetramorium gilgamesh* (LPeI 50–59). *Tetramorium naganum* shares the same shape of the petiolar node with *Tetramorium gilgamesh*, and both are also found in sympatry throughout most of their distribution ranges. However, differences in eye size and gastral pilosity distinguish between both species fairly well.

On the basis of the available material it seems that intraspecific variation is generally low in *Tetramorium gilgamesh*.

##### Tetramorium naganum

Bolton

http://species-id.net/wiki/Tetramorium_naganum

**Figure 9.**
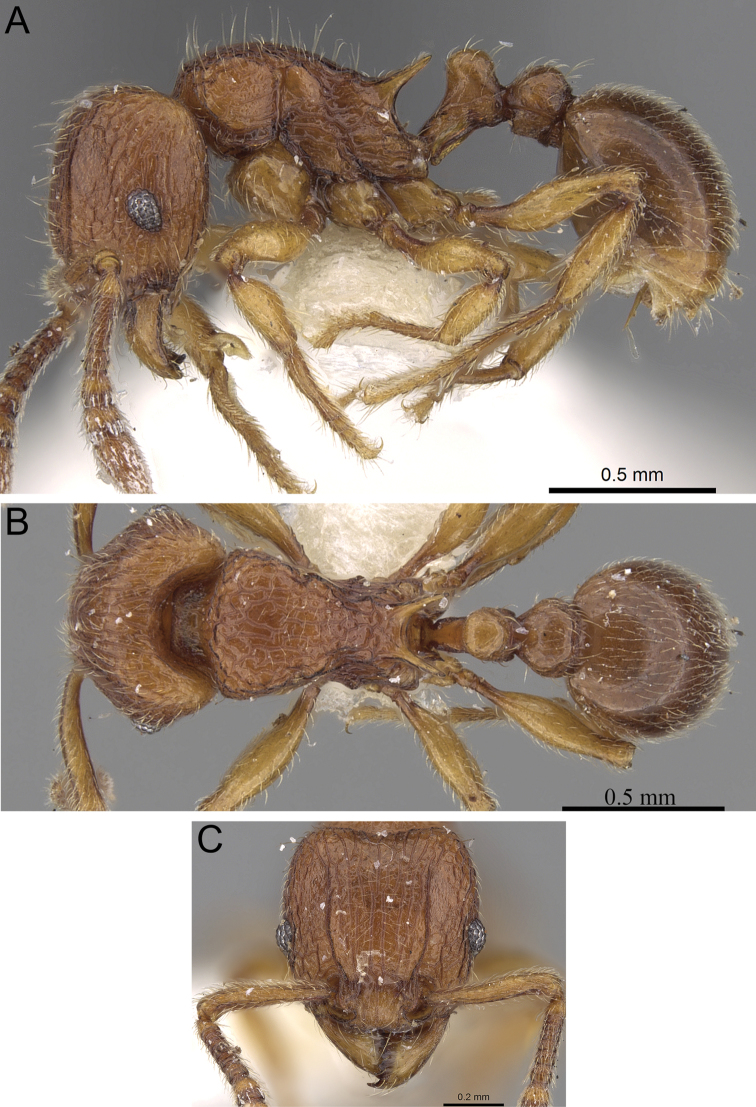
*Tetramorium naganum* holotype worker (CASENT0280584). **A** Body in profile **B** Body in dorsal view **C** Head in full-face view.

Figs 1B , 2C , 4B , 9 , 61

Tetramorium naganum Bolton, 1979: 150.

###### Type material.

**Holotype**, pinned worker, MADAGASCAR, Toamasina, La Mandraka, 18.912778°S, 47.892222°E 1280 m, montane forest, collection code AB41, 8.II.1977 (*W.L. & D.E. Brown*) (MCZ: MCZ_Holotype_32379) [examined]. Paratypes, 16 pinned workers and one dealate queen with same data as holotype (BMNH: CASENT0102346; CASENT0235212; MCZ: MCZ_Paratype_32379) [examined].

[Note: the GPS data of the type locality was not provided by the locality label or the original description. The data presented above is based on our own georeferencing of the rainforest locality La Mandraka and should be considered an approximation and not the exact location of the type locality of *Tetramorium naganum*.]

###### Non-type material.

MADAGASCAR: Antsiranana, Rés. Anjanaharibe-Sud, 6.5 km SSW Befingotra, 14.75°S, 49.5°E, 875 m, rainforest, 19.X.1994 (*B.L. Fisher*); Antsiranana, Rés. Anjanaharibe-Sud, 9.2 km WSW Befingotra, 14.75°S, 49.4667°E, 1280 m, montane rainforest, 5.XI.1994 (*B.L. Fisher*); Antananarivo, 3 km 41° NE Andranomay, 11.5 km 147° SSE Anjozorobe, 18.4733°S, 47.96°E, 1300 m, 5.–13.XII.2000 (*B.L. Fisher et al.*); Toamasina, 6.9 km NE Ambanizana, Ambohitsitondroina, 15.5851°S, 50.0095°E, 825 m, rainforest, 2.XII.1993 (*B.L. Fisher*); Toamasina, Ambanizana, Parc National Masoala, 15.5722°S, 50.0069°E, 1020 m, montane rainforest, 2.–6.III.2003 (*D. Andriamalala et al.*); Toamasina, Ambatoharanana, Corridor Forestier Analamay-Mantadia, 18.8042°S, 48.4008°E, 968 m, 12.–19.XII.2012 (*B.L. Fisher et al.*); Toamasina, Analamay, 18.8062°S, 48.3371°E, 1068 m, montane rainforest, 21.III.2004 (*B.L. Fisher*); Toamasina, Ankerana, 18.4017°S, 48.806°E, 1035 m, montane forest, 24.–29.I.2012 (*B.L. Fisher et al.*); Toamasina, Montagne d’Anjanaharibe, 19.5 km 27° NNE Ambinanitelo, 15.17833°S, 49.635°E, 1100 m, montane rainforest, 12.–16.III.2003 (*B.L. Fisher*).

###### Diagnosis.

*Tetramorium naganum* can be easily diagnosed within the *Tetramorium naganum* group on the basis of the following character combination: eyes small to moderate in size (OI 21–23); propodeal spines relatively long (PSLI 28–33); petiolar node in profile relatively thin, between 1.7 to 1.9 times higher than long (LPeI 54–58); waist segments with long, standing hairs; first gastral tergite with short, comparatively dense, appressed to decumbent pubescence, and without any long standing hairs.

###### Worker measurements

**(N=10).** HL 0.57–0.72 (0.63); HW 0.59–0.73 (0.65); SL 0.39–0.51 (0.46); EL 0.13–0.16 (0.14); PH 0.29–0.40 (0.33); PW 0.41–0.53 (0.45); WL 0.73–0.92 (0.78); PSL 0.18–0.22 (0.20); PTL 0.13–0.16 (0.14); PTH 0.24–0.28 (0.25); PTW 0.15–0.19 (0.16); PPL 0.18–0.22 (0.19); PPH 0.23–0.27 (0.24); PPW 0.23–0.28 (0.25); CI 94–99 (97); SI 68–77 (73); OI 21–23 (22); DMI 56–62 (58); LMI 40–45 (43); PSLI 28–33 (30); PeNI 34–38 (35); LPeI 54–58 (56); DPeI 107–122 (115); PpNI 52–59 (54); LPpI 77–84 (80); DPpI 125–137 (128); PPI 147–159 (153).

###### Worker description.

Head weakly to distinctly longer than wide (CI 94–99); posterior head margin moderately concave. Anterior clypeal margin with distinct median impression. Frontal carinae strongly developed, diverging posteriorly, and usually approaching or ending at posterior head margin; antennal scrobe present, but weak, shallow, and without defined posterior or ventral margins. Antennal scapes short, not reaching posterior head margin (SI 68–77). Eyes short to moderate (OI 21–23). Mesosomal outline in profile weakly convex, relatively high (LMI 40–45), and moderately to strongly marginate from lateral to dorsal mesosoma; promesonotal suture and metanotal groove absent. Propodeal spines spinose, long, and acute (PSLI 28–33); propodeal lobes short, triangular, and blunt or acute, always much shorter than propodeal spines. Petiolar node in profile high, rounded nodiform, with well-rounded antero- and posterodorsal margins, between 1.7 to 1.9 times higher than long (LPeI 54–58), anterior and posterior faces approximately parallel, anterodorsal and posterodorsal margins situated at about the same height (very rarely anterodorsal margin higher than posterodorsal margin) and equally marginate, petiolar dorsum always convex; node in dorsal view around 1.1 to 1.2 times wider than long (DPeI 107–122), in dorsal view pronotum between 2.7 to 3.0 times wider than petiolar node (PeNI 34–38). Postpetiole in profile globular, approximately 1.2 to 1.3 times higher than long (LPpI 77–84); in dorsal view between 1.2 to 1.4 times wider than long (DPpI 125–137), pronotum around 1.7 to 1.9 times wider than postpetiole (PpNI 52–59). Postpetiole in profile thicker and lower than petiolar node, postpetiole in dorsal view around 1.5 to 1.6 times wider than petiolar node (PPI 147–159). Mandibles variably sculptured, ranging from fully unsculptured, smooth, and shining through partially striate to fully striate; clypeus longitudinally rugose/rugulose, with two to six rugae/rugulae, median ruga always well developed and distinct, lateral rugae/rugulae usually weaker and/or interrupted; cephalic dorsum between frontal carinae with six to nine longitudinal rugae, rugae running from posterior clypeal margin to posterior head margin but very often irregular, interrupted or with cross-meshes, especially posteriorly; scrobal area usually mostly unsculptured, rarely longitudinally rugose to reticulate-rugose; lateral head longitudinally rugose to reticulate-rugose. Ground sculpture on head absent to weakly punctate. Mesosoma laterally and dorsally irregularly longitudinally rugose, rarely lateral mesosoma with a few unsculptured areas medially. Forecoxae unsculptured, smooth and shining. Ground sculpture on mesosoma very weak to absent. Waist segments and gaster completely unsculptured, smooth and shining. Head, mesosoma, and waist segments with numerous, long, and fine standing hairs; first gastral tergite with short, comparatively dense, appressed to subdecumbent pubescence. Anterior edges of antennal scapes and dorsal (outer) surfaces of hind tibiae usually with decumbent to suberect hairs. Head, mesosoma, waist segments, and gaster usually orange to light brown, rarely of darker brown, mandibles, antennae, and legs always lighter, usually light yellowish brown.

###### Distribution and biology.

*Tetramorium naganum* is found in rainforests or montane rainforests in eastern and north-eastern Madagascar (Fig. 61). The distribution range is disjunctive, very much as in *Tetramorium alperti* and *Tetramorium dalek*. *Tetramorium naganum* is either found in the region around La Mandraka, Analamay, Andranomay, and Andasibe-Mantadia, and Ankerana, or much further north between Anjanaharibe-Sud and the Masoala Peninsula (Ambanizana), with Montagne d’Anjanaharibe being in-between. The reasons behind this discontinuous distribution are not clear yet, but as for *Tetramorium alperti*, it is likely that *Tetramorium naganum* was previously much more common in eastern Malagasy humid forests, and present-day populations represent only relict populations. The altitudinal range of the species with 825–1300 m supports this. However, since *Tetramorium naganum* is not particularly abundant or common where it occurs, it may just be a relatively rare faunal element in eastern Madagascar. If so, further sampling might yield additional material in the future, especially in the geographic areas between the two main populations mentioned above. Like most species in this group, *Tetramorium naganum* seems to be a leaf litter inhabitant.

###### Discussion.

*Tetramorium naganum* is the only species of the group that was known prior to our revision, and can be seen as the core species of the group. The lack of pilosity on the first gastral tergite isolates it fairly well from *Tetramorium alperti* and *Tetramorium gilgamesh* since these possess pilosity in varying degrees of inclination, length, and abundance. The relatively thin and high petiolar node (LPeI 54–58) separates *Tetramorium naganum* from *Tetramorium alperti* and *Tetramorium enkidu*, which have much thicker petiolar nodes (LPeI 60–68). Additionally, *Tetramorium naganum* has smaller eyes (OI 21–23) than *Tetramorium gilgamesh* (OI 25–27). The last species of the group, *Tetramorium dalek*, shares the lack of long, standing pilosity on the first gastral tergite with *Tetramorium naganum*. Nevertheless, both species are very unlikely to be confused with each other. *Tetramorium dalek* is generally smaller (HW 0.45–0.54; WL 0.54–68), has shorter propodeal spines (PSLI 25–27), and lacks long, standing hairs on the waist segments, whereas *Tetramorium naganum* is generally larger (HW 0.55–0.72; WL 0.66–0.92), possesses longer propodeal spines (PSLI 28–33), and always has long, standing hairs on the waist segments.

### Revision of the *Tetramorium plesiarum* species group

#### *Tetramorium plesiarum* species group

**Diagnosis.** Eleven-segmented antennae; anterior clypeal margin medially impressed; frontal carinae strongly developed and forming dorsal margin of very well-developed antennal scrobes, scrobes moderately to very deep and with clearly defined margins all around (except in *Tetramorium gollum*); median scrobal carina very well developed and distinctly surpassing posterior eye level, usually ending between posterior eye margin and posterior scrobe margin, often approaching the latter; anterior face of mesosoma weakly developed; mesosomal outline in profile moderately to strongly convex, moderately marginate from lateral to dorsal mesosoma; mesosoma relatively high (LMI 41–56); propodeal spines usually medium-sized to long, elongate-triangular to spinose (PSLI 23–32), rarely short (PSLI 18–20); propodeal lobes usually short, triangular to elongate-triangular, and acute; petiolar node in profile nodiform to high nodiform, in profile between 1.3 to 1.8 times higher than long, (LPeI 56–76), anterior and posterior faces generally parallel, anterodorsal and posterodorsal margins situated at about same height; node in dorsal view typically distinctly wider than long, between 1.1 to 1.5 times (DPeI 108–148); postpetiole much broader than long and transverse, between 1.4 to 2 times broader than long (DPpI 141–200); sculpture on mandibles variably developed; cephalic sculpture distinct, between frontal carinae predominantly longitudinally rugose; mesosoma with well-developed longitudinally rugose sculpture; waist segments weakly to moderately sculptured, never completely unsculptured; first gastral tergite usually with weak ground sculpture at base or completely unsculptured, in *Tetramorium gollum* basal half of tergite strongly reticulate-rugose; whole body with abundant, usually dense, long, and standing hairs; sting appendage spatulate.

**Figure 10.**
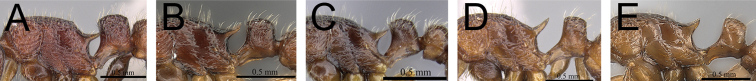
Mesosoma and petiole in profile. **A** *Tetramorium hobbit* (CASENT0019207) **B** *Tetramorium mars* (CASENT0474279) **C** *Tetramorium plesiarum* (CASENT0036474) **D** *Tetramorium gollum* (CASENT0074974) **E** *Tetramorium bressleri* (CASENT0055196).

**Comments.** The *Tetramorium plesiarum* group is a compact assemblage of five species that resemble one another very closely. All are morphologically very conspicuous elements within the Malagasy *Tetramorium* fauna, and represent arid-adapted species found mostly in the drier western and southern parts of Madagascar. Surprisingly, *Tetramorium plesiarum*, which is much less common than *Tetramorium bressleri*, *Tetramorium hobbit*, and *Tetramorium mars*, is the only member of the species group that was known prior to this revision, and only from the holotype. The other four species, *Tetramorium bressleri*, *Tetramorium gollum*, *Tetramorium hobbit*, and *Tetramorium mars*, are newly described here. All five species share a more or less similar habitus with very well developed antennal scrobes, more or less convex mesosomal profiles, usually medium-sized to long propodeal spines, higher than long and broader than long petiolar nodes, and abundant, long, and often dense pilosity. The species delimitations presented here are mainly based on differences in the shape of the petiolar node and sculpture on different parts of the body. One intriguing feature of the group is the morphological cline observable in the shape of the petiolar node (see Fig. 10), which ranges from massively enlarged and blocky (*Tetramorium hobbit*) through smaller, but still relatively blocky (*Tetramorium mars*), to much higher and thinner (*Tetramorium bressleri*, *Tetramorium gollum*, *Tetramorium plesiarum*).

*Tetramorium plesiarum* was initially placed in the *Tetramorium ranarum* species group by Bolton (1979) mainly on the basis of petiolar node shape and pilosity. However, in Hita Garcia and Fisher (2012a) we proposed a *Tetramorium plesiarum* species group based on the very conspicuous antennal scrobes present in all species of the group, a character absent in most other Malagasy *Tetramorium*. We still think that the *Tetramorium plesiarum* species group presents a well distinguishable grouping, although the conspicuous antennal scrobes unfortunately do not separate it from all other Malagasy groups. Several species within the *Tetramorium ranarum* group (e.g. *Tetramorium zenatum*, *Tetramorium ibycterum*, and two additional, yet-undescribed species) also have distinct antennal scrobes, although in these species the margins around the scrobes are much less distinctive and the scrobes themselves shallower than in the *Tetramorium plesiarum* group. However, the species of the *Tetramorium ranarum* group with a scrobe 1) either lack any long, suberect to erect pilosity on the first gastral tergite (*Tetramorium ibycterum* and another undescribed species here listed as *Tetramorium* fhg-bilb), or 2) they have long, subdecumbent pilosity (e.g. one yet-undescribed species listed here as *Tetramorium* fhg-vazi), or 3) if they possess long, standing pilosity, the petiolar node is much longer than broad (*Tetramorium zenatum*). Therefore they cannot be confused with any species of the *Tetramorium plesiarum* group.

##### Identification key to species of the *Tetramorium plesiarum* species group (workers)

**Table:** 

1	Gaster conspicuously enlarged, appearing swollen; basal half of first gastral tergite strongly sculptured (Fig. 11A) [Madagascar]	*Tetramorium gollum*
–	Gaster never enlarged or swollen; first gastral tergite not strongly sculptured; if sculpture present, then restricted to base of the tergite (Fig. 11B, C)	2
2	Petiolar node blocky, massive and very large; head, mesosoma, and waist segments with conspicuous reticulate-punctate ground sculpture (Fig. 12A) [Madagascar]	*Tetramorium hobbit*
–	Petiolar node always much smaller than above; head, mesosoma, and waist segments without conspicuous reticulate-punctate ground sculpture (Fig. 12B, C)	3
3	Antennal scapes shorter (SI 58–62); petiolar node in profile lower and thicker and in dorsal view longer (LPeI 69–76; DPeI 108–123); dorsum of petiolar node fully covered with distinctly reticulate-rugose sculpture (Fig. 13A) [Madagascar]	*Tetramorium mars*
–	Antennal scapes longer (SI 62–69); petiolar node in profile higher and thinner and in dorsal view broader (LPeI 56–63; DPeI 130–144); dorsum of petiolar node weakly rugulose/rugose, mostly smooth (Fig. 13B, C)	4
4	Generally smaller species (HW 0.67–0.76; WL 0.81–092); eyes relatively larger (OI 21–23); in profile anterodorsal margin of petiolar node clearly protruding anteriorly; mesopleuron and lateral propodeum longitudinally rugose with distinct reticulate-punctate ground sculpture, appearing matte and rough; sides of petiolar node with strong reticulate-punctate ground sculpture, appearing fairly matte and rough (Fig. 14A) [Madagascar	*Tetramorium plesiarum*
–	Generally larger species (HW 0.80–1.00; WL 0.92–1.15); eyes relatively smaller (OI 18–19); in profile anterodorsal margin of petiolar node not protruding anteriorly; mesopleuron and lateral propodeum with very little rugose/rugulose sculpture, mostly unsculptured, smooth, and shining; sides of petiolar node with weak but distinct reticulate-punctate ground sculpture, appearing only slightly matte and mostly smooth and shining (Fig. 14B) [Madagascar]	*Tetramorium bressleri*

**Figure 11.**
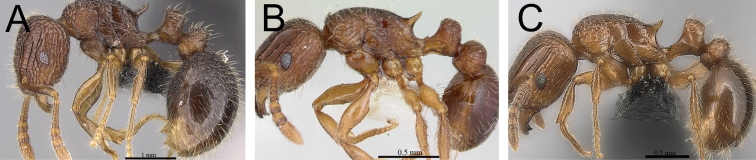
Body in profile. **A** *Tetramorium gollum* (CASENT0074849) **B** *Tetramorium plesiarum* (CASENT0172831) **C** *Tetramorium bressleri* (CASENT0055196).

**Figure 12.**
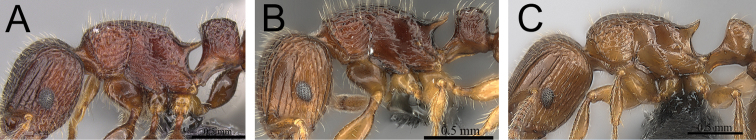
Head, mesosoma and petiole in profile. **A** *Tetramorium hobbit* (CASENT0019207) **B** *Tetramorium mars* (CASENT0474279) **C** *Tetramorium bressleri* (CASENT0055196).

**Figure 13.**
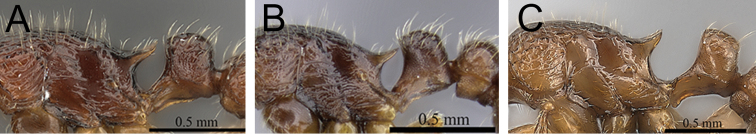
Mesosoma and petiole in profile. **A** *Tetramorium mars* (CASENT0474279) **B** *Tetramorium plesiarum* (CASENT0172831) **C** *Tetramorium bressleri* (CASENT0055196).

**Figure 14.**
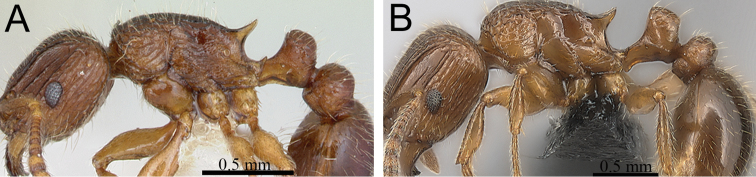
Body in profile. **A** *Tetramorium plesiarum* (CASENT0172831) **B** *Tetramorium bressleri* (CASENT0055196).

##### Tetramorium bressleri

Hita Garcia & Fisher sp. n.

http://zoobank.org/288AA21B-2B9E-49F1-8C6A-B22E6FD4619E

http://species-id.net/wiki/Tetramorium_bressleri

**Figure 15.**
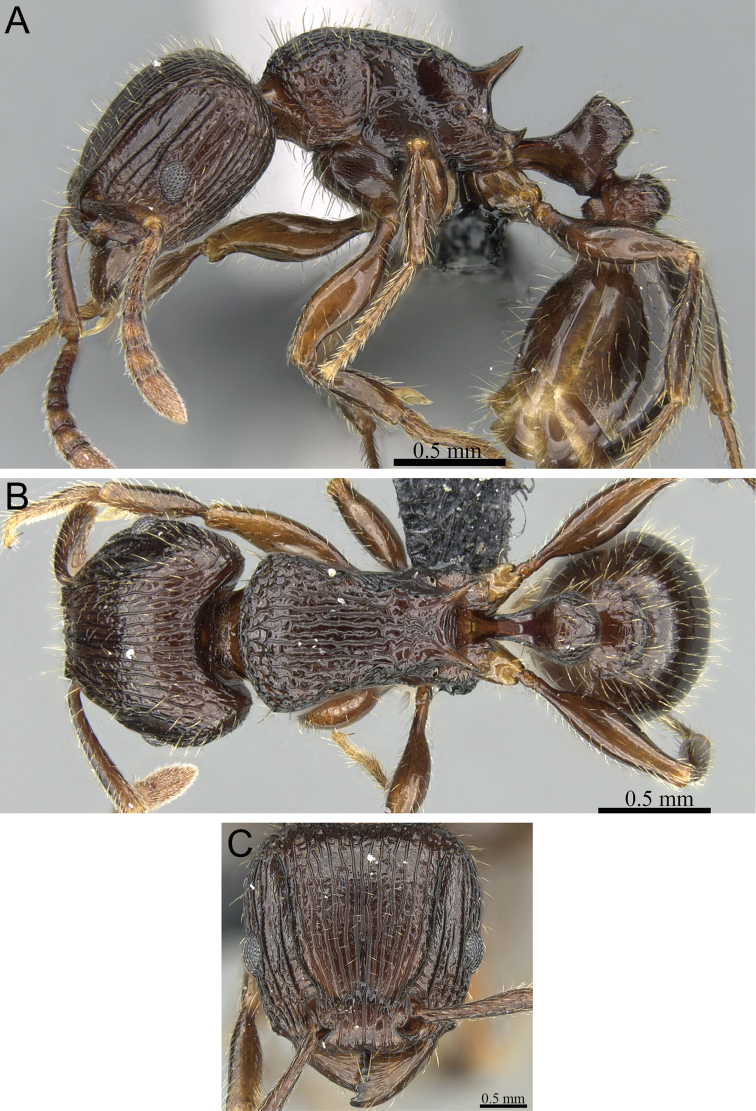
*Tetramorium bressleri* holotype worker (CASENT0035677). **A** Body in profile **B** Body in dorsal view **C** Head in full-face view.

Figs 10E , 11C , 12C , 13C , 14B , 15 , 62

###### Type material.

**Holotype**, pinned worker, MADAGASCAR, Mahajanga, Parc National de Namoroka, 9.8 km 300° WNW Vilanandro, 16.46667°S, 45.35°E, 140 m, tropical dry forest, sifted litter (leaf mold, rotten wood), collection code BLF06446, 4.–8.XI.2002 (*B.L. Fisher et al.*) (CAS: CASENT0035677). **Paratypes**, 27 pinned workers with same data as holotype (BMNH: CASENT0035669; CAS: CASENT0035636; CASENT0035640; CASENT0035642; CASENT0035647; CASENT0035648; CASENT0035649; CASENT0035652; CASENT0035653; CASENT0035654; CASENT0035656; CASENT0035658; CASENT0035660; CASENT0035662; CASENT0035666; CASENT0035667; CASENT0035670; CASENT0035674; CASENT0035675; CASENT0035678; CASENT0035681; CASENT0035686; CASENT0035687; CASENT0035689; CASENT0035694; CASENT0035703; MCZ: CASENT0035646).

###### Non-type material.

MADAGASCAR: Antananarivo, Reserve Speciale d’Ambohitantely, Forêt Ambohitantely, 20.9 km 72°NE Ankazobe, 18.2253°S, 47.2868°E, 1410 m, montane rainforest, 17.–22.IV.2001 (*B.L. Fisher et al.*); Antsiranana, Forêt d’Anabohazo, 21.6 km 247° WSW Maromandia, 14.3089°S, 47.9143°E, 120 m, tropical dry forest, 11.–16.III.2001 (*B.L. Fisher et al.*); Fianarantsoa, Ampangabe I Non Protected Area, 21.4 km W Itremo, 20.6111°S, 46.6069°E, 1414 m, savannah woodland, 21.–23.III.2010 (*A. Ravelomanana*); Fianarantsoa, Ampangabe II Non Protected Area, 21.29 km W Itremo, 20.6114°S, 46.6081°E, 1402 m, savannah woodland, 21.–23.III.2010 (*A. Ravelomanana*); Fianarantsoa, Ampangabe VI Non Protected Area, 21.16 km W Itremo, 20.6144°S, 46.6104°E, 1379 m, shrubland, 21.–23.III.2010 (*A. Ravelomanana*); Fianarantsoa, Ampangoabe V Non Protected Area, 21.37 km W Itremo, 20.6136°S, 46.608°E, 1449 m, shrubland, 22.–23.III.2010 (*A. Ravelomanana*); Fianarantsoa, Ampotoampoto I National Parc, 8.02 km NW Ilakaka, 22.6283°S, 45.1886°E, 917 m, savannah woodland, 26.–28.II.2010 (*A. Ravelomanana*); Fianarantsoa, Forêt d’Analalava, 29.6 km 280° W Ranohira, 22.5917°S, 45.1283°E, 700 m, 1.–5.II.2003 (*B.L. Fisher et al.*); Fianarantsoa, Antohatsahomby I Non Protected Area, 22.77 km NW Ambatofinandrahana, 20.5506°S, 46.5856°E, 1550 m, *Uapaca* woodland, 15.–17.III.2010 (*A. Ravelomanana*); Fianarantsoa, Forêt d’Atsirakambiaty, 7.6 km 285° WNW Itremo, 20.5933°S, 46.5633°E, 1550 m, montane rainforest, 22.–26.I.2003 (*B.L. Fisher et al.*); Fianarantsoa, Parc National d’Isalo, 9.1 km 354° N Ranohira, 22.4817°S, 45.4617°E, 725 m, gallery forest, 27.–31.I.2003 (*B.L. Fisher et al.*); Fianarantsoa, Parc National d’Isalo, Ambovo Springs, 29.3 km 4° N Ranohira, 22.2983°S, 45.3517°E, 990 m, *Uapaca* woodland, 9.–14.II.2003 (*B.L. Fisher et al.*); Fianarantsoa, Mampiarika I Non Protected Area, 28.08 km SW Ambositra, 20.7344°S, 47.0835°E, 1480 m, *Uapaca* woodland, 31.I.–2.II.2010 (*A. Ravelomanana*); Mahajanga, Forêt Ambohimanga, 26.1 km 314° Mampikony, 15.9627°S, 47.4382°E, 250 m, tropical dry forest, 13.–15.XII.2004 (*B.L. Fisher*); Mahajanga, Boeny Region, District of Soalala, Analamanitra forest, 14 km SW Mitsinjo, 16.7°S, 45.7°E, 19 m, dense dry forest, 26.II.-4.III.2008 (*M. Rin’ha*); Mahajanga, Parc National d’Ankarafantsika, Ampijoroa Station Forestiere, 40 km 306° NW Andranofasika, 16.3208°S, 46.8107°E, 130 m, tropical dry forest, 26.III.-1.IV.2001 (*B.L. Fisher et al.*); Mahajanga, Parc National d’Ankarafantsika, Ampijoroa Station Forestiere, 5.4 km 331° NW Andranofasika, 16.2989°S, 46.813°E, 70 m, tropical dry forest, 26.III.-1.IV.2001 (*B.L. Fisher et al.*); Mahajanga, Parc National d’Ankarafantsika, Forêt de Tsimaloto, 18.3 km 46° NE de Tsaramandroso, 16.2281°S, 47.1436°E, 135 m, tropical dry forest, 2.IV.-8.IV.2001 (*B.L. Fisher et al.*); Mahajanga, Parc National de Baie de Baly, 12.4 km 337° NNW Soalala, 16.01°S, 45.265°E, 10 m, tropical dry forest, 26.–30.XI.2002 (*B.L. Fisher et al.*); Mahajanga, Reserve Forestiere Beanka, 50.2 km E Maintirano, 18.0265°S, 44.0505°E, 250 m, tropical dry forest on tsingy, 19.–26.X.2009 (*B.L. Fisher et al.*); Mahajanga, Reserve Forestiere Beanka, 52.7 km E Maintirano, 18.0622°S, 44.5259°E, 300 m, tropical dry forest on tsingy, 24.–27.X.2009 (*B.L. Fisher et al.*); Mahajanga, Reserve Forestiere Beanka, 50.7 km E Maintirano, 17.8802°S, 44.4688°E, 140 m, tropical dry forest on tsingy, 29.X.–1.XI.2009 (*B.L. Fisher et al.*); Mahajanga, Reserve Forestiere Beanka, 50.2 km E Maintirano, 17.8876°S, 44.4726°E, 153 m, tropical dry forest on tsingy, 31.X.2009 (*B.L. Fisher et al.*); Mahajanga, Reserve Speciale de Bemarivo, 23.8 km 223° SW Besalampy, 16.925°S, 44.3683°E, 30 m, tropical dry forest, 19.–23.XI.2002 (*B.L. Fisher et al.*); Mahajanga, Mahavavy River, 6.2 km 145° SE Mitsinjo, 16.0517°S, 45.9083°E, 20 m, gallery forest, 1.–5.XII.2002 (*B.L. Fisher et al.*); Mahajanga, Parc National de Namoroka, 9.8 km 300° WNW Vilanandro, 16.4667°S, 45.35°E, 140 m, tropical dry forest, 4.–8.XI.2002 (*B.L. Fisher et al.*); Mahajanga, Parc National de Namoroka, 17.8 km 329° WNW Vilanandro, 16.3767°S, 45.3267°E, 100 m, tropical dry forest, 8.–12.XI.2002 (*B.L. Fisher et al.*); Mahajanga, Parc National de Namoroka, 16.9 km 317° NW Vilanandro, 16.4067°S, 45.31°E, 100 m, tropical dry forest, 12.–16.XI.2002 (*B.L. Fisher et al.*); Mahajanga, Parc National Tsingy de Bemaraha, 3.4 km 93° E Bekopaka, Tombeau Vazimba, 19.1419°S, 44.828°E, 50 m, tropical dry forest, 6.–10.XI.2001 (*B.L. Fisher et al.*); Mahajanga, Parc National Tsingy de Bemaraha, 2.5 km 62° ENE Bekopaka, Ankidrodroa River, 19.1322°S, 44.8147°E, 100 m, 11.–15.XI.2001 (*B.L. Fisher et al.*); Mahajanga, Parc National Tsingy de Bemaraha, 10.6 km ESE 123° Antsalova, 18.7094°S, 44.7182°E, 150 m, tropical dry forest on tsingy, 16.–20.XI.2001 (*B.L. Fisher et al.*); Toliara, Parc National d’Andohahela, Forêt d’Ambohibory, 1.7 km 61° ENE Tsimelahy, 36.1 km 308° NW Tolagnaro, 24.93°S, 46.6455°E, 300 m, tropical dry forest, 16.–20.I.2002 (*B.L. Fisher et al.*); Toliara, Andohahela National Park, Tsimelahy, 24.9368°S, 46.6267°E, 180 m, transition forest, 16.–26.II.2003 (*M.E. Irwin et al.*); Toliara, 18 km NNW Betroka, 23.1633°S, 45.9686°E, 825 m, savannah, 24.XI.-4.XII.1994 (*M.A. Ivie & D. A. Pollock*); Toliara, Atsimo Andrefana Region, District of Betioky, 30 km E Betioky, Beza Mahafaly Special Reserve, 23.6865°S, 44.591°E, 165 m, gallery dry deciduous forest, 1.–7.I.2002 (*M. Rin’ha*); Toliara, Beza-Mahafaly, 27 km E Betioky, 23.65°S, 44.6333°E, 135 m, tropical dry forest, 23.IV.1997 (*B.L. Fisher*); Toliara, Fiherenana, 23.1769°S, 43.9608°E, 100 m, gallery forest, 21.–24.X.2002 (*Frontier Project*); Toliara, Fiherenana, 23.177°S, 43.9614°E, gallery forest, 18.–19.VII.2003 (*Frontier Wilderness Project*); Toliara, southern Isoky-Vohimena Forest, 59 km NE Sakaraha, 22.4667°S, 44.85°E, 730 m, tropical dry forest, 21.I.1996 (*B.L. Fisher*); Toliara, Makay Mts., 21.2098°S, 45.3418°E, 525 m, gallery forest on sandy soil, 27.XI.-2.XII.2010 (*B.L. Fisher et al.*); Toliara, Makay Mts., 21.2199°S, 45.324°E, 500 m, gallery forest on sandy soil, 24.XI.-12.XII.2010 (*B.L. Fisher et al.*); Toliara, Makay Mts., 21.31°S, 45.1295°E, 590 m, dry forest on sandy soil, 3.–6.XII.2010 (*B.L. Fisher et al.*); Toliara, Vohibasia Forest, 59 km NE Sakaraha, 22.4667°S, 44.85°E, 780 m, tropical dry forest, 13.I.1996 (*B.L. Fisher*); Toliara, Parc National de Zombitse, near road, 22.8405°S, 44.7312°E, 825 m, spiny deciduous forest, 15.X.2001-23.III.2002 (*R. Harin’Hala*); Toliara, Parc National de Zombitse, near ANGAP office, 22.8865°S, 44.6922°E, 840 m, deciduous spiny forest, 9.XI.2001-26.I.2002 (*R. Harin’Hala*); Toliara, Parc National de Zombitse, 19.8 km 84° E Sakaraha, 22.8433°S, 44.71°E, 770 m, tropical dry forest, 5.–9.II.2003 (*B.L. Fisher et al.*).

###### Diagnosis.

*Tetramorium bressleri* can be recognised by the following combination of characters: larger species (HW 0.80–1.00; WL 0.92–1.15); eyes relatively small (OI 18–19); petiolar node high nodiform, not blocky and massively enlarged, anterodorsal and posterodorsal margins at about the same height and equally marginate, anterodorsal margin not protruding anteriorly nor very sharply angled; petiolar node in profile relatively high and thin, between 1.6 to 1.8 times higher than long (LPeI 56–61), in dorsal view between 1.3 to 1.5 times wider than long (DPeI 135–145); gaster never extremely enlarged and swollen; head and mesosoma without strongly developed and conspicuous reticulate-punctate ground sculpture; usually sculpture on the mesopleuron and lateral propodeum mostly absent; basal half of first gastral tergite not strongly reticulate-rugose, only base of tergite weakly sculptured; pilosity on first gastral tergite mostly erect.

###### Worker measurements

**(N=12).** HL 0.81–1.00 (0.94); HW 0.80–1.00 (0.94); SL 0.51–0.64 (0.60); EL 0.15–0.19 (0.18); PH 0.42–0.53 (0.48); PW 0.57–0.73 (0.68); WL 0.92–1.15 (1.07); PSL 0.24–0.32 (0.27); PTL 0.20–0.26 (0.23); PTH 0.35–0.42 (0.40); PTW 0.28–0.36 (0.32); PPL 0.23–0.30 (0.27); PPH 0.32–0.41 (0.38); PPW 0.35–0.45 (0.42); CI 98–102 (100); SI 62–65 (64); OI 18–19 (19); DMI 61–66 (64); LMI 43–47 (45); PSLI 26–32 (28); PeNI 45–49 (47); LPeI 56–61 (58); DPeI 135–145 (138); PpNI 59–63 (61); LPpI 70–75 (73); DPpI 150–158 (152); PPI 125–134 (130).

###### Worker description.

Head more or less as long as broad (CI 98–102); posterior head margin weakly concave. Anterior clypeal margin with distinct median impression. Frontal carinae strongly developed and forming dorsal margin of very well-developed antennal scrobes, scrobes moderately to very deep and with clearly defined margins all around; median scrobal carina very well developed and distinctly surpassing posterior eye level, usually ending halfway between posterior eye margin and posterior scrobe margin. Antennal scapes short, not reaching posterior head margin (SI 62–65). Eyes relatively small (OI 18–19). Mesosomal outline in profile weakly to moderately convex, rounded and high (LMI 43–47), moderately to strongly marginate from lateral to dorsal mesosoma; promesonotal suture and metanotal groove absent. Propodeal spines elongate-triangular to spinose, long, and acute (PSLI 26–32), propodeal lobes short, triangular, and acute, always much shorter than propodeal spines. Petiolar node in profile high, rectangular nodiform with well defined antero- and posterodorsal margins, between 1.6 to 1.8 times higher than long (LPeI 56–61), anterior and posterior faces approximately parallel, anterodorsal and posterodorsal margins situated at about the same height, petiolar dorsum flat to weakly convex, anterodorsal margin not protruding anteriorly; node in dorsal view between 1.3 to 1.5 times wider than long (DPeI 135–145), in dorsal view pronotum between 2.0 to 2.2 times wider than petiolar node (PeNI 45–49). Postpetiole in profile subglobular and weakly anteroposteriorly compressed, approximately 1.3 to 1.4 times higher than long (LPpI 70–75); in dorsal view around 1.5 to 1.6 times wider than long (DPpI 150–158), pronotum between 1.6 to 1.7 times wider than postpetiole (PpNI 59–63). Postpetiole in profile appearing slightly less voluminous than petiolar node, postpetiole in dorsal view between 1.2 to 1.4 times wider than petiolar node (PPI 125–134). Mandibles variably sculptured, either unsculptured, smooth, and shining, or partly or fully finely rugulose; clypeus longitudinally rugose/rugulose, with five to eight distinct rugae, median ruga always distinct, lateral rugae often weaker and sometimes interrupted; cephalic dorsum between frontal carinae with ten to thirteen longitudinal rugae, rugae running from posterior clypeal margin to posterior head margin, rarely interrupted or with cross-meshes; scrobal area mostly unsculptured, smooth and shiny; lateral head mainly longitudinally rugose. Ground sculpture on head usually weak to absent. Dorsum of mesosoma mostly longitudinally rugose without any distinct ground sculpture, spaces between rugae smooth and shining; lateral pronotum irregularly longitudinally rugose, often with weak to moderate punctate ground sculpture, mesopleuron and lateral propodeum usually with very little sculpture, few irregular rugae/rugulae and no ground sculpture. Forecoxae often with weak ground sculpture, but generally very smooth and shining. Petiolar node laterally with conspicuous but relatively weak reticulate-punctate ground sculpture only, appearing weakly matte but still relatively smooth and shiny; dorsum of node medially almost unsculptured, smooth, and shiny, surrounding areas rugulose, ground sculpture on petiolar dorsum neglectable. Postpetiole laterally and dorsally weakly to moderately rugose/rugulose and with conspicuous, moderate reticulate-punctate ground sculpture, appearing relatively matte; base of first gastral tergite usually weakly punctate and/or costulate and/or shagreened, remainder of tergite unsculptured, smooth, and shining. Whole body with abundant, long, and fine standing hairs; first gastral tergite with abundant, long, erect hairs and much scarcer, shorter, decumbent to subdecumbent hairs. Anterior edges of antennal scapes and dorsal (outer) surfaces of hind tibiae with decumbent to suberect hairs. Body usually of uniform brown, appendages often lighter.

###### Etymology.

The name of the new species is a patronym dedicated to Dr. Barry Lee Bressler, retired physicist, former adjunct professor of physics at Virginia Polytechnic Institute and State University, and amateur naturalist, in recognition of his interest in myrmecology and his support of ant taxonomy.

###### Distribution and biology.

As mentioned above, it is surprising that most species in the species group, except the holotype of *Tetramorium plesiarum*, were previously unknown. This is especially true for *Tetramorium bressleri*. It is by far the most common and abundant species of the *Tetramorium plesiarum* group. The material available was sampled in many localities and includes more than 500 mounted specimens with many more in alcohol. The distribution ranges of all group members strongly overlap, but the range of *Tetramorium bressleri* is by far the largest; this species is known from many more localities than the other four (Fig. 62). The southernmost localities are Andohahela, Beza Mahafaly, and Fiherenana, and from there the distribution ranges north through much of western Madagascar up to the northernmost known locality Anabohazo. The eastern limit of the range goes in an almost straight line north from Andohahela through Mampiarika, Ambohitantely, and Ambohimanga to Anabohazo. The new species prefers arid habitats such as tropical dry forests, tropical dry forests on tsingy, gallery forests, spiny deciduous forests, savannah woodland, *Uapaca* woodland, and spiny thickets. The elevational range of the species is a relatively broad one, ranging from 10 to 1550 m, but most of the material was collected at low elevations (ca. 420 m on average). *Tetramorium bressleri* was mainly sampled by pitfall trapping and litter sifting, suggesting a ground-active life style.

###### Discussion.

*Tetramorium bressleri* is not likely to be confused with *Tetramorium gollum*, *Tetramorium hobbit*, or *Tetramorium mars*, whereas differentiating between *Tetramorium bressleri* and *Tetramorium plesiarum* can be challenging at first glance. *Tetramorium bressleri* lacks the enlarged gaster and strong reticulate-rugose sculpture on the basal half of the first gastral tergite seen in *Tetramorium gollum*, nor does it have the blocky petiolar node shape of *Tetramorium mars* or the massively enlarged petiolar node of *Tetramorium hobbit*. The separation from *Tetramorium plesiarum* requires more attention and caution. The main and obvious difference is body size. *Tetramorium bressleri* is usually a much larger species (HW 0.80–1.00; WL 0.92–1.15) than *Tetramorium plesiarum* (HW 0.80–1.00; WL 0.92–1.15). There is some overlap, but this is mainly due to a few very small specimens of *Tetramorium bressleri* and one very large specimen of *Tetramorium plesiarum*. Otherwise, both species fall neatly into their respective size ranges. However, body size alone should not be used as a primary diagnostic character, and in this case, we provide more evidence for their heterospecificity. The eyes of *Tetramorium plesiarum* (OI 21–23) are larger than in *Tetramorium bressleri* (OI 18–19), although this is often difficult to assess without measuring. In addition, both can be separated by the sculpture on the mesopleuron and lateral propodeum; this sculpture is usually mostly absent in *Tetramorium bressleri*, making the area appear very smooth and shiny, while it is usually longitudinally rugose with reticulate-punctate ground sculpture in *Tetramorium plesiarum*. The sculpture on the sides of the petiolar node varies too, since it is much more reticulate-punctate and matte in *Tetramorium plesiarum* than in *Tetramorium bressleri*, in which the weak reticulate-punctate ground sculpture looks faintly matte but still relatively smooth and shiny. The shape of the petiolar node in profile is an additional useful character. In *Tetramorium plesiarum* the anterodorsal margin of the node protrudes anteriorly and is a bit more marginate than the more rounded posterodorsal margin, while in *Tetramorium bressleri* both margins are usually equally marginate and the anterodorsal margin does not protrude anteriorly at all.

Considering how common and abundant *Tetramorium bressleri* is in western Madagascar, it is interesting that it shows very little intraspecific variation and remains very stable in its morphology.

##### Tetramorium gollum

Hita Garcia & Fisher sp. n.

http://zoobank.org/60DC6DB7-5425-4597-B2E3-3D00E8AD4E2B

http://species-id.net/wiki/Tetramorium_gollum

**Figure 16.**
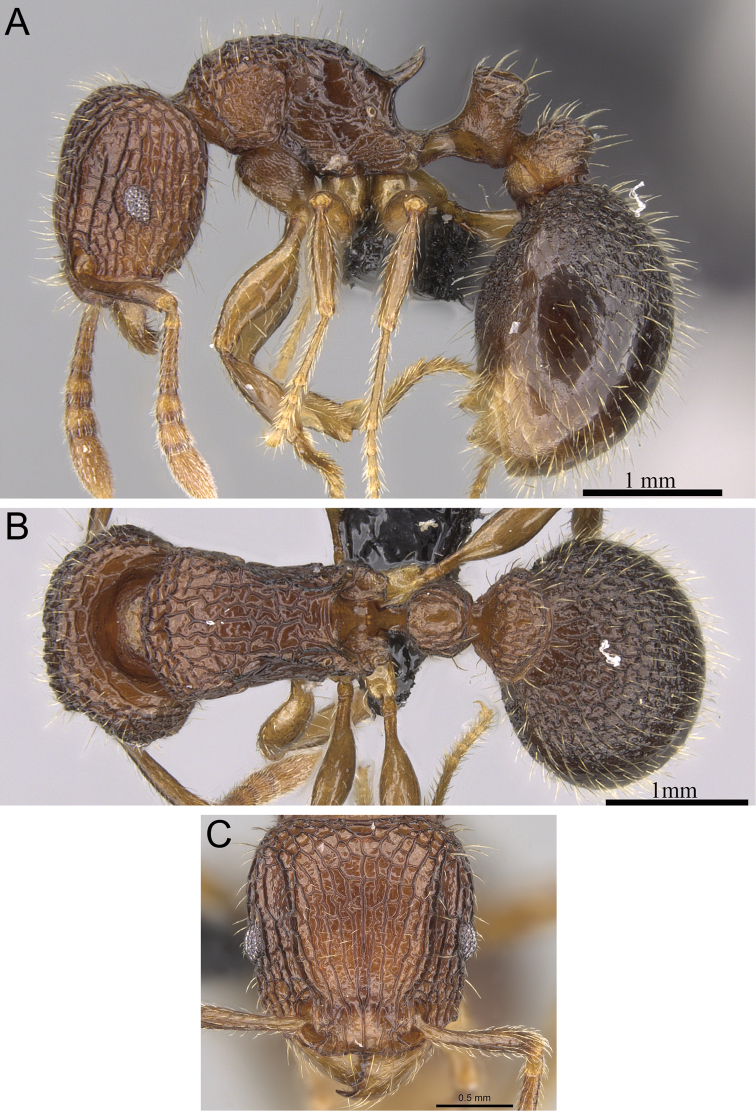
*Tetramorium gollum* holotype worker (CASENT0074849). **A** Body in profile **B** Body in dorsal view **C** Head in full-face view.

Figs 10D , 11A , 16 , 62

###### Type material.

**Holotype**, pinned worker, MADAGASCAR, Fianarantsoa, Forêt d’Analalava, 29.6 km 280° W, Ranohira, 22.59167°S, 45.12833°E, 700 m, tropical dry forest, pitfall trap, collection code BLF07381, 1.–5.II.2003 (CAS: CASENT0074849). **Paratypes**, two pinned workers with same data as holotype (CAS: CASENT0074839, CASENT0074974).

###### Diagnosis.

The strongly developed sculpture on the basal half of the first gastral tergite and the extremely swollen gaster clearly distinguish *Tetramorium gollum* from the other species in the group.

###### Worker measurements

**(N=3).** HL 0.76–0.90 (0.81); HW 0.74–0.90 (0.80); SL 0.47–0.59 (0.52); EL 0.15–0.18 (0.16); PH 0.48–0.52 (0.50); PW 0.54–0.66 (0.59); WL 0.85–1.03 (0.93); PSL 0.23–0.26 (0.25); PTL 0.20–0.25 (0.23); PTH 0.35–0.42 (0.38); PTW 0.27–0.37 (0.31); PPL 0.24–0.27 (0.25); PPH 0.37–0.43 (0.40); PPW 0.40–0.50 (0.45); CI 97–100 (98); SI 64–66 (65); OI 19–20 (20); DMI 62–64 (63); LMI 50–56 (54); PSLI 29–31 (30); PeNI 50–56 (53); LPeI 57–61 (59); DPeI 130–148 (138); PpNI 75–79 (76); LPpI 63–65 (64); DPpI 167–185 (176); PPI 135–148 (143).

###### Worker description.

Head weakly longer than wide to as long as wide (CI 97–100); posterior head margin weakly concave. Anterior clypeal margin with distinct median impression. Frontal carinae strongly developed, curving down shortly before posterior head margin, forming dorsal margin of very well-developed antennal scrobes; scrobes moderately shallow; posterior and ventral margins not fully defined, merging with very strong cephalic sculpture. Antennal scapes short, not reaching posterior head margin (SI 64–66). Eyes relatively small (OI 19–20). Mesosomal outline in profile conspicuously convex, rounded, and very high (LMI 50–56), moderately to strongly marginate from lateral to dorsal mesosoma; promesonotal suture and metanotal groove absent. Propodeal spines long (PSLI 29–31), elongate-triangular to spinose, and acute; propodeal lobes short, triangular to elongate-triangular, and acute. Petiolar node in profile high, rectangular nodiform with well-defined antero- and posterodorsal margins, between 1.6 to 1.8 times higher than long (LPeI 57–61), anterior and posterior faces approximately parallel, anterodorsal and posterodorsal margins situated at about the same height, petiolar dorsum flat to weakly convex; node in dorsal view around 1.3 to 1.5 times wider than long (DPeI 130–148), in dorsal view pronotum between 1.8 to 2.0 times wider than petiolar node (PeNI 50–56). Postpetiole in profile anteroposteriorly compressed, approximately 1.5 to 1.6 times higher than long (LPpI 63–65); in dorsal view around 1.7 to 1.9 times wider than long (DPpI 167–185), pronotum only around 1.3 times wider than postpetiole (PpNI 75–79). Postpetiole in profile appearing approximately as voluminous as petiolar node but relatively thinner, postpetiole in dorsal view approximately 1.3 to 1.5 times wider than petiolar node (PPI 135–148). Gaster extremely enlarged and swollen. Mandibles unsculptured, smooth, and shining; clypeus longitudinally rugose, with five to six distinct rugae, median ruga better developed than remainder, rugae without cross-meshes; sculpture on cephalic dorsum between frontal carinae variable: either mainly longitudinally rugose with higher proportion of reticulate-rugose areas towards posterior head margin, or irregularly reticulate-rugose with a higher proportion of longitudinally rugose sculpture restricted anteriorly close to posterior clypeal margin; scrobal area partly unsculptured; lateral head longitudinally rugose to reticulate-rugose. Mesosoma laterally reticulate-rugose to irregularly longitudinally rugose with a few shining areas on mesopleuron and propodeum, dorsally reticulate-rugose to irregularly longitudinally rugose. Forecoxae unsculptured, smooth, and shining. Petiolar node laterally with weak, longitudinally rugose sculpture mostly restricted towards dorsum, dorsum of node mostly unsculptured, smooth, and shiny; sculpture on postpetiole better developed, laterally and dorsally reticulate-rugose. Basal half of first gastral tergite and most of first sternite with very conspicuous and strongly developed reticulate-rugose sculpture superimposed on a reticulate-punctate ground sculpture; remainder of gaster unsculptured, smooth, and shining. Ground sculpture on head, mesosoma, and waist segments weak to moderate, except procoxae with weak but distinct rugulose ground sculpture. Whole body with abundant, long, and fine standing hairs; first gastral tergite with a mix of more abundant, long, erect hairs and much scarcer, shorter, decumbent to subdecumbent hairs. Anterior edges of antennal scapes and dorsal (outer) surfaces of hind tibiae with decumbent to suberect hairs. Head, mesosoma, and waist segments light to dark brown, mandibles, antennae, and legs light brown to yellow, and gaster much darker brown than remainder of body.

###### Etymology.

The new species is named after the fictional character “Gollum” from J.R.R. Tolkien’s novels “The Hobbit” and “The Lord of the Rings”. The species epithet is an arbitrary combination of letters, thus invariable.

###### Distribution and ecology.

The new species is only known from the type locality, the Forêt d’Analalava, which is a tropical dry forest located at an elevation of 700 m (Fig. 62). The biology of the species is unknown, but foraging might be undertaken on the ground since the only three known specimens were collected from a pitfall trap. Indicated by this lack of material, *Tetramorium gollum* probably is one of the more rarely encountered *Tetramorium* species in Madagascar.

###### Discussion.

*Tetramorium gollum* is a very conspicuous species within the *Tetramorium plesiarum* group and the whole Malagasy *Tetramorium* fauna. The extremely swollen gaster with strongly developed sculpture on the basal half of the tergite is easily recognisable within the species group. The only other Malagasy species with such conspicuous sculpture clearly extending to half of the tergite or more are *Tetramorium jedi* in the *Tetramorium tortuosum* group and *Tetramorium sericeiventre* in the *Tetramorium sericeiventre* group. The latter has 12 antennal segments (vs. 11 in *Tetramorium gollum*) and *Tetramorium jedi* has, among other characters, a very differently shaped petiolar node (longer than broad in *Tetramorium jedi* vs. broader than long in *Tetramorium gollum*). Beyond sculpture and the enlarged gaster, *Tetramorium gollum* is morphologically very similar to *Tetramorium bressleri*, for example the petiolar node shape is identical in both suggesting a close relationship. However, they both also differ in the shape of the postpetiole, which is much higher and broader in *Tetramorium gollum* (LPpI 63–65; DPpI 167–185) than in *Tetramorium bressleri* (LPpI 59–63; DPpI 150–158).

##### Tetramorium hobbit

Hita Garcia & Fisher sp. n.

http://zoobank.org/125B2BB7-7E47-4273-B0FA-B2676FD18D8E

http://species-id.net/wiki/Tetramorium_hobbit

**Figure 17.**
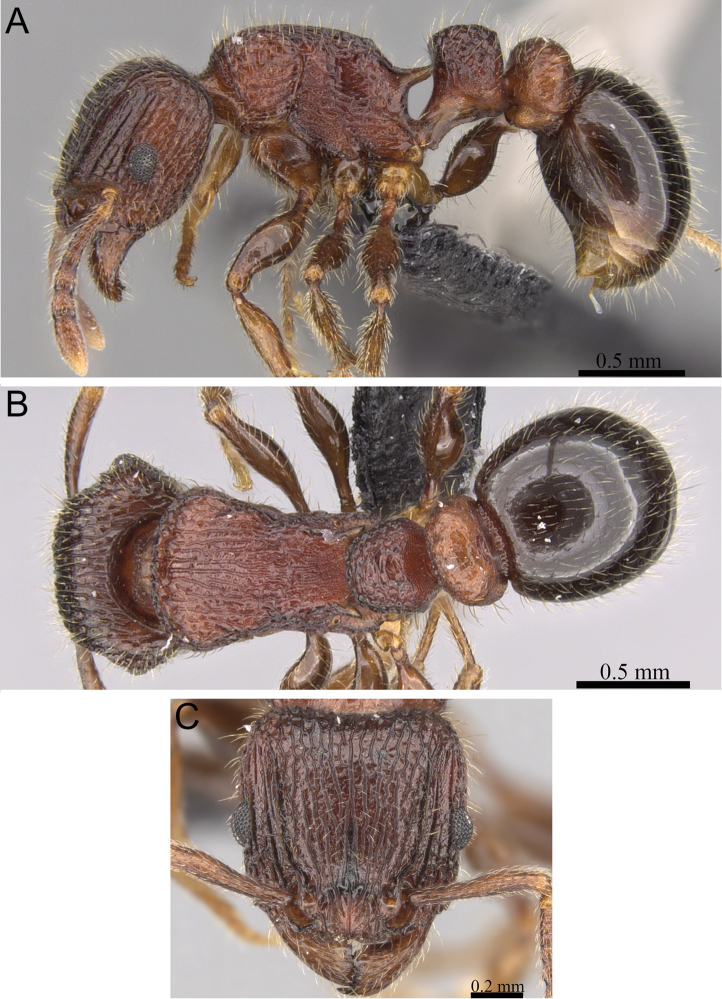
*Tetramorium hobbit* holotype worker (CASENT0019207). **A** Body in profile **B** Body in dorsal view **C** Head in full-face view.

Figs 10A , 12A , 17 , 62

###### Type material.

**Holotype**, pinned worker, MADAGASCAR, Toliara, Parc National de Tsimanampetsotsa, Forêt de Bemanateza, 20.7 km 81° E Efoetse, 23.0 km 131° SE Beheloka, 23.99222°S, 43.88067°E, 90 m, spiny forest/thicket, sifted litter (leaf mold, rotten wood), collection code BLF06254, 22.–26.III.2002 (*B.L. Fisher et al.*) (CAS: CASENT0019207). **Paratypes**, 2 pinned workers with same data as holotype (CAS: CASENT0019210; CASENT0019227); 2 pinned workers with same data as holotype except collected from pitfall trap and collection code BLF06258 (CAS: CASENT0078055; MHNG: CASENT0078059); 5 pinned workers with same data as holotype except collected from ground nest and collection code BLF06270 (CAS: CASENT0445004; CASENT0445005); and 2 pinned workers with same data as holotype except sampled from ground and collection code BLF06337 (MCZ: CASENT0445502; CASENT0445520).

###### Non-type material.

MADAGASCAR: Fianarantsoa, Ampandravelo II Non Protected Area, 10.78 km NE Ranohira, 22.5392°S, 45.5155°E, 873 m, shrubland, 20.–22.II.2010 (*A. Ravelomanana*); Fianarantsoa, Ampotoampoto I National Parc, 8.02 km NW Ilakaka, 22.6283°S, 45.1886°E, 917 m, savannah woodland, 26.–28.II.2010 (*A. Ravelomanana*); Fianarantsoa, Ampotoampoto III National Parc, 7.91 km NW Ilakaka, 22.6294°S, 45.189°E, 919 m, savannah woodland, 27.–28.II.2010 (*A. Ravelomanana*); Fianarantsoa, Ampotoampoto IV National Parc, 7.83 km NW Ilakaka, 22.6294°S, 45.1912°E, 923 m, savannah woodland, 27.–28.II.2010 (*A. Ravelomanana*); Toliara, Parc National d’Andohahela, Forêt de Manantalinjo, 33.6 km 63° ENE Amboasary, 7.6 km 99° E Hazofotsy, 24.8169°S, 46.61°E, 150 m, spiny forest/thicket, 12.–16.I.2002 (*B.L. Fisher et al.*); Toliara, Forêt de Beroboka, 5.9 km 131° SE Ankidranoka, 22.2331°S, 43.3663°E, 80 m, tropical dry forest, 12.–16.III.2002 (*B.L. Fisher et al.*); Toliara, Parc National de Kirindy Mite, 16.3 km 127° SE Belo sur Mer, 20.7953°S, 44.147°E, 80 m, tropical dry forest, 6.–10.III.2002 (*B.L. Fisher et al.*); Toliara, Makay Mts., 21.3411°S, 45.1805°E, 500 m, barren rock with sparse vegetation, burned grass, 28.XI.2010 (*B.L. Fisher et al.*); Toliara, Parc National de Tsimanampetsotsa, Forêt de Bemanateza, 20.7 km 81° E Efoetse, 23.0 km 131° SE Beheloka, 23.99222°S, 43.88067°E, 90 m, spiny forest/thicket, 22.–26.III.2002 (*B.L. Fisher et al.*).

###### Diagnosis.

*Tetramorium hobbit* is easily recognisable within the *Tetramorium plesiarum* species group due to its massively developed petiolar node and very conspicuous reticulate-punctate ground sculpture on head and mesosoma.

###### Worker measurements

**(N=12).** HL 0.79–0.88 (0.84); HW 0.80–0.88 (0.85); SL 0.52–0.58 (0.55); EL 0.16–0.20 (0.18); PH 0.42–0.53 (0.47); PW 0.63–0.72 (0.69); WL 0.96–1.11 (1.04); PSL 0.16–0.25 (0.20); PTL 0.28–0.31 (0.30); PTH 0.40–0.46 (0.44); PTW 0.38–0.45 (0.41); PPL 0.24–0.30 (0.27); PPH 0.39–0.45 (0.43); PPW 0.45–0.54 (0.51); CI 100–102 (101); SI 64–67 (65); OI 19–22 (21); DMI 63–72 (66); LMI 43–50 (46); PSLI 18–29 (23); PeNI 57–66 (60); LPeI 63–71 (68); DPeI 133–148 (140); PpNI 69–76 (74); LPpI 60–67 (63); DPpI 175–200 (188); PPI 116–126 (122).

###### Worker description.

Head as long as wide to weakly longer than wide (CI 100–102); posterior head margin weakly concave. Anterior clypeal margin with distinct median impression. Frontal carinae strongly developed and forming dorsal margin of very well-developed antennal scrobes, scrobes moderately to very deep and with clearly defined margins all around; median scrobal carina very well developed and distinctly surpassing posterior eye level, usually approaching posterior scrobe margin. Antennal scapes short, not reaching posterior head margin (SI 64–67). Eyes relatively small to moderate (OI 19–22). Mesosomal outline in profile conspicuously convex, rounded and often very high (LMI 43–50), moderately marginate from lateral to dorsal mesosoma; promesonotal suture and metanotal groove absent. Propodeal spines either short and elongate-triangular or long and spinose (PSLI 18–29; material from Andohahela and Tsimanampetsotsa with PSLI of 28–29; material from other localities PSLI 18–20), spines always with broad base and acute tip; propodeal lobes well developed, elongate-triangular, and acute, always shorter than propodeal spines. Petiolar node massively developed, very large, in profile high, rectangular, blocky nodiform with well defined antero- and posterodorsal margins, between 1.4 to 1.6 times higher than long (LPeI 63–71), anterior and posterior faces approximately parallel, anterodorsal and posterodorsal margins situated at about the same height, petiolar dorsum flat to weakly convex; node in dorsal view around 1.3 to 1.5 times wider than long (DPeI 133–148), in dorsal view pronotum around 1.5 to 1.7 times wider than petiolar node (PeNI 57–66). Postpetiole in profile subglobular to anteroposteriorly compressed, approximately 1.5 to 1.7 times higher than long (LPpI 60–67); in dorsal view around 1.7 to 1.9 times wider than long (DPpI 175–200), pronotum around 1.3 to 1.4 times wider than postpetiole (PpNI 69–76). Petiole in profile much more voluminous than postpetiole, in dorsal view postpetiole only about 1.1 to 1.3 times wider than petiolar node (PPI 116–126). Mandibles usually finely longitudinally rugulose, fairly smooth and shiny, sometimes unsculptured; clypeus longitudinally rugose, usually with five to seven very distinct and strong rugae, median ruga better developed than remainder, rugae usually without cross-meshes; cephalic dorsum between frontal carinae with 11 to 13 longitudinal rugae, rugae running from posterior clypeal margin to posterior head margin, often interrupted or with cross-meshes, especially posteriorly; scrobal area mostly unsculptured, only with ground sculpture; lateral head mainly reticulate-rugose. Mesosoma laterally reticulate-rugose to irregularly longitudinally rugose, dorsally mostly longitudinally rugose. Forecoxae unsculptured, smooth, and shining, often with weak traces of ground sculpture. In profile, basal half of petiolar node mostly unsculptured, upper half distinctly reticulate-rugose, dorsum of node distinctly reticulate-rugose; sculpture on postpetiole much weaker, laterally and dorsally only weakly rugulose. Gaster usually unsculptured, smooth and shining, sometimes with weak punctate ground sculpture at base of tergite. Ground sculpture on head, mesosoma, and petiolar node very conspicuous, usually strongly reticulate-punctate; ground sculpture on postpetiole and gaster usually much weaker but present, though sometimes absent. Whole body with very abundant, long, and fine standing hairs; first gastral tergite with abundant, long, erect hairs and much scarcer, shorter, decumbent to subdecumbent hairs. Anterior edges of antennal scapes and dorsal (outer) surfaces of hind tibiae with decumbent to suberect hairs. Body reddish to dark brown, head and gaster usually darker than rest of body.

###### Etymology.

This very hairy new species is named after the fictional people from J.R.R. Tolkien’s novels “The Hobbit” and “The Lord of the Rings”. The species epithet is an arbitrary combination of letters, thus invariant.

###### Distribution and biology.

The distribution of *Tetramorium hobbit* is approximately restricted to the southern third of the island of Madagascar (Fig. 62). In the south, the distribution range encompasses the type locality Tsimanampetsotsa in the west and Andohahela in the east, while the Makay Mountains and Isalo mark the northernmost known localities. Interestingly, the population in Andohahela seems to be slightly isolated from the other, more western localities. However, we consider this to be more of a sampling artifact. Like the remainder of the species group, *Tetramorium hobbit* prefers arid habitats, such as tropical dry forests, spiny forests and thickets, savannah woodland, and barren rocks with little vegetation. It is found at elevations of 80 to 923 m. *Tetramorium hobbit* is likely a ground-active species since it was mainly collected from leaf litter extractions and pitfall traps.

###### Discussion.

*Tetramorium hobbit* is very unlikely misidentified with the other four species of the group. The massively enlarged petiolar node, in combination with the distinct reticulate-punctate ground sculpture on head and mesosoma, are not seen in any other group member. However, of all group members, *Tetramorium mars* seems morphologically closest to *Tetramorium hobbit*, especially on the basis of the blockier petiolar node shape seen in profile that both species share. However, in addition to the smaller petiolar node and the lack of reticulate-punctate ground sculpture on head and mesosoma, *Tetramorium mars* also has shorter antennal scapes (SI 58–62; DPeI 108–122) and a narrower petiolar node in dorsal view than *Tetramorium hobbit* (SI 64–67; DPeI 133–148). *Tetramorium mars* (HW 0.69–0.74; WL 0.83–0.96) is also smaller than *Tetramorium hobbit* (HW 0.80–0.88; WL 0.96–1.10)

One noticeable intraspecific variation can be observed within *Tetramorium hobbit*. There are two distinct groups with differently developed propodeal spines. The populations from the type localities Tsimanampetsotsa and Andohahela always have relatively long spines (PSLI of 28–29), which contrast with the much shorter spines seen in the material from in Beroboka, Kirindy, Isalo, and the Makay Mountains (PSLI 18 -20). This difference is very pronounced and constant in all of the examined material. However, since there is no other obvious morphological difference between the short-spined and the long-spined populations, we treat them as conspecific in this study.

##### Tetramorium mars

Hita Garcia & Fisher sp. n.

http://zoobank.org/0682CF5D-60A1-40B2-B552-E2A2351FB575

http://species-id.net/wiki/Tetramorium_mars

**Figure 18.**
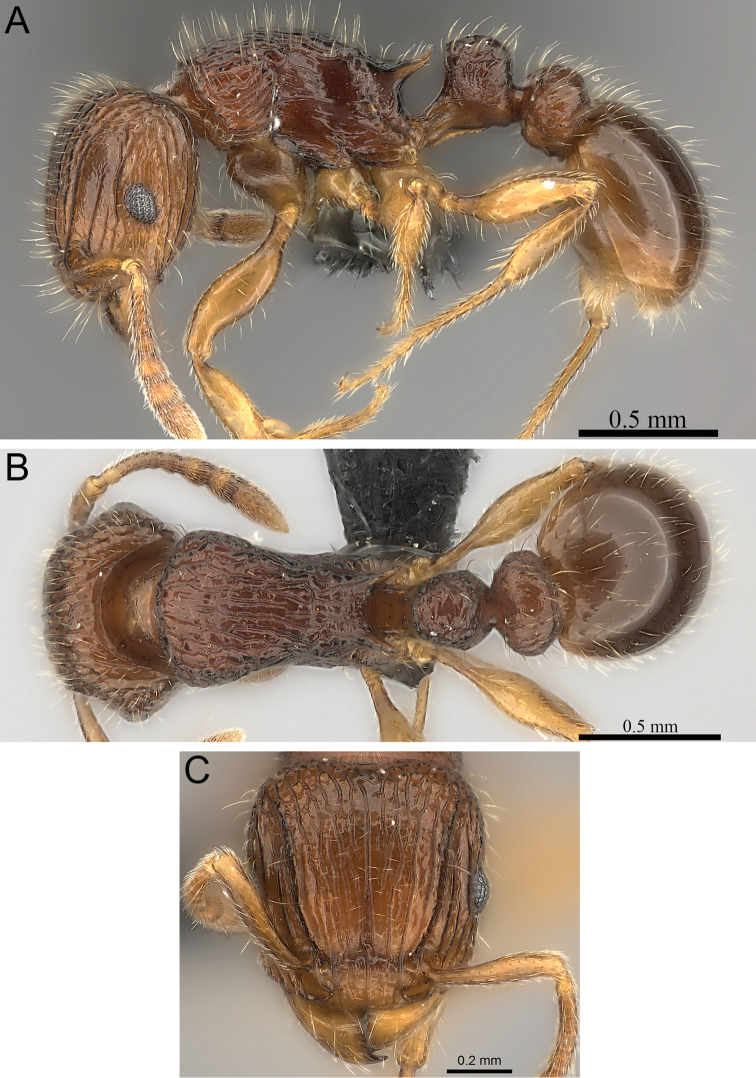
*Tetramorium mars* holotype worker (CASENT0474279). **A** Body in profile **B** Body in dorsal view **C** Head in full-face view.

Figs 10B , 12B , 13A , 18 , 62

###### Type material.

**Holotype**, pinned worker, MADAGASCAR, Toliara, Forêt de Kirindy, 15.5 km 64° ENE Marofandilia, 20.045°S, 44.6622°E, 100 m, tropical dry forest, sifted litter (leaf mold, rotten wood), BLF04605, 28.XI.2001 (*B.L. Fisher et al.*) (CAS: CASENT0474279). **Paratypes**, 24 pinned workers with same data as holotype (BMNH: CASENT0474298; CAS: CASENT0474278; CASENT0474281; CASENT0474287; CASENT0474289; CASENT0474292; CASENT0474294; CASENT0474304; CASENT0474310; CASENT0474312; CASENT0474322; CASENT0474328; CASENT0474331; CASENT0474335; CASENT0474338; CASENT0474345; CASENT0474347; CASENT0474349; CASENT0474354; CASENT0474357; CASENT0474366; CASENT0474371; MHNG: CASENT0474312; MCZ: CASENT0474286; CASENT0474307).

###### Non-type material.

MADAGASCAR: Mahajanga, Parc National d’Ankarafantsika, Forêt de Tsimaloto, 18.3 km 46° NE de Tsaramandroso, 16.2281°S, 47.1436°E, 135 m, tropical dry forest, 2.–8.IV.2001 (*B.L. Fisher et al.*); Mahajanga, Parc National d’Ankarafantsika, Ampijoroa Station Forestiere, 5.4 km 331° NW Andranofasika, 16.2989°S, 46.813°E, 70 m, tropical dry forest, 26.III.-1.IV.2001 (*B.L. Fisher et al.*); Mahajanga, Parc National de Baie de Baly, 12.4 km 337° NNW Soalala, 16.01°S, 45.265°E, 10 m, tropical dry forest, 26.–30.XI.2002 (*B.L. Fisher et al.*); Mahajanga, Parc National Tsingy de Bemaraha, 10.6 km ESE 123° Antsalova, 18.7094°S, 44.7182°E, 150 m, tropical dry forest on tsingy, 16.–20.XI.2001 (*B.L. Fisher et al.*); Toliara, Tulear, Bereboka, 60 km NE Morondava, 18.–23.V.1983 (*J.S. Noyes & M.C. Day*); Toliara, Atsimo Andrefana Region, District of Betioky, 30 km E Betioky, Beza Mahafaly Special Reserve, 23.6865°S, 44.591°E, 165 m, gallery dry deciduous forest, 29.IV.-19.V.2002 (*M. Rin’ha*); Toliara, Res. Beza-Mahafaly, Parcel 1, 23.65833°S, 44.62889°E, 160 m, tropical dry forest, 13.II.1993 (*G.D. Alpert*); Toliara, Res. Beza-Mahafaly, Parcel 1, 23.65°S, 44.63333°E, 130 m, tropical dry forest, 13.XI.1993 (*P.S. Ward*); Toliara, Beza-Mahafaly, 27 km E Betioky, 23.65°S, 44.6333°E, 135 m, tropical dry forest, 23.IV.1997 (*B.L. Fisher*); Toliara, Kirindy Forest, 20.07458°S, 44.67611°E, 100m, tropical dry forest, 20.XII.1993 (*G.D. Alpert*); Toliara, Forêt de Kirindy, 15.5 km 64° ENE Marofandilia, 20.045°S, 44.6622°E, 100 m, tropical dry forest, 28.XI.-3.XII.2001 (*B.L. Fisher et al.*); Toliara, Parc National de Kirindy Mite, 16.3 km 127° SE Belo sur Mer, 20.7953°S, 44.147°E, 80 m, tropical dry forest, 6.–10.XII.2001 (*B.L. Fisher et al.*); Toliara, Forêt de Mite, 20.7 km 29° WNW Tongobory, 23.5242°S, 44.1213°E, 75 m, gallery forest, 27.II.–3.III.2002 (*B.L. Fisher et al.*); Toliara, 48 km ENE Morondava, 20.06667°S, 44.65°E, 30 m, tropical dry forest, 4.–7.I.1991 (*D.M. Olson*).

###### Diagnosis.

*Tetramorium mars* is distinguishable from the other group members by the following combination of characters: antennal scapes very short (SI 58–62); petiolar node never enlarged and massive; node in profile high, rectangular, blocky nodiform with well-defined antero- and posterodorsal margins; anterodorsal margin not protruding anteriorly nor very sharply angled; petiolar node in profile relatively low, between 1.3 to 1.5 times higher than long (LPeI 69–76), in dorsal view only 1.1 to 1.2 times wider than long (DPeI 108–123); gaster never enlarged or swollen; ground sculpture on head and mesosoma weak to absent; first gastral tergite without strong reticulate-rugose sculpture, usually completely unsculptured, but if sculpture present, then relatively weak and restricted to base of tergite; pilosity on first gastral tergite mostly erect.

###### Worker measurements

**(N=12).** HL 0.71–0.77 (0.74); HW 0.69–0.74 (0.72); SL 0.42–0.46 (0.44); EL 0.15–0.17 (0.16); PH 0.35–0.44 (0.38); PW 0.50–0.58 (0.54); WL 0.83–0.96 (0.88); PSL 0.21–0.24 (0.22); PTL 0.22–0.27 (0.24); PTH 0.32–0.37 (0.34); PTW 0.26–0.33 (0.29); PPL 0.22–0.26 (0.24); PPH 0.28–0.33 (0.31); PPW 0.36–0.42 (0.38); CI 96–99 (98); SI 58–62 (60); OI 21–22 (22); DMI 60–64 (62); LMI 42–46 (44); PSLI 28–32 (30); PeNI 51–56 (53); LPeI 69–76 (73); DPeI 108–123 (117); PpNI 68–72 (70); LPpI 75–80 (78); DPpI 156–165 (160); PPI 129–137 (134).

###### Worker description.

Head longer than wide (CI 96–98); posterior head margin weakly concave. Anterior clypeal margin with distinct median impression. Frontal carinae strongly developed and forming dorsal margin of very well-developed antennal scrobes, scrobes moderately to very deep and with clearly defined margins all around; median scrobal carina very well developed and distinctly surpassing posterior eye level. Antennal scapes very short, not reaching posterior head margin (SI 58–62). Eyes relatively small to moderate (OI 21–22). Mesosomal outline in profile moderately convex, rounded and high (LMI 42–46), moderately to strongly marginate from lateral to dorsal mesosoma; promesonotal suture and metanotal groove absent. Propodeal spines long, elongate-triangular to spinose (PSLI 28–32), spines always with broad base and acute tip; propodeal lobes well developed, elongate-triangular, and acute, always much shorter than propodeal spines. Petiolar node in profile high, rectangular, blocky nodiform with well-defined antero- and posterodorsal margins, between 1.3 to 1.5 times higher than long (LPeI 68–76), anterior and posterior faces approximately parallel, antero- and posterodorsal margins situated at about same height, petiolar dorsum weakly convex; node in dorsal view around 1.1 to 1.2 times wider than long (DPeI 108–123), in dorsal view pronotum between 1.8 to 2.0 times wider than petiolar node (PeNI 51–56). Postpetiole in profile subglobular, approximately 1.3 times higher than long (LPpI 75–80); in dorsal view around 1.5 to 1.7 times wider than long (DPpI 156–165), pronotum around 1.4 to 1.5 times wider than postpetiole (PpNI 68–72). Postpetiole in profile appearing much less voluminous than petiolar node, postpetiole in dorsal view around 1.3 to 1.4 times wider than petiolar node (PPI 129–137). Mandibles unsculptured, smooth, and shining; clypeus longitudinally rugose/rugulose, with five to seven distinct rugae/rugulae, median ruga better developed than remainder, all rugae without cross-meshes; cephalic dorsum between frontal carinae with eight to twelve longitudinal rugae, rugae running from posterior clypeal margin to posterior head margin, often interrupted or with cross-meshes, especially posteriorly; scrobal area mostly unsculptured; lateral head mainly longitudinally rugose. Ground sculpture on head generally weak to absent, sometimes a few areas weakly punctate. Mesosoma laterally irregularly longitudinally rugose with a few shining areas on mesopleuron and propodeum, mesosomal dorsum longitudinally rugose. Forecoxae unsculptured, smooth, and shining. Ground sculpture on mesosoma absent. In profile, basal half of petiolar node mostly unsculptured, upper half distinctly reticulate-rugose, whole dorsum of node distinctly reticulate-rugose; sculpture on postpetiole much more weakly developed, laterally and dorsally weakly irregularly rugulose. Ground sculpture on waist segments variable, usually weak or absent, often petiole and/or postpetiole conspicuously reticulate-punctate. Gaster usually unsculptured, smooth, and shining, sometimes base of first gastral tergite weakly punctate. Whole body with very abundant, long, and fine standing hairs; first gastral tergite with abundant, long, erect hairs and much scarcer, shorter, decumbent to subdecumbent hairs. Anterior edges of antennal scapes and dorsal (outer) surfaces of hind tibiae with decumbent to suberect hairs. Usually body uniformly light to reddish brown, sometimes darker.

###### Etymology.

This new species is named after the ancient Roman god of war, “Mars”. The species epithet is an arbitrary combination of letters, thus invariable.

###### Distribution and biology.

*Tetramorium mars* has a broad distribution in western Madagascar (Fig. 62). It ranges from the southernmost known localities, Beza-Mahafaly and Forêt de Mite, through Kirindy Mite and Tsingy de Bemaraha to the northernmost localities, Baie de Baly and Ankarafantsika. All known localities are tropical dry or gallery forests at low elevations from 10 to 165 m. The new species was mainly sampled by litter extraction or pitfall trapping, which suggests a ground active lifestyle. Interestingly, *Tetramorium mars* was not found in Andohahela in the southeast of Madagascar, even though *Tetramorium bressleri*, *Tetramorium hobbit*, and *Tetramorium plesiarum* occur there. This is surprising since the four species of the group except *Tetramorium gollum* share more or less the same distribution range.

###### Discussion.

Within the species group *Tetramorium mars* is not likely to be confused with the other five species. The lack of strong and conspicuous sculpture on the basal half of the first gastral tergite, an enlarged gaster, and the lower and less broad petiolar node separate *Tetramorium mars* (LPeI 69–76; DPeI 108–123) clearly from *Tetramorium gollum* (LPeI 57–60; DPeI 130–148). Despite the fact that *Tetramorium mars* shares some similarities with *Tetramorium bressleri* and *Tetramorium plesiarum*, the latter also have relatively higher, thinner, and wider petiolar nodes (LPeI 56–63; DPeI 130–144) which distinguish them from *Tetramorium mars*. In addition, *Tetramorium mars* has much better developed sculpture on the dorsum of the petiolar node than *Tetramorium bressleri* or *Tetramorium plesiarum*. The species most similar morphologically to *Tetramorium mars* is *Tetramorium hobbit*. Both share a more blocky nodiform petiolar node, but which is still much larger in *Tetramorium hobbit*. Indeed, this massively developed node, in combination with very conspicuous punctate ground sculpture on head and mesosoma, separates *Tetramorium hobbit* from *Tetramorium mars*, which has a much smaller petiolar node and only weakly developed ground sculpture on head and mesosoma. Further characters that distinguish *Tetramorium mars* are the comparatively short antennal scapes (SI 58–62 vs. SI 64–67) and smaller body size (HW 0.69–0.74 vs. HW 0.80–0.88).

Despite its relatively broad distribution range, *Tetramorium mars* displays very little intraspecific or geographic variation.

##### Tetramorium plesiarum

Bolton

http://species-id.net/wiki/Tetramorium_plesiarum

**Figure 19.**
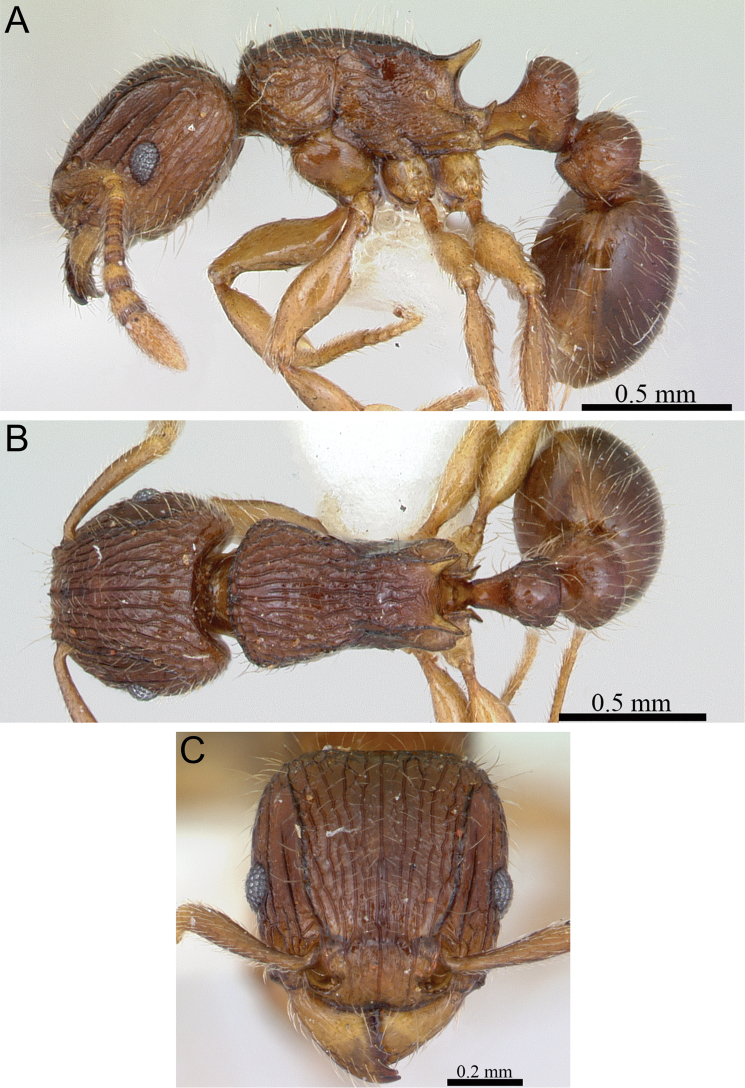
*Tetramorium plesiarum* holotype worker (CASENT0172831). **A** Body in profile **B** Body in dorsal view **C** Head in full-face view.

Figs 10C , 11B , 13B , 14A , 19 , 62

Tetramorium plesiarum Bolton, 1979: 150.

###### Type material.

**Holotype**, pinned worker, MADAGASCAR, Causse de Kelifely, 17.30306°S, 45.73444°E, forest humus and litter, dry forest, 20.–30.XI.1974 (*A. Peyrieras*) (MCZ: MCZ_Holotype_32372) [examined].

[Note: the GPS data of the type locality was not provided by the locality label or the original description. The data presented above is based on our own geo-referencing of the Kelifely limestone plateau and should be considered an approximation but not the exact position of the type locality.]

###### Non-type material.

MADAGASCAR: Mahajanga, Reserve Forestiere Beanka, 50.7 km E Maintirano, 17.8802°S, 44.4688°E, 140 m, tropical dry forest on tsingy, 29.X.–1.XI.2009 (*B.L. Fisher et al.*); Mahajanga, Parc National de Namoroka, 9.8 km 300° WNW Vilanandro, 16.4667°S, 45.35°E, 140 m, tropical dry forest, 4.–8.XI.2002 (*B.L. Fisher et al.*); Mahajanga, Parc National de Namoroka, 17.8 km 329° WNW Vilanandro, 16.3767°S, 45.3267°E, 100 m, tropical dry forest, 8.–12.XI.2002 (*B.L. Fisher et al.*); Mahajanga, Parc National de Namoroka, 16.9 km 317° NW Vilanandro, 16.4067°S, 45.31°E, 100 m, tropical dry forest, 12.–16.XI.2002 (*B.L. Fisher et al.*); Mahajanga, Parc National Tsingy de Bemaraha, 10.6 km ESE 123° Antsalova, 18.7094°S, 44.7182°E, 150 m, tropical dry forest on tsingy, 16.–20.XI.2001 (*B.L. Fisher et al.*); Toliara, Parc National d’Andohahela, Forêt de Manantalinjo, 33.6 km 63° ENE Amboasary, 7.6 km 99° E Hazofotsy, 24.8169°S, 46.61°E, 150 m, spiny forest/thicket, 12.–16.I.2002 (*B.L. Fisher et al.*); Toliara, Parc National d’Andohahela, Forêt d’Ambohibory, 1.7 km 61° ENE Tsimelahy, 36.1 km 308° NW Tolagnaro, 24.93°S, 46.6455°E, 300 m, tropical dry forest, 16.–20.I.2002 (*B.L. Fisher et al.*); Toliara, Andohahela National Park, Tsimelahy–Parcel II, 24.9368°S, 46.6267°E, 180 m, transition forest, 5.–15.II.2003 (*M.E. Irwin et al.*); Toliara, Makay Mts., 21.2184°S, 45.3106°E, 510 m, gallery forest on sandy soil, 24.–27.XI.2010 (*B.L. Fisher et al.*); Toliara, Makay Mts., 21.2176°S, 45.3392°E, 500 m, gallery forest on sandy soil, 28.XI.2010 (*B.L. Fisher et al.*); Toliara, Makay Mts., 21.3136°S, 45.1478°E, 525 m, gallery forest on sandy soil, 6.XII.2010 (*B.L. Fisher et al.*); Toliara, Vohibasia Forest, 59 km NE Sakaraha, 22.4667°S, 44.85°E, 780 m, tropical dry forest, 13.I.1996 (*B.L. Fisher*); Toliara, Parc National de Zombitse, 19.8 km 84° E Sakaraha, 22.8433°S, 44.71°E, 770 m, tropical dry forest, 5.–9.II.2003 (*B.L. Fisher et al.*).

###### Diagnosis.

The following character set separates *Tetramorium plesiarum* from the remainder of the species group: smaller species (HW 0.80–1.00; WL 0.92–1.15); eyes of moderate size (OI 21–23); petiolar node high nodiform, not massively enlarged, anterodorsal margin protruding anteriorly and slightly more marginate than posterodorsal margin; petiolar node in profile relatively high and thin, between 1.6 to 1.8 times higher than long (LPeI 56–63), in dorsal view between 1.3 to 1.4 times wider than long (DPeI 131–137); gaster never extremely enlarged and swollen; head without strongly developed and conspicuous reticulate-punctate ground sculpture; mesopleuron and lateral propodeum with moderate punctate ground sculpture; first gastral tergite without strong reticulate-rugose sculpture, usually completely unsculptured, but if sculpture present, then relatively weak and restricted to base of tergite; pilosity on first gastral tergite mostly erect.

###### Worker measurements

**(N=12).** HL 0.66–0.75 (0.73); HW 0.67–0.76 (0.72); SL 0.45–0.49 (0.47); EL 0.15–0.17 (0.15); PH 0.33–0.44 (0.36); PW 0.50–0.57 (0.53); WL 0.81–0.92 (0.85); PSL 0.16–0.21 (0.18); PTL 0.18–0.22 (0.19); PTH 0.31–0.35 (0.33); PTW 0.23–0.29 (0.26); PPL 0.22–0.26 (0.24); PPH 0.29–0.33 (0.31); PPW 0.32–0.40 (0.35); CI 97–102 (99); SI 64–69 (66); OI 21–23 (22); DMI 60–65 (63); LMI 41–47 (43); PSLI 23–28 (25); PeNI 45–51 (49); LPeI 56–63 (59); DPeI 131–137 (133); PpNI 63–70 (66); LPpI 73–79 (76); DPpI 140–157 (149); PPI 129–143 (136).

###### Worker description.

Head more or less as long as broad (CI 97–102); posterior head margin weakly concave. Anterior clypeal margin with distinct median impression. Frontal carinae strongly developed and forming dorsal margin of very well-developed antennal scrobes, scrobes moderately to very deep and with clearly defined margins all around; median scrobal carina very well developed and distinctly surpassing posterior eye level, usually ending halfway between posterior eye margin and posterior scrobe margin, often approaching posterior scrobe margin. Antennal scapes short, not reaching posterior head margin (SI 64–69). Eyes small to moderate (OI 21–23). Mesosomal outline in profile weakly to moderately convex, rounded and high (LMI 41–47), moderately marginate from lateral to dorsal mesosoma; promesonotal suture and metanotal groove absent. Propodeal spines elongate-triangular to spinose, moderately long to long, and acute (PSLI 23–28), spines always with broad base and acute tip; propodeal lobes well developed, triangular to elongate-triangular, and acute, always much shorter than propodeal spines. Petiolar node in profile high nodiform, between 1.6 to 1.8 times higher than long (LPeI 56–63), anterodorsal margin situated higher and more angled than posterodorsal, more rounded margin, petiolar dorsum weakly to moderately tapering backwards posteriorly, anterodorsal margin slightly protruding anteriorly, anterior and posterior faces approximately parallel; node in dorsal view between 1.3 to 1.4 times wider than long (DPeI 131–137), pronotum between 1.9 to 2.2 times wider than petiolar node (PeNI 45–51). Postpetiole in profile subglobular, approximately 1.3 to 1.4 times higher than long (LPpI 73–79); in dorsal view around 1.4 to 1.6 times wider than long (DPpI 140–157), in dorsal view pronotum between 1.4 to 1.6 times wider than postpetiole (PpNI 63–70). Postpetiole in profile appearing slightly less voluminous than petiolar node, postpetiole in dorsal view about 1.3 to 1.4 times wider than petiolar node (PPI 129–143). Mandibles unsculptured, smooth, and shining; clypeus longitudinally rugose, with three to five distinct rugae, median ruga better developed than remainder, rugae without cross-meshes; cephalic dorsum between frontal carinae with ten to twelve longitudinal rugae, rugae running from posterior clypeal margin to posterior head margin, often interrupted or with cross-meshes, especially posteriorly; scrobal area mostly unsculptured, relatively smooth and shiny; lateral head longitudinally rugose to reticulate-rugose. Ground sculpture on head usually present but weak. Mesosoma laterally irregularly longitudinally rugose, usually with moderate punctate ground sculpture on mesopleuron and propodeum, dorsal mesosoma longitudinally rugose without any distinct ground sculpture, spaces between rugae smooth and shining. Forecoxae unsculptured, smooth, and shining. Sides of petiolar node and postpetiole with conspicuous, moderate reticulate-punctate ground sculpture, both nodes appearing relatively matte; petiolar node and postpetiole dorsally weakly rugulose/rugose, but mostly unsculptured, and appearing smooth and shining. Gaster usually unsculptured, smooth and shining, sometimes base of first gastral tergite with traces of punctate ground sculpture. Whole body with very abundant, dense, moderately long, and fine standing hairs; first gastral tergite with abundant, long, erect hairs and much scarcer, shorter, decumbent to subdecumbent hairs. Anterior edges of antennal scapes and dorsal (outer) surfaces of hind tibiae with decumbent to suberect hairs. Body of uniform brown, appendages often lighter.

###### Distribution and biology.

Despite being known only from seven localities and fewer than 20 specimens, *Tetramorium plesiarum* has a comparatively large distribution range (Fig. 62). However, the populations appear to be relatively disjunctive. The species is known from Andohahela in the southeast of Madagascar and from six additional, more or less scattered localities in the western part of the island ranging from Zombitse and Vohibatsia north through the Makay Mountains, Tsingy de Bemaraha, and Beanka to the Kelifely plateau and Namoroka. The small number of specimens suggests that *Tetramorium plesiarum* is much less common than the sympatric *Tetramorium bressleri*, which shares much of the distribution range, but is known from many more localities and hundreds of specimens. The rarity of *Tetramorium plesiarum* might well explain the disjunctive distribution. Unfortunately, there is no “fresh” material available from the type locality since the holotype remains the only known specimen from the Kelifely plateau. All localities are tropical dry forests, tropical dry forests on tsingy, or gallery forests ranging from 100 to 780 m elevation. Like the other group members *Tetramorium plesiarum* was mostly collected by pitfall trapping and litter sifting suggesting a ground-active lifestyle.

###### Discussion.

*Tetramorium plesiarum* lacks strong specialisations, such as the strongly sculptured and enlarged gaster of *Tetramorium gollum*, or the massively developed petiolar node of *Tetramorium hobbit*. Furthermore, *Tetramorium mars* has a lower and wider petiolar node (LPeI 69–76; DPeI 108–123) compared to *Tetramorium plesiarum* (LPeI 56–63; DPeI 131–137). The latter also has much less sculpture on the dorsum of the petiolar node. Within the species group *Tetramorium plesiarum* is morphologically most similar to *Tetramorium bressleri*, and as mentioned in the description of the latter, we have treated them as conspecific through most of the revision The most noticeable difference is certainly body size since *Tetramorium bressleri* (HW 0.80–1.00; WL 0.92–1.15) is much larger than *Tetramorium plesiarum* (HW 0.80–1.00; WL 0.92–1.15). As noted above, there is some slight overlap caused by one large specimen of *Tetramorium plesiarum* and very few small specimens of *Tetramorium bressleri*, but in the main they fit their respective size ranges. Not considering body size, they also differ in eye size and sculpture on mesosoma and waist segments. The eyes of *Tetramorium plesiarum* (OI 21–23) are larger than in *Tetramorium bressleri* (OI 18–19). The mesopleuron and lateral propodeum of *Tetramorium bressleri* is usually mostly unsculptured and relatively smooth and shining, whereas this area is longitudinally rugose with reticulate-punctate ground sculpture in *Tetramorium plesiarum*. Almost the same pattern is seen in the sculpture on the lateral petiolar node as it is significantly more reticulate-punctate and matte in *Tetramorium plesiarum* than in *Tetramorium bressleri*, in which the weak reticulate-punctate ground sculpture appears faintly matte but still relatively smooth and shiny. The sculpture on the sides of the petiolar node varies, too, since it is much more reticulate-punctate and matte in *Tetramorium plesiarum* than in *Tetramorium bressleri*, in which the weak reticulate-punctate ground sculpture looks faintly matte but still relatively smooth and shiny. The shape of the petiolar node in profile is an additional useful character. Finally, in *Tetramorium plesiarum* the anterodorsal margin of the petiolar node protrudes anteriorly and is slightly more marginate than the more rounded posterodorsal margin, contrasting with the shape observed in *Tetramorium bressleri*, in which both margins are usually equally marginate and the anterodorsal margin does not protrude anteriorly at all.

### Revision of the *Tetramorium schaufussii* species group

#### *Tetramorium schaufussii* species group

**Diagnosis.** Eleven-segmented antennae; anterior clypeal margin medially impressed; frontal carinae variably developed, always present and surpassing eye level, usually weak to moderate and posteriorly merging into the surrounding sculpture, in a few species very well developed, noticeably raised and approaching posterior head margin; antennal scrobes usually weak, shallow, and without clearly defined posterior and ventral margins; anterior face of mesosoma weakly developed; mesosomal outline in profile relatively flat, usually comparatively low and elongated (LMI 35–42), usually moderately marginate from lateral to dorsal mesosoma; propodeal spines usually short to moderately long, triangular to elongate-triangular, long and spinose in a few species (PSLI 7–28); propodeal lobes usually triangular and short; petiolar node in profile rounded nodiform to high rounded nodiform with well-rounded margins, rarely high cuneiform or squamiform, node in profile between 1.2 to 3.0 times higher than long (LPeI 33–81), in dorsal view usually wider than long (DPeI 111–238), rarely longer than wide (DPeI 87–98), anterior and posterior faces parallel in most species; postpetiole in profile globular to subglobular, 1.2 to 1.7 times higher than long (LPeI 60–83), in dorsal view between 1.2 to 1.7 times wider than long (DPpI 120–168); mandibles always unsculptured, smooth, and shining; cephalic sculpture distinct, between frontal carinae predominantly longitudinally rugose; mesosoma usually with well-developed longitudinally rugose/rugulose sculpture, in some species sculpture mainly irregularly rugose/rugulose, rarely irregularly rugose/rugulose to reticulate-rugose; waist segments and gaster unsculptured, smooth, and shiny; standing pilosity always present on head, but variable on remainder of body, first gastral tergite often without standing pilosity at all, appressed pubescence on first gastral tergite variably developed, varying from very short and scarce to long and dense; sting appendage spatulate.

**Comments.** With 20 recognised species the *Tetramorium schaufussii* species group is second in terms of species-richness in the Malagasy region, following the *Tetramorium tortuosum* group with 22 (Hita Garcia and Fisher 2012b). This group is of special importance for Madagascar since many of its members are very widespread, common, and/or abundant. Almost all species prefer rainforests or montane rainforests, and with few exceptions, live in the forest leaf litter stratum.

The *Tetramorium schaufussii* species group is a very distinctive group among the thirteen groups with eleven-segmented antennae, and most of its members are unlikely to be mistaken for any other group. In general, most *Tetramorium schaufussii* group species are small to medium-sized ants, have a comparatively low and elongated mesosoma, and short to very short propodeal spines. Even though these characters are not unique to this group its members are usually easily recognisable. One interesting feature of the *Tetramorium schaufussii* group is that the mandibles of all species are completely unsculptured, smooth, and shining. This character alone already separates the *Tetramorium schaufussii* group from the *Tetramorium bessonii*, *Tetramorium bonibony*, *Tetramorium kelleri*, *Tetramorium tortuosum*, and *Tetramorium weitzeckeri* groups, in which all species have noticeably sculptured mandibles. In most other groups this character, however, is variable. Some species of the *Tetramorium dysalum* group have unsculptured mandibles, but these have either much longer propodeal spines or a much higher mesosoma. Another important group character is the lack of sculpture on the waist segments. The *Tetramorium schaufussii* group shares this character with a number of other groups, but it does distinguish the group from the *Tetramorium kelleri*, *Tetramorium ranarum*, and *Tetramorium tortuosum* groups, as well as from parts of the *Tetramorium bonibony* and *Tetramorium dysalum* groups. All these groups have at least one waist segment with distinct sculpture, but most of their species have conspicuous sculpture on both. Furthermore, in the *Tetramorium schaufussii* group the antennal scrobe is either weakly developed or absent distinguishing it immediately from the *Tetramorium plesiarum* group and a few species of the *Tetramorium ibycterum* complex of the *Tetramorium ranarum* group with moderately to very deep scrobes with clearly defined margins all around.

Moreover, all species of the *Tetramorium schaufussii* species group have relatively well developed cephalic and mesosomal sculpture. The sculpture on the cephalic dorsum between the frontal carinae is always well developed, whereas sculpture on the mesosomal dorsum can be weak in some species (but remains distinctly present). This separates the group from the *Tetramorium bessonii*, *Tetramorium marginatum*, *Tetramorium tsingy* groups, which usually have strongly reduced sculpture on the head and mesosoma. The members of the *Tetramorium marginatum* group that possess more sculpture have strongly cuneiform and/or triangular petiolar nodes, and are thus not confusable with the *Tetramorium schaufussii* group. The groups being morphologically most similar to the *Tetramorium schaufussii* group are the *Tetramorium naganum* and *Tetramorium severini* groups, as they share the usually high nodiform petiolar node and completely unsculptured waist segments. The only previously known species of both groups, *Tetramorium naganum* and *Tetramorium severini*, were initially recognised as members of the *Tetramorium schaufussii* group by Bolton (1979), but recently split by Hita Garcia and Fisher (2011, 2012a, 2012b). The separation from the *Tetramorium severini* group is relatively straightforward. The latter species is very large, very darkly coloured, and has very long to extremely long and curved propodeal spines that easily distinguish its members from the *Tetramorium schaufussii* group. The distinction between the *Tetramorium schaufussii* and the *Tetramorium naganum* group is less clear. The members of the *Tetramorium naganum* group usually have much more strongly developed frontal carinae, a generally broader head (CI 92–99), a higher mesosoma (LMI 40–46), and usually longer propodeal spines (PSLI 25–37). Some of these values overlap with a few species of *Tetramorium schaufussii* group, but they separate the bulk of species comparatively well.

Bolton (1979) divided the group into two complexes on the basis of the absence (*Tetramorium cognatum* complex) or presence (*Tetramorium schaufussii* complex) of long, standing pilosity on the first gastral tergite. We follow this division and have placed all the new species into these two complexes even though at present we have no more evidence that these complexes represent monophyletic, mutually exclusive clades or lineages within the species group. Indeed, the only separating character is gastral pilosity, and future studies on the group that ideally include molecular data will likely yield different groupings. However, with 20 species the group is relatively species-rich and the clear-cut division into these two complexes facilitates the taxonomic organisation of the species group.

##### Key to species complexes of the *Tetramorium schaufussii* species group

**Table:** 

1	First gastral tergite with appressed pubescence of varying length and without any standing hairs (Fig. 20A, B) [Madagascar and Comoros]	*Tetramorium cognatum* species complex
–	First gastral tergite with appressed pubescence of varying length and few to numerous standing hairs (Fig. 20C, D) [Madagascar and Reunion]	*Tetramorium schaufussii* species complex

**Figure 20.**
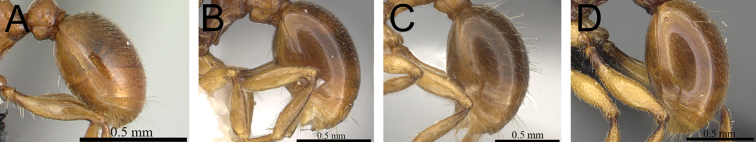
First gastral tergite in profile. **A** *Tetramorium karthala* (CASENT0136774) **B** *Tetramorium proximum* (CASENT0906146) **C** *Tetramorium merina* (CASENT0437232) **D** *Tetramorium obiwan* (CASENT0447245).

##### *Tetramorium cognatum* species complex

**Comments.** This species complex contains a compact assemblage of ten species that are separated from the *Tetramorium schaufussii* species complex by their lack of any long, standing pilosity on the first gastral tergite, a character present in all members of the *Tetramorium schaufussii* complex. The ten species of the *Tetramorium cognatum* complex might be further divided into three smaller groupings: the first of these contains the five small to moderately large species with weak to very weak frontal carinae (*Tetramorium aspis*, *Tetramorium camelliae*, *Tetramorium cognatum*, *Tetramorium karthala*, and *Tetramorium rumo*); the second includes the four larger species with very well developed frontal carinae (*Tetramorium gladius*, *Tetramorium myrmidon*, *Tetramorium proximum*, and *Tetramorium tenuinode*); the third sub-grouping contains only *Tetramorium freya*, which is the only species of the complex lacking standing pilosity on the mesosoma, but it also possesses reduced frontal carinae and a larger body size.

Four species of the complex have very restricted distribution ranges and are endemics to smaller areas or localities in Madagascar or the Comoros (Figs 63, 64): *Tetramorium aspis* (Andringitra and Ivohibe), *Tetramorium camelliae* (Ranomafana), *Tetramorium karthala* (Grand Comore), and *Tetramorium myrmidon* (Ambohijanahary), while *Tetramorium freya* was found only in a few littoral localities ranging from Nosy Be to the northernmost tip of Madagascar. The remaining species (*Tetramorium cognatum*, *Tetramorium gladius*, *Tetramorium proximum*, *Tetramorium rumo*, and *Tetramorium tenuinode*) have much wider distribution ranges, especially *Tetramorium cognatum* and *Tetramorium proximum*, which are found in almost all rainforests and montane rainforests from which material is available. Interestingly, almost all species appear be restricted to or prefer rainforests or montane rainforests, even the widely distributed *Tetramorium cognatum*, *Tetramorium proximum*, and *Tetramorium tenuinode*. However, *Tetramorium cognatum* was occasionally collected in drier habitats and *Tetramorium freya* occurs also in littoral and dry forests.

###### Identification key to species of the *Tetramorium cognatum* species complex (workers)

**Table:** 

1	Mesosoma without any long, standing hairs (Fig. 21A) [Madagascar]	*Tetramorium freya*
–	Mesosoma always with few to numerous pairs of standing hairs (Fig. 21B, C)	2
2	Petiolar node strongly squamiform, in profile 2.8 to 3.0 times higher than long (LPeI 33–36) and in dorsal view around 2.3 to 2.4 times wider than long (DPeI 228–238) (Fig. 22A) [Madagascar]	*Tetramorium camelliae*
–	Petiolar node never strongly squamiform as above, in profile 1.6 to 2.7 (usually 1.6 to 2.2) times higher than long (LPeI 37–62) and in dorsal view between 1.1 to 1.7 times wider than long DPeI 111–171) (Fig. 22B, C, D)	3
3	Eyes relatively small (OI 19–20) (Fig. 23A) [Madagascar]	*Tetramorium gladius*
–	Eyes always much larger than above (OI 24–31) (Fig. 23B, C, D)	4
4	Larger species (HW 0.58–0.81; WL 0.85–1.07); frontal carinae moderately well to strongly developed, noticeably raised, and usually approaching or ending at posterior head margin (Fig. 24A, B)	5
–	Smaller species (HW 0.43–0.55; WL 0.56–0.81); frontal carinae usually weakly developed, faintly raised, and mostly ending or fading into surrounding sculpture halfway between posterior eye margin and posterior head margin (Fig. 24C, D)	7
5	Usually significantly larger species (HW 0.65–0.81; WL 0.92–1.07); mesosoma usually with five or six pairs of long, standing hairs on pronotum and mesonotum (Fig. 25A) [Madagascar]	*Tetramorium proximum*
–	Usually smaller species (HW 0.58–0.70; WL 0.76–0.94); mesosoma always with only two pairs of long, standing hairs, one pair on anterior pronotum and one on anterior mesonotum (Fig. 25B, C)	6
6	Antennal scapes shorter (SI 66–70); petiolar node higher, around 1.8 to 2.2 times higher than long (LPeI 45–54); postpetiole in dorsal view around 1.5 to 1.7 times broader than petiolar node (PPI 148–167) (Fig. 26A, B) [Madagascar]	*Tetramorium tenuinode*
–	Antennal scapes longer (SI 74–76); petiolar node lower, around 1.6 to 1.7 times higher than long (LPeI 58–60) postpetiole in dorsal view only around 1.3 to 1.4 times broader than petiolar node (PPI 133–141) (Fig. 26C) [Madagascar]	*Tetramorium myrmidon*
7	Smaller species (HW 0.43–0.49; WL 0.56–0.67); propodeal spines moderately long to long, elongate-triangular to spinose (PSLI 22–26); petiolar node thinly cuneiform and moderately squamiform, in profile around 2.3 to 2.7 times higher than long (LPeI 37–43), in dorsal view around 1.6 to 1.7 times wider than long (DPeI 156–171); colouration always uniformly whitish yellow to light yellowish brown (Fig. 27A) [Madagascar]	*Tetramorium rumo*
–	Larger species (HW 0.48–0.55; WL 0.65–0.81); propodeal spines short to moderately long, triangular to elongate-triangular (PSLI 12–22); petiolar node high nodiform, in profile around 1.8 to 2.2 times higher than long (LPeI 46–55), in dorsal view 1.3 to 1.6 times wider than long (DPeI 129–161); colouration variable, but rarely yellowish as above (Fig. 27B, C)	8
8	Antennal scapes shorter (SI 61–67); propodeal spines reduced and very short (PSLI 12–16), propodeal spines and lobes often of same length or spines even shorter, never strongly directed towards each other; petiolar node in dorsal view around 1.3 to 1.4 times wider than long (DPeI 129–142) (Fig. 28A, B) [Madagascar]	*Tetramorium cognatum*
–	Antennal scapes longer (SI 68–72); propodeal spines short to moderately long (PSLI 18–22), spines either much longer than lobes or only weakly so and spines and lobes strongly inclined towards each other; petiolar node in dorsal view around 1.5 to 1.6 times wider than long (DPeI 146–161) (Fig. 28C, D)	9
9	Eyes smaller (OI 25–27); propodeal lobes always only weakly shorter than propodeal spines, and spines and lobes strongly inclined towards each other; dorsal mesosoma with six or more pairs of long, standing hairs on pronotum and mesonotum, and propodeum usually with one or two pairs anteriorly (Fig. 29A) [Madagascar]	*Tetramorium aspis*
–	Eyes larger (OI 29–30); propodeal lobes short and triangular, always much shorter than propodeal spines, and spines and lobes never strongly inclined towards each other; dorsal mesosoma with two to four pairs of long, standing hairs on pronotum and mesonotum, standing hairs absent from propodeum (Fig. 29B) [Comoros]	*Tetramorium karthala*

**Figure 21.**
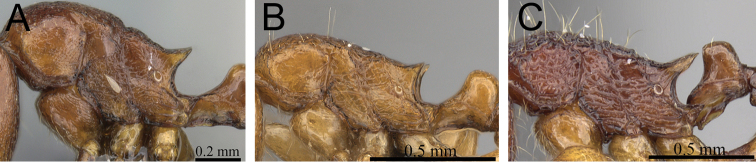
Mesosoma in profile. **A** *Tetramorium freya* (CASENT0085551) **B** *Tetramorium cognatum* (CASENT0217758) **C** *Tetramorium gladius* (CASENT0163234).

**Figure 22.**
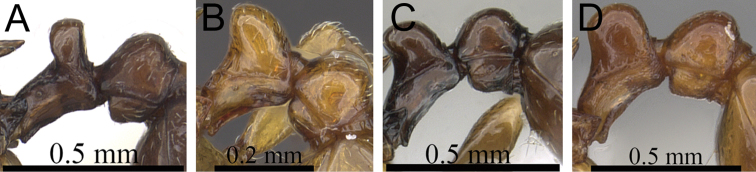
Waist segments in profile. **A** *Tetramorium camelliae* (CASENT0247496) **B** *Tetramorium rumo* (CASENT0073025) **C** *Tetramorium aspis* (CASENT0344940) **D** *Tetramorium proximum* (CASENT0906146).

**Figure 23.**
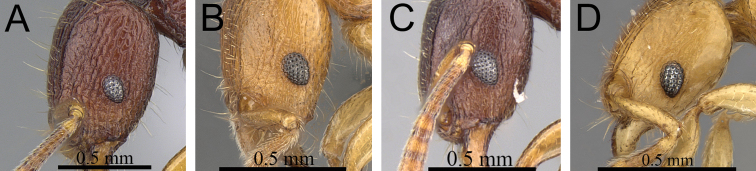
Head in profile. **A** *Tetramorium gladius* (CASENT0406982) **B** *Tetramorium cognatum* (CASENT0067891) **C** *Tetramorium myrmidon* (CASENT0028635) **D** *Tetramorium rumo* (CASENT0217759).

**Figure 24.**
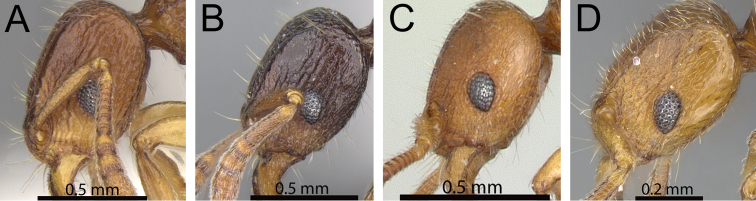
Head in profile. **A** *Tetramorium proximum* (CASENT0906146) **B** *Tetramorium tenuinode* (CASENT0040115) **C** *Tetramorium karthala* (CASENT0136774) **D** *Tetramorium rumo* (CASENT0073025).

**Figure 25.**
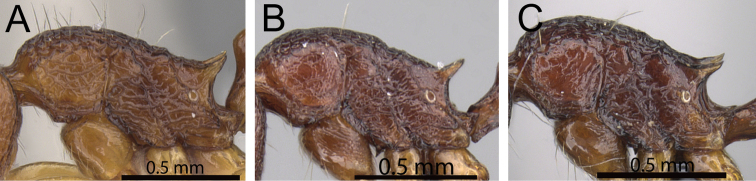
Mesosoma in profile. **A** *Tetramorium proximum* (CASENT0906146) **B** *Tetramorium myrmidon* (CASENT0028635) **C** *Tetramorium tenuinode* (CASENT0040115).

**Figure 26.**
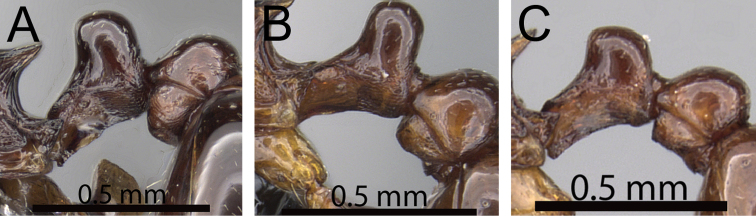
Waist segments in profile. **A** *Tetramorium tenuinode* (CASENT0162682) **B** *Tetramorium tenuinode* (CASENT0040115) **C** *Tetramorium myrmidon* (CASENT0028635).

**Figure 27.**
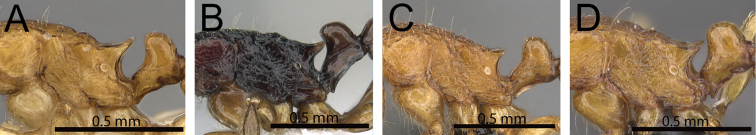
Mesosoma and petiole in profile. **A** *Tetramorium rumo* (CASENT0217759) **B** *Tetramorium aspis* (CASENT0344940) **C** *Tetramorium cognatum* (CASENT0067891) **D** *Tetramorium karthala* (CASENT0137302).

**Figure 28.**
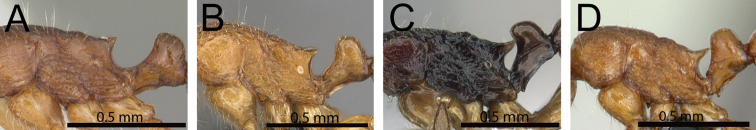
Mesosoma and petiole in profile. **A** *Tetramorium cognatum* (CASENT0102343) **B** *Tetramorium cognatum* (CASENT0067891) **C** *Tetramorium aspis* (CASENT0344940) **D** *Tetramorium karthala* (CASENT0136774).

**Figure 29.**
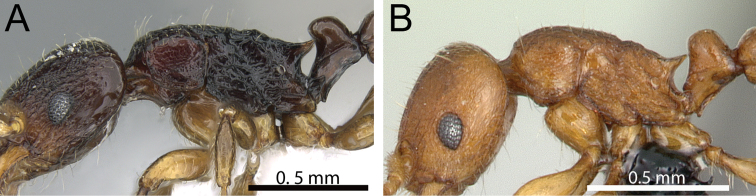
Head and mesosoma in profile. **A** *Tetramorium aspis* (CASENT0344940) **B** *Tetramorium karthala* (CASENT0136774).

###### Tetramorium aspis

Hita Garcia & Fisher sp. n.

http://zoobank.org/018A7596-2EC0-4213-B928-D034D77868B4

http://species-id.net/wiki/Tetramorium_aspis

**Figure 30.**
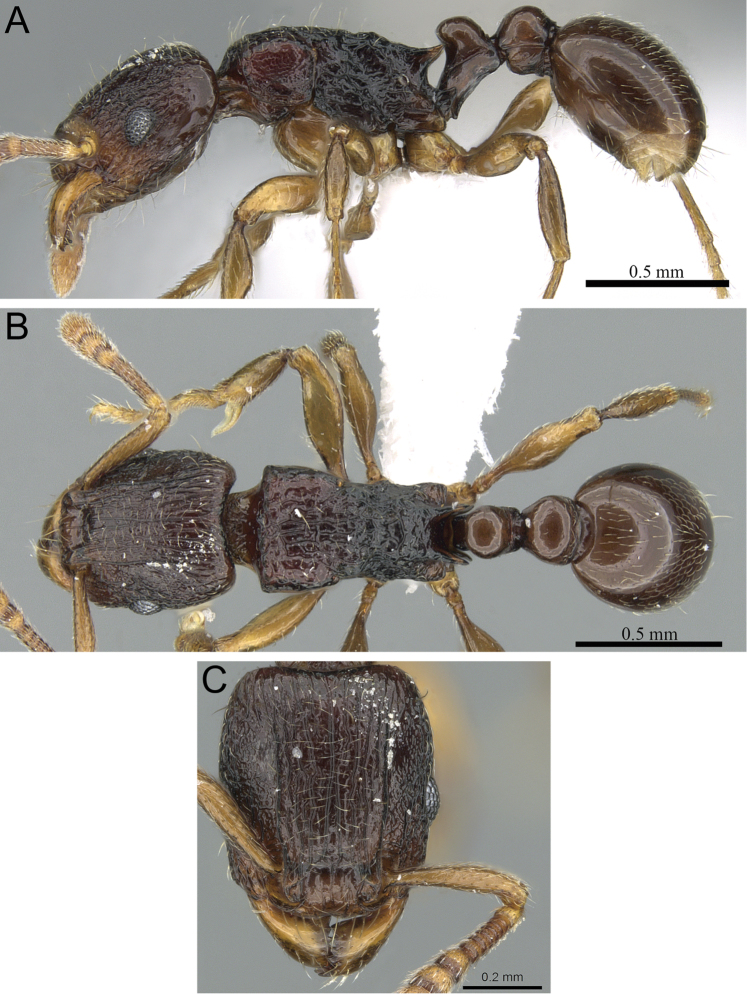
*Tetramorium aspis* holotype worker (CASENT0344940). **A** Body in profile **B** Body in dorsal view **C** Head in full-face view.

Figs 22C , 27B , 28C , 29A , 30 , 63

####### Type material.

**Holotype**, pinned worker, MADAGASCAR, Fianarantsoa, R.S. Ivohibe 8.0 km E Ivohibe, 22.48333°S, 46.96833°E, 1200 m, montane rainforest, sifted litter, (leaf mold, rotten wood), collection code BLF01747, 15.–21.X.1997 (*B.L. Fisher*) (CAS: CASENT0344940). **Paratypes**, eleven workers with same data as holotype (CAS: CASENT0189115; CASENT0198990; CASENT0198991; CASENT0198995; CASENT0198998; CASENT0217756; CASENT0247396; CASENT0247397; CASENT0247398; CASENT0247399; CASENT0247400); three workers with same data as holotype except sampled from rotten stick on ground and collection code BLF01746 (CAS: CASENT0189124); fourteen workers from Fianarantsoa, 8.0 km NE Ivohibe, 22.4217°S, 46.8983°E, 1200 m, montane rainforest, sifted litter, (leaf mold, rotten wood), collection codes BLF01751 and BLF01753, 3.–9.XI.1997 (*B.L. Fisher*) (BMNH: CASENT0247403; CAS: CASENT0189123; CASENT0189156; CASENT0198992; CASENT0198993; CASENT0198994; CASENT0198996; CASENT0247402; CASENT0247404; CASENT0247405; MCZ: CASENT0247401).

####### Non-type material.

MADAGASCAR: Fianarantsoa, 45 km S Ambalavao, 22.21667°S, 47.01667°E, 785 m, rainforest, 25.IX.1993 (*B.L. Fisher*); Fianarantsoa, Rés. Andringitra 43 km S Ambalavao, 22.23333°S, 47°E, 825 m, rainforest, 5.X.1993 (*B.L. Fisher*); Fianarantsoa, Rés. Andringitra 40 km S Ambalavao, 22.21667°S, 46.96667°E, 1275 m, montane rainforest, 15.X.1993 (*B.L. Fisher*); Fianarantsoa, Rés. Andringitra 38 km S Ambalavao, 22.2°S, 46.96667°E, 1680 m, montane rainforest, 23.X.1993 (*B.L. Fisher*); Fianarantsoa, R.S. Ivohibe, 7.5 km ENE Ivohibe, 22.47°S, 46.96°E, 900 m, rainforest, 7.–12.X.1997 (*B.L. Fisher*); Fianarantsoa, R.S. Ivohibe 8.0 km E Ivohibe, 22.48333°S, 46.96833°E, 1200 m, montane rainforest, 15.–21.X.1997 (*B.L. Fisher*); Fianarantsoa, 8.0 km NE Ivohibe, 22.4217°S, 46.8983°E, 1200 m, montane rainforest, 3.–9.XI.1997 (*B.L. Fisher*).

####### Diagnosis.

The following character combination clearly diagnoses *Tetramorium aspis* within the *Tetramorium cognatum* species complex: relatively large eyes (OI 25–27); frontal carinae weakly developed; propodeal spines short, triangular to elongate-triangular, and acute (PSLI 18–21), propodeal lobes always only weakly shorter than propodeal spines, and spines and lobes strongly inclined towards each other; petiolar node in profile high rounded nodiform, weakly squamiform and relatively thin, in profile around 2.0 to 2.2 times higher than long (LPeI 46–51), and in dorsal view around 1.4 to 1.6 times wider than long (DPeI 146–161); mesosoma with six or more pairs of long, standing hairs on pronotum and mesonotum, propodeum usually with one or two pairs located anteriorly.

####### Worker measurements

**(N=12).** HL 0.60–0.63 (0.61); HW 0.51–0.55 (0.54); SL 0.36–0.39 (0.37); EL 0.14–0.15 (0.14); PH 0.26–0.30 (0.28); PW 0.40–0.44 (0.43); WL 0.72–0.81 (0.77); PSL 0.11–0.13 (0.13); PTL 0.11–0.13 (0.12); PTH 0.23–0.25 (0.24); PTW 0.16–0.19 (0.18); PPL 0.16–0.19 (0.18); PPH 0.23–0.24 (0.24); PPW 0.22–0.24 (0.23); CI 85–88 (87); SI 68–72 (70); OI 25–27 (26); DMI 54–57 (55); LMI 35–37 (36); PSLI 18–21 (21); PeNI 40–44 (42); LPeI 46–51 (49); DPeI 146–161 (152); PpNI 51–56 (54); LPpI 70–81 (74); DPpI 126–135 (131); PPI 121–137 (129).

####### Worker description.

Head much longer than wide (CI 85–88); in full-face view posterior head margin weakly concave. Anterior clypeal margin with distinct median impression. Frontal carinae weakly developed, only faintly raised, slightly diverging posteriorly, merging with surrounding sculpture halfway between posterior eye margin and posterior head margin. Antennal scrobes weak to absent, shallow and without clear and distinct posterior and ventral margins. Antennal scapes very short, not reaching posterior head margin (SI 68–72). Eyes relatively large (OI 25–27). Mesosomal outline in profile flat to weakly convex, comparatively low and long (LMI 35–37), moderately marginate from lateral to dorsal mesosoma; promesonotal suture absent; metanotal groove usually present, but very weakly developed. Propodeal spines short, triangular to elongate-triangular, and acute (PSLI 18–21), propodeal lobes triangular to elongate-triangular, always slightly shorter than propodeal spines, in profile spines and lobes strongly inclined towards each other. Petiolar node in profile high rounded nodiform, weakly squamiform and relatively thin, around 2.0 to 2.2 times higher than long (LPeI 46–51), anterior and posterior faces approximately parallel, anterodorsal margin usually situated slightly higher and more angulate than posterodorsal margin, petiolar dorsum relatively flat to weakly convex; node in dorsal view around 1.5 to 1.6 times wider than long (DPeI 146–161), in dorsal view pronotum around 2.3 to 2.5 times wider than petiolar node (PeNI 40–44). Postpetiole in profile globular, between 1.2 to 1.4 times higher than long (LPpI 70–81); in dorsal view around 1.3 to 1.4 times wider than long (DPpI 126–135), pronotum around 1.8 to 2.0 times wider than postpetiole (PpNI 51–56). Postpetiole in profile appearing more voluminous than petiolar node, postpetiole in dorsal view around 1.2 to 1.4 times wider than petiolar node (PPI 121–137). Mandibles completely unsculptured, smooth, and shiny; clypeus weakly irregularly longitudinally rugulose, median ruga never fully developed, usually reduced to few traces, one or two mostly unbroken lateral rugulae present on each side; cephalic dorsum between frontal carinae longitudinally rugulose with seven to nine rugulae, rugulae usually running from posterior clypeal margin to posterior head margin, often irregularly shaped, interrupted or with cross-meshes; scrobal area mostly unsculptured; lateral head reticulate-rugose to longitudinally rugose, often with larger areas with reduced sculpture. Ground sculpture on head weakly to moderately reticulate-punctate, especially laterally. Dorsum and sides of mesosoma mostly irregularly longitudinally rugose/rugulose, sides of mesosoma often with some reticulate-rugose areas. Ground sculpture on dorsal mesosoma mostly absent, lateral mesosoma usually weakly to moderately reticulate-punctate. Forecoxae usually unsculptured, smooth and shining. Both waist segments and gaster fully unsculptured, smooth, and shining. Dorsum of head with several pairs of long, fine, standing hairs; mesosoma with six or more pairs on pronotum and mesonotum, propodeum usually with one or two pairs anteriorly; waist segments and first gastral tergite without any standing hairs at all; first gastral tergite with short, moderately dense, appressed pubescence. Anterior edges of antennal scapes and dorsal (outer) surfaces of hind tibiae with appressed hairs. Head, mesosoma, waist segments and gaster usually of uniform brown to dark brown, appendages yellowish to light brown; sometimes waist segments and gaster lighter than head and mesosoma but still darker than appendages.

####### Etymology.

The name of the new species is Old Greek and means “shield,” referring to the weakly squamiform condition of the petiolar node. The species epithet is a nominative noun, and thus invariant.

####### Distribution and biology.

So far *Tetramorium aspis* is known only from a few localities in the southeast of Madagascar around Ivohibe and Andringitra, where it was collected in rainforests or montane rainforests at elevations ranging from 785 to 1680 m (Fig. 63). *Tetramorium aspis* was mainly sampled from leaf litter or general collecting on the ground, which suggests that it might be a ground-active species.

####### Discussion.

The new species is clearly distinguishable within the *Tetramorium cognatum* species complex. *Tetramorium aspis* with its large eyes (OI 25–27) and presence of six or more pairs of long, standing hairs on the mesosoma is unlikely to be confused with the small-eyed *Tetramorium gladius* (OI 19–20), or *Tetramorium freya*, which lacks standing pilosity on the mesosomal dorsum. Furthermore, the weakly developed frontal carinae separate it from the species with strong frontal carinae *Tetramorium myrmidon*, *Tetramorium proximum*, and *Tetramorium tenuinode*. The latter three are also mostly larger species (HW 0.58–0.81; WL 0.76–1.07) compared to *Tetramorium aspis* (HW 0.51–0.55; WL 0.72–0.81). The remaining four species are morphologically closer to *Tetramorium aspis*. Of these, *Tetramorium camelliae* differs significantly from *Tetramorium aspis* on the basis of petiolar node development. In the latter the node is only weakly squamiform, in profile around 2.0 to 2.2 times higher than long (LPeI 46–51), and in dorsal view between 1.4 to 1.6 times wider than long (DPeI 146–161). This shape contrasts with the node of *Tetramorium camelliae*, which is around 2.8 to 3.0 times higher than long (LPeI 33–36) and around 2.3 to 2.4 times wider than long (DPeI 228–238). The best character for separating *Tetramorium aspis* from the remaining three species is the development and arrangement of propodeal spines and lobes. In *Tetramorium aspis* the spines are short, triangular to elongate-triangular, and acute (PSLI 18–21), the lobes relatively long, only weakly shorter than the spines, and the spines and lobes are strongly inclined towards each other. This arrangement is not seen in *Tetramorium cognatum*, *Tetramorium karthala* or *Tetramorium rumo* (it is present in *Tetramorium camelliae* though) since they either have short to moderate spines (*Tetramorium karthala* + *Tetramorium rumo*: PSLI 20–26), much longer than the lobes, or the spines are very short (*Tetramorium cognatum*: PSLI 12–16) and approximately same length as lobes or even shorter. Beyond this conspicuous character, *Tetramorium aspis* also has longer antennal scapes (SI 68–72), a broader petiolar node (DPeI 146–161), and a narrower postpetiole (DPpI 126–135) than *Tetramorium cognatum* (SI 61–67; DPeI 129–142; DPpI 137–153). *Tetramorium karthala*, which is only found on the Comoros, has only two to four pairs of long, standing hairs on the pronotum and mesonotum while *Tetramorium aspis* has least least six pairs. The last species, *Tetramorium rumo*, has very short antennal scapes (SI 60–66), a thinly cuneiform and moderately squamiform petiolar node, and is usually very bright yellow to light brown.

Intriguingly, *Tetramorium scutum* from the *Tetramorium sikorae* complex, which is endemic to the same geographic area, bears a strong superficial resemblance to *Tetramorium aspis*. However, they are in different species complexes based on their differences in pilosity on the first gastral tergite (present in *Tetramorium scutum* but absent in *Tetramorium aspis*). Apart from this key character they also differ slightly in eye size and propodeal spine length.

To our best knowledge, there is no apparent intraspecific variation in *Tetramorium aspis*.

###### Tetramorium camelliae

Hita Garcia & Fisher sp. n.

http://zoobank.org/E836FA0A-EA89-46D0-86A1-5518D9075C98

http://species-id.net/wiki/Tetramorium_camelliae

Figs 22A , 31 , 63

####### Type material.

**Holotype**, pinned worker, MADAGASCAR, Fianarantsoa, Ranomafana National Park, Vohiparara, 21.24444°S, 47.39694°E, 1173 m, montane forest, 16.IX.1992 (*E. Rajeriarison*) (CAS: CASENT0247496). **Paratypes**, four pinned workers with same data as holotype (BMNH: CASENT0247495; CAS: CASENT0247499; MCZ: CASENT0247498, CASENT0247500).

**Figure 31.**
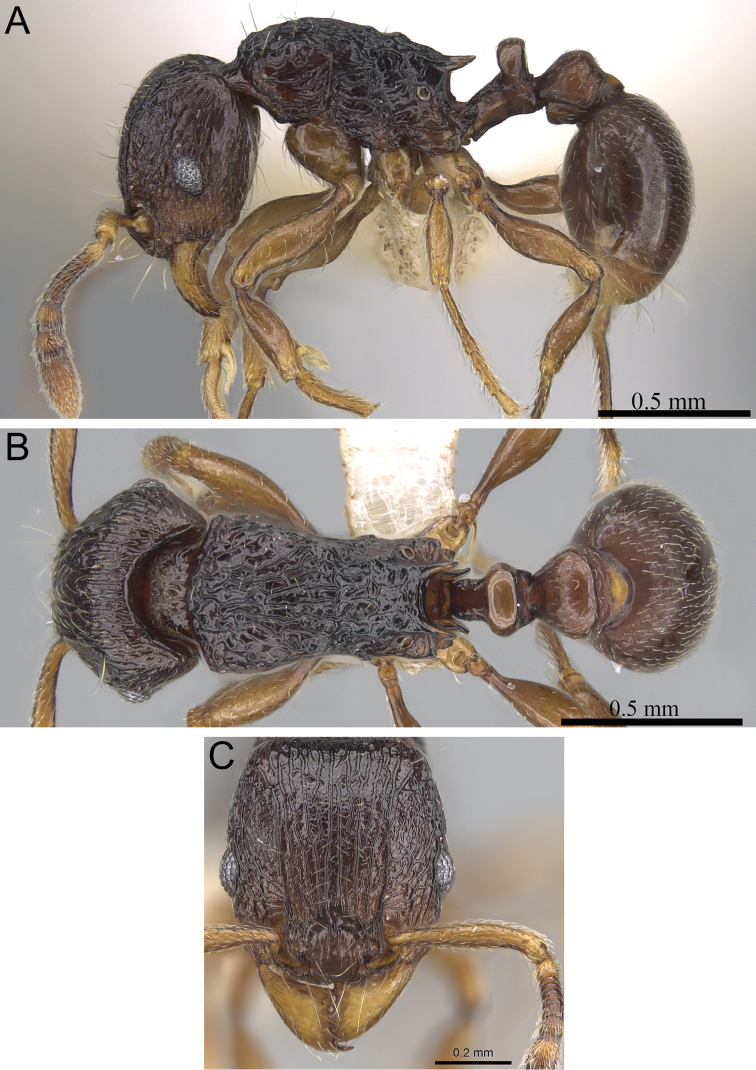
*Tetramorium camelliae* holotype worker (CASENT0247496). **A** Body in profile **B** Body in dorsal view **C** Head in full-face view.

####### Non-type material.

MADAGASCAR: Fianarantsoa, Ranomafana National Park, Vohiparara, 21.24444°S, 47.39694°E, 1173 m, montane forest, 16.IX.1992 (*E. Rajeriarison*); Fianarantsoa, Ranomafana National Park, Vatoharanana River, 4.1 km 231° SW Ranomafana, 21.29°S, 47.43333°E, 1100 m, montane rainforest, 27.–31.III.2003 (*B.L. Fisher et al.*).

####### Diagnosis.

The strongly squamiform and transverse petiolar node, which in profile is around 2.7 to 3.0 times higher than long (LPeI 33–36), and in dorsal view around 2.3 to 2.4 times wider than long (DPeI 228–238), isolates *Tetramorium camelliae* from the other members of the *Tetramorium cognatum* species complex.

####### Worker measurements

**(N=7).** HL 0.56–0.63 (0.61); HW 0.50–0.57 (0.55); SL 0.33–0.40 (0.38); EL 0.13–0.15 (0.14); PH 0.25–0.29 (0.27); PW 0.40–0.44 (0.43); WL 0.67–0.78 (0.74); PSL 0.10–0.12 (0.10); PTL 0.07–0.09 (0.08); PTH 0.21–0.25 (0.24); PTW 0.16–0.21 (0.19); PPL 0.15–0.20 (0.18); PPH 0.21–0.25 (0.24); PPW 0.20–0.26 (0.24); CI 89–91 (91); SI 66–70 (68); OI 25–26 (26); DMI 56–60 (57); LMI 36–37 (37); PSLI 17–19 (18); PeNI 40–48 (45); LPeI 33–36 (35); DPeI 228–238 (234); PpNI 50–59 (57); LPpI 69–80 (74); DPpI 133–141 (137); PPI 120–134 (126).

####### Worker description.

Head much longer than wide (CI 89–91); in full-face view posterior head margin weakly concave. Anterior clypeal margin with distinct median impression. Frontal carinae moderately developed, diverging posteriorly, merging with surrounding sculpture halfway between posterior eye margin and posterior head margin. Antennal scrobes weak, shallow and without clear and distinct posterior and ventral margins. Antennal scapes very short, not reaching posterior head margin (SI 66–70). Eyes relatively large (OI 25–26). Mesosomal outline in profile flat to weakly convex, comparatively low and long (LMI 36–37), moderately marginate from lateral to dorsal mesosoma; promesonotal suture absent; metanotal groove present, but weakly developed. Propodeal spines short, triangular to elongate-triangular, and acute (PSLI 17–19), propodeal lobes triangular to elongate-triangular and of approximately same length as propodeal spines, in profile spines and lobes strongly inclined towards each other. Petiolar node in profile strongly squamiform, around 2.7 to 3.0 times higher than long (LPeI 33–36), anterior and posterior faces approximately parallel, anterodorsal and posterodorsal margins situated at about same height, petiolar dorsum relatively flat to weakly convex; node in dorsal view transverse and 2.3 to 2.4 times wider than long (DPeI 228–238), in dorsal view pronotum between 2.1 to 2.5 times wider than petiolar node (PeNI 40–48). Postpetiole in profile subglobular, between 1.2 to 1.4 times higher than long (LPpI 69–80); in dorsal view around 1.3 to 1.4 times wider than long (DPpI 133–141), pronotum between 1.7 to 2.0 times wider than postpetiole (PpNI 50–59). Postpetiole in profile much more voluminous than petiolar node, postpetiole in dorsal view around 1.2 to 1.4 times wider than petiolar node (PPI 120–134). Mandibles completely unsculptured, smooth, and shiny; clypeus irregularly longitudinally rugulose, median ruga never fully developed, usually reduced to few traces posteriorly or medially, one or two mostly unbroken lateral rugulae present on each side; cephalic dorsum between frontal carinae longitudinally rugose/rugulose with seven to eight rugae/rugulae, rugae usually running unbroken from posterior clypeal margin to posterior head margin, sometimes interrupted or with cross-meshes; scrobal area partly unsculptured, but mostly merging with surrounding reticulate-rugose to longitudinally rugose sculpture present on lateral head. Dorsum and sides of mesosoma longitudinally rugose/rugulose. Forecoxae unsculptured, smooth and shining. Ground sculpture on head and mesosoma weak to absent. Both waist segments and gaster fully unsculptured, smooth, and shining. Dorsum of head with several pairs of long, fine, standing hairs; mesosoma with seven to eight pairs restricted to pronotum and mesonotum; propodeum, waist segments and first gastral tergite without any standing hairs; first gastral tergite with short, moderately dense, appressed pubescence. Anterior edges of antennal scapes and dorsal (outer) surfaces of hind tibiae with appressed hairs. Head, mesosoma, waist segments, and gaster uniform brown to dark brown, appendages yellowish to light brown.

####### Etymology.

The new species is dedicated to the first author’s lovely life partner, Tracy Lynn Camellia Audisio from San Francisco, California, U.S.A.

####### Distribution and biology.

*Tetramorium camelliae* is a rare species only known from Ranomafana National Park (Fig. 63) where it was collected twice in montane rainforest at elevations ranging from 1100 to 1173. The little available data indicates that *Tetramorium camelliae* lives in leaf litter.

####### Discussion.

*Tetramorium camelliae* is easily distinguishable from the other members of the *Tetramorium cognatum* species complex. The main diagnostic character is the petiolar node shape, which is strongly squamiform in *Tetramorium camelliae* (LPeI 33–36; DPeI 228–238) but not in the remainder of the species complex (LPeI 37–62; DPeI 111–171). Generally, its smaller body size, weaker frontal carinae, and gastral pubescence ally *Tetramorium camelliae* morphologically with *Tetramorium aspis*, *Tetramorium cognatum*, *Tetramorium karthala* and *Tetramorium rumo*.

###### Tetramorium cognatum

Bolton, 1979

http://species-id.net/wiki/Tetramorium_cognatum

**Figure 32.**
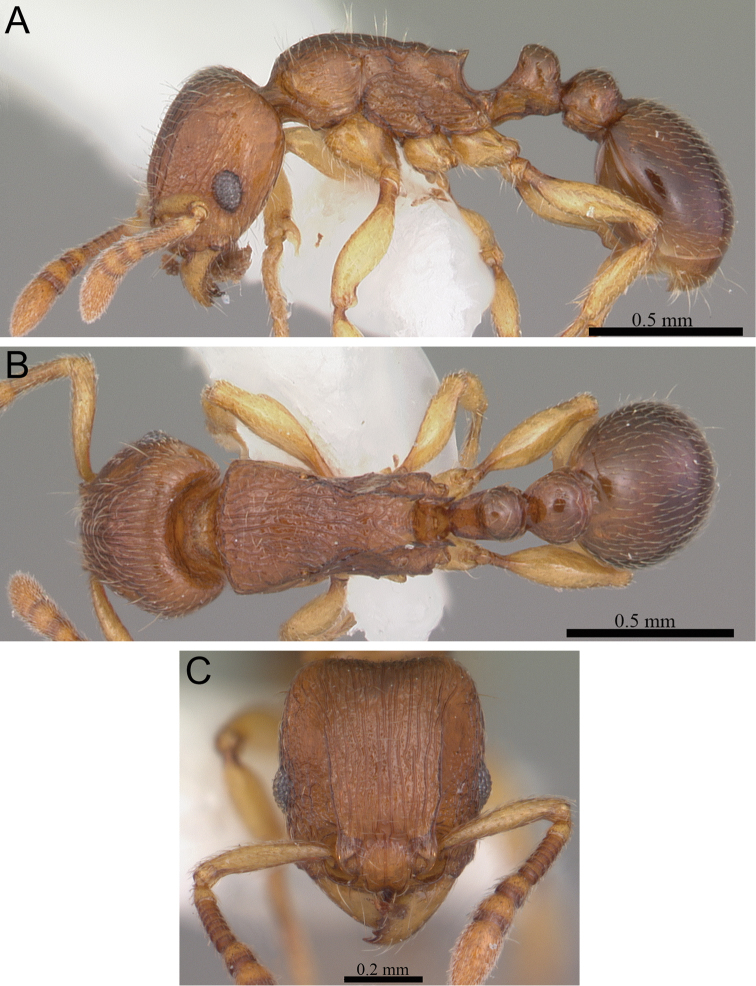
*Tetramorium cognatum* paratype worker (CASENT0102343). **A** Body in profile **B** Body in dorsal view **C** Head in full-face view.

Figs 21B , 23B , 27C , 28A, B , 32 , 63

Tetramorium cognatum Bolton, 1979: 135.

####### Type material.

**Holotype**, pinned worker, MADAGASCAR, Toamasina, Perinet & vicinity, 18.82667°S, 48.44778°E, rainforest, rotten wood, 19.III.1969 (*W.L. Brown*) (MCZ: MCZ_Holotype_32261) [examined]. **Paratypes**, eleven pinned workers with same data as holotype except collected from 17.–19.III.1969 (BMNH: CASENT0102343, CASENT0102344, CASENT0235218; MCZ: MCZ_Paratype_32261; NHMB; MHNG: CASENT0911248) [all examined, except NHMB].

[Note: the GPS data of the type locality was not provided either by the locality label or the original description. The data presented above is based on our own geo-referencing of the Périnet=Andasibe area and should be considered an approximation and not the exact position of the type locality.]

####### Non-Type material.

MADAGASCAR: no loc (*Staudinger*); Antananarivo, Réserve Spéciale d’Ambohitantely, Forêt d’Ambohitantely, 20.9 km 72° NE Ankazobe, 18.22528°S, 47.28683°E, 1410 m, montane rainforest, 17.–21.IV.2001 (*B.L. Fisher et al.*); Antananarivo, Réserve Speciale d’Ambohitantely, 18.18762°S, 47.28576°E, 1580 m, montane forest, 8.III.2012 (*B.L. Fisher et al.*); Antananarivo, Réserve Speciale d’Ambohitantely, 18.22444°S, 47.2774°E, 1490 m, montane forest, 9.III.2012 (*B.L. Fisher et al.*); Antananarivo, 3 km 41° NE Andranomay, 11.5 km 147° SSE Anjozorobe, 18.47333°S, 47.96°E, 1300 m, montane rainforest, 5.–13.XII.2000 (*B.L. Fisher et al.*); Antananarivo, Ankalalahana, 19.00711°S, 47.1337°E, 1350 m, *Uapaca* woodland, 26.III.2011 (*B.L. Fisher et al.*); Antananarivo, Mandraka Park, 18.9019°S, 47.90786°E, 1360 m, montane shrubland, 11.III.2012 (*B.L. Fisher et al.*); Antananarivo, Réserve Naturelle Sohisika, Sohisika 24.6 km NNE Ankazobe, 18.10322°S, 47.18692°E, 1464 m, gallery montane forest, 1.–2.VI.2008 (*B.L. Fisher et al.*); Antananarivo, Forêt de galerie, Telomirahavavy, 23.4 km NNE Ankazobe, 18.12167°S, 47.20627°E, 1520 m, disturbed gallery montane forest, 3.–4.VI.2008 (*B.L. Fisher et al.*); Antsiranana, Ambondrobe, 41.1 km 175° Vohemar, 13.71533°S, 50.10167°E, 10 m, littoral rainforest, 30.XI.2004 (*B.L. Fisher*); Antsiranana, Parc National Mont. d’Ambre, 1050 m, 12.II.1977 (*W.L. & D.E. Brown*); Antsiranana, Parc National Montagne d’Ambre, 3.6 km 235° SW Joffreville, 12.53444°S, 49.1795°E, 925 m, montane rainforest, 20.–26.I.2001 (*B.L. Fisher et al.*); Antsiranana, Parc National Montagne d’Ambre, 12.2 km 211° SSW Joffreville, 12.59639°S, 49.1595°E, 1300 m, montane rainforest, 2.–7.II.2001 (*B.L. Fisher et al.*); Antsiranana, Parc National Montagne d’Ambre, 12.2 km 211° SSW Joffreville, 12.59639°S, 49.1595°E, 1300 m, montane rainforest, 7.II.2001 (*G.D. Alpert*); Antsiranana, Parc National Montagne d’Ambre, 12.51778°S, 49.17957°E, 1000 m, montane rainforest, 3.–7.III.2001 (*B.L. Fisher et al.*); Antsiranana, Parc National Montagne d’Ambre, 12.52861°S, 49.17717°E, 1100 m, montane rainforest, 12.III.2001 (*B.L. Fisher et al.*); Antsiranana, Parc National Montagne d’Ambre, Ambre grand lac, 12.59656°S, 49.15932°E, 1350 m, montane rainforest, 13.XI.2007 (*B.L. Fisher et al.*); Antsiranana, Parc National Montagne d’Ambre, Lac maudit, 12.58502°S, 49.15147°E, 1250 m, montane rainforest, 13.–14.XI.2007 (*B.L. Fisher et al.*); Antsiranana, Parc National Montagne d’Ambre, Roussettes, 12.52574°S, 49.17238°E, 1025 m, montane rainforest, 15.XI.2007 (*B.L. Fisher et al.*); Antsiranana, Parc National Montagne d’Ambre, Petit lac, 12.53664°S, 49.17412°E, 1130 m, montane rainforest, 17.XI.2007 (*B.L. Fisher et al.*); Antsiranana, Parc National Montagne d’Ambre, Antomboka, 12.51269°S, 49.17807°E, 970 m, montane rainforest, 17.XI.2007 (*B.L. Fisher et al.*); Antsiranana, Parc National Montagne d’Ambre, Mahasarika, 12.53176°S, 49.17662°E, 1135 m, montane rainforest, 19.XI.2007 (*B.L. Fisher et al.*); Antsiranana, Parc National Montagne d’Ambre, Pic Bades, 12.5186°S, 49.18625°E, 900 m, montane rainforest, 20.XI.2007 (*B.L. Fisher et al.*); Antsiranana, Rés. Anjanaharibe-Sud, 17 km W Andapa, 15°45'27.9"S, 49°30'36.7"E, 875 m, rainforest, 5.XI.1994 (*G.D. Alpert*); Antsiranana, Rés. Anjanaharibe-Sud, 6.5 km SSW Befingotra, 14.75°S, 49.5°E, 875 m, rainforest, 17.–31.X.1994 (*B.L. Fisher*); Antsiranana, Rés. Anjanaharibe-Sud, 9.2 km WSW Befingotra, 14.75°S, 49.46667°E, 1180-1200 m, montane rainforest, 7.–9.XI.1994 (*B.L. Fisher*); Antsiranana, Anjanaharibe, 1.II.-1.III.2003 (*K.A. Jackson & D. Carpenter*); Antsiranana, Betaolana Forest, along Bekona River, 14.52996°S, 49.44039°E, 880 m, rainforest, 4.–5.III.2009 (*B.L. Fisher et al.*); Antsiranana, Forêt de Binara, 9.4 km 235° SW Daraina, 13.26333°S, 49.6°E, 1100 m, montane rainforest, 5.XII.2003 (*B.L. Fisher*); Antsiranana, R.S. Manongarivo, 10.8 km 229° SW Antanambao, 13.96167°S, 48.43333°E, 400 m, rainforest, 8.–13.X.1998 (*B.L. Fisher*); Antsiranana, R.S. Manongarivo, 12.8 km 228° SW Antanambao, 13.97667°S, 48.42333°E, 780 m, rainforest, 11-17.X.1998 (*B.L. Fisher*); Antsiranana, R.S. Manongarivo, 14.5 km 220° SW Antanambao, 13.99833°S, 48.42833°E, 1175 m, montane rainforest, 20.X.1998 (*B.L. Fisher*); Antsiranana, R.N.I. Marojejy, 14°26'43.2"S, 49°47'8.3"E, 375 m, 23.XI.1993 (*G.D. Alpert*); Antsiranana, Parc National de Marojejy, Manantenina River, 27.6 km 35° NE Andapa, 9.6 km 327° NNW Manantenina, 14.435°S, 49.76°E, 775 m, rainforest, 15.XI.-11.XII.2005 (*B.L. Fisher et al.*); Antsiranana, Parc National de Marojejy, Antranohofa, 26.6 km 31° NNE Andapa, 10.7 km 318° NW Manantenina, 14.44333°S, 49.74333°E, 1325 m, montane rainforest, 19.XI.2003 (*B.L. Fisher*); Antsiranana, Sakaramy, 12.44114°S, 49.23197°E, 260 m, tropical dry forest, 11.V.2011 (*B.L. Fisher et al.*); Fianarantsoa, Rés. Andringitra 40 km S Ambalavao, 22.21667°S, 46.96667°E, 1225 m, montane rainforest, 19.X.1993 (*B.L. Fisher*); Fianarantsoa, Rés. Andringitra 44 km S Ambalavao, 22.23333°S, 47°E, 800 m, rainforest, 11.X.1993 (*B.L. Fisher*); Fianarantsoa, Ampandravelo II Non Protected Area, 10.78 km NE Ranohira, 22.53917°S, 45.51548°E, 873 m, shrubland, 20.–22.II.2010 (*A. Ravelomanana*); Fianarantsoa, Parc National Befotaka-Midongy, Papango 27.7 km S Midongy-Sud, Mount Papango, 23.83517°S, 46.96367°E, 940 m, rainforest, 14.–16.XI.2006 (*B.L. Fisher et al.*); Fianarantsoa, Parc National Befotaka-Midongy, Papango 28.5 km S Midongy-Sud, Mount Papango, 23.84083°S, 46.9575°E, 1250 m, montane rainforest, 17.–18.XI.2006 (*B.L. Fisher et al.*); Fianarantsoa, Isalo National Park, Canyon de Sinze, 22°28’ S, 45°33’ E, 800 m, forest, 17.II.1993 (*E. Rajeriarison*); Fianarantsoa, Isalo IV National Parc, 12 km SW Ranohira, 22.61472°S, 45.31304°E, 867 m, *Bismarckia* woodland, 25.II.2010 (*A. Ravelomanana*); Fianarantsoa, R.S. Ivohibe, 7.5 km ENE Ivohibe, 22.47°S, 46.96°E, 900 m, rainforest, 7.–12.X.1997 (*B.L. Fisher*); Fianarantsoa, R.S. Ivohibe 8.0 km E Ivohibe, 22.48333°S, 46.96833°E, 1200 m, montane rainforest, 15.–21.X.1997 (*B.L. Fisher*); Fianarantsoa, 9.0 km NE Ivohibe, 22.42667°S, 46.93833°E, 900 m, rainforest, 12.–17.X.1997 (*B.L. Fisher*); Fianarantsoa, Manandriana I Non Protected Area, 27.11 km SW Ambositra, 20.73194°S, 47.09413°E, 1590 m, savannah grassland, 9.–11.II.2010 (*A. Ravelomanana*); Fianarantsoa, Parc National de Ranomafana, Vatoharanana River, 4.1 km 231° SW Ranomafana, 21.29°S, 47.43333°E, 1100 m, montane rainforest, 27.–31.2003 (*B.L. Fisher et al.*); Fianarantsoa, Parc National Ranomafana, Talatakely, 21.24833°S, 47.42667°E, in guava forest, 9.–26.IV.1998 (*C.E. Griswold et al.*); Fianarantsoa, Parc National de Ranomafana, Sahamalaotra River, 6.6 km 310° NW Ranomafana, 21.23667°S, 47.39667°E, 1150 m, montane rainforest, 31.III.2003 (*B.L. Fisher et al.*); Fianarantsoa, Forêt Classée Vatovavy, 7.6 km 122° Kianjavato, 21.4°S, 47.94°E, 175 m, rainforest, 6.–8.VI.2005 (*B.L. Fisher et al.*); Fianarantsoa, Forêt de Vevembe, 66.6 km 293° Farafangana, 22.791°S, 47.18183°E, 600 m, rainforest, transition to montane forest, 23.IV.2006 (*B.L. Fisher et al.*); Mahajanga, Réserve Spéciale Marotandrano, Marotandrano 48.3 km S Mandritsara, 16.28322°S, 48.81443°E, 865 m, transition humid forest, 7.XII.2007 (*B.L. Fisher et al.*); Toamasina, Montagne d’Akirindro 7.6 km 341° NNW Ambinanitelo, 15.28833°S, 49.54833°E, 600 m, rainforest, 17.–21.III.2003 (*B.L. Fisher et al.*); Toamasina, Ambanizana, Parc National Masoala, 15.57167°S, 50.00611°E, 848 m, montane rainforest, 26.II.-2.III.2003 (*D. Andriamalala et al.*); Toamasina, 5.3 km SSE Ambanizana, Andranobe, 15.67133°S, 49.97395°E, 425 m, rainforest, 21.XI.1993 (*B.L. Fisher*); Toamasina, 6.3 km S Ambanizana, Andranobe, 15.6813°S, 49.958°E, 25 m, rainforest, 23.XI.1993 (*B.L. Fisher*); Toamasina, 6.9 km NE Ambanizana, Ambohitsitondroina, 15.58506°S, 50.00952°E, 825 m, rainforest, 2.–8.XII.1993 (*B.L. Fisher*); Toamasina, Réserve Spéciale Ambatovaky, Sandrangato river, 16.7633°S, 49.26692°E, 520 m, rainforest, 22.–24.II.2010 (*B.L. Fisher et al.*); Toamasina, Forêt Ambatovy, 14.3 km 57° Moramanga, 18.85083°S, 48.32°E, 1075 m, montane rainforest, 21.III.2004-12.IV.2005 (*B.L. Fisher et al.*); Toamasina, Ambatovy, 12.4 km NE Moramanga, 18.84963°S, 48.2947°E, 1010 m, montane rainforest, 3.–6.III.2007 (*B.L. Fisher et al.*); Toamasina, Ambatovy, 12.4 km NE Moramanga, 18.83937°S, 48.30842°E, 1080 m, montane rainforest, 4.–7.III.2007 (*B.L. Fisher et al.*); Toamasina, Station Forestière Analamazaotra, Analamazaotra 1.3 km S Andasibe, 18.38466°S, 48.41271°E, 980 m, montane rainforest, 11.–13.XII.2007 (*B.L. Fisher et al.*); Toamasina, Analamay, 18.80623°S, 48.33707°E, 1068 m, montane rainforest, 21.III.2004 (*Malagasy ant team*); Toamasina, F.C. Andriantantely, 18.695°S, 48.81333°E, 530 m, rainforest, 4.–10.XII.1998 (*H.J. Ratsirarson*); Toamasina, Montagne d’Anjanaharibe, 18.0 km 21° NNE Ambinanitelo, 15.18833°S, 49.615°E, 470 m, rainforest, 8.–12.III.2003 (*B.L. Fisher et al.*); Toamasina, Montagne d’Anjanaharibe, 19.5 km 27° NNE Ambinanitelo, 15.17833°S, 49.635°E, 1100 m, montane rainforest, 12.–16.III.2003 (*B.L. Fisher et al.*); Toamasina, Reserve Betampona, Camp Vohitsivalana, 37.1 km 338° Toamasina, 17.88667°S, 49.2025°E, 520 m, rainforest, 1.–3.XII.2005 (*B.L. Fisher et al.*); Toamasina, Reserve Betampona, Camp Rendrirendry, 34.1 km 332° Toamasina, 17.924°S, 49.19967°E, 390 m, rainforest, 28.XI.2005 (*B.L. Fisher et al.*); Toamasina, Bevolota, 17.1 km N Andasibe, 18.77071°S, 48.43164°E, 995 m, montane rainforest, 12.XII.2007 (*B.L. Fisher et al.*); Toamasina, F.C. Didy, 18.19833°S, 48.57833°E, 960 m, rainforest, 16.–23.XII.1998 (*H.J. Ratsirarson*); Toamasina, P.N. Mantadia, 18.79167°S, 48.42667°E, 895 m, rainforest, 25.XI.-1.XII.1998 (*H.J. Ratsirarson*); Toamasina, Sahafina Forest, 11.4 km W Brickaville, 18.81445°S, 48.96205°E, 140 m, rainforest, 13.–14.XII.2007 (*B.L. Fisher et al.*); Toamasina, F.C. Sandranantitra, 18.04833°S, 49.09167°E, 450 m, rainforest, 18.–21.I.1999 (*H.J. Ratsirarson*); Toamasina, Torotorofotsy, 18.87082°S, 48.34737°E, 1070 m, montane rainforest, marsh edge, 24.–29.III.2004 (*Malagasy ant team*); Toamasina, Parc National de Zahamena, Onibe River, 17.75908°S, 48.85468°E, 780 m, rainforest, 21.–23.II.2009 (*B.L. Fisher et al.*); Toamasina, Parc National de Zahamena, Tetezambatana forest, near junction of Nosivola and Manakambahiny Rivers, 17.74298°S, 48.72936°E, 860 m, rainforest, 18.–19.II.2009 (*B.L. Fisher et al.*); Toliara, Réserve Spéciale d’Ambohijanahary, Forêt d’Ankazotsihitafototra, 35.2 km 312° NW Ambaravaranala, 18.26667°S, 45.40667°E, 1050 m, montane rainforest, 13.–17.I.2003 (*B.L. Fisher et al.*); Toliara, Réserve Spéciale d’Ambohijanahary, Forêt d’Ankazotsihitafototra, 34.6 km 314° NW Ambaravaranala, 18.26°S, 45.41833°E, 1100 m, montane rainforest, 16.I.2003 (*B.L. Fisher et al.*); Toliara, Rés. Andohahela, 11 km NW Enakara, 24.56667°S, 46.83333°E, 800 m, rainforest, 17.XI.1992 (*B.L. Fisher*); Toliara, Rés. Andohahela, 10 km NW Enakara, 24.56667°S, 46.81667°E, 420 m, rainforest, 19.XI.1992 (*B.L. Fisher*); Toliara, Rés. Andohahela, 10 km NW Enakara, 24.56667°S, 46.81667°E, 430 m, rainforest, 25.XI.1992 (*B.L. Fisher*); Toliara, Rés. Andohahela, 13 km NW Enakara, 24.55°S, 46.8°E, 1250 m, montane rainforest, 30.XI.1992 (*B.L. Fisher*); Toliara, Rés. Andohahela, 13 km NW Enakara, 24.55°S, 46.8°E, 1300 m, montane rainforest, 2.XII.1992 (*B.L. Fisher*); Toliara, Rés. Andohahela, 13 km NW Enakara, 24.56667°S, 46.81667°E, 900 m, rainforest, 3.XII.1992 (*B.L. Fisher*); Toliara, Rés. Andohahela, 3 km E Mahamavo, 24°45’ S, 46°45’ E, 1050 m, montane rainforest, 5.XI.1993 (*P.S. Ward*); Toliara, Parc National d’Andohahela, Col du Sedro, 3.8 km 113° ESE Mahamavo, 37.6 km 341° NNW Tolagnaro, 24.76389°S, 46.75167°E, 900 m, montane rainforest, 21.–25.I.2002 (*B.L. Fisher et al.*); Toliara, Parc National d’Andohahela, Manampanihy River, 5.4 km 113° ESE Mahamavo, 36.7 km 343° NNW Tolagnaro, 24.76389°S, 46.76683°E, 650 m, rainforest, 24.I.2002 (*B.L. Fisher et al.*); Toliara, Forêt Ivohibe 55.6 km N Tolagnaro, 24.56167°S, 47.20017°E, 650 m, rainforest, 4.XII.2006 (*B.L. Fisher et al.*); Toliara, Réserve Spéciale Kalambatritra, Befarara, 23.4178°S, 46.4478°E, 1390 m, montane rainforest, 7.–8.II.2009 (*B.L. Fisher et al.*); Toliara, Réserve Spéciale Kalambatritra, Betanana, 23.4144°S, 46.459°E, 1360 m, montane rainforest, 8.II.2009 (*B.L. Fisher et al.*); Toliara, Réserve Spéciale Kalambatritra, Ampanihy, 23.4635°S, 46.4631°E, 1270 m, montane rainforest, 9.–10.II.2009 (*B.L. Fisher et al.*); Toliara, Réserve Spéciale Kalambatritra, Ampanihy, 23.463°S, 46.47057°E, 1269 m, montane rainforest, 10.II.2009 (*B.L. Fisher et al.*); Toliara, Réserve Spéciale Kalambatritra, Ambinanitelo, 23.4502°S, 46.45658°E, 1325 m, montane rainforest, 11.II.2009 (*B.L. Fisher et al.*); Toliara, Grand Lavasoa, 25.9 km W Tolagnaro, 25.08767°S, 46.749°E, 450 m, rainforest, 30.XI.-2.XII.2006 (*B.L. Fisher et al.*); Toliara, 4.4 km 148° SSE Lavanono, 25.45056°S, 44.97417°E, 60 m, spiny forest/thicket, 17.II.2002 (*B.L. Fisher et al.*); Toliara, 2.7 km WNW 302° Ste. Luce, 24.77167°S, 47.17167°E, 20 m, littoral rainforest, 9.–11.XII.1998 (*B.L. Fisher*); Toliara, Manatantely, 8.9 km NW Tolagnaro, 24.9815°S, 46.92567°E, 100 m, rainforest, 27.XI.2006 (*B.L. Fisher et al.*).

####### Diagnosis.

*Tetramorium cognatum* can be easily separated from the other members of the *Tetramorium cognatum* species group by the following character combination: eyes very large (OI 27–29); antennal scapes very short (SI 61–67); frontal carinae weakly developed; propodeal spines reduced to very short, triangular teeth (PSLI 12–16), spines and lobes usually of approximately same length, often spines weakly shorter than lobes, very rarely spines weakly longer than lobes, never strongly directed towards each other; petiolar node high nodiform, in profile around 1.8 to 2.0 times higher than long (LPeI 49–55), in dorsal view around 1.3 to 1.4 times wider than long (DPeI 129–142); dorsal mesosoma normally with four to six (sometimes reduced to two to three) pairs of long, standing hairs occurring from anterior pronotum to metanotal groove, but absent from propodeum.

####### Worker measurements

**(N=25).** HL 0.53–0.61 (0.57); HW 0.48–0.54 (0.51); SL 0.31–0.36 (0.33); EL 0.13–0.15 (0.14); PH 0.25–0.29 (0.27); PW 0.37–0.42 (0.39); WL 0.65–0.75 (0.70); PSL 0.07–0.10 (0.08); PTL 0.11–0.13 (0.12); PTH 0.21–0.25 (0.23); PTW 0.15–0.17 (0.16); PPL 0.14–0.16 (0.15); PPH 0.20–0.23 (0.21); PPW 0.20–0.23 (0.22); CI 87–91 (90); SI 61–67 (65); OI 27–29 (28); DMI 54–59 (56); LMI 36–40 (38); PSLI 12–16 (14); PeNI 38–44 (41); LPeI 49–55 (52); DPeI 129–142 (135); PpNI 53–59 (56); LPpI 66–75 (70); DPpI 137–153 (145); PPI 131–147 (137).

####### Worker description.

Head much longer than wide (CI 87–91); in full-face view posterior head margin usually very weakly concave. Anterior clypeal margin with distinct median impression. Frontal carinae weakly developed, only faintly raised, slightly diverging posteriorly, and relatively long, often ending shortly before posterior head margin. Antennal scrobes very weakly developed, shallow and without clear and distinct posterior and ventral margins. Antennal scapes very short, not reaching posterior head margin (SI 61–67). Eyes very large (OI 27–29). Mesosomal outline in profile flat to weakly convex, comparatively low and long (LMI 36–40), moderately marginate from lateral to dorsal mesosoma; promesonotal suture absent; metanotal groove usually present, but relatively weak. Propodeal spines reduced to very short, triangular teeth (PSLI 12–16), propodeal lobes short and triangular, spines and lobes usually of approximately same length, often spines weakly shorter than lobes, very rarely spines weakly longer than lobes. Petiolar node in profile rounded high nodiform, around 1.8 to 2.0 times higher than long (LPeI 49–55), anterior and posterior faces approximately parallel, anterodorsal margin usually sharper than more rounded posterodorsal margin, both margins often situated at about same height, often anterodorsal margin slightly higher, petiolar dorsum usually weakly convex; node in dorsal view around 1.3 to 2 times wider than long (DPeI 129–142), in dorsal view pronotum between 2.3 to 2.7 times wider than petiolar node (PeNI 38–44). Postpetiole in profile globular, rarely subglobular, between 1.3 to 1.5 times higher than long (LPpI 66–75); in dorsal view around 1.3 to 1.5 times wider than long (DPpI 137–153), pronotum between 1.7 to 1.9 times wider than postpetiole (PpNI 53–59). Postpetiole in profile usually appearing shorter and less voluminous than petiolar node, postpetiole in dorsal view between 1.3 to 1.5 times wider than petiolar node (PPI 131–147). Mandibles completely unsculptured, smooth, and shiny; clypeus usually irregularly longitudinally rugulose, median ruga often unbroken and well developed, often partly reduced, and often fully absent; lateral rugulae ranging from one to three on each side, often irregularly shaped, broken, or reduced to traces; cephalic dorsum between frontal carinae longitudinally rugulose with six to ten fine rugulae, rugulae usually running from posterior clypeal margin to posterior head margin, mostly irregularly shaped, interrupted, meandering or with cross-meshes, and sometimes becoming much weaker posteriorly; scrobal area mostly unsculptured, merging with surrounding sculpture; lateral head reticulate-rugose to longitudinally rugose, often with larger areas of reduced sculpture; ground sculpture on head weakly to moderately punctate, sometimes completely absent. Dorsum of mesosoma irregularly longitudinally rugulose, sometimes weakly so; lateral mesosoma usually mostly irregularly longitudinally rugulose, lateral pronotum often much weaker-sculptured, almost smooth, and sometimes reticulate-rugulose; ground sculpture on mesosoma usually weakly to moderately punctate, sometimes completely absent. Forecoxae usually unsculptured, smooth and shining, sometimes with traces of ground sculpture dorsally. Both waist segments and gaster fully unsculptured, smooth, and shining. Dorsum of head with several pairs of long, standing hairs, dorsal mesosoma normally with four to six (sometimes reduced to two to three) pairs occurring from anterior pronotum to metanotal groove, but absent from propodeum; waist segments and first gastral tergite without any long, standing hairs at all; first gastral tergite with moderately long, dense, appressed to decumbent pubescence. Anterior edges of antennal scapes and dorsal (outer) surfaces of hind tibiae with appressed to subdecumbent hairs. Body colouration variable, ranging from uniformly bright yellow to very dark brown, if colour brown to dark brown, appendages usually lighter.

####### Distribution and biology.

This species is one of the most common *Tetramorium* encountered in the rainforests and montane rainforests of Madagascar (Fig. 63). Its distribution range encompasses almost all sampled forests in eastern and northern Madagascar, as well as the isolated humid forests in central and western parts of the island (e.g. Ambohijanahary, Isalo). Surprisingly, *Tetramorium cognatum* also seems to do fairly well outside humid forests since it is also found in a few disturbed gallery forests, in *Uapaca* woodland and tropical dry forests, and very rarely in spiny forest/thicket and savannah/grassland. Additional sampling in arid habitats might show that *Tetramorium cognatum* is even more widely distributed than currently understood. The species was mostly collected from leaf litter and the ground.

####### Discussion.

*Tetramorium cognatum* is an important species within this species complex. As outlined above, it is very widely distributed and common, and co-occurs in sympatry with almost all other members of the complex. It is easily distinguishable from the usually larger *Tetramorium freya*, which also lacks pilosity on the dorsal mesosoma, and *Tetramorium gladius*, the species with the smallest eyes in the complex (OI 19–20). Furthermore, *Tetramorium cognatum* possesses relatively weakly developed frontal carinae, separating it from *Tetramorium myrmidon*, *Tetramorium proximum*, and *Tetramorium tenuinode*. This character allies *Tetramorium cognatum* with *Tetramorium aspis*, *Tetramorium camelliae*, *Tetramorium karthala*, and *Tetramorium rumo*, to which it is generally closer in morphology. Of these species close to *Tetramorium cognatum*, *Tetramorium karthala* (endemic to Grand Comore) has longer antennal scapes (SI 70–74) and propodeal spines (PSLI 20–22) than *Tetramorium cognatum* (SI 61–67; PSLI 12–16). *Tetramorium camelliae*, only found in Ranomafana, and *Tetramorium rumo* have squamiform or thinly cuneiform petiolar nodes, which in profile are between 2.3 to 3.0 times higher than long (LPeI 33–43) while the node of *Tetramorium cognatum* is only 1.8 to 2.0 times higher than long (LPeI 49–55). *Tetramorium aspis*, which is only found in the area around Andringitra and Ivohibe, has longer propodeal spines (PSLI 18–21) than *Tetramorium cognatum*. More importantly however, the propodeal spines and lobes of *Tetramorium cognatum* are not strongly inclined towards each other as they are in *Tetramorium aspis*.

Considering its local abundance, relative flexibility in habitat requirements, and widespread distribution, *Tetramorium cognatum* shows startlingly little intraspecific variation. From the southern spiny forest Lavanono and the montane rainforests in Andohahela to the northernmost known localities at Montagne d’Ambre/Sakaramy, *Tetramorium cognatum* is always easily recognisable. However, some noticeable variation in sculpture and body colouration still exists. Colouration ranges from very light yellow through all shades of yellowish orange or brown to very dark brown, almost black. The southeastern and northern populations are mostly light yellow to light brown while most material from the central-eastern populations is mainly brown to very dark brown. There is also some variation in the degree to which sculpture is developed on the clypeus and the sides of the head and mesosoma as outlined in the description above.

###### Tetramorium freya

Hita Garcia & Fisher sp. n.

http://zoobank.org/65E4760A-E053-4CB1-809A-7DE69A408EE3

http://species-id.net/wiki/Tetramorium_freya

Figs 21A , 33 , 63

####### Type material.

**Holotype**, pinned worker, MADAGASCAR, Antsiranana, Diego-Suarez, 7 km N Joffreville [camp 2 of Fisher], 12.33333°S, 49.25°E, 360 m, in dry forest, Malaise trap, collection code MA-01-07-08, 6.–20.III.2001 (*R. Harin’Hala*) (CAS: CASENT0085551). **Paratype**, one pinned worker with same data as holotype except collected 20.III.–7.IV.2001 and collection code MA-01-07-09 (CASENT0083419).

**Figure 33.**
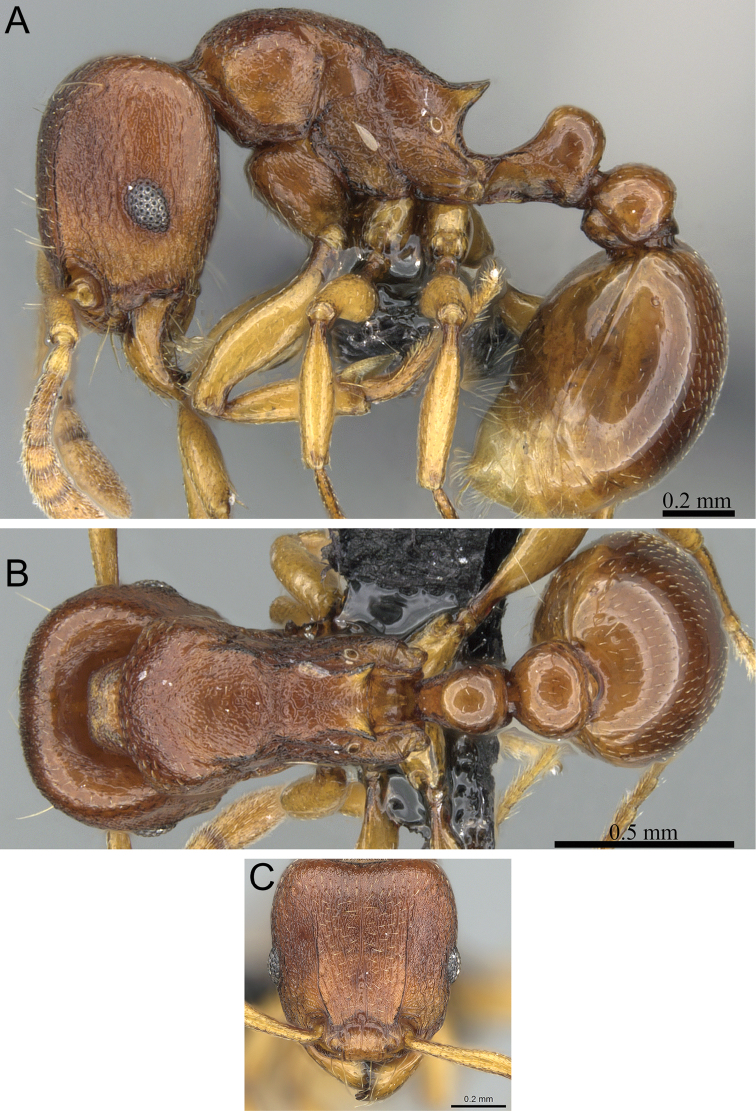
*Tetramorium freya* holotype worker (CASENT0085551). **A** Body in profile **B** Body in dorsal view **C** Head in full-face view.

####### Non-type material.

MADAGASCAR: Antsiranana, 4 km SW Ambositra (=Joffreville), 12.51667°S, 49.18333°E, 1000 m, rainforest,1.XII.1990 (*P.S. Ward*); Antsiranana, Nosy Be, Réserve Naturelle Intégrale de Lokobe, 6.3 km 112° ESE Hellville, 13.41933°S, 48.33117°E, 30 m, rainforest, 19.–24.III.2001 (*B.L. Fisher et al.*); Antsiranana, Sakalava Beach (vegetated beach dunes), 12.26278°S, 49.3975°E, 10 m, across sandy trail in dwarf littoral forest, 20.–28.VII.2001 (*R. Harin’Hala*).

####### Diagnosis.

The following character combination distinguishes *Tetramorium freya* from the other species of the *Tetramorium cognatum* species complex: frontal carinae weakly developed, merging with surrounding sculpture halfway between posterior eye margin and posterior head margin; petiolar node high nodiform with well rounded margins, in profile around 1.6 to 1.7 times higher than long (LPeI 58–62), in dorsal view 1.2 to 1.3 times wider than long (DPeI 124–131); lack of any long, standing pilosity on dorsal mesosoma.

####### Worker measurements

**(N=5).** HL 0.61–0.74 (0.68); HW 0.56–0.68 (0.62); SL 0.40–0.46 (0.44); EL 0.14–0.17 (0.16); PH 0.30–0.35 (0.33); PW 0.38–0.43 (0.40); WL 0.72–0.87 (0.81); PSL 0.12–0.15 (0.13); PTL 0.13–0.16 (0.15); PTH 0.21–0.27 (0.25); PTW 0.17–0.20 (0.19); PPL 0.16–0.21 (0.19); PPH 0.22–0.28 (0.25); PPW 0.23–0.28 (0.25); CI 90–92 (91); SI 68–71 (70); OI 25; DMI 44–54 (50); LMI 40–42 (41); PSLI 18–20 (19); PeNI 42–50 (46); LPeI 58–62 (59); DPeI 124–131 (127); PpNI 59–70 (63); LPpI 72–77 (75); DPpI 127–144 (134); PPI 133–140 (136).

####### Worker description.

Head much longer than wide (CI 90–92); in full-face view posterior head margin weakly concave. Anterior clypeal margin with distinct median impression. Frontal carinae weakly to moderately developed, merging with surrounding sculpture halfway between posterior eye margin and posterior head margin. Antennal scrobes weak to absent, shallow and without clear and distinct posterior and ventral margins. Antennal scapes very short, not reaching posterior head margin (SI 68–71). Eyes relatively large (OI 25). Mesosomal outline in profile weakly to moderately convex, moderately high and stout (LMI 40–42), weakly to moderately marginate from lateral to dorsal mesosoma; promesonotal suture absent; metanotal groove present, but weakly developed. Propodeal spines short, triangular to elongate-triangular, and acute (PSLI 18–20), propodeal lobes triangular and short, always much shorter than propodeal spines. Petiolar node in profile high nodiform with well-rounded margins, around 1.6 to 1.7 times higher than long (LPeI 58–62), anterior and posterior faces approximately parallel and rounding smoothly onto dorsum, anterodorsal and posterodorsal margins situated at about same height, petiolar dorsum relatively noticeably convex; node in dorsal view 1.2 to 1.3 times wider than long (DPeI 124–131), in dorsal view pronotum between 2.0 to 2.4 times wider than petiolar node (PeNI 42–50). Postpetiole in profile globular to subglobular, between 1.3 to 1.4 times higher than long (LPpI 72–77); in dorsal view around 1.3 to 1.4 times wider than long (DPpI 127–144), pronotum between 1.4 to 1.8 times wider than postpetiole (PpNI 59–70). Postpetiole in profile appearing approximately as voluminous as petiolar node, postpetiole in dorsal view around 1.3 to 1.4 times wider than petiolar node (PPI 133–140). Mandibles completely unsculptured, smooth, and shiny; clypeus in parts irregularly longitudinally rugulose, median ruga never fully developed, usually reduced to a few traces, one or two irregular and broken lateral rugulae present on each side; cephalic dorsum between frontal carinae weakly irregularly longitudinally rugulose, rugulae weak, often interrupted or with cross-meshes and becoming much weaker posteriorly; scrobal area mostly irregularly longitudinally rugulose and merging with surrounding irregularly longitudinally rugose to reticulate-rugose sculpture present on lateral head. Head with very conspicuous punctate ground sculpture. Mesosoma laterally and dorsally with traces of irregular rugulae or reticulate-rugose sculpture overlaying a distinct punctate ground sculpture. Forecoxae in parts weakly longitudinally rugulose and partly unsculptured, smooth, and shining. Both waist segments and gaster fully unsculptured, smooth, and shining. Dorsum of head with several pairs of hairs. Mesosoma, waist segments, and first gastral tergite without any standing hairs at all; first gastral tergite with very short, moderately dense, and appressed pubescence. Anterior edges of antennal scapes and dorsal (outer) surfaces of hind tibiae with appressed hairs. Head, mesosoma, waist segments, and gaster uniform brown, appendages yellowish to light brown.

####### Etymology.

The name of the new species was inspired and derived from the name of the Old Norse goddess Freyja (which means “Lady” in Old Norse) who was considered the goddess of love, beauty, fertility, war, and death. As a name taken from Norse mythology, it is treated as an arbitrary combination of letters, thus invariant.

####### Distribution and biology.

*Tetramorium freya* is a rare species only known from a few localities at the northernmost tip of Madagascar and from the island of Nosy Be (Fig. 63). The species was collected in rainforests, dry forests, and coastal dunes at elevations from 10 to 1000 m. Also, *Tetramorium freya* was mostly sampled from Malaise traps or from vegetation and only once from leaf litter; considering there are only five known specimens, it seems likely that *Tetramorium freya* nests and forages in vegetation. Additional sampling in lower vegetation might yield more material of this otherwise very rare species.

####### Discussion.

The identification of *Tetramorium freya* is easy and straightforward since it is the only species of the species complex without any long, standing pilosity on the mesosomal dorsum. Beyond mesosomal pilosity, *Tetramorium freya* also has weakly developed frontal carinae and sculpture on the head and mesosoma, which separate it from *Tetramorium gladius*, *Tetramorium myrmidon*, *Tetramorium proximum*, and *Tetramorium tenuinode*. In addition, *Tetramorium freya* possesses a stouter and higher mesosoma (LMI 40–42) and a lower and thicker petiolar node (LPeI 58–62, around 1.6 to 1.7 times higher than long) compared to *Tetramorium aspis*, *Tetramorium camelliae*, *Tetramorium cognatum*, *Tetramorium karthala*, and *Tetramorium rumo* (LMI 35–40; LPeI 33–55, around 1.8 to 3.0 times higher than long).

Based on the limited number of specimens available for this study, there seems to be no significant intraspecific variation in *Tetramorium freya*.

###### Tetramorium gladius

Hita Garcia & Fisher sp. n.

http://zoobank.org/04D35BE9-26E7-450E-B5FC-3EE67E7B7934

http://species-id.net/wiki/Tetramorium_gladius

**Figure 34.**
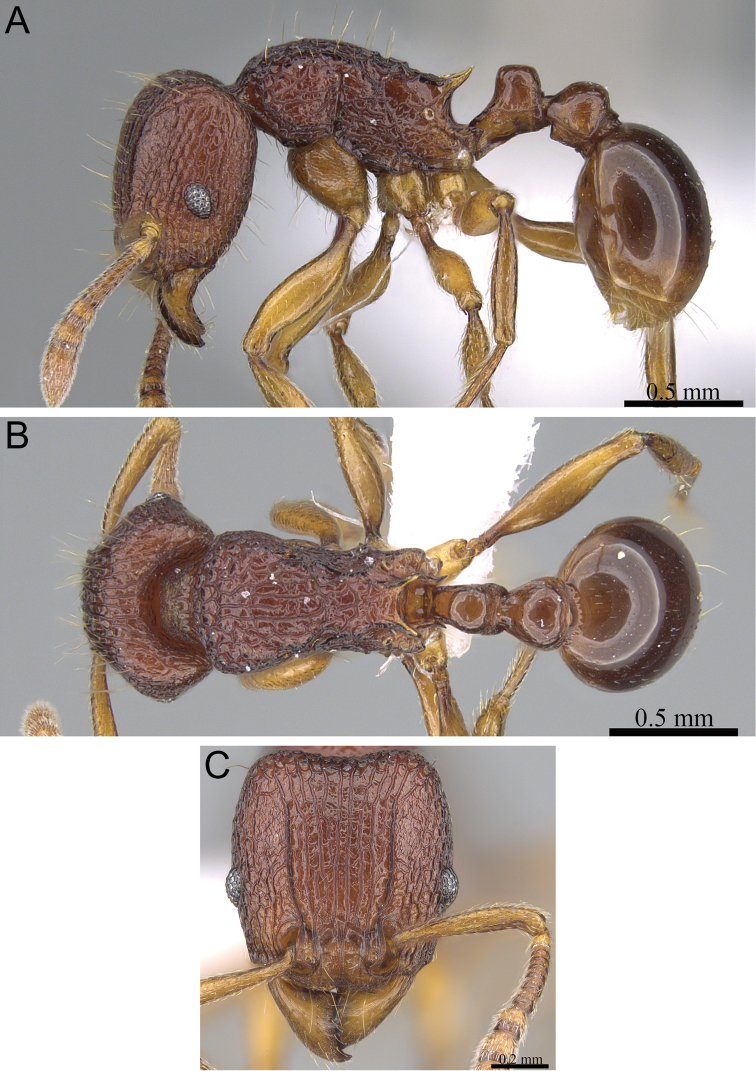
*Tetramorium gladius* holotype worker (CASENT0406982). **A** Body in profile **B** Body in dorsal view **C** Head in full-face view.

Figs 21C , 23A , 34 , 63

####### Type material.

**Holotype**, pinned worker, MADAGASCAR, Antananarivo, 3 km 41° NE Andranomay, 11.5 km 147° SSE Anjozorobe, 18.47333°S, 47.96°E, 1300 m, montane rainforest, sifted litter (leaf mold, rotten wood), collection code BLF02464, 5.–13.XII (*B.L. Fisher et al.*) (CAS: CASENT0406982). **Paratypes**, one pinned worker with same data as holotype (CAS: CASENT0406981); four pinned worker with same data as holotype but collected ex rotten stick on ground and collection codes BLF02407 and BLF02408 (CAS: CASENT0406964; CASENT0406971); fifteen pinned workers with same data as holotype but collected ex rotten log and collection codes BLF02429, BLF02498, and BLF02500 (CAS: CASENT0406966; CASENT0406967; CASENT0406968; CASENT0406969; CASENT0406974; CASENT0406975; MCZ: CASENT0406976); one pinned worker with same data as holotype but collected ex rotten tree stump and collection code BLF02486 (BMNH: CASENT0406965).

####### Non-type material.

MADAGASCAR: Antananarivo, Réserve Spéciale d’Ambohitantely, 18.22444°S, 47.2774°E, 1490 m, montane forest, 9.III.2012 (*B.L. Fisher et al.*); Antananarivo, 3 km 41° NE Andranomay, 11.5 km 147° SSE Anjozorobe, 18.47333°S, 47.96°E, 1300 m, montane rainforest, 5.–13.XII (*B.L. Fisher et al.*); Antsiranana, Rés. Anjanaharibe-Sud, 9.2 km WSW Befingotra, 14.75°S, 49.46667°E, 1200 m, montane rainforest, 9.XI.1994 (*B.L. Fisher*); Antsiranana, Forêt de Binara, 9.4 km 235° SW Daraina, 13.26333°S, 49.6°E, 1100 m, montane rainforest, 5.–6.XII.2003 (*B.L. Fisher*); Toamasina, 6.9 km NE Ambanizana, Ambohitsitondroina, 15.58506°S, 50.00952°E, 825 m, rainforest, 2.XII.1993 (*B.L. Fisher*); Toamasina, 5.3 km SSE Ambanizana, Andranobe, 15.67133°S, 49.97395°E, 425 m, rainforest, 21.XI.1993 (*B.L. Fisher*); Toamasina, Réserve Spéciale Ambatovaky, Sandrangato river, 16.77274°S, 49.26551°E, 450 m, rainforest, 20.–22.II.2010 (*B.L. Fisher et al.*).

####### Diagnosis.

The relatively small eyes (OI 19–20) of *Tetramorium gladius* separate it from the other members of the *Tetramorium cognatum* species complex.

####### Worker measurements

**(N=12).** HL 0.71–0.87 (0.81); HW 0.65–0.82 (0.75); SL 0.48–0.58 (0.54); EL 0.13–0.16 (0.15); PH 0.32–0.41 (0.37); PW 0.47–0.61 (0.55); WL 0.87–1.08 (1.00); PSL 0.17–0.22 (0.19); PTL 0.14–0.18 (0.15); PTH 0.24–0.33 (0.29); PTW 0.17–0.22 (0.19); PPL 0.20–0.27 (0.23); PPH 0.25–0.34 (0.30); PPW 0.26–0.34 (0.29); CI 92–95 (93); SI 71–74 (72); OI 19–20 (20); DMI 54–56 (55); LMI 36–38 (37); PSLI 21–28 (23); PeNI 31–36 (35); LPeI 48–58 (53); DPeI 113–133 (124); PpNI 49–56 (53); LPpI 70–82 (78); DPpI 120–133 (125); PPI 148–159 (153).

####### Worker description.

Head longer than wide (CI 92–95); in full-face view posterior head margin weakly to moderately concave. Anterior clypeal margin with distinct median impression. Frontal carinae moderately to well developed, diverging posteriorly, either merging with surrounding sculpture halfway between posterior eye margin and posterior head margin or only becoming weaker after posterior eye level but still approaching posterior head margin. Antennal scrobes very weak, shallow, and without clear and distinct posterior and ventral margins. Antennal scapes short, not reaching posterior head margin (SI 71–74). Eyes relatively small (OI 19–20). Mesosomal outline in profile flat to weakly convex, moderately low and long (LMI 36–38), moderately marginate from lateral to dorsal mesosoma; promesonotal suture absent; metanotal groove mostly reduced and absent, if present weakly developed. Propodeal spines moderately long to long, elongate-triangular to spinose, and usually acute (PSLI 21–28), propodeal lobes short and triangular, always much shorter than propodeal spines. Petiolar node in profile nodiform to high rounded nodiform, around 1.7 to 2.1 times higher than long (LPeI 48–58), anterior and posterior faces approximately parallel, anterodorsal and posterodorsal margins usually situated at about same height and weakly rounded, petiolar dorsum weakly to moderately convex; node in dorsal view between 1.1 to 1.3 times wider than long (DPeI 113–133), in dorsal view pronotum between 2.8 to 3.2 times wider than petiolar node (PeNI 31–36). Postpetiole in profile globular, around 1.2 to 1.4 times higher than long (LPpI 70–82); in dorsal view around 1.2 to 1.3 times wider than long (DPpI 120–133), pronotum around 1.8 to 2.0 times wider than postpetiole (PpNI 49–56). Postpetiole in profile usually appearing more voluminous than petiolar node, postpetiole in dorsal view around 1.5 to 1.6 times wider than petiolar node (PPI 148–159). Mandibles completely unsculptured, smooth, and shiny; clypeus longitudinally rugose/rugulose with three to seven median ruga always fully developed and distinct, and one to three mostly unbroken lateral rugae/rugulae present on each side; cephalic dorsum between frontal carinae longitudinally rugose with seven to nine rugae, rugae running mostly unbroken from posterior clypeal margin to posterior head margin, sometimes interrupted or with cross-meshes; scrobal area partly unsculptured, but mostly merging with surrounding reticulate-rugose to longitudinally rugose sculpture present on lateral head. Dorsum and sides of mesosoma mostly irregularly longitudinally rugose. Forecoxae unsculptured, smooth, and shining. Ground sculpture on head and mesosoma usually weak to absent. Both waist segments and gaster fully unsculptured, smooth, and shining. Dorsum of head with several pairs of long, fine, standing hairs; dorsum of mesosoma with six to eight pairs on pronotum and mesonotum, anterior propodeum with one pair; waist segments and first gastral tergite without any standing hairs; first gastral tergite with very short, scarce, appressed pubescence. Anterior edges of antennal scapes and dorsal (outer) surfaces of hind tibiae with appressed, rarely decumbent hairs. Head, mesosoma, waist segments, and gaster uniform reddish, orange-brown, appendages always lighter, yellowish to light brown.

####### Etymology.

The name of the new species is the ancient Latin general word for sword, although it mostly refers to the short swords used by foot soldiers of the Roman legion. The name was chosen based on the shape of the propodeal spines in most specimens of *Tetramorium gladius*, which resemble this type of sword. The species epithet is a nominative noun, and thus invariant.

####### Distribution and biology.

*Tetramorium gladius* has a relatively wide but patchy distribution in central, central-eastern, and northern Madagascar (Fig. 63). The southernmost localities are Ambohitantely and Andranomay and the northernmost is Binara with Ambatovaky, Ambanizana, and Anjanaharibe-Sud in-between. All localities are rainforests and montane rainforests at elevations ranging from 425 to 1490 m. *Tetramorium gladius* seems to be only moderately common and living in the leaf litter.

####### Discussion.

The relatively small eyes of *Tetramorium gladius* (OI 19–20) differentiate it immediately from the remainder of the *Tetramorium cognatum* species complex, in which all species have much larger eyes (OI 24–31). Apart from this, *Tetramorium gladius* appears to be morphologically close to *Tetramorium myrmidon*, *Tetramorium proximum*, and *Tetramorium tenuinode* since they all share a larger body size and very well developed frontal carinae and sculpture.

Little morphological variation is observed in *Tetramorium gladius*, with the exception, that the propodeal spines are longer in specimens from Ambatovaky (PSLI 26–28) compared to the rest of the material (PSLI 21–24).

###### Tetramorium karthala

Hita Garcia & Fisher sp. n.

http://zoobank.org/9B534784-C0F8-4B8C-AC93-77B3CBDB6DCC

http://species-id.net/wiki/Tetramorium_karthala

**Figure 35.**
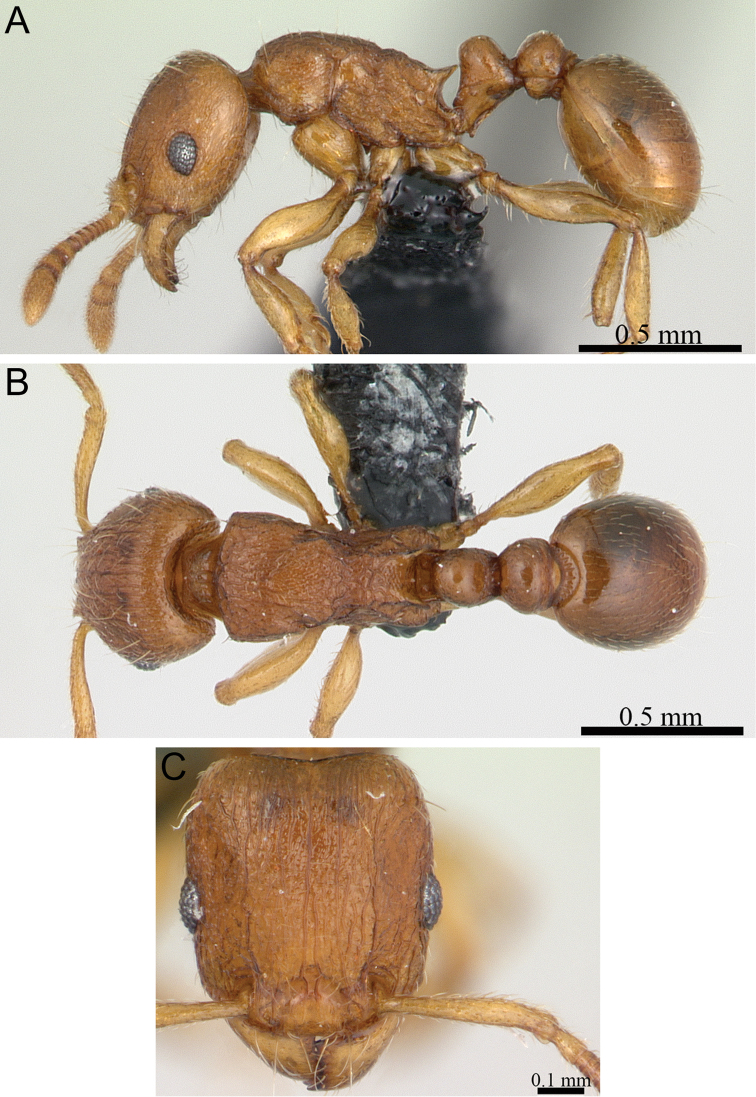
*Tetramorium karthala* paratype worker (CASENT0136774). **A** Body in profile **B** Body in dorsal view **C** Head in full-face view.

Figs 20A , 24C , 27D , 28D , 29B , 35 , 63

####### Type material.

**Holotype**, pinned worker, COMOROS, Grande Comore, Karthala, 11.82699°S, 43.4295°E, 1000 m, montane rainforest, sifted litter (leaf mold, rotten wood), collection code BLF19734, 14.–15.III.2008 (*B.L. Fisher et al.*) (CAS: CASENT0137302). **Paratypes**, three pinned workers with same data as holotype (CAS: CASENT0137426; CASENT0137440; CASENT0137510); two workers with same data as holotype except collected from rotten log and collection code BLF19750 (MCZ: CASENT0135232); six pinned workers from Grande Comore, Karthala, 11.81336°S, 43.41945°E, 1125 m, montane rainforest, sifted litter (leaf mold, rotten wood), collection code BLF19700, 13.–14.III.2008 (*B.L. Fisher et al.*) (BMNH: CASENT0136774; CAS: CASENT0136766; CASENT0137214; CASENT0137221; CASENT0137244; CASENT0137263;).

####### Non-type material.

COMOROS: Grande Comore, Karthala, 11.81336°S, 43.41945°E, 1125 m, montane rainforest, 13.III.2008 (*B.L. Fisher et al.*).

####### Diagnosis.

*Tetramorium karthala* is diagnosable within the *Tetramorium cognatum* complex on the basis of the following character combination: very large eyes (OI 29–30); short antennal scapes (SI 70–74); frontal carinae weakly to moderately developed; propodeal spines short to moderate, elongate-triangular to spinose, and usually acute (PSLI 20–22), propodeal lobes always much shorter than spines and lobes never strongly inclined towards each other; petiolar node rounded high nodiform, in profile around 1.8 to 2.0 times higher than long (LPeI 50–54), in dorsal view around 1.5 to 1.6 times wider than long (DPeI 148–158); mesosoma with two to four pairs of long, standing hairs on pronotum and mesonotum.

####### Worker measurements (N=10).

L 0.59–0.63 (0.61); HW 0.51–0.55 (0.53); SL 0.36–0.39 (0.38); EL 0.15–0.16 (0.15); PH 0.27–0.31 (0.29); PW 0.38–0.42 (0.40); WL 0.72–0.79 (0.75); PSL 0.12–0.14 (0.13); PTL 0.12–0.14 (0.13); PTH 0.23–0.26 (0.24); PTW 0.18–0.21 (0.19); PPL 0.15–0.17 (0.15); PPH 0.22–0.25 (0.23); PPW 0.22–0.25 (0.24); CI 86–87 (87); SI 70–74 (72); OI 29–30 (29); DMI 51–55 (54); LMI 37–39 (39); PSLI 20–22 (21); PeNI 45–49 (47); LPeI 50–54 (51); DPeI 148–158 (151); PpNI 55–61 (58); LPpI 65–68 (66); DPpI 138–158 (151); PPI 112–131 (124).

####### Worker description.

Head much longer than wide (CI 86–87); posterior head margin weakly concave. Anterior clypeal margin with distinct median impression. Frontal carinae weakly to moderately developed, only weakly raised, and ending between posterior head margin and posterior head margin. Antennal scrobes very weakly developed to almost absent, shallow and without clear and distinct posterior and ventral margins. Antennal scapes short, not reaching posterior head margin (SI 70–74). Eyes very large (OI 29–30). Mesosomal outline in profile flat to weakly convex, moderately low and long (LMI 37–39), moderately marginate from lateral to dorsal mesosoma; promesonotal suture absent; metanotal groove present and distinctly developed. Propodeal spines short to moderately long, elongate-triangular to spinose, and usually acute (PSLI 20–22), propodeal lobes short and triangular, always much shorter than propodeal spines, spines and lobes never strongly inclined towards each other. Petiolar node in profile rounded high nodiform, around 1.8 to 2.0 times higher than long (LPeI 50–54), usually anterior and posterior faces more or less parallel, anterodorsal and posterodorsal margins both well-rounded and usually situated at about same height, petiolar dorsum moderately convex; node in dorsal view around 1.5 to 1.6 times wider than long (DPeI 148–158), in dorsal view pronotum between 2.0 to 2.2 times wider than petiolar node (PeNI 45–49). Postpetiole in profile subglobular, between 1.4 to 1.6 times higher than long (LPpI 65–68); in dorsal view around 1.4 to 1.6 times wider than long (DPpI 138–158), pronotum around 1.6 to 1.8 times wider than postpetiole (PpNI 55–61). Postpetiole in profile usually appearing of more or less same volume as petiolar node, postpetiole in dorsal view around 1.1 to 1.3 times wider than petiolar node (PPI 112–131). Mandibles completely unsculptured, smooth, and shiny; clypeus usually irregularly longitudinally rugulose with three to five, often broken, rugulae, sometimes rugulae reduced to one or two, median ruga/rugula often developed, but usually broken, rarely completely absent; cephalic dorsum between frontal carinae longitudinally rugose/rugulose with seven to nine rugae/rugulae, rugae usually running from posterior clypeal margin to posterior head margin, often interrupted, rarely with cross-meshes; scrobal area mostly unsculptured with ground sculpture only, lateral head reticulate-rugose to longitudinally rugose. Dorsum and sides of mesosoma mostly irregularly longitudinally rugose/rugulose. Forecoxae dorsally with few traces of rugulae, otherwise mostly unsculptured, smooth, and shining. Ground sculpture on head and mesosoma very distinct, usually reticulate-punctate with few areas very finely reticulate-rugulose. Both waist segments and gaster fully unsculptured, smooth, and shining. Dorsum of head with numerous pairs of standing, long, fine hairs; mesosoma usually with two to four pairs: one long pair on anterior pronotum and one long pair on anterior mesonotum, sometimes two shorter pairs present, one on posterior pronotum and one on posterior mesonotum; propodeum, waist segments and first gastral tergite without any standing hairs; first gastral tergite with short, moderately dense, appressed pubescence. Anterior edges of antennal scapes and dorsal (outer) surfaces of hind tibiae with appressed to decumbent hairs. Head, mesosoma, waist segments and gaster uniform light brown, appendages often lighter, more yellowish brown.

####### Etymology.

The new species is named after the type locality, Karthala Forest on Mount Karthala volcano on the island of Grand Comore. The species epithet is a noun in apposition, and thus invariable.

####### Distribution and biology.

This new species is only known from the type locality, which is a montane rainforest on the Comorian island Grande Comore (Fig. 63). At present, it is the only described *Tetramorium* species endemic to any Comorian island. All other *Tetramorium* species (except one undescribed species from the *Tetramorium ranarum* species group from Anjouan) found on the Comoros Islands are either introduced tramp species or species shared between Africa, the Comoros and Madagascar, or between the Comoros and Madagascar. Intriguingly, the type locality appears to have a quite unique fauna. Just recently Fischer and Fisher (2013) described a new *Pheidole* species (*Pheidole vulcan* Fischer & Fisher) from this locality, which is also endemic to the island of Grande Comore. *Tetramorium karthala* is at elevations between 1000 to 1125 m where it was mainly collected from leaf litter, under moss, or within rotten logs.

####### Discussion.

*Tetramorium karthala* is easily identifiable in the Malagasy region since it is the only species of the *Tetramorium cognatum* species complex and the whole *Tetramorium schaufussii* species group found on the Comores. Even without considering geography the new species can be well distinguished from the remainder of its species complex. In general, it is morphologically close to *Tetramorium aspis*, *Tetramorium camelliae*, *Tetramorium cognatum*, and *Tetramorium rumo*, whereas it differs more strongly from the rest of the complex. Since *Tetramorium karthala* has several pairs of long, standing hairs on the dorsum of the mesosoma and much larger eyes (OI 29–30), it is unlikely to be mistaken for *Tetramorium freya*, which has no standing pilosity on the mesosomal dorsum, or for *Tetramorium gladius*, which has the smallest eyes in the complex (OI 19–20). Also, *Tetramorium karthala* cannot be confused with *Tetramorium myrmidon*, *Tetramorium proximum*, or *Tetramorium tenuinode* as the latter three have very well developed, noticeably raised, and long frontal carinae compared to the much weaker frontal carinae seen in *Tetramorium karthala*.

The separation from the other five species, which appear closer to *Tetramorium karthala*, is also straightforward. *Tetramorium camelliae* has a strongly squamiform petiolar node which is around 2.8 to 3.0 times higher than long (LPeI 33–36) and around 2.3 to 2.4 times wider than long (DPeI 228–238), while *Tetramorium karthala* has a high nodiform petiolar node which is around 1.8 to 2.0 times higher than long (LPeI 50–54) and around 1.5 to 1.6 times wider than long (DPeI 148–158). The smallest species of the complex, *Tetramorium rumo* (HW 0.43–0.49; WL 0.56–0.67), differs from *Tetramorium karthala* (HW 0.51–0.55; WL 0.72–0.79) not only in body size but also in the shape of the petiolar node. The node of *Tetramorium rumo* is thinly cuneiform and moderately squamiform, in profile 2.3 to 2.7 times higher than long (LPeI 37–43) while, as noted above, the node of *Tetramorium karthala* is high nodiform and thicker, only 1.8 to 2.0 times higher than long (LPeI 50–54). Furthermore, in *Tetramorium aspis* the propodeal spines and lobes are strongly inclined towards each other, whereas the spines and lobes of *Tetramorium karthala* are not. The latter species also has two to four pairs of long, standing hairs on the dorsal pronotum and mesonotum while *Tetramorium aspis* has at least six on the pronotum and mesonotum and usually one or two on the anterior propodeum. The last species in the complex, *Tetramorium cognatum*, appears morphologically very close to *Tetramorium karthala*, but has noticeably shorter antennal scapes (SI 61–67) and propodeal spines (PSLI 12–16) than *Tetramorium karthala* (SI 70–74; PSLI 20–22).

To our knowledge, there is no significant intraspecific variation in *Tetramorium karthala*.

###### Tetramorium myrmidon

Hita Garcia & Fisher sp. n.

http://zoobank.org/11BD4CF2-149D-4A6A-9B9C-C59C0030B466

http://species-id.net/wiki/Tetramorium_myrmidon

**Figure 36.**
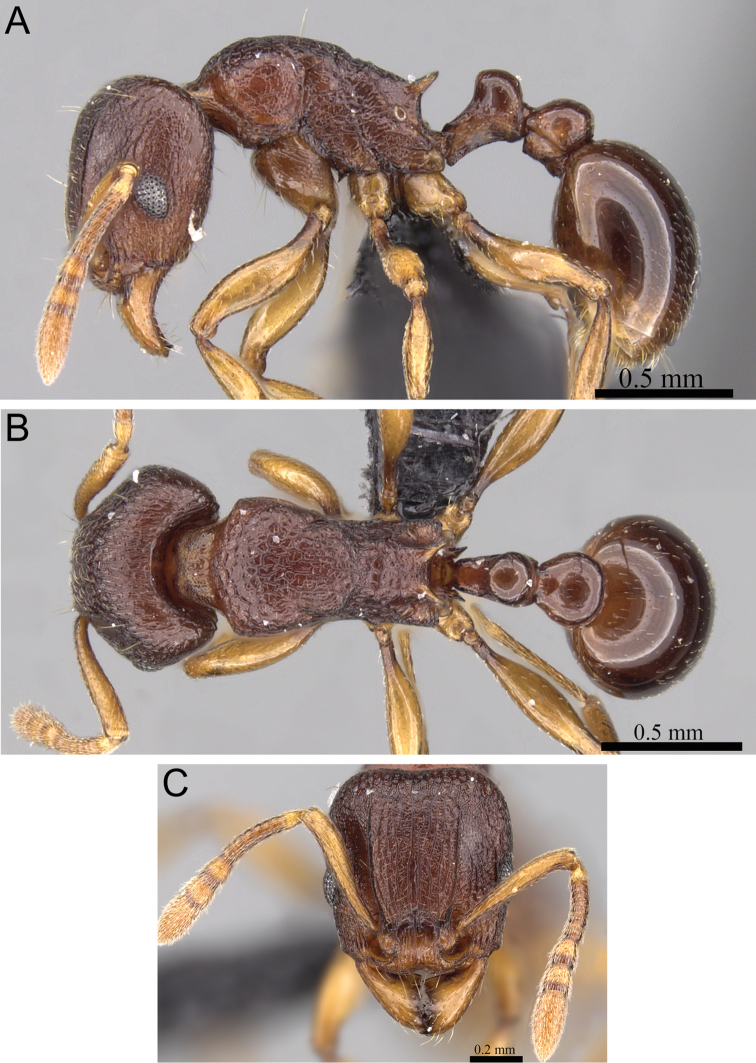
*Tetramorium myrmidon* holotype worker (CASENT0028635). **A** Body in profile **B** Body in dorsal view **C** Head in full-face view.

Figs 23C , 25B , 26C , 36 , 64

####### Type material.

**Holotype**, pinned worker, MADAGASCAR, Toliara, Réserve Spéciale d’Ambohijanahary, Forêt d’Ankazotsihitafototra, 35.2 km 312° NW Ambaravaranala, 18.26667°S, 45.40667°E, 1050 m, montane rainforest, sifted litter (leaf mold, rotten wood), collection code BLF07020, 13.–17.I.2003 (*B.L. Fisher et al.*) (CAS: CASENT0028635). **Paratypes**, two workers with same data as holotype (CAS: CASENT0028642; CASENT0028643); and three workers MADAGASCAR, Toliara, Réserve Spéciale d’Ambohijanahary, Forêt d’Ankazotsihitafototra, 34.6 km 314° NW Ambaravaranala, 18.26°S, 45.41833°E, 1100 m, montane rainforest, sifted litter (leaf mold, rotten wood), collection code BLF07086, 16.I.2003 (*B.L. Fisher et al.*) (CAS: CASENT0029810; CASENT0029821; CASENT0030099).

####### Diagnosis.

*Tetramorium myrmidon* can be easily distinguished from the other members of the group by the following combination of characters: eyes relatively large (OI 24–25); antennal scapes short (SI 74–76); frontal carinae well developed, noticeably raised, ending shortly before posterior head margin; petiolar node high rounded nodiform, in profile around 1.7 times higher than long (LPeI 58–60), in dorsal view around 1.2 to 1.3 times wider than long (DPeI 121–129); in dorsal view postpetiole around 1.3 to 1.5 times broader than petiolar node (PPI 133–141); dorsum of mesosoma with two pairs of long, standing hairs on pronotum and mesonotum.

####### Worker measurements

**(N=6).** HL 0.70–0.76 (0.73); HW 0.62–0.69 (0.65); SL 0.47–0.52 (0.49); EL 0.16–0.18 (0.16); PH 0.33–0.37 (0.35); PW 0.45–0.50 (0.47); WL 0.85–0.94 (0.89); PSL 0.13–0.16 (0.14); PTL 0.14–0.16 (0.15); PTH 0.24–0.27 (0.25); PTW 0.18–0.20 (0.19); PPL 0.18–0.21 (0.19); PPH 0.24–0.28 (0.26); PPW 0.24–0.28 (0.26); CI 88–91 (90); SI 74–76 (75); OI 24–25 (25); DMI 52–54 (53); LMI 38–40 (39); PSLI 18–20 (19); PeNI 39–40 (39); LPeI 58–60 (59); DPeI 121–129 (125); PpNI 52–55 (54); LPpI 73–77 (76); DPpI 129–136 (133); PPI 133–141 (138).

####### Worker description.

Head much longer than wide (CI 88–91); in full-face view posterior head margin weakly concave. Anterior clypeal margin with distinct median impression. Frontal carinae well developed, noticeably raised, diverging posteriorly, merging with surrounding sculpture shortly before posterior head margin. Antennal scrobes very weakly developed, almost absent, shallow and without clear and distinct posterior and ventral margins. Antennal scapes short, not reaching posterior head margin (SI 74–76). Eyes relatively large (OI 24–25). Mesosomal outline in profile flat to weakly convex, moderately low and long (LMI 38–40), moderately marginate from lateral to dorsal mesosoma; promesonotal suture absent; metanotal groove present, but weakly developed. Propodeal spines short to moderate, elongate-triangular, and acute (PSLI 18–20), propodeal lobes triangular and short, always much shorter than propodeal spines. Petiolar node in profile high nodiform with well rounded margins, around 1.7 times higher than long (LPeI 58–60), anterior and posterior faces approximately parallel, anterodorsal and posterodorsal margins situated at about same height, anterodorsal margin slightly more angulate than posterodorsal, more rounded margin, petiolar dorsum relatively flat to weakly convex; node in dorsal view 1.2 to 1.3 times wider than long (DPeI 121–129), in dorsal view pronotum between 2.5 to 2.6 times wider than petiolar node (PeNI 39–40). Postpetiole in profile globular, between 1.3 to 1.4 times higher than long (LPpI 73–77); in dorsal view around 1.3 to 1.4 times wider than long (DPpI 129–136), pronotum around 1.8 to 1.9 times wider than postpetiole (PpNI 52–55). Postpetiole in profile appearing more voluminous than petiolar node, postpetiole in dorsal view around 1.3 to 1.4 times wider than petiolar node (PPI 133–141). Mandibles completely unsculptured, smooth, and shiny; clypeus irregularly longitudinally rugulose with three to seven rugulae, median ruga usually fully developed, one to three mostly unbroken lateral rugulae present on each side; cephalic dorsum between frontal carinae longitudinally rugulose with seven to nine rugulae, rugulae usually running from posterior clypeal margin to posterior head margin, often interrupted or with cross-meshes, especially posteriorly; scrobal area mostly unsculptured with ground sculpture only, lateral head reticulate-rugose to longitudinally rugulose. Ground sculpture on head well developed and distinct, mostly reticulate-punctate. Dorsum and sides of mesosoma irregularly longitudinally rugulose to reticulate-rugulose. Forecoxae dorsally weakly rugulose, but mostly unsculptured, smooth, and shining. Ground sculpture on mesosoma weakly to moderately punctate. Both waist segments and gaster fully unsculptured, smooth, and shining. Dorsum of head with several pairs of standing, long, fine hairs; dorsum of mesosoma with two pairs only, one on anterior pronotum and one on anterior mesonotum; propodeum, waist segments, and first gastral tergite without any standing hairs; first gastral tergite with very short, scarce, appressed pubescence. Anterior edges of antennal scapes and dorsal (outer) surfaces of hind tibiae with appressed hairs. Head, mesosoma, waist segments, and gaster uniformly brown to dark brown, appendages yellowish to light brown.

####### Etymology.

The name of the new species is inspired by the ancient Greek “myrmidons”, which were skilled warriors and known as the legendary “ant-people” who inhabited the Greek island of Aegina. The species epithet is a noun in apposition, and thus invariant.

####### Distribution and biology.

The new species is so far only known from Ambohijanahary, which is an isolated montane rainforest located in the midwest of Madagascar (Fig. 64), where it was collected only twice. With only six known specimens it represents an extremely rare species. *Tetramorium myrmidon* was collected from leaf litter at elevations of 1050 to 1100 m.

####### Discussion.

Based on its larger body size and very well developed frontal carinae, *Tetramorium myrmidon* differs strongly from the smaller species *Tetramorium aspis*, *Tetramorium camelliae*, *Tetramorium cognatum*, *Tetramorium karthala* and *Tetramorium rumo*, all with more weakly developed frontal carinae, while being presumably morphologically closer to *Tetramorium gladius*, *Tetramorium proximum* and *Tetramorium tenuinode*. Of these, *Tetramorium gladius* possesses very small eyes (OI 19–20), while the eyes of *Tetramorium myrmidon* are much larger (OI 24–25). *Tetramorium tenuinode* has shorter antennal scapes (SI 66–70) and a thinner petiolar node, in profile 1.8 to 2.2 times higher than long (LPeI 45–54), in contrast to *Tetramorium myrmidon* with its longer scapes (SI 74–76) and lower and thicker petiolar node, which is in profile around 1.7 times higher than long (LPeI 58–60). The widespread *Tetramorium proximum*, however, appears to be the species morphologically closest to *Tetramorium myrmidon* and most of their morphometric ranges and characters overlap. Nonetheless, we consider both sufficiently demarcated from each other since they are found to co-occur in sympatry in Ambohijanahary. The specimens of *Tetramorium proximum* from this locality differ from *Tetramorium myrmidon* by having five to six pairs of long, standing hairs on the mesosoma and a generally thinner and higher petiolar node. Finally, *Tetramorium myrmidon* is unlikely to be confused with the last species of the complex, *Tetramorium freya*, since the latter lacks long, standing pilosity on the mesosomal dorsum (present in *Tetramorium myrmidon*).

The small number of specimens from just two collection events in the same locality does not permit proper assessment of intraspecific variation.

###### Tetramorium proximum

Bolton, 1979

http://species-id.net/wiki/Tetramorium_proximum

**Figure 37.**
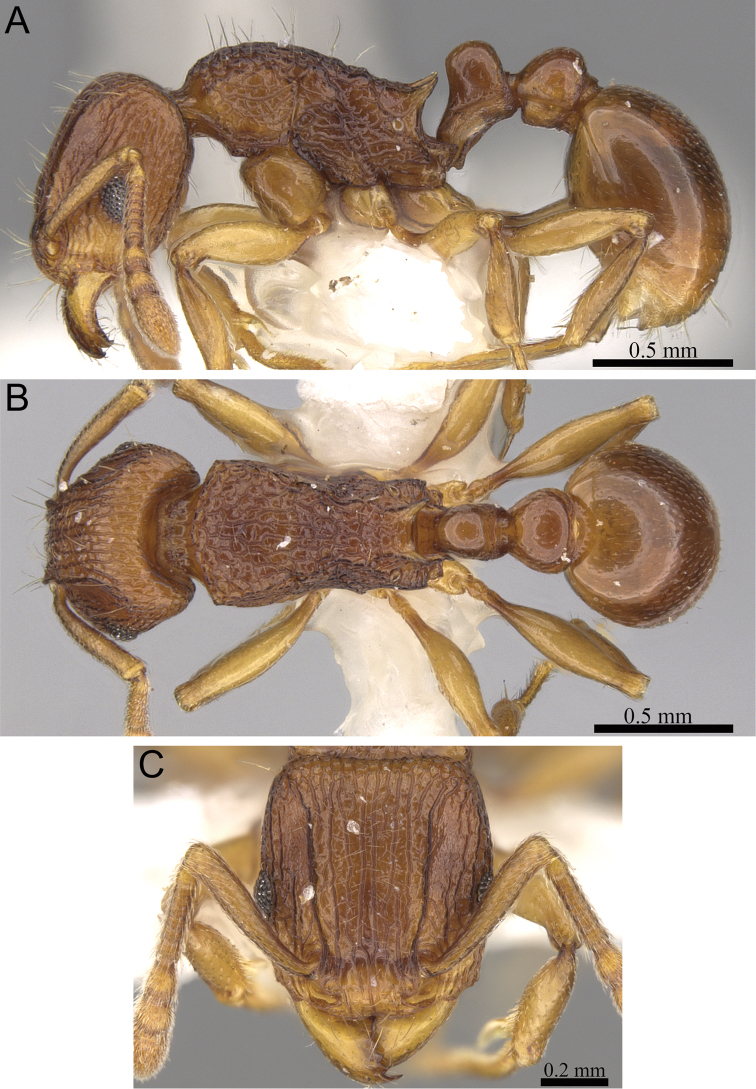
*Tetramorium proximum* holotype worker (CASENT0906146). **A** Body in profile **B** Body in dorsal view **C** Head in full-face view.

Figs 20B , 22D , 24A , 25A , 37 , 64

Tetramorium proximum Bolton, 1979: 137.

####### Type material.

**Holotype**, pinned worker, MADAGASCAR, Toamasina, Périnet & vicinity, 18.82667°S, 48.44778°E, rainforest, rotten wood, 18.III.1969 (*W. L. Brown*) (MCZ: MCZ_Holotype_32264) [examined]. **Paratypes**, ten pinned workers and one pinned queen with same data as holotype (BMNH: CASENT0102341; CASENT0102342; CASENT0235211; MCZ: MCZ_Paratype_32264).

[Note: the GPS data of the type locality was not provided either by the locality label or the original description. The data presented above is based on our own geo-referencing of the Périnet=Andasibe area and should be considered an approximation and not the exact position of the type locality.]

####### Non-type material.

MADAGASCAR: Antananarivo, Tsimbazaza, 18.928°S, 47.527°E, 1300 m, park/garden, 16.XII.2006 (*B.L. Fisher et al.*); Antsiranana, Parc National Montagne d’Ambre, 3.6 km 235° SW Joffreville, 12.53444°S, 49.1795°E, 925 m, montane rainforest, 20.–26.I.2001 (*B.L. Fisher et al.*); Antsiranana, Parc National Montagne d’Ambre, 12.2 km 211° SSW Joffreville, 12.59639°S, 49.1595°E, 1300 m, montane rainforest, 7.II.2001 (*G.D. Alpert*); Antsiranana, Parc National Montagne d’Ambre [1st campsite], 12.51444°S, 49.18139°E, 960 m, rainforest, 5.–21.IV.2001 (*R. Harin’Hala*); Antsiranana, Parc National Montagne d’Ambre, 12.51389°S, 49.17784°E, 984 m, montane rainforest, 23.II.2001 (*B.L. Fisher et al.*); Antsiranana, Parc National Montagne d’Ambre, 12.51778°S, 49.17957°E, 1000 m, montane rainforest, 3.–6.III.2001 (*B.L. Fisher et al.*); Antsiranana, Parc National Montagne d’Ambre, 12.52306°S, 49.17901°E, 1100 m, montane rainforest, 11.III.2001 (*B.L. Fisher et al.*); Antsiranana, Parc National Montagne d’Ambre, Lac maudit, 12.58502°S, 49.15147°E, 1250 m, montane rainforest, 13.XI.2007 (*B.L. Fisher et al.*); Antsiranana, Parc National Montagne d’Ambre, Roussettes, 12.52574°S, 49.17238°E, 1025 m, montane rainforest, 15.XI.2007 (*B.L. Fisher et al.*); Antsiranana, Parc National Montagne d’Ambre, Petit lac, 12.53664°S, 49.17412°E, 1130 m, montane rainforest, 17.XI.2007 (*B.L. Fisher et al.*); Antsiranana, Parc National Montagne d’Ambre, Antomboka, 12.51269°S, 49.17807°E, 970 m, montane rainforest, 17.XI.2007 (*B.L. Fisher et al.*); Antsiranana, Parc National Montagne d’Ambre, Mahasarika, 12.53176°S, 49.17662°E, 1135 m, montane rainforest, 17.XI.2007 (*B.L. Fisher et al.*); Antsiranana, Rés. Anjanaharibe-Sud, 6.5 km SSW Befingotra, 14.75°S, 49.5°E, 875 m, rainforest, 19.X.1994 (*B.L. Fisher*); Antsiranana, Forêt de Binara, 9.1 km 233° SW Daraina, 13.26333°S, 49.60333°E, 800 m, rainforest, 3.XII.2003 (*B.L. Fisher*); Antsiranana, Forêt de Binara, 9.4 km 235° SW Daraina, 13.26333°S, 49.6°E, 1100 m, montane rainforest, 5.XII.2003 (*B.L. Fisher*); Antsiranana, Betaolana Forest, along Bekona River, 14.52996°S, 49.44039°E, 880 m, rainforest, 4.–5.III.2009 (*B.L. Fisher et al.*); Antsiranana, R.S. Manongarivo, 10.8 km 229° SW Antanambao, 13.96167°S, 48.43333°E, 400 m, rainforest, 8.–13.X.1998 (*B.L. Fisher*); Antsiranana, R.S. Manongarivo, 12.8 km 228° SW Antanambao, 13.97667°S, 48.42333°E, 780 m, rainforest, 11.X.1998 (*B.L. Fisher*); Antsiranana, R.S. Manongarivo, 14.5 km 220° SW Antanambao, 13.99833°S, 48.42833°E, 1175 m, montane rainforest, 20.X.1998 (*B.L. Fisher*); Antsiranana, Parc National de Marojejy, Manantenina River, 28.0 km 38° NE Andapa, 8.2 km 333° NNW Manantenina, 14.43667°S, 49.775°E, 450 m, rainforest, 12.–15.XI.2003 (*B.L. Fisher et al.*); Antsiranana, Parc National de Marojejy, Manantenina River, 27.6 km 35° NE Andapa, 9.6 km 327° NNW Manantenina, 14.435°S, 49.76°E, 775 m, rainforest, 15.–18.XI.2003 (*B.L. Fisher et al.*); Fianarantsoa, 45 km S Ambalavao, 22.21667°S, 47.01667°E, 785 m, rainforest, 25.–30.IX.1993 (*B.L. Fisher*); Fianarantsoa, 2 km W Andrambovato, along river Tatamaly, 21.51167°S, 47.41°E, 1075 m, montane rainforest, 3.–5.VI.2005 (*B.L. Fisher et al.*); Fianarantsoa, Rés. Andringitra, 43 km S Ambalavao, 22.23333°S, 47°E, 825 m, rainforest, 5.X.1993 (*B.L. Fisher*); Fianarantsoa, Rés. Andringitra, 40 km S Ambalavao, 22.21667°S, 46.96667°E, 1275 m, montane rainforest, 15.–19.X.1993 (*B.L. Fisher*); Fianarantsoa, Rés. Andringitra, 38 km S Ambalavao, 22.2°S, 46.96667°E, 1680 m, montane rainforest, 23.X.1993 (*B.L. Fisher*); Fianarantsoa, Parc National Befotaka-Midongy, Papango 28.5 km S Midongy-Sud, Mount Papango, 23.84083°S, 46.9575°E, 1250 m, montane rainforest, 17.–18.XI.2006 (*B.L. Fisher et al.*); Fianarantsoa, 900 m E of Isalo National Park Interpretive Center, stream area, 22.62667°S, 45.35817°E, 750 m, open area near stream, 3.–10.III.2002 (*R. Harin’Hala*); Fianarantsoa, R.S. Ivohibe, 7.5 km ENE Ivohibe, 22.47°S, 46.96°E, 900 m, rainforest, 7.–12.X.1997 (*B.L. Fisher*); Fianarantsoa, R.S. Ivohibe, 8.0 km E Ivohibe, 22.48333°S, 46.96833°E, 1200 m, montane rainforest, 15.–21.X.1997 (*B.L. Fisher*); Fianarantsoa, R.S. Ivohibe, 6.5 km ESE Ivohibe, 22.49667°S, 46.955°E, 1575 m, montane rainforest, 24.–30.X.1997 (*B.L. Fisher*); Fianarantsoa, 8.0 km NE Ivohibe, 22.42167°S, 46.89833°E, 1200 m, montane rainforest, 3.–9.XI.1997 (*B.L. Fisher*); Fianarantsoa, 9.0 km NE Ivohibe, 22.42667°S, 46.93833°E, 900 m, rainforest, 12.–17.XI.1997 (*B.L. Fisher*); Fianarantsoa, Parc Nationale Ranomafana, Talatakely, 21.24833°S, 47.42667°E, in guava forest, 9.–26.IV.1998 (*C.E. Griswold et al.*); Fianarantsoa, Ranomafana National Park, Vohiparara Kidonavo 2, 21.22603°S, 47.36963°E, 1100 m, 13.III.2003 (*V.C. Clark*); Fianarantsoa, Parc National de Ranomafana, Vatoharanana, 21.28955°S, 47.4304, 1100 m, 30.III.2003 (*V.C. Clark*); Fianarantsoa, Parc National de Ranomafana, Vatoharanana River, 4.1 km 231° SW Ranomafana, 21.29°S, 47.43333°E, 1100 m, montane rainforest, 27.–31.III.2003 (*B.L. Fisher et al.*); Fianarantsoa, Forêt Classée Vatovavy, 7.6 km 122° Kianjavato, 21.4°S, 47.94°E, 175 m, rainforest, 6.–8.VI.2005 (*B.L. Fisher et al.*); Fianarantsoa, Forêt de Vevembe, 66.6 km 293° Farafangana, 22.791°S, 47.18183°E, 600 m, rainforest, transition to montane forest, 23.–24.IV.2006 (*B.L. Fisher et al.*); Mahajanga, Réserve Spéciale Marotandrano, Marotandrano 48.3 km S Mandritsara, 16.28322°S, 48.81443°E, 865 m, transition humid forest, 7.XII.2007 (*B.L. Fisher et al.*); Toamasina, 6.3 km S Ambanizana, Andranobe, 15.6813°S, 49.958°E, 25 m, rainforest, 14.XI.1993 (*B.L. Fisher*); Toamasina, Forêt Ambatovy, 14.3 km 57° Moramanga, 18.85083°S, 48.32°E, 1075 m, montane rainforest, 21.III.2004 (*Malagasy ant team*); Toamasina, Ambatovy, 12.4 km NE Moramanga, 18.84773°S, 48.29568°E, 1000–1010 m, montane rainforest, 3.–8.III.2007 (*B.L. Fisher et al.*); Toamasina, Amparihibe, 15°2’ S, 49°34’ E, II.–III.2003 (*K.A. Jackson & D. Carpenter*); Toamasina, Station Forestière Analamazaotra, Analamazaotra 1.3 km S Andasibe, 18.38466°S, 48.41271°E, 980 m, montane rainforest, 11.–13.XII.2007 (*B.L. Fisher et al.*); Toamasina, Montagne d’Anjanaharibe, 18.0 km 21° NNE Ambinanitelo, 15.18833°S, 49.615°E, 470 m, rainforest, 8.–12.III.2003 (*B.L. Fisher et al.*); Toamasina, Ankerana, 18.40062°S, 48.81311°E, 865 m, rainforest, 17.–18.I.2012 (*B.L. Fisher et al.*); Toamasina, Ankerana, 18.40829°S, 48.82107°E, 750 m, rainforest, 21.–22.I.2012 (*B.L. Fisher et al.*); Toamasina, Ankerana, 18.40636°S, 48.80254°E, 1108 m, montane rainforest, 29.I.2012 (*B.L. Fisher et al.*); Toamasina, Reserve Betampona, Camp Rendrirendry, 34.1 km 332° Toamasina, 17.924°S, 49.19967°E, 390 m, rainforest, 28.XI.2005 (*B.L. Fisher et al.*); Toamasina, Réserve Nationale Intégrale Betampona, Betampona 35.1 km NW Toamasina, 17.91801°S, 49.20074°E, 500 m, rainforest, 16.XII.2007 (*B.L. Fisher et al.*); Toamasina, Réserve Naturelle Betampona, 34.08 km 332° Toamasina, 17.91977°S, 49.20039°E, 525 m, rainforest, 17.II.–5.X.2008 (*B.L. Fisher*); Toamasina, Réserve Naturelle Betampona, 34.1 km 332° Toamasina, 17.916136°S, 49.20185°E, 550 m, rainforest, 5.X.–14.XII.2008 (*B.L. Fisher*); Toamasina, F.C. Didy, 18.19833°S, 48.57833°E, 960 m, rainforest, 16.–23.XII.1998 (*H.J. Ratsirarson*); Toamasina, Parc National Mananara-Nord, 7.1 km 261° Antanambe, 16.455°S, 49.7875°E, 225 m, rainforest, 14.XI.2005 (*B.L. Fisher et al.*); Toamasina, P.N. Mantadia, 18.79167°S, 48.42667°E, 895 m, rainforest, 28.XI.–1.XII.1998 (*H.J. Ratsirarson*); Toamasina, F.C. Sandranantitra, 18.04833°S, 49.09167°E, 450 m, rainforest, 21.I.1999 (*H.J. Ratsirarson*); Toamasina, Torotorofotsy, 18.87082°S, 48.34737°E, 1070 m, montane rainforest, marsh edge, 24.–27.III.2004 (*Malagasy ant team*); Toamasina, Parc National de Zahamena, Tetezambatana forest, near junction of Nosivola and Manakambahiny rivers, 17.74298°S, 48.72936°E, 860 m, rainforest, 18.–19.II.2009 (*B.L. Fisher et al.*); Toamasina, Parc National de Zahamena, 17.73359°S, 48.72625°E, 950 m, rainforest, 19.II.2009 (*B.L. Fisher et al.*); Toamasina, Parc National de Zahamena, Onibe River, 17.75908°S, 48.85468°E, 780 m, rainforest, 21.II.2009 (*B.L. Fisher et al.*); Toamasina, Parc National de Zahamena, Besaky River, 17.75244°S, 48.85321°E, 760 m, rainforest, 22.II.2009 (*B.L. Fisher et al.*); Toliara, Réserve Spéciale d’Ambohijanahary, Forêt d’Ankazotsihitafototra, 35.2 km 312° NW Ambaravaranala, 18.26667°S, 45.40667°E, 1050 m, montane rainforest, 13.–17.I.2003 (*B.L. Fisher et al.*); Toliara, Réserve Spéciale d’Ambohijanahary, Forêt d’Ankazotsihitafototra, 34.6 km 314° NW Ambaravaranala, 18.26°S, 45.41833°E, 1100 m, montane rainforest, 16.I.2003 (*B.L. Fisher et al.*); Toliara, Forêt Classée d’Analavelona, 29.2 km 343° NNW Mahaboboka, 22.675°S, 44.19°E, 1100 m, montane rainforest, 18.–22.II.2003 (*B.L. Fisher et al.*); Toliara, Forêt Classée d’Analavelona, 29.4 km 343° NNW Mahaboboka, 22.675°S, 44.18667°E, 1050 m, montane rainforest, 21.II.2003 (*B.L. Fisher et al.*); Toliara, Rés. Andohahela, 13 km NW Enakara, 24.55°S, 46.8°E, 1250 m, montane rainforest, 30.XI.1992 (*B.L. Fisher*); Toliara, Rés. Andohahela, 11 km NW Enakara, 24.5667°S, 46.83333°E, 800 m, rainforest, 17.–20.XI.1992 (*B.L. Fisher*); Toliara, Parc National d’Andohahela, Col du Sedro, 3.8 km 113° ESE Mahamavo, 37.6 km 341° NNW Tolagnaro, 24.76389°S, 46.75167°E, 900 m, montane rainforest, 21.–25.I.2002 (*B.L. Fisher et al.*); Toliara, Forêt Ivohibe 55.0 km N Tolagnaro, 24.569°S, 47.204°E, 200 m, rainforest, 2.–4.XII.2006 (*B.L. Fisher et al.*); Toliara, Réserve Spéciale Kalambatritra, Ampanihy, 23.463°S, 46.47057°E, 1269 m, montane rainforest, 10.II.2009 (*B.L. Fisher et al.*).

####### Diagnosis.

*Tetramorium proximum* can be distinguished from the other members of the species complex on the basis of the following character combination: eyes relatively large (OI 24–28); antennal scapes short (SI 74–76); frontal carinae strongly developed, noticeably raised, and reaching or ending shortly before posterior head margin; propodeal spines short to medium-sized, elongate-triangular, and usually acute (PSLI 17–21); petiolar node high rounded nodiform, in profile around 1.7 to 1.9 times higher than long (LPeI 52–60), and in dorsal view around 1.3 to 1.5 times wider than long (DPpI 135–153); mesosoma with four to nine pairs of long, fine, standing hairs, usually five or six ranging from anterior pronotum to metanotal groove.

####### Worker measurements

**(N=12).** HL 0.72–0.88 (0.78); HW 0.65–0.81 (0.72); SL 0.49–0.60 (0.54); EL 0.18–0.20 (0.19); PH 0.34–0.40 (0.37); PW 0.49–0.60 (0.54); WL 0.92–1.07 (0.98); PSL 0.12–0.18 (0.15); PTL 0.15–0.19 (0.18); PTH 0.29–0.35 (0.32); PTW 0.18–0.23 (0.21); PPL 0.19–0.23 (0.22); PPH 0.29–0.35 (0.31); PPW 0.28–0.34 (0.31); CI 89–94 (92); SI 74–76 (75); OI 24–28 (26); DMI 53–58 (55); LMI 37–40 (38); PSLI 17–21 (19); PeNI 37–42 (39); LPeI 52–60 (56); DPeI 111–130 (120); PpNI 52–61 (57); LPpI 65–74 (69); DPpI 135–153 (145); PPI 135–160 (148).

####### Worker description.

Head much longer than wide (CI 89–94); posterior head margin weakly concave. Anterior clypeal margin with distinct median impression. Frontal carinae strongly developed, noticeably raised, diverging posteriorly, and either reaching or ending shortly before posterior head margin. Antennal scrobes present and distinct but weak, shallow and without clear and distinct posterior and ventral margins. Antennal scapes short, not reaching posterior head margin (SI 74–76). Eyes relatively large (24–28). Mesosomal outline in profile flat to weakly convex, relatively low and long (LMI 37–40), moderately marginate from lateral to dorsal mesosoma; promesonotal suture absent; metanotal groove weakly to moderately developed. Propodeal spines short to medium-sized, elongate-triangular, and usually acute (PSLI 17–21), propodeal lobes short, triangular, and blunt or acute, always much shorter than propodeal spines. Petiolar node in profile high nodiform with well-rounded antero- and posterodorsal margins and strongly convex dorsum, around 1.7 to 1.9 times higher than long (LPeI 52–60), anterior and posterior faces approximately parallel, antero- and posterodorsal margins situated at about same height; petiolar node in dorsal view around 1.1 to 1.3 times wider than long (DPeI 111–130), in dorsal view pronotum around 2.4 to 2.7 times wider than petiolar node (PeNI 37–42). Postpetiole in profile subglobular and weakly anteroposteriorly compressed, around 1.4 to 1.5 times higher than long (LPpI 65–74); in dorsal view around 1.3 to 1.5 times wider than long (DPpI 135–153), pronotum around 1.7 to 1.9 times wider than postpetiole (PpNI 52–61). Postpetiole in profile appearing approximately of same volume as petiolar node, and both nodes of approximately similar height; postpetiole in dorsal view between 1.3 to 1.6 times wider than petiolar node (PPI 135–160). Mandibles completely unsculptured, smooth, and shiny; clypeus longitudinally rugose with usually three to five rugae, median ruga usually distinct and fully developed, sometimes partly reduced or broken, one or two weaker rugae present on each side; cephalic dorsum between frontal carinae longitudinally rugose with six to nine rugae, rugae running from posterior clypeal margin to posterior head margin, but often interrupted, splitting up or with cross-meshes; scrobal area mostly unsculptured; lateral head reticulate-rugose to longitudinally rugose. Ground sculpture on head weakly to moderately punctate. Dorsum of mesosoma mostly reticulate-rugose with smaller proportions of irregularly longitudinally rugose sculpture medially; lateral mesosoma mostly irregularly longitudinally rugose. Forecoxae mostly unsculptured, smooth, and shining, sometimes with traces of ground sculpture. Ground sculpture on mesosoma usually weak to absent. Both waist segments and gaster fully unsculptured, smooth, and shining. Dorsum of head with several pairs of long, fine, standing hairs; mesosoma with four to nine pairs of long, fine, standing hairs, usually with five or six ranging from anterior pronotum to metanotal groove, rarely propodeum with one pair anteriorly; waist segments and first gastral tergite without any standing hairs; appressed pubescence on first gastral tergite highly variable: ranging from short to very short and relatively scarce to relatively long and dense. Anterior edges of antennal scapes and dorsal (outer) surfaces of hind tibiae usually with appressed, rarely decumbent, hairs. Head, mesosoma, waist segments, and gaster light brown to reddish brown, rarely of darker brown; mandibles, antennae, and legs lighter, usually light yellowish brown.

####### Distribution and biology.

*Tetramorium proximum* has a very broad distribution in Madagascar, and is indeed found in most sampled rainforests or montane rainforests at elevations from 25 to 1680 m (Fig. 64). The southernmost locality is Andohahela in the southeast; from there the species ranges with few interruptions up to Montagne d’Ambre at the northern tip of Madagascar. In addition to the eastern and northern forest belt, *Tetramorium proximum* is also found in the few isolated humid forests located further west, such as Analavelona, Isalo, and Ambohijanahary. *Tetramorium proximum* appears to live in leaf litter.

####### Discussion.

*Tetramorium proximum* is a very distinct species within the species complex. Due to its large body size (HW 0.65–0.81; WL 0.92–1.07) and strongly developed frontal carinae it cannot be mistaken for the five smaller species *Tetramorium aspis*, *Tetramorium camelliae*, *Tetramorium cognatum*, *Tetramorium karthala*, and *Tetramorium rumo* (HW 0.43–0.57; WL 0.56–0.81), all of which have weakly to moderately developed frontal carinae. Nor can *Tetramorium proximum* with its larger eyes (OI 24–28) and its long, standing pilosity on the dorsal mesosoma be confused with the smaller-eyed *Tetramorium gladius* (OI 19–20) or *Tetramorium freya*, which lacks any standing pilosity on the mesosoma. The remaining two species, *Tetramorium myrmidon* and *Tetramorium tenuinode*, are both morphologically close and relatively similar to *Tetramorium proximum*. The separation of these three requires more attention.

*Tetramorium tenuinode*, despite sharing a superficially similar habitus, differs from *Tetramorium proximum* in a number of characters, and co-occurs with the latter species throughout most of its distribution range while both species maintain their species-specific differences. The main diagnostic character is the pilosity on the dorsal mesosoma, which consists of two pairs of long, standing hairs (one on anterior pronotum and one on anterior mesonotum) in *Tetramorium tenuinode*, while in *Tetramorium proximum* there are usually five to six pairs (rarely four or more than six) from anterior pronotum to posterior mesonotum. Furthermore, *Tetramorium proximum* has significantly longer antennal scapes (SI 74–77) than *Tetramorium tenuinode* (SI 66–70), and is normally a darker colour. Also, *Tetramorium tenuinode* generally has a thinner petiolar node in lateral view that is around 1.8 to 2.2 times higher than long (LPeI 45–54), while the node of *Tetramorium proximum* is around 1.7 to 1.9 times higher than long (LPeI 52–60). There is some overlap in these morphometric ranges, but there is a strong general trend towards a much thinner node in *Tetramorium tenuinode*, which together with the other characters mentioned above works very well in distinguishing both species. The remaining species, *Tetramorium myrmidon*, might be confused with *Tetramorium proximum* since they share most morphological characters and their morphometric ranges overlap. Nevertheless, *Tetramorium myrmidon* is only found in Ambohijanahary where it co-occurs with *Tetramorium proximum*, and there they are both easily separable based on mesosomal pilosity (two pairs in *Tetramorium myrmidon* vs. five or six in *Tetramorium proximum*) and petiolar node height.

*Tetramorium proximum* is a relatively variable species with respect to certain characters. The greatest variation can be seen in gastral pubescence, which is always strongly appressed, but can vary from very short and scarce to relatively long and dense. Often this variation can be observed within the same locality or series, sometimes all specimens from one locality have short pubescence while populations from other localities have long pubescence. However, it seems that there is also a geographical component to this variation. The material from the southeast has predominantly long and dense pubescence, and the form with short pubescence is relatively rare, but still present. The material from central eastern Madagascar is a well-balanced mix of short and long pubescence. Interestingly, the material from the northeast, north, and the isolated forests in the west possesses almost exclusively short and scarce pubescence. Another, even though less variable character, is the shape of the petiolar node, which is high nodiform with a rounded dorsum, but can vary in height or thickness from population to population. Furthermore, some populations have fewer or more pairs of long, standing hairs on the mesosomal dorsum. The most common count is five to six pairs ranging throughout most of the distribution range, but in some localities one can see only four pairs while in others six to seven, and in a few series seven to eight pairs are common. The variation observed in *Tetramorium proximum* is not surprising, however, due to its wide distribution and commonness. Compared to other widely distributed and common Malagasy or Afrotropical *Tetramorium* species, this species actually shows much less variation.

###### Tetramorium rumo

Hita Garcia & Fisher sp. n.

http://zoobank.org/555CD002-455A-4AE0-A404-99F38EEFF755

http://species-id.net/wiki/Tetramorium_rumo

**Figure 38.**
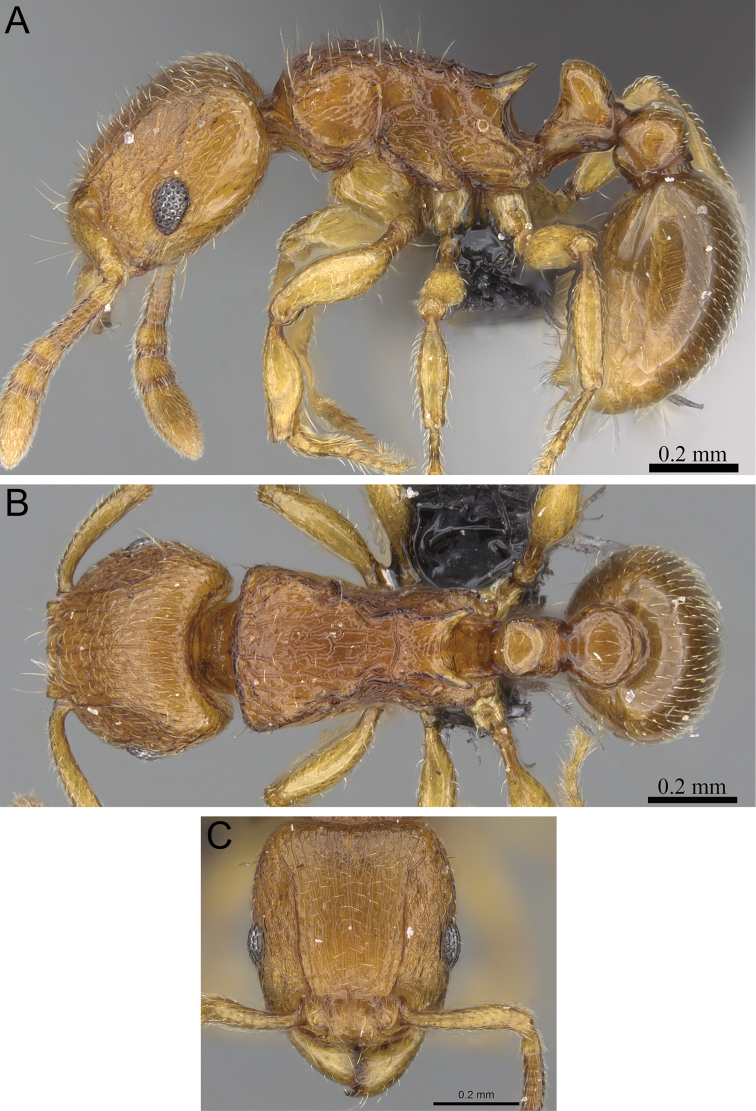
*Tetramorium rumo* holotype worker (CASENT0073025). **A** Body in profile **B** Body in dorsal view **C** Head in full-face view.

Figs 22B , 23D , 24D , 27A , 38 , 64

####### Type material.

**Holotype**, pinned worker, MADAGASCAR, Fianarantsoa, Réserve Speciale Manombo 24.5 km 228° Farafangana, 23.01583°S, 47.719°E, 30 m, lowland rainforest, collection code BLF13963, 20.IV.2006 (*B.L. Fisher et al.*) (CAS: CASENT0073025). **Paratypes**, seven pinned workers with same data as holotype (BMNH: CASENT0071823; CAS: CASENT0071827; CASENT0072469; CASENT0073028; CASENT0073033; CASENT0073038; CASENT0073039).

####### Non-type material.

MADAGASCAR: Fianarantsoa, Réserve Forestière d’Agnalazaha, Mahabo, 42.9 km 215° Farafangana, 23.19383°S, 47.723°E, 20 m, littoral rainforest, 19.IV.2006 (*B.L. Fisher et al.*); Fianarantsoa, P.N. Ranomafana, Tolongoina-Ampasimpotsy 3, 21.47412°S, 47.55742°E, 520 m, stomach contents of *Mantella bernhardi* Vences *et al.*, 11.IV.2003 (*V.C. Clark*); Fianarantsoa, Forêt de Vevembe, 66.6 km 293° Farafangana, 22.791°S, 47.18183°E, 600 m, rainforest, transition to montane forest, 23.–24.IV.2006 (*B.L. Fisher et al.*); Toamasina, 6 km ESE Andasibe (=Perinet), 18.95°S, 48.46667°E, 900 m, rainforest, 17.XI.1990 (*P.S. Ward*); Toamasina, F.C. Andriantantely, 18.695°S, 48.81333°E, 530 m, rainforest, 7.XII.1998 (*H.J. Ratsirarson*); Toamasina, Ankerana, 18.4061S, 48.82029°E, 725 m, rainforest, 16.–21.I.2012 (*B.L. Fisher et al.*); Toamasina, Ankerana, 18.40829°S, 48.82107°E, 750 m, rainforest, 21.–26.I.2012 (*B.L. Fisher et al.*); Toamasina, Reserve Betampona, Camp Vohitsivalana, 37.1 km 338° Toamasina, 17.88667°S, 49.2025°E, 520 m, rainforest, 1.–3.XII.2005 (*B.L. Fisher et al.*); Toamasina, F.C. Sandranantitra, 18.04833°S, 49.09167°E, 450 m, rainforest, 21.I.1999 (*H.J. Ratsirarson*); Toliara, Rés. Andohahela, 10 km NW Enakara, 24.56667°S, 46.81667°E, 420–430 m, rainforest, 15.–22.XI.1992 (*B.L. Fisher*); Toliara, Rés. Andohahela, 13 km NW Enakara, 24.55°S, 46.8°E, 1250 m, montane rainforest, 30.XI.1992 (*B.L. Fisher*); Toliara, Rés. Andohahela, 6 km SSW Eminiminy, 24.73333°S, 46.8°E, 330 m, rainforest, 4.II.1993 (*P.S. Ward*); Toliara, Parc National Andohahela, Col de Tanatana, 33.3 km NW Tolagnaro, 24.7585°S, 46.85367°E, 275 m, rainforest, 28.XI.2006 (*B.L. Fisher et al.*); Toliara, Andohahela, 24.77639°S, 46.70528°E, 320 m, 9.XII.2007 (*A. Ballerio*); Toliara, Forêt Ivohibe, 55.0 km N Tolagnaro, 24.569°S, 47.204°E, 200 m, rainforest, 2.–4.XII.2006 (*B.L. Fisher et al.*).

####### Diagnosis.

*Tetramorium rumo* can be well recognised within the *Tetramorium cognatum* species complex on the basis of the following character combination: very large eyes (OI 28–31); antennal scapes very short (SI 60–66); propodeal spines moderately long, elongate-triangular to spinose, and usually acute (PSLI 22–26); propodeal spines and lobes not strongly inclined towards each other; petiolar node thinly cuneiform and moderately squamiform, in profile around 2.3 to 2.7 times higher than long (LPeI 37–43), in dorsal view between 1.5 to 1.7 times wider than long (DPeI 156–171); mesosoma either with at least seven pairs of long, standing hairs on pronotum and mesonotum and propodeum sometimes with one pair anteriorly, or with just two pairs of long, standing hairs, one on anterior pronotum and one on anterior mesonotum.

####### Worker measurements

**(N=12).** HL 0.47–0.55 (0.51); HW 0.43–0.49 (0.45); SL 0.26–0.32 (0.29); EL 0.12–0.14 (0.13); PH 0.21–0.24 (0.23); PW 0.34–0.39 (0.36); WL 0.56–0.67 (0.60); PSL 0.11–0.13 (0.12); PTL 0.07–0.09 (0.08); PTH 0.19–0.23 (0.21); PTW 0.12–0.15 (0.14); PPL 0.12–0.15 (0.13); PPH 0.17–0.20 (0.19); PPW 0.18–0.22 (0.20); CI 88–91 (90); SI 60–66 (64); OI 28–31 (29); DMI 58–62 (60); LMI 36–38 (38); PSLI 22–26 (23); PeNI 35–39 (38); LPeI 37–43 (41); DPeI 156–171 (162); PpNI 51–56 (54); LPpI 68–75 (72); DPpI 138–152 (146); PPI 141–152 (145).

####### Worker description.

Head much longer than wide (CI 88–91); in full-face view posterior head margin weakly concave. Anterior clypeal margin with distinct median impression. Frontal carinae very weakly developed, only feebly raised, usually ending shortly after posterior eye margin or merging with cephalic sculpture halfway between posterior eye margin and posterior head margin. Antennal scrobes very weak to absent, very shallow and without clear and distinct posterior and ventral margins. Antennal scapes very short, not reaching posterior head margin (SI 60–66). Eyes very large (OI 28–31). Mesosomal outline in profile flat to weakly convex, comparatively low and long (LMI 36–38), moderately marginate from lateral to dorsal mesosoma; promesonotal suture absent; metanotal groove very weak to absent. Propodeal spines moderately long, elongate-triangular to spinose, and usually acute (PSLI 22–26), propodeal lobes triangular and short, always much shorter than propodeal spines, in profile spines and lobes not strongly inclined towards each other. Petiolar node thinly cuneiform and moderately squamiform, in profile around 2.3 to 2.7 times higher than long (LPeI 37–43), anterior and posterior faces not parallel, anterodorsal margin usually situated higher and more strongly angled than posterodorsal margin, petiolar dorsum relatively flat to weakly convex and tapering backwards posteriorly; node in dorsal view between 1.5 to 1.7 times wider than long (DPeI 156–171), in dorsal view pronotum around 2.6 to 2.8 times wider than petiolar node (PeNI 35–39). Postpetiole in profile globular, between 1.3 to 1.5 times higher than long (LPpI 68–75); in dorsal view around 1.4 to 1.5 times wider than long (DPpI 138–152), pronotum around 1.8 to 1.9 times wider than postpetiole (PpNI 51–56). Postpetiole in profile appearing shorter and thicker than petiolar node, postpetiole in dorsal view around 1.4 to 1.5 times wider than petiolar node (PPI 141–152). Mandibles completely unsculptured, smooth, and shiny; clypeus weakly irregularly longitudinally rugulose, median rugula usually present but rarely fully developed, one or two mostly broken lateral rugulae present on each side; cephalic dorsum between frontal carinae longitudinally rugulose with eight to eleven fine rugulae, rugulae usually running from posterior clypeal margin to posterior head margin, often irregularly shaped, interrupted or with cross-meshes; scrobal area mostly unsculptured; lateral head mainly longitudinally rugulose to reticulate-rugulose, but larger areas often only weakly sculptured and appearing fairly smooth and shiny. Ground sculpture on head variable, ranging from weakly developed or absent to moderately punctate. Dorsum of mesosoma mostly longitudinally rugulose; lateral mesosoma mostly unsculptured with smaller, irregularly longitudinally rugulose or reticulate-rugulose areas. Ground sculpture on mesosoma very weak to absent. Forecoxae usually unsculptured, smooth, and shining. Both waist segments and gaster fully unsculptured, smooth, and shining. Dorsum of head with several pairs of long, fine, standing hairs; pilosity on dorsal mesosoma variable: southern populations with at least seven pairs on pronotum and mesonotum, propodeum sometimes with one pair anteriorly, and northern populations with only two pairs, one on anterior pronotum and one on anterior mesonotum; waist segments and first gastral tergite without any standing hairs; first gastral tergite with short, dense, appressed (rarely decumbent) pubescence. Anterior edges of antennal scapes and dorsal (outer) surfaces of hind tibiae with appressed to decumbent hairs. Body usually uniformly whitish yellow to light brown, very rarely darker.

####### Etymology.

The new species is named after the fictional character “Rumo” from Walter Moers’ fantasy novel “Rumo and His Miraculous Adventures”. *Tetramorium rumo* is a very bright species, almost white, with distinct propodeal spines reminiscent of “Rumo”, who is a white wolperting with short but acute horns. The species epithet is an arbitrary combination of letter, thus invariable.

####### Distribution and biology.

The new species is another rainforest inhabitant mainly found in eastern Madagascar (Fig. 64). However, the distribution range is somewhat unusual since *Tetramorium rumo* is found in the southeast from Andohahela to Ranomafana, and then much further north from Perinet to Betampona. The reasons for this patchy distribution are unclear. *Tetramorium rumo* was mainly sampled in rainforests, rarely montane rainforests or littoral rainforests, at elevations ranging from 20 to 1250 m. In addition, *Tetramorium rumo* appears to live in leaf litter.

####### Discussion.

*Tetramorium rumo* is very unlikely to be confused with the much larger species *Tetramorium freya*, *Tetramorium gladius*, *Tetramorium myrmidon*, *Tetramorium proximum*, and *Tetramorium tenuinode*. All the latter species, except *Tetramorium freya*, also have very well developed frontal carinae, which contrast with the reduced and very weak frontal carinae seen in *Tetramorium rumo*. *Tetramorium freya* has weaker frontal carinae than the other four species, but in contrast to *Tetramorium rumo* this species does not have any standing pilosity on the mesosomal dorsum. Furthermore, *Tetramorium gladius* has very small eyes (OI 19–20) compared to *Tetramorium rumo*, which has very large eyes (OI 28–31). The remaining four species of the *Tetramorium cognatum* complex are morphologically much closer to *Tetramorium rumo* than the five species mentioned above. However, *Tetramorium camelliae* is easily separable from *Tetramorium rumo* on the basis of the petiolar node shape, which is strongly squamiform and transverse in the former (LPeI 33–36; DPeI 228–238), contrasting with the highly nodiform to thinly cuneiform node of the latter (LPeI 37–43; DPeI 156–171). *Tetramorium aspis* and *Tetramorium karthala* both have longer antennal scapes (SI 68–74), shorter propodeal spines (PSLI 18–22), thicker and lower petiolar nodes in profile (LPeI 46–54), and less transverse nodes in dorsal view (DPeI 146–161) compared to *Tetramorium rumo* (SI 60–66; LPeI 37–43; DPeI 156–171). In addition, *Tetramorium aspis* has propodeal spines and lobes strongly inclined towards each other, an arrangement absent in *Tetramorium rumo*, and *Tetramorium karthala* is only found on the Comorian island of Grand Comore while *Tetramorium rumo* is distributed in Madagascar.

The last species of the *Tetramorium cognatum* complex, *Tetramorium cognatum* itself, is probably the closest relative of *Tetramorium rumo* within the complex, and without careful examination they could be confused in some cases. However, both differ clearly in propodeal spine length and petiolar node shape. *Tetramorium rumo* has relatively long and spinose propodeal spines (PSLI 22–26) which contrast with the much more reduced, short, and triangular teeth of *Tetramorium cognatum* (PSLI 12–16). Also, the thinly cuneiform petiolar node of *Tetramorium rumo*, which is 2.3 to 2.7 times higher than long (LPeI 37–43) and around 1.6 to 1.7 times wider than long (DPeI 156–171), discriminates it from *Tetramorium cognatum*, in which the node is 1.8 to 2.0 times higher than long (LPeI 49–55) and around 1.3 to 1.4 times wider than long (DPeI 129–142).

Nonetheless, the species most similar to *Tetramorium rumo* is likely *Tetramorium rala* from the *Tetramorium schaufussi* complex. Both species share a very similar habitus. Compared to all other species of the *Tetramorium schaufussi* species group, they are smaller in size, have relatively longer propodeal spines, a high nodiform to thinly cuneiform petiolar node, lack standing pilosity on the waist segments, and possess very bright body colouration. We have placed them in different species complexes on the basis of the presence (*Tetramorium rala*) or absence (*Tetramorium rumo*) of standing pilosity on the first gastral tergite, but otherwise these two species could be easily confused. However, despite the very strong similarities, substantial differences separate these species from each other. Most obviously, the petiolar node of *Tetramorium rumo* is thinner and stronger anteroposteriorly compressed, in profile around 2.3 to 2.7 times higher than long (LPeI 37–43), and in dorsal view between 1.5 to 1.7 times wider than long (DPeI 156–171). By contrast, *Tetramorium rala* has a node in profile is 2.0 to 2.2 times higher than long (LPeI 45–50), and in dorsal view around 1.3 to 1.5 times broader than long (DPeI 129–145). Also, *Tetramorium rumo* has larger eyes (OI 28–31) than *Tetramorium rala* (OI 26–28). In addition, their distribution ranges strongly overlap in central-eastern Madagascar and both species maintain their species identities without intermediate forms.

As mentioned above, there is some noteworthy variation in mesosomal pilosity in *Tetramorium rumo*. The populations in the southeast from Andohahela to Vevembe and the type locality Manombo all have at least seven pairs of long, standing hairs on the pronotum and mesonotum and the propodeum sometimes has one pair anteriorly, whereas the populations further north from Perinet to Betampona all have just two pairs of long, standing hairs (one on the anterior pronotum and one on the anterior mesonotum). Nevertheless, since both population groups are widely separated geographically and other character divides them, we consider this a clear case of geographical variation.

###### Tetramorium tenuinode

Hita Garcia & Fisher sp. n.

http://zoobank.org/4856B8A4-FBBA-4629-8619-21F62101BAA7

http://species-id.net/wiki/Tetramorium_tenuinode

**Figure 39.**
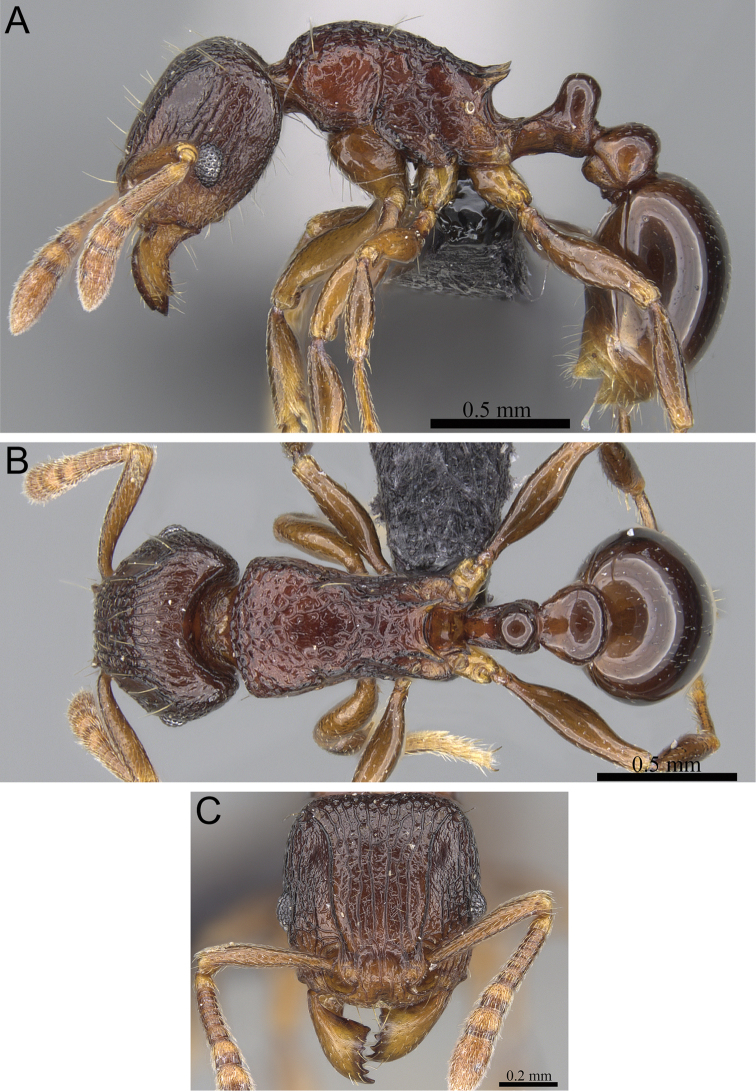
*Tetramorium tenuinode* holotype worker (CASENT0040115). **A** Body in profile **B** Body in dorsal view **C** Head in full-face view.

Figs 24B , 25C , 26A , 26B , 39 , 64

####### Type material.

**Holotype**, pinned worker, MADAGASCAR, Fianarantsoa, Parc National de Ranomafana, Vatoharanana River, 4.1 km 231° SW Ranomafana, 21.29°S, 47.43333°E, 1100 m, montane rainforest, sifted litter (leaf mold, rotten wood), collection code BLF08400, 27.–31.III.2003 (*B.L. Fisher et al.*) (CAS: CASENT0040115). **Paratypes**, 41 pinned workers with same data as holotype (CAS: CASENT0039644; CASENT0039646; CASENT0039651; CASENT0039655; CASENT0039660; CASENT0039664; CASENT0039668; CASENT0039671; CASENT0039673; CASENT0039737; CASENT0039739; CASENT0039745; CASENT0039749; CASENT0039750; CASENT0039754; CASENT0039759; CASENT0039761; CASENT0039809; CASENT0040000; CASENT0040020; CASENT0040023; CASENT0040035; CASENT0040036; CASENT0040090; CASENT0040092; CASENT0040096; CASENT0040099; CASENT0040105; CASENT0040106; CASENT0040112; CASENT0040123; CASENT0040181; CASENT0040279; CASENT0040284; CASENT0040285; CASENT0040293; CASENT0040296; CASENT0040304; CASENT0040306; CASENT0040311; CASENT0040317); and nine pinned workers with same data as holotype except collected ex rotten log and collection code BLF08488 (BMNH: CASENT0497623; CAS: CASENT0497621; MCZ: CASENT0497622).

####### Non-type material.

MADAGASCAR: Antsiranana, Forêt Ambanitaza, 26.1 km 347° Antalaha, 14.67933°S, 50.18367°E, 240 m, rainforest, 27.XI.1994 (*B.L. Fisher*); Antsiranana, 1 km W Andampibe, Cap Masoala, 15.69361°S, 50.18139°E, 125 m, lowland rainforest, 1.XII.1993 (*G.D. Alpert*); Fianarantsoa, 45 km S Ambalavao, 22.21667°S, 47.01667°E, 785 m, rainforest, 25.IX.1993 (*B.L. Fisher*); Fianarantsoa, 45 km S Ambalavao, 22.21667°S, 47.01667°E, 720 m, rainforest edge, 31.X.1993 (*B.L. Fisher*); Fianarantsoa, 2 km W Andrambovato, along river Tatamaly, 21.51167°S, 47.41°E, 1075 m, montane rainforest, 3.–5.VI.2005 (*B.L. Fisher et al.*); Fianarantsoa, Rés. Andringitra, 43 km S Ambalavao, 22.23333°S, 47°E, 825 m, rainforest, 5.X.1993 (*B.L. Fisher*); Fianarantsoa, R.S. Ivohibe, 7.5 km ENE Ivohibe, 22.47°S, 46.96°E, 900 m, rainforest, 7.–12.X.1997 (*B.L. Fisher*); Fianarantsoa, R.S. Ivohibe, 8.0 km E Ivohibe, 22.48333°S, 46.96833°E, 1200 m, montane rainforest, 15.–21.X.1997 (*B.L. Fisher*); Fianarantsoa, 9.0 km NE Ivohibe, 22.42667°S, 46.93833°E, 900 m, rainforest, 17.XI.1997 (*B.L. Fisher*); Fianarantsoa, Réserve Speciale Manombo 24.5 km 228° Farafangana, 23.01583°S, 47.719°E, 30 m, lowland rainforest, 20.IV.2006 (*B.L. Fisher et al.*); Fianarantsoa, Ranomafana Nat. Park, Maharira forest, 21°S, 47°E, 1190 m, montane forest, 11.X.1992 (*E. Rajeriarison*); Fianarantsoa, Parc National de Ranomafana, Vatoharanana River, 4.1 km 231° SW Ranomafana, 21.29°S, 47.43333°E, 1100 m, montane rainforest, 27.–31.III.2003 (*B.L. Fisher et al.*); Fianarantsoa, Forêt de Vevembe, 66.6 km 293° Farafangana, 22.791°S, 47.18183°E, 600 m, rainforest, transition to montane forest, 23.IV.2006 (*B.L. Fisher et al.*); Toamasina, Montagne d’Akirindro 7.6 km 341° NNW Ambinanitelo, 15.28833°S, 49.54833°E, 600 m, rainforest, 17.–21.III.2003 (*B.L. Fisher et al.*); Toamasina, 6.3 km S Ambanizana, Andranobe, 15.6813°S, 49.958°E, 25 m, rainforest, 14.XI.1993 (*B.L. Fisher*); Toamasina, Réserve Spéciale Ambatovaky, Sandrangato river, 16.77274°S, 49.26551°E, 450 m, rainforest, 20.–22.II.2010 (*B.L. Fisher et al.*); Toamasina, Réserve Spéciale Ambatovaky, Sandrangato river, 16.7633°S, 49.26692°E, 520 m, rainforest, 22.–24.II.2010 (*B.L. Fisher et al.*); Toamasina, Réserve Spéciale Ambatovaky, Sandrangato river, 16.81753°S, 49.29498°E, 360 m, rainforest, 25.–27.II.2010 (*B.L. Fisher et al.*); Toamasina, Forêt Ambatovy, 14.3 km 57° Moramanga, 18.85083°S, 48.32°E, 1075 m, montane rainforest, 21.III.2004 (*Malagasy ant team*); Toamasina, Ambatovy, 12.4 km NE Moramanga, 18.83937°S, 48.30842°E, 1080 m, montane rainforest, 4.–8.III.2007 (*B.L. Fisher et al.*); Toamasina, Analamay, 18.80623°S, 48.33707°E, 1068 m, montane rainforest, 21.III.2004 (*Malagasy ant team*); Toamasina, Corridor Forestier Analamay-Mantadia, Ambatoharanana, 18.80388°S, 48.40506°E, 1013 m, rainforest, 12.–19.XII.2012 (*B.L.Fisher et al.*); Toamasina, Corridor Forestier Analamay-Mantadia, Ambohibolakely, 18.76087°S, 48.37128°E, 1044 m, rainforest, 23.–28.XI.2012 (*B.L.Fisher et al.*); Toamasina, Corridor Forestier Analamay-Mantadia, Ambohibolakely, 18.77898°S, 48.36375°E, 918 m, rainforest, 23.–28.XI.2012 (*B.L.Fisher et al.*); Toamasina, Corridor Forestier Analamay-Mantadia, Ambohibolakely, 18.76131°S, 48.36437°E, 983 m, rainforest, 26.XI.2012 (*B.L.Fisher et al.*); Toamasina, Corridor Forestier Analamay-Mantadia, Tsaravoniana, 18.76124°S, 48.42134°E, 939 m, rainforest, 2.–7.XII.2012 (*B.L.Fisher et al.*); Toamasina, Corridor Forestier Analamay-Mantadia, Tsaravoniana, 18.76465°S, 48.41938°E, 1039 m, rainforest, 2.–7.XII.2012 (*B.L.Fisher et al.*); Toamasina, Station Forestière Analamazaotra, Analamazaotra 1.3 km S Andasibe, 18.38466°S, 48.41271°E, 980 m, montane rainforest, 11.–13.XII.2007 (*B.L. Fisher et al.*); Toamasina, F.C. Andriantantely, 18.695°S, 48.81333°E, 530 m, rainforest, 4.–7.XII.1998 (*H.J. Ratsirarson*); Toamasina, Montagne d’Anjanaharibe, 18.0 km 21° NNE Ambinanitelo, 15.18833°S, 49.615°E, 470 m, rainforest, 8.–12.III.2003 (*B.L. Fisher et al.*); Toamasina, Ankerana, 18.40829°S, 48.82107°E, 750 m, rainforest, 21.–26.I.2012 (*B.L. Fisher et al.*); Toamasina, Ankerana, 18.4104°S, 48.8189°E, 855 m, rainforest, 22.–27.I.2012 (*B.L. Fisher et al.*); Toamasina, Reserve Betampona, Camp Vohitsivalana, 37.1 km 338° Toamasina, 17.88667°S, 49.2025°E, 520 m, rainforest, 1.–3.XII.2005 (*B.L.Fisher et al.*); Toamasina, Reserve Betampona, Camp Rendrirendry, 34.1 km 332° Toamasina, 17.924°S, 49.19967°E, 390 m, rainforest, 28.XI.2005 (*B.L.Fisher et al.*); Toamasina, F.C. Didy, 18.19833°S, 48.57833°E, 960 m, rainforest, 16.–23.XII.1998 (*H.J. Ratsirarson*); Toamasina, Parc National Mananara-Nord, 7.1 km 261° Antanambe, 16.455°S, 49.7875°E, 225 m, rainforest, 14.XI.2005 (*B.L. Fisher et al.*); Toamasina, 19 km ESE Maroantsetra, 15.48333°S, 49.9°E, 350 m, rainforest, 22.IV.1989 (*P.S. Ward*); Toamasina, 16 km S Moramanga, 19.08333°S, 48.23333°E, 950 m, rainforest, 18.XI.1990 (*P.S. Ward*); Toamasina, Moramanga, 18.94417°S, 48.23067°E, 922 m, urban/garden, 14.II.2007 (*B.L. Fisher et al.*); Toamasina, F.C. Sandranantitra, 18.04833°S, 49.09167°E, 450 m, rainforest, 18.–24.I.1999 (*H.J. Ratsirarson*); Toamasina, Parc National de Zahamena, Onibe River, 17.75908°S, 48.85468°E, 780 m, rainforest, 21.–23.II.2009 (*B.L. Fisher et al.*); Toamasina, Parc National de Zahamena, Tetezambatana forest, near junction of Nosivola and Manakambahiny rivers, 17.74298°S, 48.72936°E, 860 m, rainforest, 18.–19.II.2009 (*B.L. Fisher et al.*); Toliara, Rés. Andohahela, 10 km NW Enakara, 24.56667°S, 46.81667°E, 430 m, rainforest, 22.XI.1992 (*B.L. Fisher*); Toliara, Rés. Andohahela, 6 km SSW Eminiminy, 24.73333°S, 46.8°E, 330 m, rainforest, 4.II.1993 (*G.D. Alpert & P.S. Ward*); Toliara, Parc National d’Andohahela, Col du Sedro, 3.8 km 113° ESE Mahamavo, 37.6 km 341° NNW Tolagnaro, 24.76389°S, 46.75167°E, 900 m, montane rainforest, 21.–25.I.2002 (*B.L. Fisher et al.*); Toliara, Andohahela, 24.77639°S, 46.70528°E, 320 m, 9.XII.2007 (*A. Ballerio*); Toliara, Grand Lavasoa, 25.9 km W Tolagnaro, 25.08767°S, 46.749°E, 450 m, rainforest, 30.XI.-2.XII.2006 (*B.L. Fisher et al.*).

####### Diagnosis.

The following character set clearly distinguishes *Tetramorium tenuinode* from the remainder of the *Tetramorium cognatum* species complex: eyes relatively large (OI 25–27); antennal scapes very short (SI 66–70); frontal carinae well developed, noticeably raised, and approaching or ending at posterior head margin; petiolar node high rounded nodiform, in profile around 1.8 to 2.2 times higher than long (LPeI 45–54), in dorsal view around 1.3 to 1.4 times wider than long (DPeI 125–143); mesosoma with only two pairs of long, standing hairs, one on anterior pronotum and one on anterior mesonotum.

####### Worker measurements

**(N=12).** HL 0.62–0.75 (0.71); HW 0.58–0.70 (0.66); SL 0.40–0.47 (0.45); EL 0.16–0.18 (0.17); PH 0.30–0.36 (0.34); PW 0.42–0.54 (0.50); WL 0.76–0.94 (0.88); PSL 0.11–0.18 (0.14); PTL 0.13–0.17 (0.15); PTH 0.26–0.32 (0.30); PTW 0.16–0.21 (0.20); PPL 0.15–0.22 (0.20); PPH 0.25–0.32 (0.30); PPW 0.24–0.33 (0.30); CI 91–93 (92); SI 66–70 (68); OI 25–27 (26); DMI 55–59 (57); LMI 38–40 (39); PSLI 17–24 (20); PeNI 36–42 (39); LPeI 45–54 (49); DPeI 125–143 (133); PpNI 57–64 (61); LPpI 60–72 (67); DPpI 143–168 (153); PPI 148–167 (155).

####### Worker description.

Head longer than wide (CI 91–93); in full-face view posterior head margin weakly to moderately concave. Anterior clypeal margin with distinct median impression. Frontal carinae well developed, noticeably raised, diverging posteriorly, approaching or ending at posterior head margin. Antennal scrobes weakly developed, shallow and without clear and distinct posterior and ventral margins. Antennal scapes very short, not reaching posterior head margin (SI 66–70). Eyes relatively large (OI 25–27). Mesosomal outline in profile flat to weakly convex, moderately low and long (LMI 38–40), moderately marginate from lateral to dorsal mesosoma; promesonotal suture absent; metanotal groove very weak to absent. Propodeal spines short to moderately long, usually elongate-triangular, and acute (PSLI 17–24), propodeal lobes short and triangular, always much shorter than propodeal spines. Petiolar node in profile high rounded nodiform and relatively thin, around 1.8 to 2.2 times higher than long (LPeI 45–54), anterior and posterior faces approximately parallel, anterodorsal and posterodorsal margins usually situated at about same height and moderately rounded, petiolar dorsum distinctly convex; petiolar node in dorsal view around 1.2 to 1.4 times wider than long (DPeI 125–143), in dorsal view pronotum between 2.4 to 2.8 times wider than petiolar node (PeNI 36–42). Postpetiole in profile subglobular and weakly anteroposteriorly compressed, around 1.4 to 1.7 times higher than long (LPpI 60–72); in dorsal view between 1.4 to 1.7 times wider than long (DPpI 143–168), pronotum around 1.6 to 1.7 times wider than postpetiole (PpNI 57–64). Postpetiole in profile appearing more voluminous than petiolar node, postpetiole in dorsal view around 1.5 to 1.7 times wider than petiolar node (PPI 148–167). Mandibles completely unsculptured, smooth, and shiny; clypeus irregularly longitudinally rugose/rugulose with two to seven rugae/rugulae, median ruga rarely fully developed, usually broken, most other rugulae usually broken, sometimes merging with each other; cephalic dorsum between frontal carinae longitudinally rugose, usually with six to seven, rarely eight or nine rugae, rugae running from posterior clypeal margin to posterior head margin, often interrupted, splitting up or with cross-meshes, especially posteriorly; scrobal area partly unsculptured, but mostly merging with surrounding longitudinally rugose to reticulate-rugose sculpture present on lateral head. Ground sculpture on head weakly to moderately punctate. Dorsum and sides of mesosoma reticulate-rugose/rugulose to irregularly longitudinally rugose/rugulose, lateral pronotum sometimes only weakly sculptured and relatively smooth and shining. Forecoxae mostly unsculptured, smooth and shining, sometimes with traces of ground sculpture. Ground sculpture on mesosoma usually weak to absent. Both waist segments and gaster fully unsculptured, smooth, and shining. Dorsum of head with several pairs of long, fine, standing hairs; dorsum of mesosoma with two pairs only, one on anterior pronotum and one on anterior mesonotum; propodeum, waist segments and first gastral tergite without any standing hairs at all; first gastral tergite with very short, scarce, appressed pubescence. Anterior edges of antennal scapes and dorsal (outer) surfaces of hind tibiae usually with appressed, rarely decumbent, hairs. Head, mesosoma, waist segments and gaster usually uniformly chestnut brown to very dark brown, sometimes of lighter reddish brown; appendages usually of slightly lighter brown than remainder of body.

####### Etymology.

The name of the new species is a combination of the Latin adjective “tenuis”, meaning thin, and the Latin noun “nodus”, meaning node.

####### Distribution and biology.

*Tetramorium tenuinode* is widely distributed in eastern Madagascar (Fig. 64). It is found with few interruptions in an almost straight line from Grand Lavasoa and Andohahela in the southeast to Ambanitaza in the northeast. The new species clearly prefers rainforests and montane rainforests at elevations from 25 to 1200 m, even though it was also collected several times from guava forest and urban gardens. In addition, *Tetramorium tenuinode* appears to be a leaf litter inhabitant since almost all of the material was collected from this microhabitat.

####### Discussion.

This new species is easily identifiable within its species complex. The presence of well developed, raised, and long frontal carinae separates *Tetramorium tenuinode* from the species *Tetramorium aspis*, *Tetramorium camelliae*, *Tetramorium cognatum*, *Tetramorium karthala*, and *Tetramorium rumo*. These five species are generally also much smaller (WL 0.56–0.81) than *Tetramorium tenuinode* (WL 0.76–0.94). Also, *Tetramorium tenuinode* possesses much larger eyes (OI 25–27) than *Tetramorium gladius* (OI 19–20) and much shorter antennal scapes (SI 66–70) than *Tetramorium myrmidon* and *Tetramorium proximum* (SI 74–77). Lastly, the presence of two pairs of long, standing hairs on the dorsal mesosoma distinguishes *Tetramorium tenuinode* from *Tetramorium freya*, which lacks any standing hairs on the dorsal mesosoma. The latter species also displays weaker sculpture on the head and mesosoma than *Tetramorium tenuinode*.

It should be noted, however, that *Tetramorium tenuinode* is morphologically still very close to *Tetramorium proximum*, and on very superficial observation it is possible to confuse both. At the initial stage of the revision we considered *Tetramorium tenuinode* conspecific with *Tetramorium proximum*, a very variable species. However, after the detailed examination of more than five hundred pinned workers, it became apparent that the material consisted of these two fairly distinct species. Indeed, even though *Tetramorium proximum* is more broadly distributed than *Tetramorium tenuinode*, both co-occur in sympatry throughout most of their respective distribution ranges, and both are always very well distinguishable. The most obvious character to separate both species is pilosity on the dorsal mesosoma. In *Tetramorium tenuinode* this consists of two pairs of long, standing hairs only, one on anterior pronotum and one on anterior mesonotum, which contrasts with the usually five to six (rarely four or more than six) pairs found from anterior pronotum to posterior mesonotum in *Tetramorium proximum*. In addition to mesosomal pilosity and the shorter antennal scapes mentioned above, *Tetramorium tenuinode* usually also has a thinner petiolar node in profile, which is around 1.8 to 2.2 times higher than long (LPeI 45–54), while the node of *Tetramorium proximum* is around 1.7 to 1.9 times higher than long (LPeI 52–60). The identification key presented above might suggest that *Tetramorium tenuinode* can be easily confused with *Tetramorium myrmidon*, but this is not the case since the two species differ in a number of characters. Among others, *Tetramorium tenuinode* has a broader head (CI 91–93), shorter antennal scapes (SI 66–70), and a higher petiolar node (LPeI 45–54) than *Tetramorium myrmidon* (CI 88–91; SI 74–76; LPeI 58–60).

In contrast to the more variable *Tetramorium proximum*, *Tetramorium tenuinode* has little observed intraspecific variation. Some populations vary very slightly in propodeal spine length, thickness of the petiolar node, or body colouration.

##### *Tetramorium schaufussii* species complex

**Comments.** This complex contains ten species, which are characterised and distinguishable from the *Tetramorium cognatum* species complex by their presence of standing pilosity on the first gastral tergite. All species are endemic to Madagascar, except *Tetramorium schaufussii*, which is also known from Reunion. Also, they inhabit rainforests or montane rainforests and are only occasionally found in other habitats. The taxonomy of this species complex is challenging. Some species like *Tetramorium nassonowii*, *Tetramorium pseudogladius*, *Tetramorium rala* or *Tetramorium scutum* are relatively easily recognisable due to species-specific character states, but this is not true for the rest of the complex. *Tetramorium schaufussii* and *Tetramorium xanthogaster* are especially widespread species with high degrees of intraspecific variation, which causes a number of problems for the delimitation of species boundaries for most member of the complex. Intriguingly, only two species of this complex have very limited distribution ranges: *Tetramorium scutum* is only known from Ivohibe and *Tetramorium pseudogladius* from Zahamena. The remainder are all much more widespread.

###### Identification key to species of the *Tetramorium schaufussii* species complex (workers)

**Table:** 

1	Petiolar node relatively long and low, in profile around 1.2 to 1.4 times higher than long (LPeI 72–81) and in dorsal view between 1.0 to 1.2 times longer than wide (DPeI 87–98) (Fig. 40A) [Madagascar]	*Tetramorium nassonowii*
–	Petiolar node shorter and higher than above, in profile around 1.5 to 2.2 times higher than long (LPeI 45–67) and in dorsal view between 1.1 to 1.5 times wider than long (DPeI 109–154) (Fig. 40B, C)	2
2	Eyes relatively small (OI 20) (Fig. 41A) [Madagascar].	*Tetramorium pseudogladius*
–	Eyes usually much larger than above (OI 25–28, rarely OI 22–24) (Fig. 41A, B, C)	3
3	Propodeal spines moderate to long (PSLI 22–24) and propodeal lobes relatively well developed, spines and lobes strongly inclined towards each other (Fig. 42A) [Madagascar]	*Tetramorium scutum*
–	Character combination never as above, propodeal spines usually shorter than PSLI 18, if longer than PSLI 21, then propodeal spines and lobes not inclined towards each other (Fig. 42B, C, D)	4
4	Smaller species (HW 0.46–0.49; WL 0.58–0.64); propodeal spines relatively long (PSLI 21–25); petiolar node thinly cuneiform, in profile 2.0 to 2.2 times higher than long (LPeI 45–50); waist segments without long, standing pilosity; body colour whitish yellow to light brown (Fig. 43A) [Madagascar]	*Tetramorium rala*
–	Character combination never as above, larger species (HW 0.53–0.84; WL 0.70–1.20) with relatively short propodeal spines/teeth, which are always shorter than above (PSLI 8–18); petiolar node variably shaped but always lower than above, in profile around 1.5 to 1.9 times higher than long (LPeI 52–67); waist segments with or without long, standing pilosity; body colour variable (Fig. 43A, B, C)	5
5	Larger species (HW 0.71–0.84; WL 1.00–1.20); antennal scapes relatively long (SI 77–82); dorsum of promesonotum with seven to ten pairs of long, standing hairs; waist segments usually without any long, standing pilosity, rarely one pair present on petiole or postpetiole (Fig. 44A) [Madagascar]	*Tetramorium obiwan*
–	Character combination never as above; antennal scapes always shorter (SI 66–75); usually smaller species, but if body size in range above HW 0.70 and WL 1.00, then both waist segments with several pairs of long, standing hairs (Fig. 44B, C, D)	6
6	Dorsum of promesonotum usually with two to four pairs of long, standing hairs; propodeum and waist segments without any standing pilosity (Fig. 45A) [Madagascar]	*Tetramorium sikorae*
–	Dorsum of promesonotum always with more than four pairs of long, standing hairs; waist segments always with standing pilosity (Fig. 45B, C, D)	7
7	Bicoloured species, head and/or mesosoma very dark brown to black and strongly contrasting with yellow to light brown waist segments and gaster (rarely also mesosoma) (Fig. 46A, B) [Madagascar]	*Tetramorium xanthogaster* (in parts)
–	More or less uniformly coloured species, sometimes gaster darker than rest of body (Fig. 46C, D)	8
8	Head relatively long and thin (CI 86–90); clypeus always with well-developed and regular median longitudinal ruga (Fig. 47A, B) [Madagascar, Reunion]	*Tetramorium schaufussii*
–	Head relatively shorter and thicker (CI 92–95); clypeus usually with either median area unsculptured or traces of median ruga present but weakly developed and irregularly shaped (Fig. 47C, D), very rarely with a well-developed longitudinal ruga (Fig. 47E)	9
9	Frontal carinae relatively better developed; cephalic dorsum between frontal carinae with nine to thirteen relatively regularly shaped, mostly unbroken rugae; propodeal spines short to medium-sized (PSLI 18–22) (Fig. 48A, B) [Madagascar]	*Tetramorium monticola*
–	Frontal carinae relatively more weakly developed; cephalic dorsum between frontal carinae with six to ten relatively irregularly shaped, often meandering or broken rugulae; propodeal spines reduced to short to very short teeth/denticles (PSLI 8–16) (Fig. 48C, D)	10
10	Relatively smaller species (HW 0.54–0.69; WL 0.72–0.92); dorsum of propodeum usually with long, standing pilosity (Fig. 49A) [Madagascar]	*Tetramorium xanthogaster* (in parts)
–	Relatively larger species (HW 0.69–0.79; WL 1.01–1.10); dorsum of propodeum without any long, standing pilosity (Fig. 49B) [Madagascar]	*Tetramorium merina*

**Figure 40.**
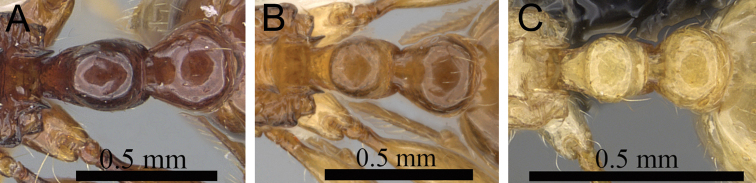
Waist segments in dorsal view. **A** *Tetramorium nassonowii* (CASENT0195504) **B** *Tetramorium merina* (CASENT0437232) **C** *Tetramorium xanthogaster* (CASENT0218041).

**Figure 41.**
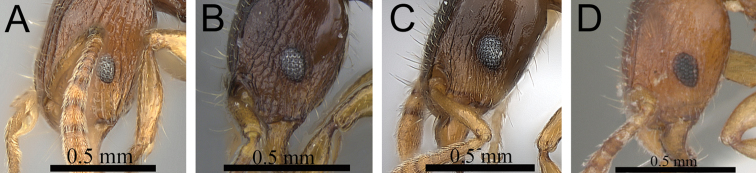
Head in profile. **A** *Tetramorium pseudogladius* (CASENT0153605) **B** *Tetramorium scutum* (CASENT0189116) **C** *Tetramorium xanthogaster* (CASENT0189134) **D** *Tetramorium sikorae* (CASENT0102402).

**Figure 42.**
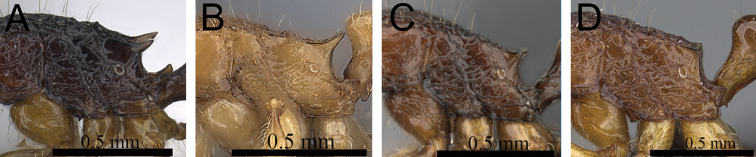
Mesosoma in profile. **A** *Tetramorium scutum* (CASENT0189116)**B** *Tetramorium monticola* (CASENT0077172) **C** *Tetramorium schaufussii* (CASENT0217760) **D** *Tetramorium merina* (CASENT0437226).

**Figure 43.**
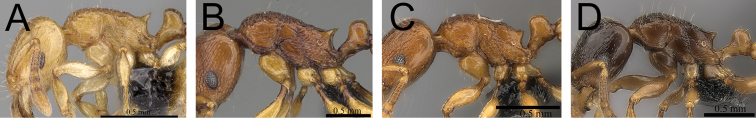
Head, mesosoma, and petiole in profile. **A** *Tetramorium rala* (CASENT0162115) **B** *Tetramorium obiwan* (CASENT0447245) **C** *Tetramorium sikorae* (CASENT0051946) **D** *Tetramorium xanthogaster* (CASENT0006330).

**Figure 44.**
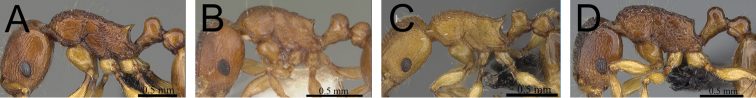
Head, mesosoma, and waist segments in profile. **A** *Tetramorium obiwan* (CASENT0447245) **B** *Tetramorium sikorae* (CASENT0102402) **C** *Tetramorium monticola* (CASENT0152401) **D** *Tetramorium merina* (CASENT0437226).

**Figure 45.**
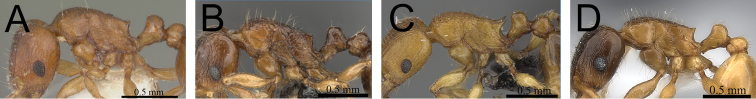
Head, mesosoma, and waist segments in profile. **A** *Tetramorium sikorae* (CASENT0102402) **B** *Tetramorium schaufussii* (CASENT0474409) **C** *Tetramorium monticola* (CASENT0152401) **D** *Tetramorium xanthogaster* (CASENT0189134).

**Figure 46.**
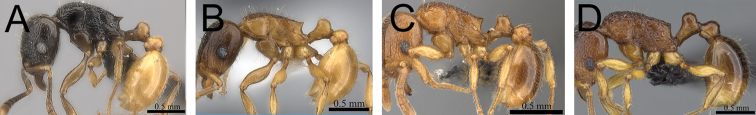
Body in profile. **A** *Tetramorium xanthogaster* (CASENT0485100) **B** *Tetramorium xanthogaster* (CASENT0189134) **C** *Tetramorium schaufussii* (CASENT0152490) **D** *Tetramorium merina* (CASENT0437226).

**Figure 47.**
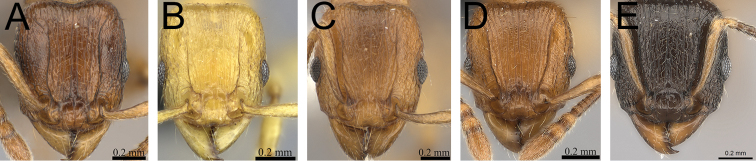
Head in full-face view. **A** *Tetramorium schaufussii* (CASENT0474409) **B** *Tetramorium schaufussii* (CASENT0497951) **C** *Tetramorium merina* (CASENT0437232) **D** *Tetramorium monticola* (CASENT0152427) **E** *Tetramorium xanthogaster* (CASENT0491748).

**Figure 48.**
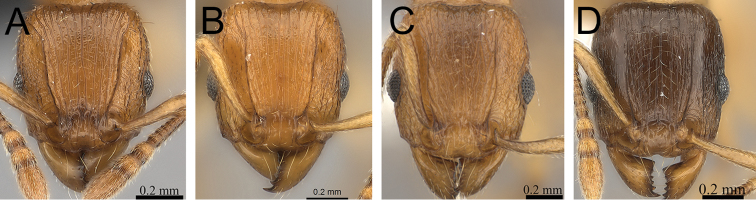
Head in full-face view. **A** *Tetramorium monticola* (CASENT0152427) **B** *Tetramorium monticola* (CASENT0497970) **C** *Tetramorium merina* (CASENT0437232) **D** *Tetramorium xanthogaster* (CASENT0189134).

**Figure 49.**
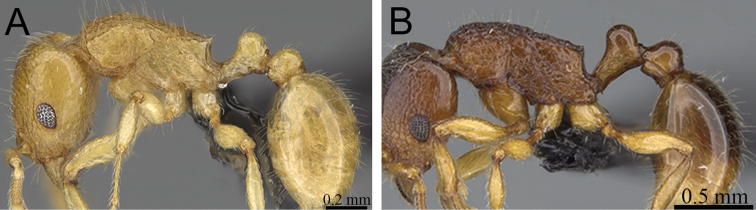
Body in profile. **A** *Tetramorium xanthogaster* (CASENT0218041) **B** *Tetramorium merina* (CASENT0437226).

###### Tetramorium merina

Hita Garcia & Fisher sp. n.

http://zoobank.org/EDBCDB39-4EDB-439C-9812-7675585170BD

http://species-id.net/wiki/Tetramorium_merina

Figs 20C , 40B , 42D , 44D , 46D , 47C , 48C , 49B , 50 , 65

####### Type material.

**Holotype**, pinned worker, MADAGASCAR, Antananarivo, Réserve Spéciale d’Ambohitantely, Forêt d’Ambohitantely, 20.9 km 72° NE Ankazobe, 18.22528°S, 47.28683°E, 1410 m, montane rainforest, ex rotten log, collection code BLF03710, 17.–22.IV.2001 (*B.L. Fisher et al.*) (CAS: CASENT0437226). **Paratypes**, 13 pinned workers with same data as holotype (BMNH: CASENT0437223; CAS: CASENT0437222; CASENT0437228; CASENT0437229; CASENT0437230; CASENT0437231; CASENT0437232; CASENT0437233; MCZ: CASENT0437225).

**Figure 50.**
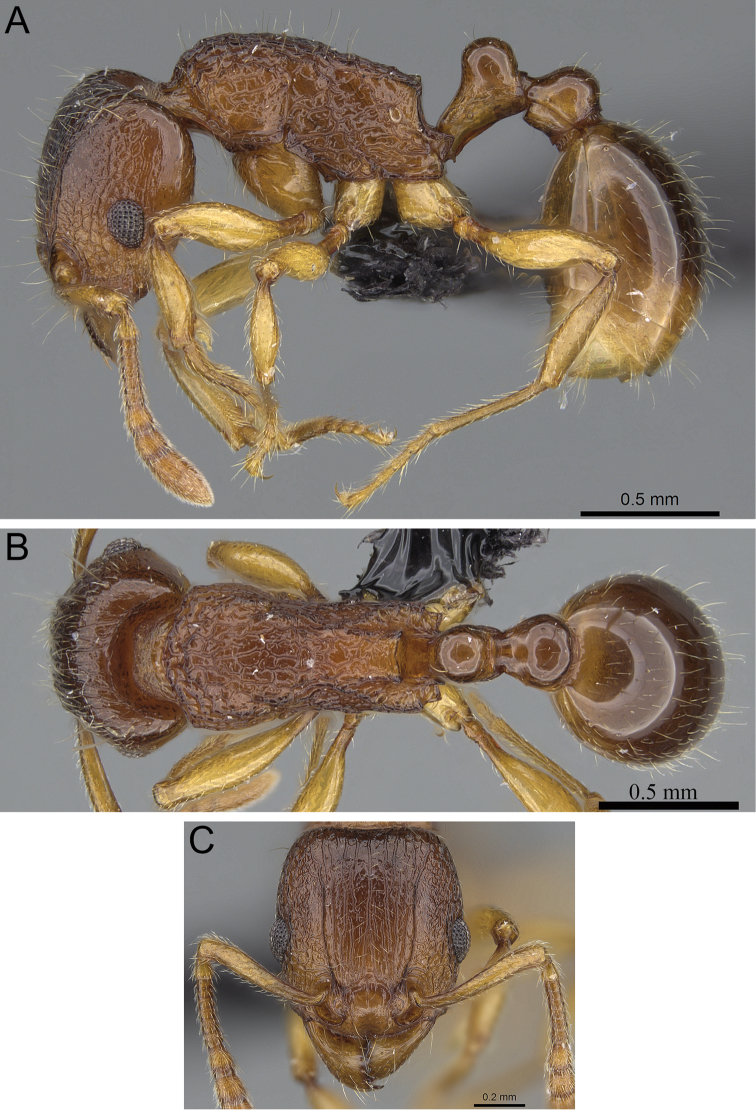
*Tetramorium merina* holotype worker (CASENT0437226). **A** Body in profile **B** Body in dorsal view **C** Head in full-face view.

####### Non-type material.

MADAGASCAR: no locality (*Sikora*); Antananarivo, Réserve Spéciale d’Ambohitantely, Forêt d’Ambohitantely, 20.9 km 72° NE d Ankazobe, 18.22528°S, 47.28683°E, 1410 m, montane rainforest, 17.–22.IV.2001 (*B.L. Fisher et al.*); Antananarivo, Réserve Spéciale d’Ambohitantely, Forêt d’Ambohitantely, Jardin Botanique, 24.1 km 59° NE d Ankazobe, 18.17139°S, 47.28182°E, 1620 m, montane rainforest, 17.–22.IV.2001 (*B.L. Fisher et al.*); Antananarivo, Réserve Spéciale d’Ambohitantely, Forêt d’Ambohitantely, 18.18762°S, 47.28576°E, 1580 m, montane forest, 8.III.2012 (*B.L. Fisher et al.*); Antananarivo, Réserve Spéciale d’Ambohitantely, Forêt d’Ambohitantely, 18.22444°S, 47.2774°E, 1490 m, montane forest, 9.III.2012 (*B.L. Fisher et al.*); Antananarivo, 3 km 41° NE Andranomay, 11.5 km 147° SSE Anjozorobe, 18.47333°S, 47.96°E, 1300 m, montane rainforest, 5.–13.XII.2000 (*B.L. Fisher et al.*); Antananarivo, Stn. Forestiére Manjakatompo, 19.35°S, 47.31667°E, 1600 m, montane rainforest, 20.II.1993 (*P.S. Ward*); Antananarivo, Réserve Naturelle Sohisika, Sohisika 24.6 km NNE Ankazobe, 18.10322°S, 47.18692°E, 1464 m, gallery montane forest, 1.–2.VI.2008 (*B.L. Fisher et al.*); Fianarantsoa, 29 km SSW Ambositra, Ankazomivady, 20.77667°S, 47.165°E, 1700 m, disturbed montane forest, 10.–15.I.1998 (*H.G. Robertson*); Fianarantsoa, Forêt d’Atsirakambiaty, 7.6 km 285° WNW Itremo, 20.59333°S, 46.56333°E, 1550 m, montane rainforest, 22.–26.I.2003 (*B.L. Fisher et al.*).

####### Diagnosis.

The following character combination separates *Tetramorium merina* from the remaining species of the *Tetramorium schaufussii* complex: larger species (HW 0.69–0.79; WL 1.01–1.10); head longer than wide (CI 92–95); eyes relatively large (OI 26–28); frontal carinae usually weakly developed, only slightly raised, often not better developed than cephalic rugulae, and usually fading out halfway between posterior eye margin and posterior head margin; propodeal spines reduced to very short triangular teeth/denticles (PSLI 8–11), propodeal lobes short and triangular, always appearing more voluminous than propodeal teeth, and spines and lobes not strongly inclined towards each other; petiolar node rounded nodiform to rounded high nodiform, in profile around 1.6 to 1.8 times higher than long (LPeI 57–64), in dorsal view around 1.2 times wider than long (DPeI 117–124); dorsum of promesonotum with at least ten pairs of long, fine, standing hairs, propodeum without standing pilosity, and both waist segments with few long pairs each; body uniformly light to dark brown, gaster often darker, appendages usually lighter.

####### Worker measurements

**(N=12).** HL 0.75–0.84 (0.79); HW 0.69–0.79 (0.73); SL 0.51–0.57 (0.54); EL 0.18–0.21 (0.19); PH 0.37–0.40 (0.39); PW 0.52–0.57 (0.54); WL 1.01–1.10 (1.05); PSL 0.06–0.10 (0.08); PTL 0.16–0.20 (0.18); PTH 0.28–0.31 (0.30); PTW 0.19–0.24 (0.22); PPL 0.21–0.24 (0.22); PPH 0.28–0.30 (0.29); PPW 0.27–0.31 (0.29); CI 92–95 (93); SI 72–75 (74); OI 26–28 (26); DMI 50–53 (52); LMI 36–38 (37); PSLI 8–11 (10); PeNI 36–43 (40); LPeI 57–64 (61); DPeI 117–124 (120); PpNI 50–56 (53); LPpI 71–80 (75); DPpI 128–136 (131); PPI 125–142 (133).

####### Worker description.

Head longer than wide (CI 92–95); posterior head margin weakly concave. Anterior clypeal margin with distinct median impression. Frontal carinae usually weakly developed, only slightly raised, often not better developed than cephalic rugulae, and usually fading out halfway between posterior eye margin and posterior head margin. Antennal scrobes present but weak, shallow and without clear and distinct posterior and ventral margins. Antennal scapes short, not reaching posterior head margin (SI 72–75). Eyes relatively large (OI 26–28). Mesosomal outline in profile flat to weakly convex, comparatively low and elongate (LMI 36–38), weakly marginate from lateral to dorsal mesosoma; promesonotal suture absent; metanotal groove very weakly developed to absent. Propodeal spines reduced to very short, triangular, and acute or blunt teeth/denticles (PSLI 8–11), propodeal lobes short, triangular, and acute or blunt, always appearing more voluminous than propodeal teeth, spines and lobes not strongly inclined towards each other. Petiolar node in profile nodiform to high nodiform with well-rounded antero- and posterodorsal margins, around 1.6 to 1.8 times higher than long (LPeI 57–64), anterior and posterior faces approximately parallel, anterodorsal and posterodorsal margins situated at about same height, petiolar dorsum weakly to moderately convex; petiolar node in dorsal view around 1.2 times wider than long (DPeI 117–124), in dorsal view pronotum between 2.3 to 2.7 times wider than petiolar node (PeNI 36–43). Postpetiole in profile globular, approximately 1.3 to 1.4 times higher than long (LPpI 71–80); in dorsal view around 1.3 to 1.4 times wider than long (DPpI 128–136), pronotum between 1.8 to 2.0 times wider than postpetiole (PpNI 50–56). Postpetiole in profile appearing slightly lower or of same height but thicker than petiolar node, postpetiole in dorsal view around 1.3 to 1.4 times wider than petiolar node (PPI 125–142). Mandibles unsculptured, smooth, and shiny; clypeus weakly longitudinally rugulose with two to five lateral rugulae, median area usually completely unsculptured or only weakly sculptured, median ruga usually absent, at most traces present, lateral rugulae often interrupted or irregularly shaped, but at least one lateral rugula well developed on each side; cephalic dorsum between frontal carinae irregularly longitudinally rugulose with eight to ten rugulae; rugulae running from posterior clypeal margin to posterior head margin, often meandering, broken or with cross-meshes; scrobal area mostly unsculptured and laterally merging with surrounding reticulate-rugose sculpture present on lateral head; ground sculpture on head weakly to moderately punctate. Dorsum of mesosoma reticulate-rugose, especially anteriorly, to irregularly longitudinally rugose, lateral mesosoma mostly irregularly longitudinally rugose; ground sculpture on mesosoma weak to absent. Forecoxae mainly unsculptured, smooth, and shining. Waist segments and gaster unsculptured, smooth, and shining. Dorsum of head with numerous pairs of long, fine, standing hairs; dorsum of mesosoma with at least eleven or twelve pairs of long, standing hairs ranging from anterior pronotum to posterior mesonotum, propodeum without long, standing pilosity; petiole usually with two pairs and postpetiole with three to four pairs; first gastral tergite with short, scarce, appressed pubescence in combination with abundant, long, standing hairs. Anterior edges of antennal scapes and dorsal (outer) surfaces of hind tibiae with decumbent to suberect hairs. Body uniformly light brown to dark brown, gaster often of darker brown, appendages usually lighter.

####### Etymology.

The new species is named for the Merina people of Madagascar, in honor of their culture and language. The distribution range of *Tetramorium merina* fits the boundaries of the traditional Merina kingdom very well. The species epithet is an arbitrary combination of letters, thus invariant.

####### Distribution and biology.

*Tetramorium merina* is only known from the Central Highlands of Madagascar (Fig. 65). Its distribution ranges from the two southern-most localities Atsirakambiaty and Ankazomivady through Manjakatompo north to Andranomay, Ambohitantely and Sohisika. All localities are montane rainforests situated at elevations of 1410 to 1700 m, where *Tetramorium merina* appears to live in leaf litter.

####### Discussion.

*Tetramorium merina* is only moderately distinct within the *Tetramorium schaufussii* complex, and can be confused with several other species. The possession of large eyes (OI 26–28) separates *Tetramorium merina* from *Tetramorium pseudogladius* (OI 20), and the petiolar node, which in dorsal view is around 1.2 times wider than long (DPeI 117–124) distinguishes it from *Tetramorium nassonowii* since the petiolar node of the latter in dorsal view is between 1.0 to 1.2 times longer than wide (DPeI 87–98). *Tetramorium merina* with its very short propodeal teeth (PSLI 8–11) is also not likely to be mistaken for *Tetramorium scutum*, which possesses much longer propodeal spines (PSLI 22–24) and relatively well-developed propodeal lobes. Also, in *Tetramorium scutum* the propodeal spines and lobes are strongly inclined towards each other, a condition absent in *Tetramorium merina*. *Tetramorium rala*, which is also the smallest species of the complex (HW 0.46–0.49; WL 0.58–0.64), has relatively long propodeal spines (PSLI 21–25), and the body is very bright yellowish to light brown, and thus not confusable with *Tetramorium merina*. In addition, the latter has numerous pairs of long, fine, standing hairs on each waist segment distinguishing it from *Tetramorium sikorae*, which lacks any standing pilosity on the waist segments.

The species closest to *Tetramorium merina* are *Tetramorium monticola*, *Tetramorium obiwan*, *Tetramorium schaufussii*, and *Tetramorium xanthogaster*, and the differentiation between these is sometimes challenging. *Tetramorium merina* is a relatively large species (HW 0.69–0.79; WL 1.01–1.10) and could be confused with *Tetramorium obiwan*, which is in the same size range or even larger (HW 0.71–0.84; WL 1.00–1.20), or *Tetramorium xanthogaster*, which is usually smaller but reaches its upper size limit in the range of *Tetramorium merina* (HW 0.54–0.75; WL 0.72–1.05). However, *Tetramorium obiwan* has longer antennal scapes (SI 77–82), better-developed frontal carinae, and usually no standing pilosity on the waist segments contrasting with the shorter antennal scapes (SI 72–75), much weaker frontal carinae and the presence of several pairs of standing pilosity on the waist segments of *Tetramorium merina*. Furthermore, *Tetramorium xanthogaster*, despite being a very variable species throughout its range, can be easily separated from *Tetramorium merina* in the central Highlands where they are usually found occurring in sympatry. In this region *Tetramorium xanthogaster* is always strongly bicoloured with head and mesosoma very dark brown to black, strongly contrasting with the yellow to light brown waist segments and gaster. Also, *Tetramorium xanthogaster* usually has long, fine, standing pilosity on the propodeal dorsum while *Tetramorium merina* lacks any standing pilosity on the propodeum. Few other diagnostic characters separate both species, but the fact that they co-occur in sympatry in several localities without any intermediate forms is a good indication of their heterospecificity. *Tetramorium monticola*, which is found in the northeast and north of Madagascar, does not co-occur with *Tetramorium merina*, and both are relatively easy to differentiate. In *Tetramorium monticola* the frontal carinae are relatively better developed, the cephalic dorsum between them has nine to thirteen relatively regularly shaped, mostly unbroken rugae, and the propodeum is armed with short to medium-sized spines (PSLI 18–22) while *Tetramorium merina* has much weaker frontal carinae with eight to ten, often broken and irregular rugulae, and the propodeum is only armed with small teeth/denticles (PSLI 8–11).

The most difficult species to separate from *Tetramorium merina* is certainly *Tetramorium schaufussii*. As is the case with *Tetramorium xanthogaster*, *Tetramorium merina* is also found regularly in sympatry with *Tetramorium schaufussii*, and again, they are separable based on a few characters. *Tetramorium merina* is larger in size (HW 0.69–0.79; WL 1.01–1.10), has a broader head (CI 92–95), and longer (though not significantly so) antennal scapes (SI 72–75) than *Tetramorium schaufussii* (HW 0.51–0.68; WL 0.73–0.93; CI 86–90; SI 66–73). These differences seem difficult to assess without measuring, except for body size, which however, can vary from one population to another and therefore should only be used with caution. Nevertheless, where the two species are found in sympatry (often they are found within the same litter sample) they can be easily discriminated based on body size. In contrast to *Tetramorium schaufussii* and *Tetramorium xanthogaster*, which are both very variable species, *Tetramorium merina* is very stable in its morphological appearance.

###### Tetramorium monticola

Hita Garcia & Fisher sp. n.

http://zoobank.org/F1CDB914-B817-44BA-8825-013D6B9DFD96

http://species-id.net/wiki/Tetramorium_monticola

**Figure 51.**
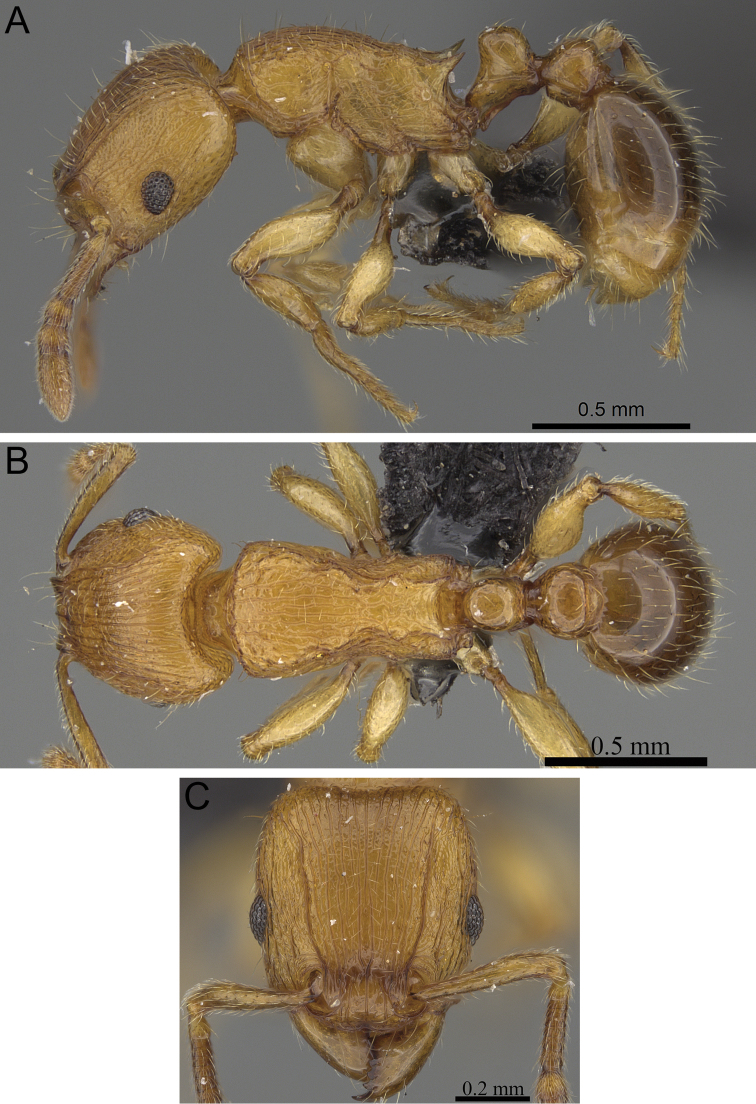
*Tetramorium monticola* holotype worker (CASENT0152401). **A** Body in profile **B** Body in dorsal view **C** Head in full-face view.

Figs 42B , 44C , 45C , 47D , 48A , 48B , 51 , 65

####### Type material.

**Holotype**, pinned worker, MADAGASCAR, Antsiranana, Betaolana Forest, along Bekona River, 14.52996°S, 49.44039°E, 880 m, rainforest, ex rotten log, collection code BLF22473, 4.III.2009 (*B.L. Fisher et al.*) (CAS: CASENT0152401). **Paratypes**, 1 pinned worker with same data as holotype (CAS: CASENT0152402); three pinned workers with same data as holotype except collection codes BLF22465, BLF22486, and BLF22504 (CAS: CASENT0151864; CASENT0151868; CASENT0152427); and four pinned workers with same data as holotype except collection date 5.III.2009 and collection codes BLF22612, BLF22644, and BLF22667 (BMNH: CASENT0151589; CAS: CASENT0151590; CASENT0151949; MCZ: CASENT0152285).

####### Non-type material.

MADAGASCAR: Antsiranana, Rés. Anjanaharibe-Sud, 9.2 km WSW Befingotra, 14.75°S, 49.46667°E, 1200–1280 m, montane rainforest, 6.–10.XI.1994 (*B.L. Fisher*); Antsiranana, Betaolana Forest, along Bekona River, 14.52996°S, 49.44039°E, 880 m, rainforest, 4.–5.III.2009 (*B.L. Fisher et al.*); Antsiranana, Makirovana forest, 14.17066°S, 49.95409°E, 415 m, rainforest, 29.IV.2011 (*B.L. Fisher et al.*); Antsiranana, Makirovana forest, 14.16666°S, 49.95°E, 715 m, rainforest, 2.V.2011 (*B.L. Fisher et al.*); Antsiranana, R.S. Manongarivo, 14.5 km 220° SW Antanambao, 13.99833°S, 48.42833°E, 1175 m, montane rainforest, 20.X.1998 (*B.L. Fisher*); Antsiranana, RNI Marojejy, 10.5km NW Manantenina, 14.43333°S, 49.75°E, 1625 m, montane rainforest, 6.–12.XI.1996 (*E.L. Quinter*); Antsiranana, Parc National de Marojejy, Manantenina River, 28.0 km 38° NE Andapa, 8.2 km 333° NNW Manantenina, 14.43667, S, 49.775°E, 450 m, rainforest, 12.–15.XI.2003 (*B.L. Fisher et al.*); Antsiranana, Parc National de Marojejy, Manantenina River, 27.6 km 35° NE Andapa, 9.6 km 327° NNW Manantenina, 14.435°S, 49.76°E, 775 m, rainforest, 17.XI.2003 (*B.L. Fisher*); Antsiranana, Parc National de Marojejy, Antranohofa, 26.6 km 31° NNE Andapa, 10.7 km 318° NW Manantenina, 14.44333°S, 49.74333°E, 1325 m, montane rainforest, 18.–21.XI.2003 (*B.L. Fisher*); Antsiranana, Parc National de Marojejy, 25.7 km 32° NNE Andapa, 10.3 km 314° NW Manantenina, 14.445°S, 49.74167°E, 1575 m, montane rainforest, 21.–25.XI.2003 (*B.L. Fisher*); Antsiranana, Parc National de Marojejy, 25.4 km 30° NNE Andapa, 10.9 km 311° NW Manantenina, 14.445°S, 49.735°E, 2000 m, montane rainforest, 23.XI.2003 (*B.L. Fisher*); Toamasina, Montagne d’Akirindro 7.6 km 341° NNW Ambinanitelo, 15.28833°S, 49.54833°E, 600 m, rainforest, 17.–21.III.2003 (*B.L. Fisher et al.*); Toamasina, 6.9 km NE Ambanizana, Ambohitsitondroina, 15.56667°S, 50°E, 1000 m, montane rainforest, 8.XII.1993 (*B.L. Fisher*); Toamasina, Ambanizana, Parc National Masoala, 15.57167°S, 50.00611°E, 848–925 m, montane rainforest, 26.II.-2.III.2003 (*D. Andriamalala, D. Silva, et al.*); Toamasina, Montagne d’Anjanaharibe, 18 km 21° NNE Ambinanitelo, 15.18833°S, 49.615°E, 470 m, rainforest, 8.–12.III.2003 (*B.L. Fisher et al.*); Toamasina, Montagne d’Anjanaharibe, 19.5 km 27° NNE Ambinanitelo, 15.17833°S, 49.635°E, 1100 m, montane rainforest, 12.–16.III.2003 (*B.L. Fisher et al.*); Toamasina, F.C. Sandranantitra, 18.04833°S, 49.09167°E, 450 m, rainforest, 18.–21.I.1999 (*H.J. Ratsirarson*); Toamasina, Parc National de Zahamena, Tetezambatana forest, near junction of Nosivola and Manakambahiny Rivers, 17.74298°S, 48.72936°E, 860 m, rainforest, 18.II.2009 (*B.L. Fisher et al.*); Toamasina, Parc National de Zahamena, 17.73359°S, 48.72625°E, 950 m, rainforest, 19.II.2009 (*B.L. Fisher et al.*); Toamasina, Parc National de Zahamena, Onibe River, 17.75908°S, 48.85468°E, 780 m, rainforest, 21.II.2009 (*B.L. Fisher et al.*).

####### Diagnosis.

The following character combination distinguishes *Tetramorium monticola* from the other species of the *Tetramorium schaufussii* complex: head longer than wide (CI 92–94); eyes relatively large (OI 24–28); antennal scapes very short (SI 67–74); propodeal spines of moderate length, elongate-triangular to spinose, and usually acute (PSLI 8–11), propodeal lobes short, triangular, and usually blunt, always much smaller than propodeal spines, spines and lobes not strongly inclined towards each other; petiolar node high nodiform to high cuneiform, in profile around 1.7 to 2.0 times higher than long (LPeI 50–60), node in dorsal view around 1.2 to 1.3 times wider than long (DPeI 124–133); both waist segments with long, standing pilosity; body uniformly dark yellow to orange light brown.

####### Worker measurements

**(N=12).** HL 0.55–0.72 (0.62); HW 0.51–0.67 (0.58); SL 0.34–0.46 (0.40); EL 0.13–0.16 (0.15); PH 0.26–0.33 (0.29); PW 0.35–0.48 (0.41); WL 0.67–0.88 (0.77); PSL 0.10–0.15 (0.12); PTL 0.11–0.15 (0.12); PTH 0.20–0.26 (0.23); PTW 0.13–0.19 (0.16); PPL 0.14–0.18 (0.16); PPH 0.18–0.24 (0.21); PPW 0.19–0.26 (0.23); CI 92–94 (93); SI 67–74 (69); OI 24–28 (25); DMI 51–55 (54); LMI 35–39 (37); PSLI 18–22 (20); PeNI 37–42 (39); LPeI 50–60 (54); DPeI 124–133 (129); PpNI 54–60 (56); LPpI 71–78 (76); DPpI 131–153 (142); PPI 138–150 (143).

####### Worker description.

Head longer than wide (CI 92–94); posterior head margin weakly concave. Anterior clypeal margin with distinct median impression. Frontal carinae usually well developed, moderately raised on anterior half, strongly diverging posteriorly, and usually approaching or ending at posterior head margin. Antennal scrobes usually weakly developed, shallow and without clear and distinct posterior and ventral margins, sometimes scrobe better developed, slightly deeper and with weak posterior, and rarely even ventral margin. Antennal scapes very short, not reaching posterior head margin (SI 67–74). Eyes relatively large (OI 24–28). Mesosomal outline in profile flat to weakly convex, comparatively low and elongate (LMI 35–39), weakly marginate from lateral to dorsal mesosoma; promesonotal suture absent; metanotal groove weakly to moderately developed, sometimes slightly impressed, but mostly moderately deep. Propodeal spines of moderate length, elongate-triangular to spinose, and usually acute (PSLI 8–11), propodeal lobes short, triangular, and usually blunt, always much smaller than propodeal spines, spines and lobes not strongly inclined towards each other. Petiolar node in profile high nodiform to high cuneiform, around 1.7 to 2.0 times higher than long (LPeI 50–60), anterior and posterior faces approximately parallel, anterodorsal margin always higher and more angled than posterodorsal margin, petiolar dorsum usually weakly convex and tapering backwards posteriorly; petiolar node in dorsal view around 1.2 to 1.3 times wider than long (DPeI 124–133), in dorsal view pronotum between 2.4 to 2.7 times wider than petiolar node (PeNI 37–42). Postpetiole in profile globular, approximately 1.3 to 1.4 times higher than long (LPpI 71–78); in dorsal view around 1.3 to 1.5 times wider than long (DPpI 131–153), pronotum between 1.7 to 1.9 times wider than postpetiole (PpNI 54–60). Postpetiole in profile appearing always lower and having less volume than petiolar node, postpetiole in dorsal view around 1.4 to 1.5 times wider than petiolar node (PPI 138–150). Mandibles unsculptured, smooth, and shiny; clypeus usually longitudinally rugulose with two to six rugulae, median area usually either completely unsculptured without median rugula or only weakly sculptured with traces of median rugula, very rarely median rugula fully developed, lateral rugulae usually well developed and unbroken, sometimes irregularly shaped or broken; cephalic dorsum between frontal carinae longitudinally rugose with nine to thirteen rugae; rugae running from posterior clypeal margin to posterior head margin, generally very regularly shaped and only rarely broken or with cross-meshes; scrobal area partly unsculptured and laterally merging with surrounding longitudinally rugulose to reticulate-rugose sculpture present on lateral head; ground sculpture on head usually moderately punctate. Dorsum of mesosoma usually longitudinally rugose, sometimes irregularly so; lateral mesosoma irregularly longitudinally rugose to reticulate-rugose, sometimes lateral pronotum with less sculpture; ground sculpture on mesosoma weak to absent. Forecoxae always unsculptured, smooth and shining. Waist segments and gaster unsculptured, smooth, and shining. Dorsum of head with numerous pairs of long, fine, standing hairs; dorsum of promesonotum with at least ten pairs of long, standing hairs ranging from anterior pronotum to posterior mesonotum, propodeum usually without long, standing pilosity, sometimes with one or two shorter pairs of hairs; petiole usually with at least two pairs and postpetiole with at least three to four pairs; first gastral tergite with short, scarce to abundant, decumbent to appressed pubescence in combination with abundant, long, standing hairs. Anterior edges of antennal scapes and dorsal (outer) surfaces of hind tibiae usually with decumbent to suberect hairs. Body uniformly dark yellow to orange light brown, gaster sometimes of darker brown, appendages usually slightly lighter.

####### Etymology.

The name of the new species is a Latin noun and means “inhabitant of the mountains” referring to the fact that *Tetramorium monticola* is predominantly found in higher elevation montane forests. The species epithet is a nominative noun, and thus invariant.

####### Distribution and biology.

*Tetramorium monticola* is distributed in the northeast and north of Madagascar (Fig. 65). The distribution range is outlined by Manongarivo in the west, the northernmost locality Makirovana, and the easternmost Ambanizana on the Masoala Peninsula. Most of the material available was collected in that triangle, but *Tetramorium monticola* is also known from Zahamena and Sandranantitra, which are located much further south. All localities are rainforests or montane rainforests situated at elevation ranging from 415 to 2000 m. Also, it seems that *Tetramorium monticola* lives in leaf litter.

####### Discussion.

*Tetramorium monticola* is well recognisable within the *Tetramorium schaufussii* complex. It differs from *Tetramorium pseudogladius* (OI 20) by its much larger eye size (OI 24–28), and from *Tetramorium scutum* by having propodeal spines and lobes not strongly inclined towards each other. Also, *Tetramorium monticola* (DPeI 124–133; LPeI 50–60) is very unlikely to be confused with *Tetramorium nassonowii*, which has a much longer and lower petiolar node (DPeI 87–98; LPeI 72–81). In addition, *Tetramorium monticola* is a relatively hairy species with long pilosity all over the body, which separates it from the few species with partly reduced pilosity, such as *Tetramorium rala*, *Tetramorium sikorae*, and *Tetramorium obiwan*, which all usually lack standing pilosity on the propodeum and waist segments, except in *Tetramorium obiwan*, where the petiole or postpetiole occasionally have a few long hairs. Nevertheless, *Tetramorium monticola* cannot be mistaken for *Tetramorium obiwan*. The latter is of larger body size (HW 0.71–0.84; WL 1.00–1.20), has longer antennal scapes (SI 77–82), and has a petiolar node with the antero- and posterodorsal margins situated at about the same height and well rounded. *Tetramorium monticola* is much smaller than that (HW 0.51–0.67; WL 0.67–0.88), has shorter antennal scapes (SI 67–74), and its petiolar node has the anterodorsal margin always higher and a little bit more angled than the posterodorsal margin with the petiolar dorsum tapering backwards.

The remaining three species, *Tetramorium merina*, *Tetramorium schaufussii*, and *Tetramorium xanthogaster*, are morphologically closer to *Tetramorium monticola*, and their separation requires more attention. *Tetramorium schaufussii* can be easily mistaken for *Tetramorium monticola* in northern Madagascar where both are sympatric, but *Tetramorium schaufussii* differs from *Tetramorium monticola* (and the other two) by having a much longer and thinner head in full-face view (CI 86–90). The other two species, *Tetramorium merina* and *Tetramorium xanthogaster*, usually have shorter, and in the case of *Tetramorium merina* much shorter, propodeal spines (PSLI 8–16) than *Tetramorium monticola* (PSLI 18–22). However, since the spine length is somewhat variable in the latter species, this character cannot be the sole distinguishing feature. In addition, *Tetramorium monticola* also differs from the other two by having relatively better developed frontal carinae and the cephalic dorsum between them with nine to thirteen relatively regularly shaped, mostly unbroken rugae. *Tetramorium merina* and *Tetramorium xanthogaster* have relatively weaker developed, and often shorter, frontal carinae and a cephalic dorsum with six to ten relatively irregularly shaped, often meandering or broken rugulae.

###### Tetramorium nassonowii

Forel, 1892 stat. n.

Figs 40A , 52 , 65

Tetramorium nassonowii Forel, 1892: 521. [Synonymy with *Tetramorium schaufussii* by Bolton 1979: 137]

####### Type material.

**Lectotype [designated here**], pinned worker, MADAGASCAR, Antananarivo (Province central de Madagascar), Andrangoloaca, 47.27729°E, 18.22198 S (*Sikora*) (MHNG: CASENT0101289) [examined]. **Paralectotype [designated here**], pinned worker with same data as lectotype (MHNG: CASENT0101290).

[Note 1: the GPS data of the type locality was not provided by the locality label or the original description. The data presented above is based on our own geo-referencing of the Foret d’ Andrangoloaca and should be considered as an approximation and not the exact position of the type locality.]

[Note 2: one specimen from the MHNG collection (CASENT0101556) with the label data “*Tetramorium nassonowii* Forel var., no. 14, Moramanga (*Sikora*)” also has a red type label. However, we do not consider this as a real name-bearing type specimen since the original description of Forel (1892) only mentioned the type material from Andrangoloaca and nothing from Moramanga. This specimen from Moramanga (which is also partly broken) is actually not conspecific with *Tetramorium nassonowii*, but belongs to the newly described species *Tetramorium obiwan*.]

**Figure 52.**
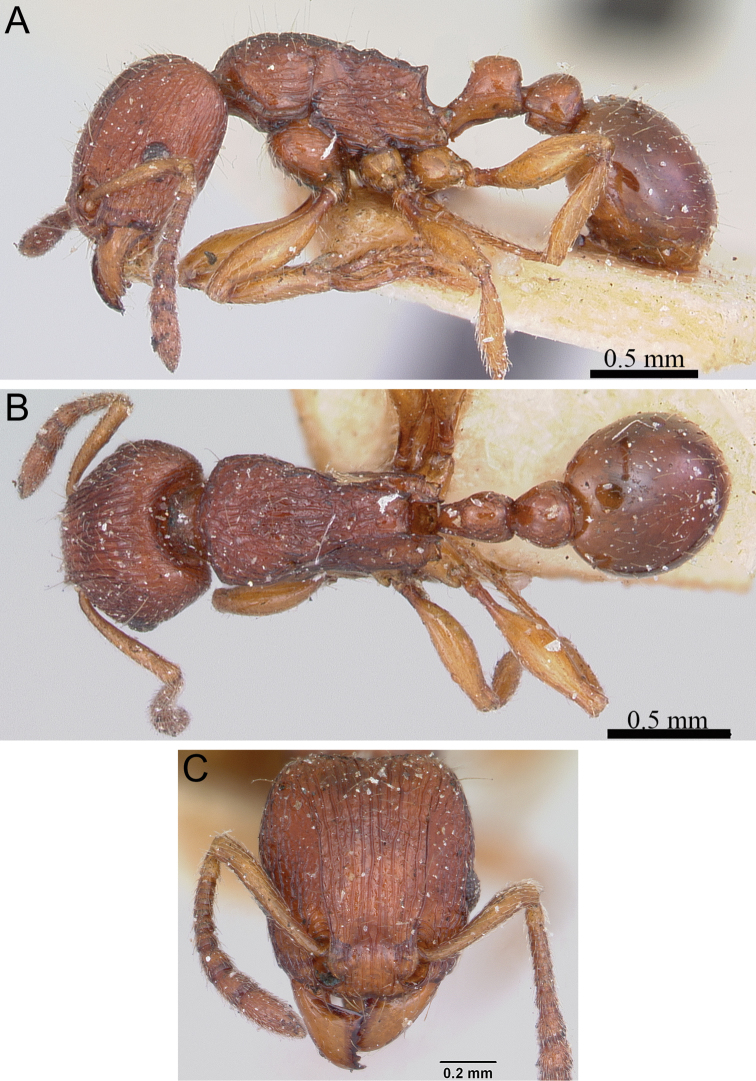
*Tetramorium nassonowii* syntype worker (CASENT0101289). **A** Body in profile **B** Body in dorsal view **C** Head in full-face view.

####### Non-type material.

MADAGASCAR: Antananarivo, 3 km 41° NE Andranomay, 11.5 km 147° SSE Anjozorobe, 18.47333°S, 47.96°E, 1300 m, montane rainforest, 5.–13.XII.2003 (*B.L. Fisher et al*); Antsiranana, Rés. Anjanaharibe-Sud, 9.2 km WSW Befingotra, 14.75°S, 49.46667°E, 1260 m, 11.XI.1994 (*B.L. Fisher*); Antsiranana, Rés. Anjanaharibe-Sud, 11.0 km WSW Befingotra, 14.75°S, 49.45°E, 1565 m, montane rainforest, 16.XI.1994 (*B.L. Fisher*); Fianarantsoa, 29 km SSW Ambositra, Ankazomivady, 20.77667°S, 47.165°E, 1700 m, disturbed montane rainforest, 7.I.1998 (*B.L. Fisher*); Fianarantsoa, Ranomafana National Park, 21.21667°S, 47.41667°E, 706 m, montane rainforest, 1.X.1992 (*E. Rajeriarison*); Toamasina, 5.3 km SSE Ambanizana, Andranobe, 15.67133°S, 49.97395°E, 425 m, rainforest, 21.XI.1993 (*B.L. Fisher*); Toamasina, F.C. Andriantantely, 18.695°S, 48.81333°E, 530 m, rainforest, 4.–10.XII.1998 (*H.J. Ratsirarson*); Toamasina, Montagne d’Anjanaharibe, 19.5 km 27° NNE Ambinanitelo, 15.17833°S, 49.635°E, 1100 m, montane rainforest, 12.–16.III.2003 (*B.L. Fisher et al.*); Toamasina, F.C. Didy, 18.19833°S, 48.57833°E, 960 m, rainforest, 16.–23.XII.1998 (*H.J. Ratsirarson*); Toliara, Parc National d’Andohahela, Manampanihy River, 5.4 km 113° ESE Mahamavo, 36.7 km 343° NNW Tolagnaro, 24.76389°S, 46.76683°E, 650 m, rainforest, 24.I.2002 (*B.L. Fisher et al.*); Toliara, Réserve Spéciale Kalambatritra, Befarara, 23.4178°S, 46.4478°E, 1390 m, montane rainforest, 7.II.2009 (*B.L. Fisher et al.*); Toliara, Réserve Spéciale Kalambatritra, Befarara, 23.4144°S, 46.459°E, 1360 m, montane rainforest, 8.II.2009 (*B.L. Fisher et al.*); Toliara, Réserve Spéciale Kalambatritra, Befarara, 23.4502°S, 46.45658°E, 1325 m, montane rainforest, 11.II.2009 (*B.L. Fisher et al.*).

####### Diagnosis.

*Tetramorium nassonowii* is clearly recognisable within the *Tetramorium schaufussii* complex on the basis of its petiolar node shape, which is relatively long and low, in profile around 1.2 to 1.4 times higher than long (LPeI 72–81) and in dorsal view between 1.0 to 1.2 times longer than wide (DPeI 87–98).

####### Worker measurements

**(N=12).** HL 0.82–0.96 (0.87); HW 0.74–0.86 (0.78); SL 0.52–0.64 (0.57); EL 0.18–0.21 (0.19); PH 0.38–0.45 (0.41); PW 0.54–0.62 (0.57); WL 1.02–1.16 (1.08); PSL 0.06–0.09 (0.08); PTL 0.21–0.26 (0.23); PTH 0.28–0.34 (0.30); PTW 0.20–0.25 (0.22); PPL 0.21–0.26 (0.23); PPH 0.28–0.34 (0.30); PPW 0.27–0.34 (0.30); CI 90–92 (91); SI 70–74 (72); OI 23–25 (24); DMI 51–54 (53); LMI 36–39 (38); PSLI 7–11 (9); PeNI 36–40 (38); LPeI 72–81 (77); DPeI 87–98 (95); PpNI 50–55 (52); LPpI 75–82 (78); DPpI 122–134 (128); PPI 130–145 (137).

####### Worker description.

Head clearly longer than wide (CI 90–92); posterior head margin weakly concave. Anterior clypeal margin with distinct median impression. Frontal carinae weakly to moderately developed, moderately raised, diverging posteriorly, and usually fading out halfway between posterior eye margin and posterior head margin or approaching posterior head margin. Antennal scrobes present but weak, shallow and without clear and distinct posterior and ventral margins. Antennal scapes short, not reaching posterior head margin (SI 70–74). Eyes moderate to large (OI 23–25). Mesosomal outline in profile flat to weakly convex, comparatively low and long (LMI 36–39), weakly to moderately marginate from lateral to dorsal mesosoma; promesonotal suture absent; metanotal groove weakly developed or absent. Propodeal spines reduced to very short teeth (PSLI 7–11), propodeal lobes short, triangular, and blunt or acute, usually longer than propodeal spines, rarely as long as propodeal spines, spines and lobes not strongly inclined towards each other. Petiolar node rounded nodiform, in profile around 1.2 to 1.4 times higher than long (LPeI 72–81), anterior and posterior faces not parallel, anterodorsal and posterodorsal margins situated at about same height, petiolar dorsum distinctly convex; node in dorsal view weakly longer than wide (DPeI 92–96), in dorsal view pronotum between 2.5 to 2.8 times wider than petiolar node (PeNI 36–40). Postpetiole in profile globular, approximately 1.2 to 1.3 times higher than long (LPpI 75–82); in dorsal view around 1.2 to 1.3 times wider than long (DPpI 122–134), pronotum between 1.8 to 2.0 times wider than postpetiole (PpNI 50–55). Postpetiole in profile appearing more or less of same volume as petiolar node, postpetiole in dorsal view around 1.3 to 1.5 times wider than petiolar node (PPI 130–145). Mandibles unsculptured, smooth, and shiny; clypeus weakly longitudinally rugulose with three to seven rugulae, rugulae often interrupted or irregularly shaped, median area often weakly sculptured, median ruga usually absent or mostly reduced, very rarely fully developed; cephalic dorsum between frontal carinae irregularly longitudinally rugose/rugulose with six to nine rugae/rugulae; rugae/rugulae running from posterior clypeal margin to posterior head margin, often meandering, broken or with cross-meshes; scrobal area mostly unsculptured and laterally merging with surrounding reticulate-rugose to longitudinally rugose sculpture present on lateral head; ground sculpture on head weak to absent. Dorsum of mesosoma irregularly longitudinally rugose to reticulate-rugose, lateral mesosoma mostly irregularly longitudinally rugose; ground sculpture on mesosoma weak to absent. Forecoxae mainly unsculptured, smooth and shining. Waist segments and gaster unsculptured, smooth, and shining. Dorsum of head with several pairs of long, fine, standing hairs; dorsum of mesosoma with at least six or seven pairs of long, standing hairs ranging from anterior pronotum to posterior mesonotum, propodeum without long, standing pilosity; petiole with one pair and postpetiole with one or two pairs; first gastral tergite with short, scarce, appressed pubescence in combination with scattered, long, standing hairs. Anterior edges of antennal scapes and dorsal (outer) surfaces of hind tibiae with appressed to decumbent hairs. Body uniformly light brown to dark brown colour, appendages often lighter.

####### Distribution and biology.

*Tetramorium nassonowii* is distributed in the montane rainforests and rainforest belt of eastern Madagascar (Fig. 65) at altitudes ranging from 425 to 1700 m, although it is predominantly found in montane rainforests situated higher than 1000 m. Also, based on the available collection data, it seems that *Tetramorium nassonowii* inhabits leaf litter or the ground.

####### Discussion.

In this study we propose to raise *Tetramorium nassonowii*, which was first described by Forel (1892), to species rank. In the original description Forel (1892) compared *Tetramorium nassonowii* with *Tetramorium schaufussii*, and found them to be different in petiolar node shape and propodeal spine length. Much later, in his revision of the Malagasy *Tetramorium*, Bolton (1979) synonymised *Tetramorium nassonowii* under *Tetramorium schaufussii*, likely because the limited material available for both species suggested their conspecificity. Indeed, the only genuine specimens of *Tetramorium nassonowii* available to Forel and Bolton were the two syntypes from MHNG. Based on the examination of a few thousand specimens from the whole *Tetramorium schaufussii* complex, we believe the material listed as *Tetramorium schaufussii* by Bolton (1979) to consist of the species *Tetramorium merina*, *Tetramorium nassonowii*, *Tetramorium obiwan*, and *Tetramorium schaufussi*. *Tetramorium nassonowii* is especially distinctive within the complex due to its large body size and characteristic petiolar node shape. As noted in the diagnosis, the lower and longer petiolar node shape of *Tetramorium nassonowii*, which in profile is around 1.2 to 1.4 times higher than long (LPeI 72–81) and in dorsal view between 1.0 to 1.2 times longer than wide (DPeI 87–98), clearly distinguishes it from the remainder of the *Tetramorium schaufussii* species complex. The other species all have a higher and broader petiolar node, in profile around 1.5 to 2.2 times higher than long (LPeI 45–67) and in dorsal view between 1.1 to 1.5 times wider than long (DPeI 109–154).

Despite the relatively wide distribution, we find *Tetramorium nassonowii* without much observable intraspecific variation.

###### Tetramorium obiwan

Hita Garcia & Fisher sp. n.

http://zoobank.org/8EE7AACC-818A-4B99-AB95-75CD1969ECA7

http://species-id.net/wiki/Tetramorium_obiwan

**Figure 53.**
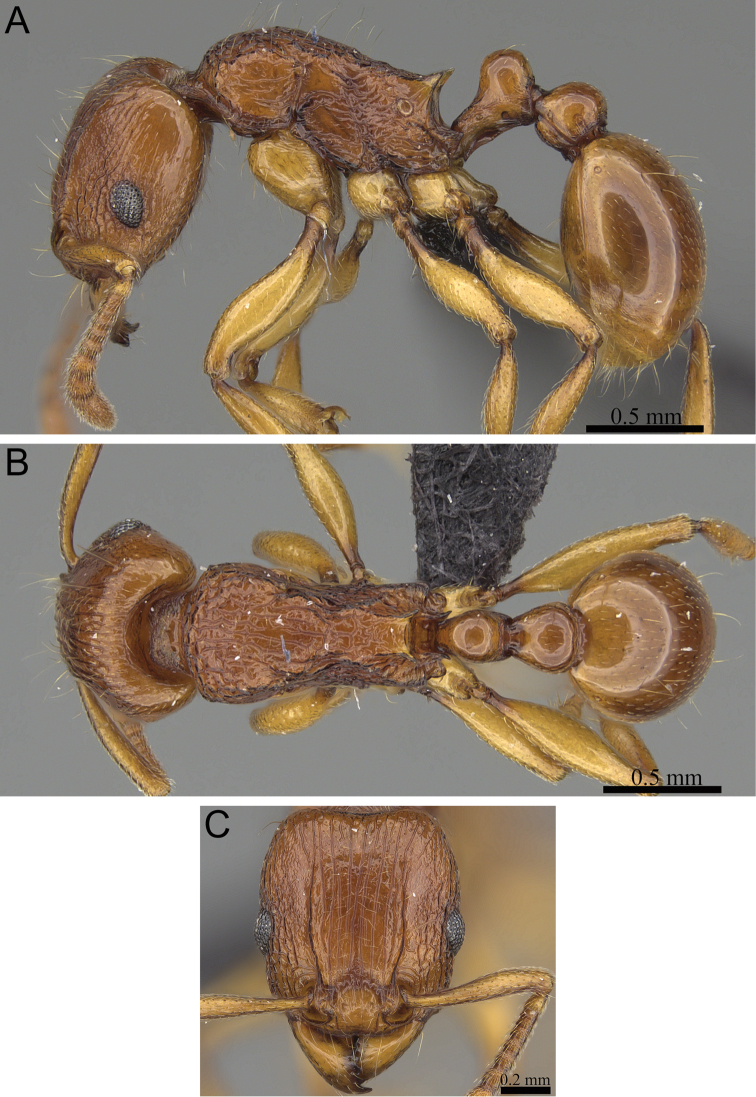
*Tetramorium obiwan* holotype worker (CASENT0447245). **A** Body in profile **B** Body in dorsal view **C** Head in full-face view.

Figs 20D , 43B , 44A , 53 , 65

####### Type material.

**Holotype**, pinned worker, MADAGASCAR, Toliara, Parc National d’Andohahela, Col du Sedro, 3.8 km 113° ESE Mahamavo, 37.6 km 341° NNW Tolagnaro, 24.76389°S, 46.75167°E, 900 m, montane rainforest, beating low vegetation, collection code BLF05014, 21.–25.I.2002 (*B.L. Fisher et al.*) (CAS: CASENT0447245). **Paratypes**, three pinned workers with same data as holotype (CAS: CASENT0447199; CASENT0447207; MCZ: CASENT0447237); eight pinned workers with same data as holotype except sampled from canopy moss and leaf litter and collection codes BLF05036 and BLF05136 (CAS: CASENT0451274; CASENT0451275; CASENT0451276; CASENT0455961); four pinned workers with same data as holotype except sampled from sifted litter and collection code BLF05010 (BMNH: CASENT0484542; CAS: CASENT0484425; CASENT0484536; CASENT0484554).

####### Non-type material.

MADAGASCAR: Antananarivo, 3 km 41° NE Andranomay, 11.5 km 147° SSE Anjozorobe, 18.47333°S, 47.96°E, 1300 m, montane rainforest, 5.–13.XII.2000 (*B.L. Fisher et al.*); Antsiranana, Rés. Anjanaharibe-Sud, 9.2 km WSW Befingotra, 14.75°S, 49.46667°E, 1260 m, montane rainforest, 11.XI.1994 (*B.L. Fisher*); Antsiranana, Betaolana Forest, along Bekona River, 14.52996°S, 49.44039°E, 880 m, rainforest, 4.III.2009 (*B.L. Fisher et al.*); Fianarantsoa, Rés. Andringitra, 40 km S Ambalavao, 22.21667°S, 46.96667°E, 1225 m, montane rainforest, 19.X.1993 (*B.L. Fisher*); Fianarantsoa, Parc National Befotaka-Midongy, Papango 28.5 km S Midongy-Sud, Mount Papango, 23.84083°S, 46.9575°E, 1250 m, montane rainforest, 17.–18.XI.2006 (*B.L. Fisher et al.*); Fianarantsoa, Ranomafana National Park, radio tower, 21.25083°S, 47.40717°E, 1130 m, forest edge, mixed tropical forest, open area, 4.–21.I.2002 (*M. Irwin & R. Harin’Hala*); Fianarantsoa, Parc National de Ranomafana, Vatoharanana River, 4.1 km 231° SW Ranomafana, 21.29°S, 47.43333°E, 1100 m, montane rainforest, 27.–31.III.2003 (*B.L. Fisher et al.*); Fianarantsoa, Parc National de Ranomafana, Sahamalaotra River, 6.6 km 310° NW Ranomafana, 21.23667°S, 47.39667°E, 1150 m, montane rainforest, 31.III.2003 (*B.L. Fisher et al.*); Toamasina, 6 km ESE Andasibe (=Perinet), 18.95°S, 48.46667°E, 900 m, rainforest, 17.XI.1990 (*P.S. Ward*); Toamasina, Corridor Forestier Analamay-Mantadia, Ambatoharanana, 18.80388°S, 48.40506°E, 1013 m, rainforest, 12.–19.2012 (*B.L. Fisher et al.*); Toamasina, Corridor Forestier Analamay-Mantadia, Ambatoharanana, 18.79956°S, 48.4028°E, 1058 m, rainforest, 12.–19.2012 (*B.L. Fisher et al.*); Toamasina, Corridor Forestier Analamay-Mantadia, Ambatoharanana, 18.80398°S, 48.40358°E, 1064 m, rainforest, 12.–19.2012 (*B.L. Fisher et al.*); Toamasina, Ankerana, 18.4017°S, 48.80605°E, 1035 m, montane forest, 26.I.2012 (*B.L. Fisher et al.*); Toliara, Parc National d’Andohahela, Col du Sedro, 3.8 km 113° ESE Mahamavo, 37.6 km 341° NNW Tolagnaro, 24.76389°S, 46.75167°E, 900 m, montane rainforest, 21.–25.I.2002 (*B.L. Fisher et al.*); Toliara, Forêt Ivohibe 55.6 km N Tolagnaro, 24.56167°S, 47.20017°E, 650 m, rainforest, 4.XII.2006 (*B.L. Fisher et al.*).

####### Diagnosis.

The following character combination distinguishes *Tetramorium obiwan* from the other species of the *Tetramorium schaufussii* complex: relatively larger species (HW 0.71–0.84; WL 1.00–1.20); frontal carinae moderately to very well developed, diverging posteriorly, becoming weaker halfway between posterior eye margin and posterior head margin, and ending at or shortly before posterior head margin; antennal scapes short to moderate (SI 77–82); eyes relatively large (OI 25–27); propodeal spines short, triangular to elongate-triangular, and acute (PSLI 10–16), propodeal lobes short, triangular, and blunt or acute, always much shorter than propodeal spines, spines and lobes never strongly inclined towards each other; petiolar node high rounded nodiform, in profile around 1.6 to 1.9 times higher than long (LPeI 54–64), and in dorsal view around 1.1 to 1.3 times wider than long (DPeI 109–129); waist segments usually without any standing hairs, sometimes one pair of long, standing hairs present on petiole and/or postpetiole.

####### Worker measurements

**(N=15).** HL 0.78–0.93 (0.86); HW 0.71–0.84 (0.78); SL 0.56–0.69 (0.62); EL 0.18–0.22 (0.20); PH 0.36–0.44 (0.41); PW 0.50–0.60 (0.57); WL 1.00–1.20 (1.10); PSL 0.08–0.14 (0.12); PTL 0.15–0.20 (0.18); PTH 0.27–0.33 (0.30); PTW 0.18–0.23 (0.21); PPL 0.19–0.24 (0.23); PPH 0.27–0.32 (0.31); PPW 0.25–0.32 (0.29); CI 89–92 (90); SI 77–82 (80); OI 25–27 (26); DMI 50–53 (51); LMI 36–38 (37); PSLI 10–16 (14); PeNI 35–39 (36); LPeI 54–64 (58); DPeI 109–129 (117); PpNI 50–53 (51); LPpI 70–77 (74); DPpI 124–135 (129); PPI 135–148 (142).

####### Worker description.

Head clearly longer than wide (89–92); posterior head margin weakly concave. Anterior clypeal margin with distinct median impression. Frontal carinae moderately to very well developed, diverging posteriorly, becoming weaker halfway between posterior eye margin and posterior head margin, and ending at or shortly before posterior head margin. Antennal scrobes very weak to absent, shallow, and without any posterior and ventral margins. Antennal scapes short to moderate, not reaching posterior head margin (SI 77–82). Eyes relatively large (OI 25–27). Mesosomal outline in profile relatively flat to weakly convex, comparatively low and elongate (LMI 36–38), weakly to moderately marginate from lateral to dorsal mesosoma; promesonotal suture absent; metanotal groove either weakly developed or absent. Propodeal spines short, triangular to elongate-triangular, and acute (PSLI 10–16), propodeal lobes short, triangular, and blunt or acute, always much shorter than propodeal spines, spines and lobes not strongly inclined towards each other. Petiolar node in profile high rounded nodiform, around 1.6 to 1.9 times higher than long (LPeI 54–64), anterior and posterior faces approximately parallel, anterodorsal and posterodorsal margins situated at about same height and well rounded, petiolar dorsum weakly to moderately convex; node in dorsal view around 1.1 to 1.3 times wider than long (DPeI 109–129); in dorsal view pronotum around 2.6 to 2.9 times wider than petiolar node (PeNI 35–39). Postpetiole in profile globular, around 1.3 to 1.4 times higher than long (LPpI 70–77); in dorsal view around 1.2 to 1.4 times wider than long (DPpI 124–135); in dorsal view pronotum around 1.9 to 2.0 times wider than postpetiole (PpNI 50–53). Postpetiole in profile appearing more or less of same volume as petiolar node, postpetiole in dorsal view between 1.3 to 1.5 times wider than petiolar node (PPI 135–148). Mandibles unsculptured, smooth, and shiny; clypeus weakly longitudinally rugulose with three to five, rarely more, rugulae, rugulae often interrupted or irregularly shaped, median area usually weakly sculptured without median rugula, median rugula sometimes partly and rarely fully developed; cephalic dorsum between frontal carinae longitudinally rugose/rugulose with seven to ten rugae/rugulae; rugae/rugulae running from posterior clypeal margin to posterior head margin, often interrupted, or with cross-meshes; scrobal area mostly unsculptured and laterally merging with surrounding reticulate-rugose to longitudinally rugose sculpture present on lateral head, sometimes head posterolaterally with very little sculpture, very smooth, and shining. Dorsum of mesosoma irregularly longitudinally rugose to reticulate-rugose, anterior pronotum especially reticulate-rugose; lateral mesosoma mostly irregularly longitudinally rugose with few unsculptured areas on lateral pronotum and/or katepisternum. Forecoxae mainly unsculptured, smooth, and shining. Waist segments and gaster unsculptured, smooth, and shining. Ground sculpture everywhere on body weak to absent. Dorsum of head with numerous pairs of long, fine, standing hairs; dorsum of mesosoma with seven to ten pairs, hairs present from anterior pronotum to posterior mesonotum, propodeum without standing pilosity; usually waist segments without any standing hairs, sometimes one pair of long, standing hairs present on petiole and/or postpetiole; first gastral tergite with very short, scarce, appressed pubescence in combination with few scarce, long, standing hairs. Anterior edges of antennal scapes and dorsal (outer) surfaces of hind tibiae usually with appressed, rarely decumbent hairs. Head, mesosoma, waist segments and gaster uniformly light reddish, orange-brown to dark brown contrasting with lighter yellowish to light brown mandibles, antennae, and legs.

####### Etymology.

The name of the new species is inspired by a fictional character from George Lucas’ “Star Wars” Universe: the wise Jedi master Obi-Wan Kenobi. The species epithet is an arbitrary combination of letters, thus invariant.

####### Distribution and biology.

*Tetramorium obiwan* is widely but relatively patchily distributed throughout the rainforests and montane rainforests of eastern Madagascar (Fig. 65). Its known distribution encompasses nine localities, ranging from Andohahela in the southeast to Anjanaharibe-Sud in the northeast. A likely explanation for this patchy distribution record may be that *Tetramorium obiwan* lives in the vegetation and is consequently less often sampled by ground or leaf litter methods. The available collection data supports this, as *Tetramorium obiwan* was mainly collected by beating low vegetation or Malaise traps, and rarely from pitfall traps or leaf litter. If true, then *Tetramorium obiwan* is possibly much more widespread than currently known, and more collecting in lower vegetation will likely provide additional material of this species. Furthermore, *Tetramorium obiwan* seems to prefer higher elevations. It can be found from 675 to 1300 m, but most collections were from the upper altitudinal range limit.

####### Discussion.

*Tetramorium obiwan* is a fairly conspicuous member of the *Tetramorium schaufussii* complex, thus easily separable from the other species. As mentioned in the diagnosis above, its larger body size (HW 0.71–0.84; WL 1.00–1.20), strongly developed frontal carinae, relatively long antennal scapes (SI 77–82), relatively large eyes (OI 25–27), lack of median clypeal ruga, and (usually) lack of long, standing pilosity on the waist segments will separate it from the other species of the complex. Except for the relatively long antennal scapes, none of the characters listed is unique to *Tetramorium obiwan*, but the combination renders its identification very straightforward. Still, in a few cases it is possible to mistake *Tetramorium obiwan* at first glance for *Tetramorium merina* or *Tetramorium nassonowii*, which are also larger species. Nevertheless, *Tetramorium merina* has noticeably much weaker frontal carinae than *Tetramorium obiwan*. *Tetramorium nassonowii* is likely the species closest to *Tetramorium obiwan* but they differ significantly in the shape of the petiolar node. The node of *Tetramorium obiwan* is higher and broader, in profile around 1.6 to 1.9 times higher than long (LPeI 54–64), and in dorsal view around 1.1 to 1.3 times wider than long (DPeI 109–129), whereas the node of *Tetramorium nassonowii* is relatively long and low, in profile around 1.2 to 1.4 times higher than long (LPeI 72–81), and in dorsal view between 1.0 to 1.2 times longer than wide (DPeI 87–98). Despite being that distinctive in its own species complex, *Tetramorium obiwan* is comparatively similar to *Tetramorium proximum* from the *Tetramorium cognatum* species complex in that they are both large, reddish-brown species with relatively well-developed frontal carinae and a high rounded nodiform petiolar node. Nevertheless, the presence of long, standing pilosity on the first gastral tergite easily distinguishes *Tetramorium obiwan* from *Tetramorium proximum* that lacks any standing pilosity on the first gastral tergite.

Intraspecific variation in *Tetramorium obiwan* seems relatively low, especially considering that the species is fairly widely distributed.

###### Tetramorium pseudogladius

Hita Garcia & Fisher sp. n.

http://zoobank.org/4C29B150-F567-4F4D-AB0F-062EA2D2EDCD

http://species-id.net/wiki/Tetramorium_pseudogladius

Figs 41A , 54 , 65

####### Type material.

**Holotype**, pinned worker, MADAGASCAR, Toamasina, Parc National de Zahamena, Tetezambatana forest, near junction of Nosivola and Manakambahiny Rivers, 17.74298°S, 48.72936°E, 860 m, rainforest, sifted litter (leaf mold, rotten wood), collection code BLF21974, 18.–19.II.2009 (*B.L. Fisher et al.*) (CAS: CASENT0153605).

**Figure 54.**
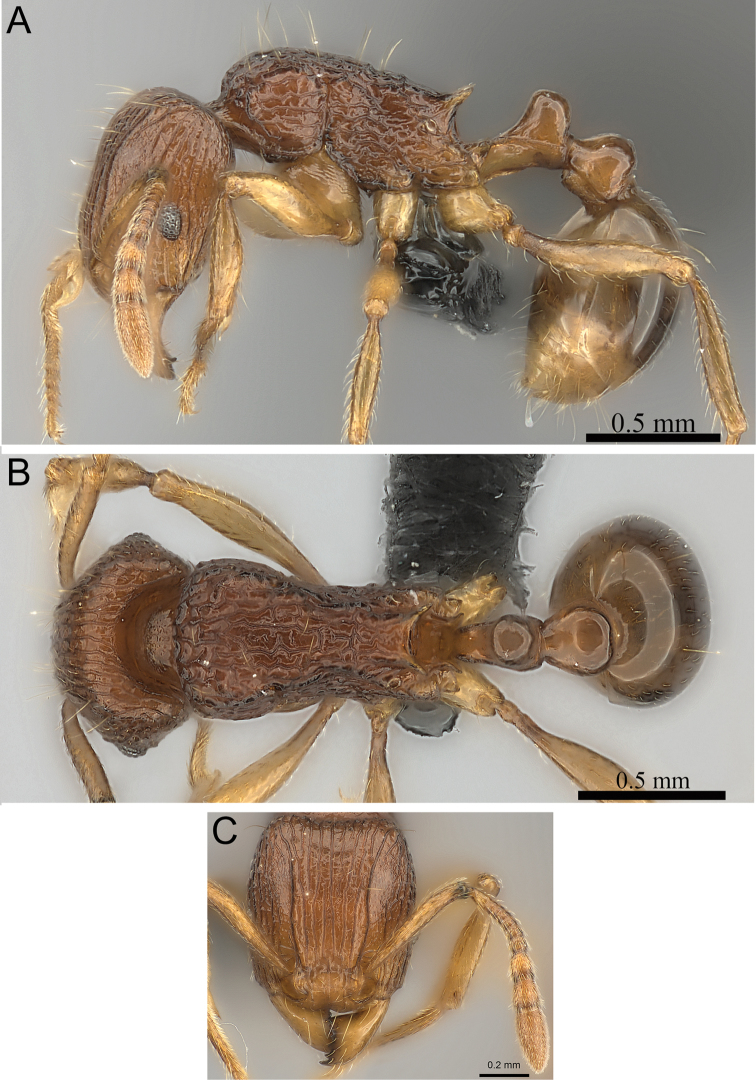
*Tetramorium pseudogladius* holotype worker (CASENT0153605). **A** Body in profile **B** Body in dorsal view **C** Head in full-face view.

####### Diagnosis.

*Tetramorium pseudogladius* can be easily separated from the remainder of the species complex by its relatively small eyes (OI 20).

####### Worker measurements

**(N=1).** HL 0.74; HW 0.68; SL 0.55; EL 0.14; PH 0.36; PW 0.53; WL 0.96; PSL 0.16; PTL 0.14; PTH 0.27; PTW 0.19; PPL 0.21; PPH 0.28; PPW 0.27; CI 91; SI 80; OI 20; DMI 55; LMI 38; PSLI 22; PeNI 35; LPeI 52; DPeI 132; PpNI 51; LPpI 75; DPpI 129; PPI 146.

####### Worker description.

Head longer than wide (CI 91); in full-face view posterior head margin weakly to moderately concave. Anterior clypeal margin with distinct median impression. Frontal carinae well developed, diverging posteriorly, approaching posterolateral corners of head. Antennal scrobes very weak, shallow and without clear and distinct posterior and ventral margins. Antennal scapes of moderate length, not reaching posterior head margin (SI 80). Eyes relatively small (OI 20). Mesosomal outline in profile relatively flat, moderately low and long (LMI 38), moderately marginate from lateral to dorsal mesosoma; promesonotal suture absent; metanotal groove mostly reduced. Propodeal spines moderately long, elongate-triangular to spinose, acute (PSLI 22), propodeal lobes short and triangular, much shorter than propodeal spines, spines and lobes not strongly inclined towards each other. Petiolar node in profile high rounded nodiform, around 1.9 times higher than long (LPeI 52), anterior and posterior faces approximately parallel, anterodorsal and posterodorsal margins situated at about same height and weakly rounded, petiolar dorsum weakly convex; node in dorsal view around 1.3 times wider than long (DPeI 132); in dorsal view pronotum around 2.8 to 2.9 times wider than petiolar node (PeNI 35). Postpetiole in profile globular, around 1.3 times higher than long (LPpI 75); in dorsal view around 1.3 times wider than long (DPpI 129); in dorsal view pronotum around 1.9 to 2.0 times wider than postpetiole (PpNI 51). Postpetiole in profile appearing more or less of same volume as petiolar node, postpetiole in dorsal view around 1.4 to 1.5 times wider than petiolar node (PPI 146). Mandibles completely unsculptured, smooth, and shiny; clypeus longitudinally rugulose with three similarly weak but unbroken rugulae; cephalic dorsum between frontal carinae longitudinally rugose with six or seven rugae, rugae running mostly unbroken from posterior clypeal margin to posterior head margin, a few rugae interrupted or with cross-meshes; scrobal area partly unsculptured, but mostly merging with surrounding reticulate-rugose to longitudinally rugose sculpture present on lateral head; ground sculpture on head weakly to moderately punctate. Dorsum and sides of mesosoma mostly irregularly longitudinally rugose; forecoxae mostly unsculptured, smooth, and shining; ground sculpture on mesosoma very weak to absent. Both waist segments and gaster completely unsculptured, smooth, and shining. Dorsum of head with several pairs of long, fine, standing hairs; dorsum of mesosoma with six pairs restricted to pronotum and mesonotum, propodeum without standing pilosity; waist segments and first gastral tergite without any standing hairs; first gastral tergite with very short, scarce, appressed pubescence in combination with a few scarce, long, standing hairs. Anterior edges of antennal scapes and dorsal (outer) surfaces of hind tibiae with appressed to decumbent hairs. Head, mesosoma, waist segments and gaster uniformly reddish, orange-brown contrasting with lighter yellowish to light brown mandibles, antennae, and legs.

####### Etymology.

The name of the new species is a combination of the ancient Greek word “pseudes”, which means “false” or “lying”, and the species name of *Tetramorium gladius* from the *Tetramorium cognatum* complex. This combined name takes account for the fact that both species are almost identical in morphology. The species epithet is treated as a nominative noun, and is thus invariant.

####### Distribution and biology.

At present, *Tetramorium pseudogladius* is known only from the type locality, Parc National de Zahamena (Fig. 65) where it was collected in lowland rainforest at an altitude of 860 m. In addition, the new species was sampled from leaf litter.

####### Discussion.

Like *Tetramorium gladius* in the *Tetramorium cognatum* complex, *Tetramorium pseudogladius* is also immediately recognisable on the basis of its much smaller eyes (OI 20). The other species of the *Tetramorium schaufussii* complex all have much larger eyes (OI 22–28). In addition, *Tetramorium pseudogladius* lacks the long, standing pilosity on the waist segments present in most other members of the species complex, and has relatively long antennal scapes (SI 80).

Generally, *Tetramorium pseudogladius* looks very similar to *Tetramorium gladius* in the *Tetramorium cognatum* species complex and they also share most of their morphometric range. Indeed, if not for the few long, standing hairs on its first gastral tergite, the holotype of *Tetramorium pseudogladius* could be easily confused with *Tetramorium gladius*. They also differ in antennal scape length, however, which is longer in *Tetramorium pseudogladius* (SI 80) than in *Tetramorium gladius* (SI 71–74). Until more material from the type locality becomes available, we consider these differences as diagnostic to delimit the species boundary between these two species.

###### Tetramorium rala

Hita Garcia & Fisher sp. n.

http://zoobank.org/60FD1630-6771-49E0-8C47-4FFCE28FA02C

http://species-id.net/wiki/Tetramorium_rala

Figs 43A , 55 , 65

####### Type material.

**Holotype**, pinned worker, MADAGASCAR, Antsiranana, Parc National de Marojejy, Manantenina River, 28.0 km 38° NE Andapa, 8.2 km 333° NNW Manantenina, 14.43667°S, 49.775°E, 450 m, rainforest, sifted litter (leaf mold, rotten wood), collection code BLF08722, 12.–15.XI.2003 (*B.L. Fisher et al.*) (CAS: CASENT0046163). **Paratypes**, five pinned workers with same data as holotype (BMNH: CASENT0046035; CAS: CASENT0045975; CASENT0046039; CASENT0046049; MCZ: CASENT0046176); and nine pinned workers with same data as holotype except sampled from rotten log and collection code BLF08772 (CAS: CASENT0077304; CASENT0077305; CASENT0077306).

**Figure 55.**
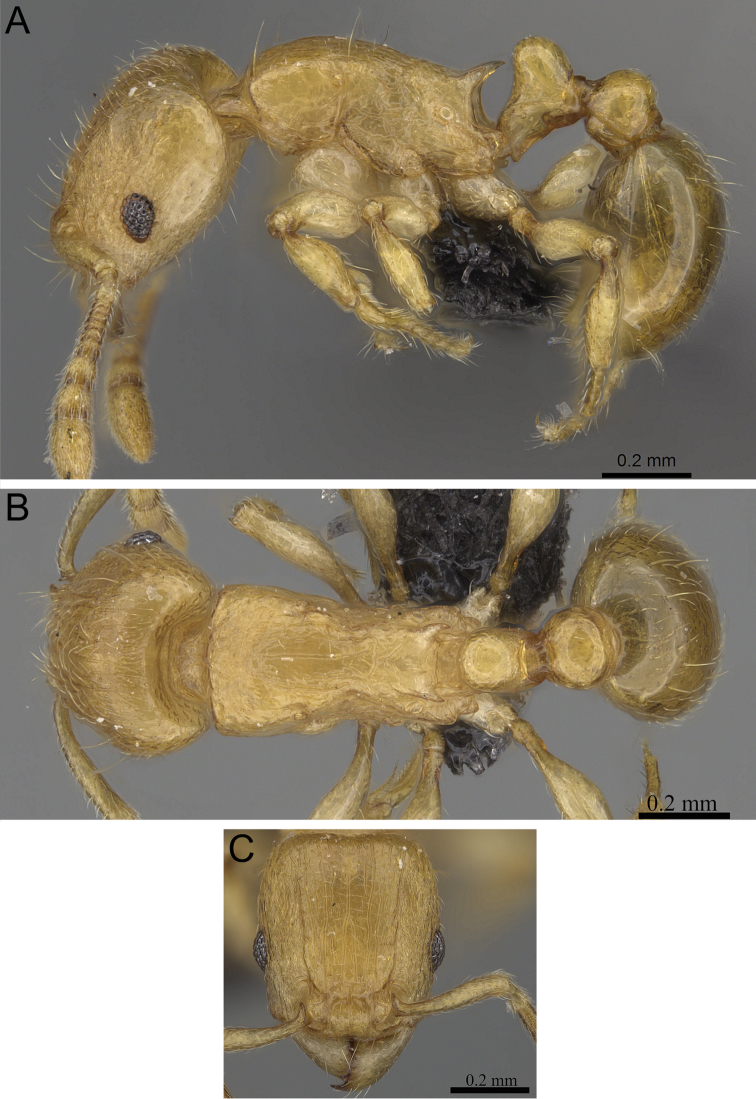
*Tetramorium rala* holotype worker (CASENT0046163). **A** Body in profile **B** Body in dorsal view **C** Head in full-face view.

####### Non-type material.

MADAGASCAR: Antsiranana, Cap Masoala, 19 km W Andampibe, 15.69361°S, 50.18167°E, 125 m, lowland rainforest, 2.XII.1992 (*G.A. Alpert*); Antsiranana, Makirovana forest, 14.17066°S, 49.95409°E, 415 m, rainforest, 28.–29.IV.2011 (*B.L. Fisher et al.*); Antsiranana, Makirovana forest, 14.16044°S, 49.95216°E, 550 m, rainforest, 1.V.2011 (*B.L. Fisher et al.*); Antsiranana, Parc National de Marojejy, Manantenina River, 28.0 km 38° NE Andapa, 8.2 km 333° NNW Manantenina, 14.43667°S, 49.775°E, 450 m, rainforest, 12.–15.XI.2003 (*B.L. Fisher et al.*); Antsiranana, 15 km S Sambava, 10 m, 19.XII.1972 (J.-M. Bentsch); Fianarantsoa, Réserve Speciale Manombo 24.5 km 228° Farafangana, 23.01583°S, 47.719°E, 30 m, rainforest, 21.IV.2005 (*B.L. Fisher et al.*); Toamasina, 6.3 km S Ambanizana, Andranobe, 15.6813°S, 49.958°E, 25 m, rainforest, 14.XI.1993 (*B.L. Fisher*); Toamasina, 5.3 km SSE Ambanizana, Andranobe, 15.67133°S, 49.97395°E, 425 m, rainforest, 21.XI.1993 (*B.L. Fisher*); Toamasina, Réserve Spéciale Ambatovaky, Sandrangato river, 16.77274°S, 49.26551°E, 450 m, rainforest, 20.–22.II.2010 (*B.L. Fisher et al.*); Toamasina, Réserve Spéciale Ambatovaky, Sandrangato river, 16.76912°S, 49.26704°E, 475 m, rainforest, 21.II.2010 (*B.L. Fisher et al.*); Toamasina, Réserve Spéciale Ambatovaky, Sandrangato river, 16.7633°S, 49.26692°E, 520 m, rainforest, 22.II.2010 (*B.L. Fisher et al.*); Toamasina, 19 km ESE Maroantsetra, 15.48333°S, 49.9°E, 300 m, rainforest, 22.IV.1989 (*P.S. Ward*); Toamasina, Nosy Mangabe, 15.5°S, 49.76667°E, 20–50 m, rainforest, 20.IV.1989 (*P.S. Ward*); Toamasina, F.C. Sandranantitra, 18.04833°S, 49.09167°E, 450 m, rainforest, 18.–21.I.1999 (*H.J. Ratsirarson*); Toamasina, S.F. Tampolo, 10 km NNE Fenoarivo Atn., 17.2825°S, 49.43°E, 10 m, littoral rainforest, 4.IV.1997 (*B.L. Fisher*).

####### Diagnosis.

The following character combination distinguishes *Tetramorium rala* from the remainder of the *Tetramorium schaufussii* species complex: relatively small species (HW 0.46–0.49; WL 0.58–0.64); eyes relatively large (OI 26–28); antennal scapes very short (SI 61–68); frontal carinae usually very weakly developed, only feebly raised, usually ending shortly after posterior eye margin or merging with cephalic sculpture halfway between posterior eye margin and posterior head margin; propodeal spines medium-sized to long, elongate-triangular to spinose, and acute (PSLI 21–25), propodeal lobes short, triangular, and acute or blunt, but always much shorter than propodeal spines, spines and lobes not strongly inclined towards each other; petiolar node in profile high rounded nodiform to thinly cuneiform, in profile 2.0 to 2.2 times higher than long (LPeI 45–50), in dorsal view around 1.3 to 1.5 times broader than long (DPeI 129–145); mesosoma with three to four pairs on dorsal promesonotum, propodeum and waist segments without any standing pilosity; body uniformly whitish yellow to light brown.

####### Worker measurements

**(N=15).** HL 0.49–0.53 (0.51); HW 0.46–0.49 (0.47); SL 0.28–0.33 (0.31); EL 0.12–0.13 (0.13); PH 0.22–0.24 (0.23); PW 0.32–0.37 (0.35); WL 0.58–0.64 (0.61); PSL 0.11–0.13 (0.12); PTL 0.09–0.11 (0.10); PTH 0.20–0.23 (0.21); PTW 0.13–0.16 (0.14); PPL 0.12–0.14 (0.13); PPH 0.18–0.21 (0.19); PPW 0.18–0.22 (0.19); CI 90–94 (92); SI 61–68 (65); OI 26–28 (27); DMI 52–60 (57); LMI 36–39 (37); PSLI 21–25 (23); PeNI 37–43 (40); LPeI 45–50 (49); DPeI 129–145 (136); PpNI 52–59 (54); LPpI 67–75 (70); DPpI 141–157 (148); PPI 129–142 (138).

####### Worker description.

Head clearly longer than wide (CI 90–94); posterior head margin weakly concave, almost straight. Anterior clypeal margin with distinct median impression. Frontal carinae usually very weakly developed, only feebly raised, usually ending shortly after posterior eye margin or merging with cephalic sculpture halfway between posterior eye margin and posterior head margin. Antennal scrobes very weak to absent, very shallow and without clear and distinct posterior and ventral margins. Antennal scapes very short, not reaching posterior head margin (SI 61–68). Eyes relatively large (OI 26–28). Mesosomal outline in profile flat to weakly convex, comparatively low and long (LMI 36–39), moderately marginate from lateral to dorsal mesosoma; promesonotal suture absent; metanotal groove very weak to absent. Propodeal spines medium-sized, elongate-triangular to spinose, and acute (PSLI 21–25), propodeal lobes short, triangular, and acute or blunt, but always much shorter than propodeal spines, spines and lobes not strongly inclined towards each other. Petiolar node in profile high rounded nodiform to thinly cuneiform, around 2.0 to 2.2 times higher than long (LPeI 45–50), anterior and posterior faces not parallel, anterodorsal margin usually situated higher and more strongly angled than posterodorsal margin, petiolar dorsum relatively flat to weakly convex and tapering backwards posteriorly; node in dorsal view around 1.3 to 1.5 times broader than long (DPeI 129–145), in dorsal view pronotum between 2.3 to 2.7 times wider than petiolar node (PeNI 37–43). Postpetiole in profile globular to subglobular, approximately 1.3 to 1.5 times higher than long (LPpI 67–75); in dorsal view around 1.4 to 1.6 times wider than long (DPpI 141–157), pronotum between 1.7 to 1.9 times wider than postpetiole (PpNI 52–59). Postpetiole in profile lower, thicker, and more rounded than petiolar node, postpetiole in dorsal view around 1.3 to 1.4 times wider than petiolar node (PPI 129–142). Mandibles completely unsculptured, smooth, and shiny; clypeus longitudinally rugulose with three to five rugulae, median rugula always present and usually fully developed, one or two mostly entire, rarely broken, lateral rugulae present on each side; cephalic dorsum between frontal carinae irregularly longitudinally rugulose with seven to ten fine rugulae, rugulae usually running from posterior clypeal margin to posterior head margin, but mostly irregularly shaped, interrupted or with cross-meshes; scrobal area mostly unsculptured; lateral head mainly longitudinally rugulose to reticulate-rugulose, but larger areas often only weakly sculptured and appearing fairly smooth and shiny; ground sculpture on head weakly to moderately punctate. Dorsum of mesosoma ranging from weakly longitudinally rugulose with larger areas with almost completely reduced sculpture to longitudinally rugose with well developed rugae; lateral mesosoma weakly to moderately irregularly longitudinally rugulose or reticulate-rugulose, often with larger areas of almost completely reduced sculpture; ground sculpture on mesosoma usually weak to absent, sometimes moderately punctate. Forecoxae, both waist segments, and gaster fully unsculptured, smooth, and shining. Dorsum of head with several pairs of long, fine, standing hairs; mesosoma with three to four pairs on promesonotum; propodeum and waist segments without any standing pilosity; first gastral tergite with short, moderately dense, appressed (rarely decumbent) pubescence combined with several scattered, long, fine erect to suberect hairs; anterior edges of antennal scapes and dorsal (outer) surfaces of hind tibiae with appressed to decumbent hairs. Body uniformly whitish yellow to light brown.

####### Etymology.

The new species is named after the fictional character “Rala” from Walter Moers’ fantasy novel “Rumo and His Miraculous Adventures”. The species epithet is an arbitrary combination of letter, thus invariable.

####### Distribution and biology.

The distribution range of *Tetramorium rala* is relatively disjunctive (Fig. 65). Its main distribution seems to be in the northeast in the area around the Bay of Antongil and the Masoala Peninsula north to Marojejy and Makirovana. Further south of this main cluster of localities *Tetramorium rala* is only known from three additional places: Ambatovaky, Tampolo, and Manombo. One explanation for this patchy distribution would be a preferense for lowland rainforests. *Tetramorium rala* lives in rainforests and littoral forests at lower elevations ranging from 10 to 550 m where it is found in leaf litter. Very few intact lowland rainforests remain south of its main distribution in the northeast of Madagascar.

####### Discussion.

*Tetramorium rala* is a very distinctive member of the *Tetramorium schaufussii* complex due to its smaller size, relatively long propodeal spines, high nodiform to thinly cuneiform petiolar node, lack of standing pilosity on the waist segments, and very bright body colouration. Consequently, it is very unlikely to be confused with any other species. Most species are much larger in body size, have shorter propodeal spines, and/or possess standing pilosity on the waist segments. Only some smaller specimens of *Tetramorium schaufussii* could at first glance be mistaken with *Tetramorium rala*, but these two species are effortlessly separable. *Tetramorium schaufussii* has much shorter propodeal spines (PSLI 11–18), a lower petiolar node, which in profile is only 1.6 to 1.9 times higher than long (LPeI 52–63), and long, standing pilosity on the waist segments. By contrast, in *Tetramorium rala* the propodeal spines are much longer (PSLI 21–25), the petiolar node is higher, in profile around 2.0 to 2.2 higher than long (LPeI 45–50), and the waist segments lack standing pilosity.

However, even though *Tetramorium rala* is easily identifiable within the *Tetramorium schaufussii* complex, it is morphologically very close to *Tetramorium rumo* from the *Tetramorium cognatum* complex since they share a very similar gestalt. Both species are smaller in size, have relatively longer propodeal spines (compared to most other species of the species group), a high nodiform to thinly cuneiform petiolar node, lack standing pilosity on the waist segments, and possess very bright body colouration. The main separating character, which also places them in separate species complexes, is the lack of standing pilosity on the first gastral tergite in *Tetramorium rumo* versus the presence of standing pilosity on the first gastral tergite in *Tetramorium rala*. Both species are morphologically closer to each other than to any other species of the *Tetramorium schaufussii* species group, but there are highly diagnostic differences to support their heterospecificity. The petiolar node of *Tetramorium rumo* is thinner and stronger anteroposteriorly compressed, in profile around 2.3 to 2.7 times higher than long (LPeI 37–43), and in dorsal view between 1.5 to 1.7 times wider than long (DPeI 156–171), whereas *Tetramorium rala* has a node which in profile is 2.0 to 2.2 times higher than long (LPeI 45–50), and in dorsal view around 1.3 to 1.5 times broader than long (DPeI 129–145). In addition, *Tetramorium rumo* also has larger eyes (OI 28–31) than *Tetramorium rala* (OI 26–28). Further evidence of their heterospecifity can be deduced from their distribution ranges, which overlap in central-eastern Madagascar, but both species maintain their species-specific characteristic without intermediate forms. In Manombo both species were also found living in sympatry in close proximity.

###### Tetramorium schaufussii

Forel, 1891

http://species-id.net/wiki/Tetramorium_schaufussii

**Figure 56.**
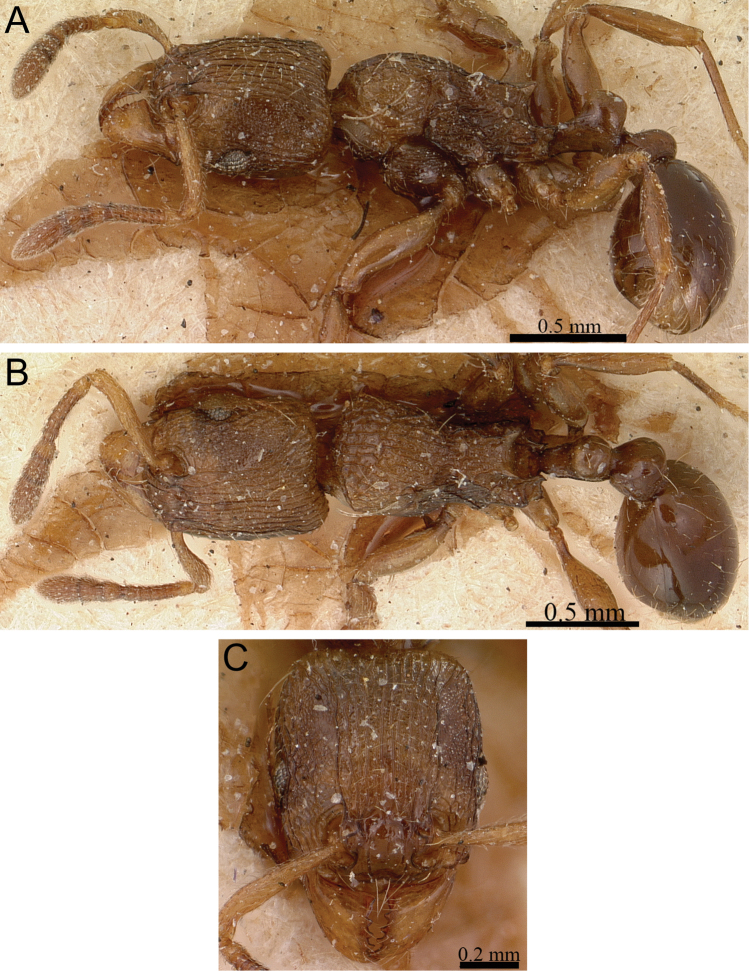
*Tetramorium schaufussii* holotype worker (CASENT0101697). **A** Body in profile **B** Body in dorsal view **C** Head in full-face view.

Figs 42C , 45B , 46C , 47A, B , 56 , 66

Tetramorium schaufussii Forel, 1891: 158.

####### Type material.

**Holotype**, pinned worker, MADAGASCAR, Central Madagascar (*C. Schaufuss*) (MHNG: CASENT0101697).

####### Non-type material.

MADAGASCAR: Antananarivo, Réserve Spéciale d’Ambohitantely, Forêt d’Ambohitantely, Jardin Botanique, 24.1 km 59° NE Ankazobe, 18.17139°S, 47.28182°E, 1620 m, montane rainforest, 17.–22.IV.2001 (*B.L. Fisher et al.*); Antananarivo, 3 km 41° NE Andranomay, 11.5 km 147° SSE Anjozorobe, 18.47333°S, 47.96°E, 1300 m, montane rainforest, 5.–13.XII.2000 (*B.L. Fisher et al.*); Antananarivo, Andrangoloaca (*Sikora*); Antananarivo, Stn. Forestiére Manjakatompo, 19.35°S, 47.31667°E, 1600 m, montane rainforest, 20.II.1993 (*P.S. Ward*); Antsiranana, Réserve Spéciale de l’Ankarana, 13.6 km 192° SSW Anivorano Nord, 12.86361°S, 49.22583°E, 210 m, tropical dry forest, 19.–20.II.2001 (*G.D. Alpert*); Antsiranana, Parc National Montagne d’Ambre, Ambre grand lac, 12.59656°S, 49.15932°E, 1350 m, montane rainforest, 13.XI.2007 (*B.L. Fisher et al.*); Antsiranana, Parc National Montagne d’Ambre, Lac Maudit, 12.58502°S, 49.15147°E, 1250 m, montane rainforest, 13.–14.XI.2007 (*B.L. Fisher et al.*); Antsiranana, Parc National Montagne d’Ambre, 12.2 km 211° SSW Joffreville, 12.59639°S, 49.1595°E, 1300 m, montane rainforest, 2.–7.II.2001 (*B.L. Fisher et al.*); Antsiranana, Parc National Montagne d’Ambre, 12.53417°S, 49.17607°E, 1325 m, montane rainforest, 12.III.2011 (*B.L. Fisher et al.*); Antsiranana, Rés. Anjanaharibe-Sud, 9.2 km WSW Befingotra, 14.75°S, 49.46667°E, 1200 m, montane rainforest, 9.–11.XI.1994 (*B.L. Fisher*); Antsiranana, Rés. Anjanaharibe-Sud, 11.0 km WSW Befingotra, 14.75°S, 49.45°E, 1550–1565 m, montane rainforest, 15.–21.XI.1994 (*B.L. Fisher*); Antsiranana, Forêt de Binara, 9.4 km 235° SW Daraina, 13.26333°S, 49.6°E, 1100 m, montane rainforest, 5.XII.2003 (*B.L. Fisher*); Antsiranana, Makirovana forest, 14.16666°S, 49.95°E, 715 m, rainforest, 2.V.2011 (*B.L. Fisher et al.*); Antsiranana, R.S. Manongarivo, 12.8 km 228° SW Antanambao, 13.97667°S, 48.42333°E, 780 m, rainforest, 11.–17.X.1998 (*B.L. Fisher*); Antsiranana, R.S. Manongarivo, 14.5 km 220° SW Antanambao, 13.99833°S, 48.42833°E, 1175 m, montane rainforest, 19-25.X.1998 (*B.L. Fisher*); Antsiranana, R.S. Manongarivo, 17.3 km 218° SW Antanambao, 14.02167°S, 48.41833°E, 1580 m, montane rainforest, 27.X.1998 (*B.L. Fisher*); Antsiranana, R.S. Manongarivo, 20.4 km 219° SW Antanambao, 14.04667°S, 48.40167°E, 1860 m, montane rainforest, 3.XI.1998 (*B.L. Fisher*); Antsiranana, R.N.I. Marojejy, 11 km NW Manantenina, 14.45°S, 49.73333°E, 1875 m, montane rainforest, 13.–19.XI.1996 (*E.L. Quinter*); Antsiranana, R.N.I. Marojejy, 11 km NW Manantenina, 14.43333, 49.75°E, 1225 m, montane rainforest, 25.X.–19.XI.1996 (*E.L. Quinter*); Antsiranana, Parc National de Marojejy, Manantenina River, 28.0 km 38° NE Andapa, 8.2 km 333° NNW Manantenina, 14.43667°S, 49.775°E, 450 m, rainforest, 12.–15.XI.2003 (*B.L. Fisher et al.*); Antsiranana, Parc National de Marojejy, Antranohofa, 26.6 km 31° NNE Andapa, 10.7 km 318° NW Manantenina, 14.44333°S, 49.74333°E, 1325 m, montane rainforest, 19.–20.XI.2003 (*B.L. Fisher*); Antsiranana, Parc National de Marojejy, 25.7 km 32° NNE Andapa, 10.3 km 314° NW Manantenina, 14.445°S, 49.74167°E, 1575 m, montane rainforest, 21.–22.XI.2003 (*B.L. Fisher*); Fianarantsoa, 28 km SSW Ambositra, 20.76667°S, 47.18333°E, 1660 m, rainforest, 29.IV.1989 (*P.S. Ward*); Fianarantsoa, 27.4 km SSW Ambositra, 20.77°S, 47.18667°E, 1600 m, montane rainforest, 15.I.1998 (*B.L. Fisher*); Fianarantsoa, 26.8 km SW Ambositra, Parc naturel communautaire, 20.775°S, 47.18362°E, 1755 m, disturbed montane rainforest, 20.V.2008 (*B.L. Fisher et al.*); Fianarantsoa, Rés. Andringitra, 43 km S Ambalavao, 22.23333°S, 47°E, 825 m, rainforest, 5.X.1993 (*B.L. Fisher*); Fianarantsoa, Rés. Andringitra, 38 km S Ambalavao, 22.2°S, 46.96667°E, 1680 m, montane rainforest, 23.X.1993 (*B.L. Fisher*); Fianarantsoa, P. N. Andringitra, Forêt Ravaro 12.5 km SW Antanifotsy, 22.21167°S, 46.845°E, 1650 m, montane rainforest, 10.–15.I.2000 (*S. Razafimandimby*); Fianarantsoa, Forêt d’Atsirakambiaty, 7.6 km 285° WNW Itremo, 20.59333°S, 46.56333°E, 150 m, montane rainforest, 22.–26.I.2003 (*B.L. Fisher et al.*); Fianarantsoa, Parc National Befotaka-Midongy, Papango 27.7 km S Midongy-Sud, Mount Papango, 23.83517°S, 46.96367°E, 940 m, rainforest, 13.–19.XI.2006 (*B.L. Fisher et al.*); Fianarantsoa, Parc National Befotaka-Midongy, Papango 28.5 km S Midongy-Sud, Mount Papango, 23.84083°S, 46.9575°E, 1250 m, montane rainforest, 17.–18.2006 (*B.L. Fisher et al.*); Fianarantsoa, R.S. Ivohibe, 6.5 km ESE Ivohibe, 22.49667°S, 46.955°E, 1575 m, montane rainforest, 24.–30.X.1997 (*B.L. Fisher*); Fianarantsoa, 7 km W Ranomafana, 1100 m, montane rainforest, 1.–7.I.1988 (*W.E. Steiner*); Fianarantsoa, Ranomafana National Park, Maharira forest, 21°S, 47°E, 1375 m, montane rainforest, 12.X.1992 (*E. Rajeriarison*); Fianarantsoa, Ranomafana National Park, Maharira forest, 21°S, 47°E, 1130 m, montane rainforest, 29.XII.1992 (*E. Rajeriarison*); Fianarantsoa, Parc National de Ranomafana, Vatoharanana River, 4.1 km 231° SW Ranomafana, 21.29°S, 47.43333°E, 1100 m, montane rainforest, 27.–31.III.2003 (*B.L. Fisher et al.*); Fianarantsoa, Parc National de Ranomafana, Sahamalaotra River, 6.6 km 310° NW Ranomafana, m, 21.23667°S, 47.39667°E, 1150 m, montane rainforest, 31.III.2003 (*B.L. Fisher et al.*); Mahajanga, Réserve Spéciale Marotandrano, Marotandrano 48.3 km S Mandritsara, 16.28322°S, 48.81443°E, 865 m, transition humid forest, 7.XII.2007 (*B.L. Fisher et al.*); Toamasina, 6.9 km NE Ambanizana, Ambohitsitondroina, 15.58506°S, 50.00952°E, 825 m, rainforest, 2.XII.1992 (*B.L. Fisher*); Toamasina, Réserve Spéciale Ambatovaky, Sandrangato river, 16.76912°S, 49.26704°E, 475 m, rainforest, 21.II.2010 (*B.L. Fisher et al.*); Toamasina, Réserve Spéciale Ambatovaky, Sandrangato river, 16.7674°S, 49.26813°E, 500 m, rainforest, 23.II.2010 (*B.L. Fisher et al.*); Toamasina, Réserve Spéciale Ambatovaky, Sandrangato river, 16.81745°S, 49.2925°E, 400 m, rainforest, 26.II.2010(*B.L. Fisher et al.*); Toamasina, Analamay, 18.80623°S, 48.33707°E, 1068 m, montane rainforest, 23.III.2004 (*Malagasy ant team*); Toamasina, 6 km ESE Andasibe (=Perinet), 18.95°S, 48.46667°E, 900 m, rainforest, 17.XI.1990 (*P.S. Ward*); Toamasina, Montagne d’Anjanaharibe, 19.5 km 27° NNE Ambinanitelo, 15.17833°S, 49.635°E, 1100 m, montane rainforest, 12.–16.III.2003 (*B.L. Fisher et al.*); Toamasina, Reserve Betampona, Camp Rendrirendry 34.1 km 332° Toamasina, 17.924°S, 49.19967°E, 390 m, rainforest, 29.–30.XI.2005 (*B.L. Fisher et al.*); Toamasina, Parc National de Zahamena, Tetezambatana forest, near junction of Nosivola and Manakambahiny rivers, 17.74298°S, 48.72936°E, 860 m, rainforest, 19.II.2009 (*B.L. Fisher et al.*); Toamasina, Parc National de Zahamena, Onibe River, 17.75908°S, 48.85468°E, 780 m, rainforest, 22.II.2009 (*B.L. Fisher et al.*); Toliara, Parc National d’Andohahela, Col du Sedro, 3.8 km 113° ESE Mahamavo, 37.6 km 341° NNW Tolagnaro, 24.76389°S, 46.75167°E, 900 m, montane rainforest, 21.–25.I. (*B.L. Fisher et al.*); Toliara, Réserve Spéciale Kalambatritra, Betanana, 23.4144°S, 46.459°E, 1360 m, montane rainforest, 8.II.2010 (*B.L. Fisher et al.*); Toliara, Réserve Spéciale Kalambatritra, Ambinanitelo, 23.4502°S, 46.45658°E, 1325 m, montane rainforest, 11.II.2010 (*B.L. Fisher et al.*); Toliara, Réserve Spéciale Kalambatritra, Ampanihy, 23.4635°S, 46.4631°E, 1270 m, montane rainforest, 9.–10.II.2010 (*B.L. Fisher et al.*); REUNION: no locality data (*G. Mayr*).

####### Diagnosis.

The following character combination separates *Tetramorium schaufussii* from the remaining species of the *Tetramorium schaufussii* complex: head much longer than wide (CI 86–90); antennal scapes short to very short (SI 66–73); eyes moderate to large (OI 24–28); frontal carinae usually moderately well developed, slightly diverging posteriorly, and typically fading out or merging with surrounding sculpture halfway between posterior eye and posterior head margin; propodeal spines usually short, triangular to elongate-triangular (PSLI 11–18), propodeal lobes short and triangular, usually of approximately same length as propodeal spines, sometimes lobes longer, rarely much shorter than spines, but spines and lobes never strongly inclined towards each other; petiolar node in profile high rounded nodiform, around 1.6 to 1.9 times higher than long (LPeI 52–63), in dorsal view between 1.1 to 1.4 times wider than long (DPeI 112–136); propodeum without standing pilosity; petiole and postpetiole with long, standing pilosity.

####### Worker measurements

**(N=60).** HL 0.59–0.76 (0.67); HW 0.51–0.68 (0.60); SL 0.36–0.50 (0.42); EL 0.14–0.19 (0.16); PH 0.26–0.35 (0.31); PW 0.38–0.50 (0.45); WL 0.70–0.93 (0.82); PSL 0.07–0.14 (0.10); PTL 0.12–0.17 (0.14); PTH 0.21–0.29 (0.25); PTW 0.15–0.20 (0.17); PPL 0.15–0.22 (0.18); PPH 0.20–0.28 (0.24); PPW 0.21–0.28 (0.25); CI 86–90 (88); SI 66–73 (71); OI 24–28 (26); DMI 52–59 (54); LMI 35–39 (37); PSLI 11–18 (15); PeNI 35–41 (39); LPeI 52–63 (57); DPeI 112–136 (124); PpNI 53–61 (56); LPpI 69–83 (75); DPpI 125–150 (137); PPI 131–153 (142).

####### Worker description.

Head much longer than wide (CI 86–90); posterior head margin weakly concave. Anterior clypeal margin with distinct median impression. Frontal carinae usually moderately well developed, slightly diverging posteriorly, and typically fading out or merging with surrounding sculpture halfway between posterior eye and posterior head margin. Antennal scrobes weak to absent, shallow and without clear and distinct posterior and ventral margins. Antennal scapes short to very short, not reaching posterior head margin (SI 66–73). Eyes moderate to large (OI 24–28). Mesosomal outline in profile flat to weakly convex, comparatively low and long (LMI 35–39), moderately marginate from lateral to dorsal mesosoma; promesonotal suture absent; metanotal groove either weakly developed or absent. Propodeal spines usually short, triangular to elongate-triangular (PSLI 11–18), propodeal lobes short and triangular, usually of approximately same length as propodeal spines, sometimes lobes longer, rarely much shorter than spines, but never strongly inclined towards each other. Petiolar node in profile high rounded nodiform, around 1.6 to 1.9 times higher than long (LPeI 52–63), anterior and posterior faces approximately parallel, usually anterodorsal and posterodorsal margins situated at about same height and equally angled, sometimes anterodorsal margin slightly higher, petiolar dorsum generally weakly convex, sometimes flat; node in dorsal view between 1.1 to 1.4 times wider than long (DPeI 112–136), in dorsal view pronotum between 2.4 to 2.8 times wider than petiolar node (PeNI 35–41). Postpetiole in profile globular, around 1.2 to 1.4 times higher than long (LPpI 69–83); in dorsal view between 1.2 to 1.5 times wider than long (DPpI 125–150), pronotum between 1.6 to 1.9 times wider than postpetiole (PpNI 53–61). Postpetiole in profile appearing more or less of similar volume as petiolar node, postpetiole in dorsal view around 1.3 to 1.5 times wider than petiolar node (PPI 131–153). Mandibles completely unsculptured, smooth, and shiny; clypeus longitudinally rugulose/rugose with three to six usually regularly shaped and unbroken rugulae/rugae, median ruga usually fully developed and distinct, very rarely broken, one or two lateral rugulae/rugae present on each side; cephalic dorsum between frontal carinae longitudinally rugulose/rugose with seven to ten rugae/rugulae, rugae/rugulae usually running from posterior clypeal margin to posterior head margin, often irregularly shaped, interrupted, or with cross-meshes; scrobal area partly unsculptured and merging with surrounding sculpture; lateral head reticulate-rugose to longitudinally rugose, often posteriorly mostly unsculptured. Ground sculpture on head usually well developed, moderately reticulate-punctate, especially on cephalic dorsum and scrobal area, sometimes ground sculpture much weaker, almost absent. Dorsum of mesosoma usually irregularly longitudinally rugose to reticulate-rugose, sometimes almost completely reticulate-rugose with few longitudinally rugose elements; lateral mesosoma mostly irregularly longitudinally rugose to reticulate-rugose, often lateral pronotum weaker sculptured to almost unsculptured. Ground sculpture on mesosoma variably developed, usually weakly to moderately punctate, sometimes very weak or absent. Forecoxae either unsculptured, smooth, and shining or with longitudinally rugulose or reticulate-rugose sculpture on upper half. Both waist segments and gaster fully unsculptured, smooth, and shining. Dorsum of head with several pairs of long, fine, standing hairs; mesosoma with six or more pairs on promesonotum, propodeum without standing pilosity; petiole usually with one or two and postpetiole with two to three pairs of long, standing hairs; first gastral tergite with short, moderately dense, appressed pubescence in combination with several scattered to numerous, long, standing hairs. Anterior edges of antennal scapes and dorsal (outer) surfaces of hind tibiae with appressed to decumbent hairs. Head, mesosoma, waist segments, and gaster uniformly light yellowish brown to very dark brown, almost black contrasting with lighter yellowish to light brown mandibles, antennae, and legs.

####### Distribution and biology.

*Tetramorium schaufussii* is broadly distributed throughout most of the humid forest zones from the southeast to the north of Madagascar (Fig. 66). Surprisingly, *Tetramorium schaufussii* is also known from one very old collection event from the island of Reunion, but apart from that no modern collection exists from that island. Despite that we list Reunion as record for *Tetramorium schaufussii* we consider that record as problematic and highly questionable. In Madagascar *Tetramorium schaufussii* is almost always found in rainforests or montane rainforests at elevations from 210 to 1875 m, even though most of the material was collected at elevations higher than 1000 m. In addition, *Tetramorium schaufussii* seems to be an inhabitant of the leaf litter stratum.

####### Discussion.

*Tetramorium schaufussi* is the most widespread, common, and abundant species of the *Tetramorium schaufussi* complex. Also, *Tetramorium schaufussii* co-occurs with all other members of the complex throughout its range. It can be well separated from most species, but its separation from a few morphologically close forms is more challenging. Due to its larger eyes (OI 24–28) *Tetramorium schaufussii* is very unlikely to be mistaken for *Tetramorium pseudogladius* (OI 20). Additionally, its petiolar node shape, which is always broader than long (DPeI 112–136), distinguishes it from *Tetramorium nassonowii*, which has a longer than broad node (DPeI 87–98). The species *Tetramorium scutum*, despite being morphologically relatively similar, has longer propodeal spines (PSLI 22–24) and the spines and propodeal lobes are strongly inclined towards each other. *Tetramorium schaufussii* always has shorter spines (PSLI 11–18) and the spines and lobes are never strongly inclined towards each other. *Tetramorium rala* and *Tetramorium sikorae* lack long, standing pilosity on the waist segments, separating them easily from *Tetramorium schaufussii*, but is should be noted that without consideration of the pilosity on the waist segments, *Tetramorium sikorae* is generally very similar to *Tetramorium schaufussii*. The difference, however, is very constant and both are usually found in sympatry, leading us to the decision to retain them as two species. Furthermore, most of the material of *Tetramorium obiwan* also lacks pilosity on the waist segments, distinguishing it from *Tetramorium schaufussii*, and, if pilosity is present, it is reduced to one pair on the petiole or postpetiole. Additionally, *Tetramorium obiwan* possesses longer antennal scapes (SI 77–82) and is much larger (HW 0.71–0.84; WL 1.00–1.20) than *Tetramorium schaufussii* (SI 66–73; HW 0.51–0.68; WL 0.70–0.93). In *Tetramorium* body size is usually not useful and often even misleading due to high intraspecific variation in many species (Hita Garcia et al. 2010; Hita Garcia and Fisher 2012a), but in the case of these two species we can confidently separate them by size.

The three species *Tetramorium merina*, *Tetramorium monticola*, and *Tetramorium xanthogaster* are often difficult to separate from *Tetramorium schaufussii*. The most important discriminating difference between these three and *Tetramorium schaufussii* is the shape of the head. In *Tetramorium schaufussii* the head is usually much longer and thinner (CI 86–90 vs. CI 91–95 in the other three species). Additionally, in *Tetramorium schaufussii* the clypeus almost always has a very distinct and unbroken median longitudinal ruga, whereas *Tetramorium merina*, *Tetramorium monticola*, and *Tetramorium xanthogaster* normally have the median area fully unsculptured without any median ruga/rugula at all, or the median ruga/rugula is present, but then it is usually interrupted, very irregularly shaped, or only present as traces. This character has to be treated with caution however, as there are a few specimens in *Tetramorium merina*, *Tetramorium monticola*, and *Tetramorium xanthogaster*, in which the median ruga/rugula is present. The delimitations of *Tetramorium merina* and *Tetramorium schaufussii* can be difficult, but in areas where they co-occur in the Central Highlands of Madagascar they can be easily separated by body size. In that area *Tetramorium merina* is always much larger in size and has shorter propodeal spines (PSLI 7–11) than *Tetramorium schaufussii* (PSLI 11–18).

It must be pointed out that *Tetramorium schaufussii* is highly variable, likely the most variable species of the *Tetramorium schaufussii* complex and the whole species group. There are several important characters varying significantly throughout the distribution range that need to be addressed here. The frontal carinae are moderately well developed in most of the material, but in one series from Mt. Anjanaharibe they are reduced and much weaker. As do several other species of the group, *Tetramorium schaufussii* possesses a comparatively variable petiolar node shape. It is generally high rounded nodiform with the anterodorsal and posterodorsal margins situated at about the same height and both equally rounded or angled. However, the node is often lower and thicker, and this variation can be seen within the same series or population. Sometimes the node also has a slightly higher and more angled anterodorsal margin in comparison to the posterodorsal margin, even though only weakly so. In addition, even though mostly stable, the propodeal spines do occasionally vary. They are usually short to very short (PSLI 11–15), but some specimens from Andranomay, Binara, and Marojejy have longer spines (PSLI 16–18). Also highly variable is colouration that also contains a geographic component. Most of the material from the southeast to the central east is much darker in colour, usually dark brown to almost black. The material from the north is very variable in colour ranging from very bright yellow (Mt. Anjanaharibe) to almost black (Marojejy), but usually constant on a local scale. Parallel to the colouration, there is also geographic variation observable in the sculpture on the forecoxae. Most of the darker material has the upper part of the forecoxae distinctly sculptured, whereas in most of the brighter material the forecoxae are completely unsculptured. However, this is not always the case, and thus not used for diagnostics.

The series from Mt. Anjanaharibe mentioned above also requires some comments. The specimens are much brighter in color, smaller in size, and have much weaker frontal carinae than the rest of the *Tetramorium schaufussii* material. These are not unlikely to turn out to be a distinct species, but for the moment we keep it as a smaller, brighter geographical variation of *Tetramorium schaufussii*.

###### Tetramorium scutum

Hita Garcia & Fisher sp. n.

http://zoobank.org/7D34D957-AAD0-4C22-81C5-A99EACC35303

http://species-id.net/wiki/Tetramorium_scutum

**Figure 57.**
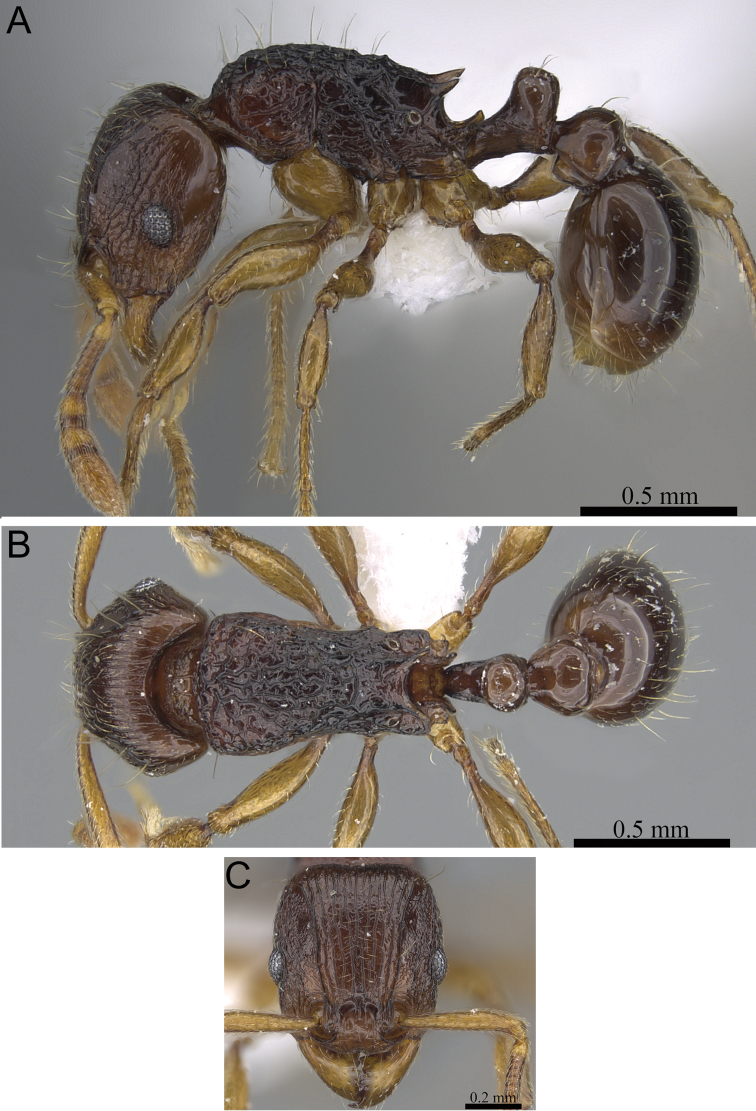
*Tetramorium scutum* holotype worker (CASENT0189116). **A** Body in profile **B** Body in dorsal view **C** Head in full-face view.

Figs 41B , 42A , 57 , 66

####### Type material.

**Holotype**, pinned worker, MADAGASCAR, Fianarantsoa, R.S. Ivohibe 8.0 km E Ivohibe, 22.48333°S, 46.96833°E, 1200 m, montane rainforest, sifted litter (leaf mold, rotten wood), collection code BLF01747, 15.–21.X.1997 (*B.L. Fisher*) (CAS: CASENT0189116). **Paratypes**, five pinned workers with same data as holotype (BMNH: CASENT0218026; CAS: CASENT0218027; CASENT0248303; CASENT0248304; CASENT0248305); five pinned workers from Fianarantsoa, 8.0 km NE Ivohibe, 22.42167°S, 46.89833°E, 1200 m, montane rainforest, sifted litter (leaf mold, rotten wood), collection code BLF01753, 3.–9.XI.1997 (*B.L. Fisher*) (CAS: CASENT0218022; CASENT0218023; CASENT0218028; CASENT0248309; MCZ: CASENT0248308); seven pinned workers from Fianarantsoa, R.S. Ivohibe, 6.5 km ESE Ivohibe, 22.49667°S, 46.955°E, 1575 m, montane rainforest, sifted litter (leaf mold, rotten wood), collection code BLF01751, 24.–30.X.1997 (*B.L. Fisher*) (CAS: CASENT0189154; CASENT0218021; CASENT0218024; CASENT0218025; CASENT0248306; CASENT0248307).

####### Diagnosis.

*Tetramorium scutum* can be separated from the remainder of the species complex by the following character combination: moderate to large eyes (OI 24–25); short antennal scapes (SI 71–74); frontal carinae moderately to well developed, slightly diverging posteriorly, ending at or shortly before posterior head margin; propodeal spines moderate to long (PSLI 22–24), propodeal lobes elongate-triangular, always weakly shorter than propodeal spines, in profile spines and lobes strongly inclined towards each other; petiolar node in profile around 2.0 to 2.1 times higher than long (LPeI 48–50) and in dorsal view around 1.4 to 1.5 times wider than long (DPeI 144–154); dorsum of mesosoma with six or more pairs of long, standing hairs from anterior pronotum to posterior mesonotum, propodeum without any standing pilosity; waist segments with long, standing pilosity.

####### Worker measurements

**(N=10).** HL 0.65–0.70 (0.67); HW 0.60–0.64 (0.61); SL 0.43–0.45 (0.44); EL 0.15–0.16 (0.15); PH 0.31–0.33 (0.32); PW 0.44–0.48 (0.45); WL 0.79–0.86 (0.83); PSL 0.15–0.17 (0.16); PTL 0.12–0.14 (0.13); PTH 0.25–0.28 (0.27); PTW 0.19–0.21 (0.19); PPL 0.18–0.21 (0.19); PPH 0.25–0.29 (0.XX); PPW 0.25–0.29 (0.27); CI 90–92 (91); SI 71–74 (72); OI 24–25 (24); DMI 54–59 (55); LMI 38–39 (38); PSLI 22–24 (23); PeNI 41–44 (43); LPeI 48–50 (49); DPeI 144–154 (148); PpNI 57–62 (59); LPpI 70–74 (72); DPpI 134–140 (138); PPI 133–142 (138).

####### Worker description.

Head longer than wide (CI 90–92); in full-face view posterior head margin weakly concave. Anterior clypeal margin with distinct median impression. Frontal carinae moderately to well developed, slightly diverging posteriorly, ending at or shortly before posterior head margin. Antennal scrobes weak to absent, shallow and without clear and distinct posterior and ventral margins. Antennal scapes short, not reaching posterior head margin (SI 71–74). Eyes moderate to relatively large (OI 24–25). Mesosomal outline in profile flat to weakly convex, comparatively low and long (LMI 38–39), moderately marginate from lateral to dorsal mesosoma; promesonotal suture absent; metanotal groove weak or absent. Propodeal spines moderate to long, elongate-triangular to spinose, and acute (PSLI 22–24); propodeal lobes elongate-triangular, always weakly shorter than propodeal spines, in profile spines and lobes strongly inclined towards each other. Petiolar node in profile high rounded nodiform to weakly squamiform, around 2.0 to 2.1 times higher than long (LPeI 48–50), anterior and posterior faces approximately parallel, anterodorsal and posterodorsal margins situated at about same height, petiolar dorsum relatively flat; node in dorsal view between 1.4 to 1.6 times wider than long (DPeI 144–154), in dorsal view pronotum between 2.2 to 2.5 times wider than petiolar node (PeNI 41–44). Postpetiole in profile subglobular, around 1.3 to 1.4 times higher than long (LPpI 70–74); in dorsal view around 1.3 to 1.4 times wider than long (DPpI 134–140), pronotum between 1.6 to 1.8 times wider than postpetiole (PpNI 57–62). Postpetiole in profile appearing slightly more voluminous than petiolar node, postpetiole in dorsal view around 1.3 to 1.4 times wider than petiolar node (PPI 133–142). Mandibles completely unsculptured, smooth, and shiny; clypeus weakly, irregularly, longitudinally rugulose with median area mostly unsculptured, smooth, and shiny, sometimes median rugula present, but then weakly developed and/or interrupted, one or two weak, irregular and broken rugulae present on each side; cephalic dorsum between frontal carinae longitudinally rugulose with six to nine rugae/rugulae, rugae/rugulae usually running from posterior clypeal margin to posterior head margin, often irregularly shaped, interrupted or with cross-meshes; scrobal area partly unsculptured and merging with surrounding sculpture; lateral head anteriorly reticulate-rugose to longitudinally rugose and posteriorly mostly unsculptured. Ground sculpture on head weakly to moderately reticulate-punctate, especially laterally. Dorsum of mesosoma irregularly longitudinally rugose; lateral mesosoma mostly irregularly longitudinally rugose with some reticulate-rugose elements. Ground sculpture on mesosoma weak to absent. Forecoxae unsculptured, smooth, and shining. Both waist segments and gaster fully unsculptured, smooth, and shining. Dorsum of head with several pairs of long, fine, standing hairs; mesosoma with six or more pairs from anterior pronotum to posterior mesonotum, propodeum without standing pilosity; petiole with one and postpetiole with one or two pairs of long, standing hairs; first gastral tergite with short, moderately dense, appressed pubescence in combination with several scattered, long, standing hairs. Anterior edges of antennal scapes and dorsal (outer) surfaces of hind tibiae with appressed hairs. Head, mesosoma, waist segments and gaster uniformly brown to dark brown contrasting with yellowish to light brown mandibles, antennae, and legs.

####### Etymology.

The name of the new species is Latin and means “shield”, referring to the weakly squamiform condition of the petiolar node. The species epithet is a nominative noun, and thus invariant.

####### Distribution and biology.

This new species is only known from the area around Ivohibe (Fig. 66) where it was collected from a few montane rainforest localities situated at elevations of 1200 to 1575 m. All specimens were sampled from leaf litter.

####### Discussion.

*Tetramorium scutum* is relatively easy to distinguish within the species complex since it is the only species in which the moderately long spines (PSLI 22–22) and the comparatively long propodeal lobes are strongly inclined towards each other. In all the other group members the spines are either much shorter or if they are of approximately similar length as in *Tetramorium scutum*, then the lobes are much smaller and spines and lobes are never strongly inclined towards each other. Interestingly, the species most similar to *Tetramorium scutum* are *Tetramorium aspis* and *Tetramorium camelliae* from the *Tetramorium cognatum* complex, and we suspect that they are also more closely related to each other than to the rest of the group. These three species all have strongly inclined spines and lobes and are also found in the same small area in the southeast of Madagascar.

The new species is only known from a relatively small area and consequently shows very little intraspecific variation.

###### Tetramorium sikorae

Forel, 1892

http://species-id.net/wiki/Tetramorium_sikorae

**Figure 58.**
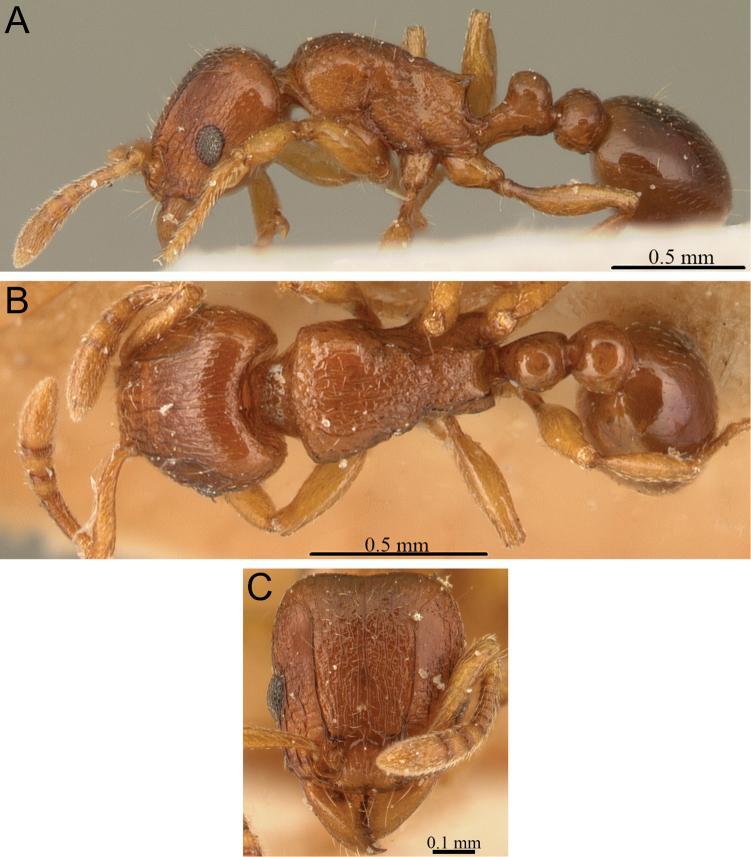
*Tetramorium sikorae* syntype worker of junior synonym *Tetramorium latior* (CASENT0101141). **A** Body in profile **B** Body in dorsal view **C** Head in full-face view.

Figs 41D , 43C , 44B , 45A , 58 , 66

Tetramorium (Xiphomyrmex) sikorae Forel, 1892: 522. [Combination in *Xiphomyrmex*: Emery 1895: 343; in *Tetramorium*: Bolton 1979: 138]
Xiphomyrmex latior Santschi, 1926: 243. [Synonymy with *Tetramorium sikorae* by Bolton 1979: 138; here confirmed]

####### Type material.

Of *Tetramorium sikorae*: **lectotype [designated here**], pinned worker, MADAGASCAR, Toamasina, Amparafaravantsiv, Mangoro Ufer 984, 20°S, 47.08333°E (*Sikora*) (MHNG: CASENT0101260) [examined]. **Paralectotypes [designated here**], three pinned workers with same data as holotype (MHNG: CASENT0101259; CASENT0101898) [examined]. Of *Tetramorium latior*: **syntypes**, six pinned workers, MADAGASCAR, Fianarantsoa, 21.45°S, 47.083333°E (*Descarpentries*) (NHMB: CASENT0101140; CASENT0101141; CASENT0101142; CASENT0101143; CASENT0101144) [examined].

[Note: the GPS data of both type localities was not provided by either locality labels or the original descriptions. The GPS data presented above is based on our own geo-referencing of the locality Amparafara, which is close to the Mangoro River, and the city Fianarantsoa. The data for both locliaties should be considered as approximations and not the exact positions of the type localities.]

####### Non-type material.

MADAGASCAR: no locality, 1.II.1977 (*W.L. & D.E. Brown*); Antsiranana, Forêt Ambanitaza, 26.1 km 347° Antalaha, 14.67933°S, 50.18367°E, 240 m, rainforest, 27.XI.2004 (*B.L. Fisher*); Antsiranana, Res. d’ Anjanaharibe-Sud, 17 km W Andapa, 14.75775°S, 49.51019°E, 875 m, primary rainforest, 7.XI.1994 (*G.D. Alpert*); Antsiranana, Forêt de Binara, 9.4 km 235° SW Daraina, 13.26333°S, 49.6°E, 1100 m, montane rainforest, 6.XII.2003 (*B.L. Fisher*); Antsiranana, Parc National de Marojejy, Manantenina River, 28.0 km 38° NE Andapa, 8.2 km 333° NNW Manantenina, 14.43667°S, 49.775°E, 450 m, rainforest, 12.–15.XI.2003 (*B.L. Fisher et al.*); Antsiranana, Parc National de Marojejy, Manantenina River, 27.6 km 35° NE Andapa, 9.6 km 327° NNW Manantenina, 14.435°S, 49.76°E, 775 m, rainforest, 16.XI.2003 (*B.L. Fisher*); Fianarantsoa, 2 km W Andrambovato, along river Tatamaly, 21.51167°S, 47.41°E, 1075 m, cultivated land (tavy), recent one- to two-year-old tavy, 3.–5.VI.2005 (*B.L. Fisher et al.*); Toamasina, Corridor Forestier Analamay-Mantadia, Ambatoharanana, 18.80388°S, 48.40506°E, 1013 m, rainforest, 12.–19.XII.2012 (*B.L. Fisher et al.*); Toamasina, Parc National d´ Andasibe-Mantadia, Forêt de Mantadia, 25.7 km 248° Moramanga, 18.81402°S, 48.43028°E, 1040 m, rainforest, 13.VII.2006 (*F.N. Raharimalala & B. Blaimer*); Toamasina, Ankerana, 18.4061°S, 48.82029°E, 725 m, rainforest, 20.I.2012 (*B.L. Fisher et al.*); Toamasina, Morarano-Chrome, 25 km W, forest, 15.VI.1991 (*A. Pauly*); Toamasina, Torotorofotsy, 18.87082°S, 48.34737°E, 1070 m, montane rainforest, marsh edge, 27.III.2004 (*Malagasy ant team*); Toliara, Réserve Spéciale d’Ambohijanahary, Forêt d’Ankazotsihitafototra, 35.2 km 312° NW Ambaravaranala, 18.26667°S, 45.40667°E, 1050 m, montane rainforest, 13.–17.III.2003 (*B.L. Fisher et al.*).

####### Diagnosis.

The following character combination distinguishes *Tetramorium sikorae* from the remainder of the *Tetramorium schaufussii* species complex: smaller species (HW 0.53–0.61; WL 0.70–0.82); head much longer than wide (CI 86–89); antennal scapes short (SI 70–74); eyes relatively large (OI 26–28); frontal carinae moderately developed, slightly diverging posteriorly, becoming weaker halfway between posterior eye margin and posterior head margin, ending at or shortly before posterior head margin; propodeal spines reduced to short, triangular teeth (PSLI 11–14), propodeal lobes short, triangular, and usually blunt, approximately of same size as propodeal teeth, and in profile teeth and lobes not strongly inclined towards each other; petiolar node high rounded nodiform, in profile around 1.7 to 1.9 times higher than long (LPeI 53–59), in dorsal view around 1.2 to 1.3 times wider than long (DPeI 120–133); dorsum of mesosoma usually with two, sometimes four, pairs of long, standing hairs on promesonotum; propodeum and waist segments without any long, standing pilosity.

####### Worker measurements

**(N=15).** HL 0.60–0.69 (0.62); HW 0.53–0.61 (0.55); SL 0.38–0.43 (0.39); EL 0.14–0.16 (0.15); PH 0.26–0.31 (0.28); PW 0.39–0.47 (0.42); WL 0.70–0.82 (0.74); PSL 0.07–0.09 (0.08); PTL 0.12–0.15 (0.13); PTH 0.21–0.26 (0.23); PTW 0.16–0.19 (0.17); PPL 0.14–0.17 (0.16); PPH 0.21–0.25 (0.23); PPW 0.21–0.26 (0.23); CI 86–89 (88); SI 70–74 (72); OI 26–28 (27); DMI 53–63 (57); LMI 36–39 (38); PSLI 11–14 (12); PeNI 38–43 (40); LPeI 53–59 (57); DPeI 120–133 (129); PpNI 51–58 (55); LPpI 67–72 (69); DPpI 142–157 (149); PPI 131–144 (137).

####### Worker description.

Head much longer than wide (CI 86–89); in full-face view posterior head margin weakly to moderately concave. Anterior clypeal margin with distinct median impression. Frontal carinae moderately developed, slightly diverging posteriorly, becoming weaker halfway between posterior eye margin and posterior head margin, ending at or shortly before posterior head margin. Antennal scrobes weak to absent, shallow and without clear and distinct posterior and ventral margins. Antennal scapes short, not reaching posterior head margin (SI 70–74). Eyes relatively large (OI 26–28). Mesosomal outline in profile flat to weakly convex, comparatively low and long (LMI 36–39), moderately marginate from lateral to dorsal mesosoma; promesonotal suture absent; metanotal groove weak or absent. Propodeal spines reduced to short, triangular, and usually blunt, rarely acute, teeth (PSLI 11–14); propodeal lobes short, triangular, and usually blunt, approximately of same size as propodeal teeth, in profile teeth and lobes not strongly inclined towards each other. Petiolar node in profile high rounded nodiform, around 1.7 to 1.9 times higher than long (LPeI 53–59), anterior and posterior faces approximately parallel, usually anterodorsal and posterodorsal margins situated at about same height and equally rounded, petiolar dorsum weakly to moderately convex; node in dorsal view around 1.2 to 1.3 times wider than long (DPeI 120–133), in dorsal view pronotum between 2.3 to 2.6 times wider than petiolar node (PeNI 38–43). Postpetiole in profile subglobular, around 1.4 to 1.5 times higher than long (LPpI 67–72); in dorsal view around 1.4 to 1.6 times wider than long (DPpI 142–157), pronotum between 1.7 to 2.0 times wider than postpetiole (PpNI 51–58). Postpetiole in profile usually appearing less voluminous than petiolar node, postpetiole in dorsal view around 1.3 to 1.4 times wider than petiolar node (PPI 131–144). Mandibles completely unsculptured, smooth, and shiny; clypeus longitudinally rugulose with three to seven rugulae, median rugula always present and usually fully developed, one or two mostly entire, rarely broken, lateral rugulae present on each side; cephalic dorsum between frontal carinae irregularly longitudinally rugulose with six to ten rugulae, rugulae usually running from posterior clypeal margin to posterior head margin, but mostly irregularly shaped, meandering, interrupted or with cross-meshes; scrobal area mostly unsculptured; lateral head reticulate-rugulose to longitudinally rugulose, posteriorly often partly unsculptured; ground sculpture on head weakly to moderately punctate. Dorsum and sides of mesosoma irregularly longitudinally rugulose to reticulate-rugulose, sometimes in parts only very weakly sculptured and relatively smooth; ground sculpture on mesosoma weak to absent. Forecoxae, both waist segments, and gaster fully unsculptured, smooth, and shining. Dorsum of head with numerous pairs of long, fine, standing hairs; dorsal promesonotum usually with two pairs, rarely three or four; propodeum and waist segments without long, standing pilosity; first gastral tergite with short, moderately dense, appressed pubescence in combination with several scattered, long, standing hairs. Anterior edges of antennal scapes and dorsal (outer) surfaces of hind tibiae with appressed to decumbent hairs. Head, mesosoma, waist segments and gaster uniformly orange brown contrasting with yellowish to light brown mandibles, antennae, and legs.

####### Distribution and biology.

*Tetramorium sikorae* is widely distributed in Madagascar from Andrambovato in Fianarantsoa in the southeast to Binara in the north, and is also found in the isolated montane forest Ambohijanahary in western Madagascar (Fig. 66). Its main distribution is centered in central-eastern Madagascar in the area from Torotorofotsy and Andasibe-Mantadia north to Betampona and Zahamena. *Tetramorium sikorae* clearly prefers montane rainforests, rarely lowland rainforests, and the elevational range of 240 m to 1100 m must be taken with caution since it is predominantly found at the higher range limit. Also, *Tetramorium sikorae* seems to be a leaf litter inhabitant.

####### Discussion.

*Tetramorium sikorae* is easily identifiable within the *Tetramorium schaufussii* species complex. The character that best separates it from most species is the lack of long, standing pilosity on the waist segments since such pilosity is present in *Tetramorium merina*, *Tetramorium monticola*, *Tetramorium nassonowii*, *Tetramorium pseudogladius*, *Tetramorium schaufussii*, *Tetramorium scutum*, and *Tetramorium xanthogaster*. Only *Tetramorium rala* and *Tetramorium obiwan* share the lack of pilosity on the waist segments, and sometimes pilosity is present in the latter species. However, *Tetramorium sikorae* is unlikely to be confused with *Tetramorium obiwan* for several reasons. First, the latter species is much larger in body size (HW 0.71–0.84; WL 1.00–1.20) than *Tetramorium sikorae* (HW 0.53–0.61; WL 0.70–0.82). Second, *Tetramorium obiwan* has longer antennal scapes (SI 77 -82) than *Tetramorium sikorae* (SI 70–74). Third, both species appear to live in different microhabitats with *Tetramorium obiwan* in the lower vegetation and *Tetramorium sikorae* in leaf litter. Beyond the differing pilosity on the waist segments, *Tetramorium sikorae* is morphologically very close to *Tetramorium schaufussii*. Since the presence or absence of this pilosity seems fairly stable at species levelwe retain these as separate species. *Tetramorium sikorae* is also relatively similar to *Tetramorium cognatum* in the *Tetramorium cognatum* complex, but apart from the gastral pilosity diagnostic for the species complexes, both species also differ in antennal scape length.

Currently, there seems to be no obvious intraspecific variation in *Tetramorium sikorae*.

###### Tetramorium xanthogaster

Santschi, 1911

http://species-id.net/wiki/Tetramorium_xanthogaster

Figs 40C , 41C , 43D , 45D , 46A , 46B , 47E , 48D , 49A , 59 , 66

Tetramorium (Xyphomyrmex) sikorae var. *xanthogaster* Santschi, 1911: 124. [Original spelling of *Tetramorium xantogaster* justifiably emended to *Tetramorium xanthogaster* by Wheeler 1922: 1032] [Raised to species by Bolton 1979: 139]

####### Type material.

**Holotype**, pinned worker, MADAGASCAR (*M.J. de Gaulle*) (NHMB: CASENT0101146) [examined].

**Figure 59.**
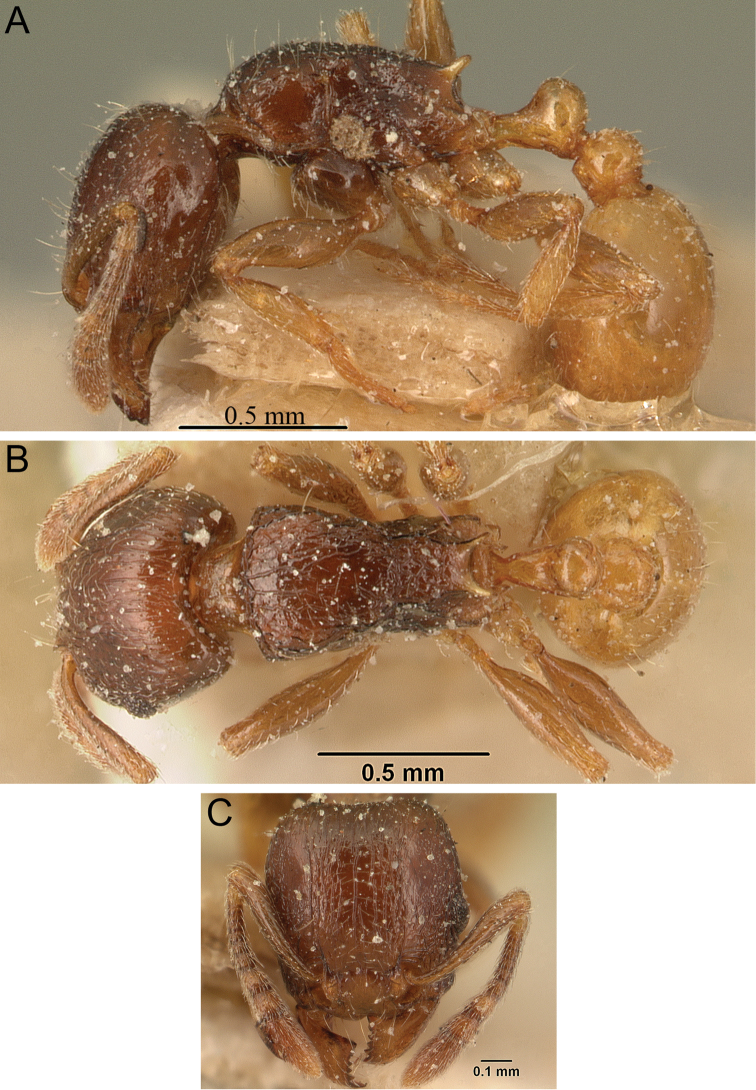
*Tetramorium xanthogaster* holotype worker (CASENT0101146). **A** Body in profile **B** Body in dorsal view **C** Head in full-face view.

####### Non-type material.

MADAGASCAR: Antananarivo, Réserve Spéciale d’Ambohitantely, Forêt d Ambohitantely, 20.9 km 72° NE Ankazobe, 18.22528°S, 47.28683°E, 1410 m, montane rainforest, 17.–22.IV.2001 (*B.L. Fisher et al.*); Antananarivo, Ankokoy Forest, 3 km E Ibity, 20.0675°S, 46.9995°E, 1700 m, *Uapaca* forest, 4.–14.XI.2008 (*M.E. Irwin & Rin’ha*); Antsiranana, Parc National Montagne d’Ambre, 3.6 km 235° SW Joffreville, 12.53444°S, 49.1795°E, 925 m, montane rainforest, 20.–26.I.2001 (*B.L. Fisher et al.*); Antsiranana, Parc National Montagne d’Ambre [1st campsite], 12.51444°S, 49.18139°E, 960 m, rainforest, 21.–26.I.2001 (*M.E. Irwin et al.*); Antsiranana, Parc National Montagne d’Ambre, 12.2 km 211° SSW Joffreville, 12.59639°S, 49.1595°E, 1300 m, montane rainforest, 2.–7.II.2001 (*B.L. Fisher et al.*); Antsiranana, Parc National Montagne d’Ambre, 12.52306°S, 49.17901°E, 1100 m, montane rainforest, 11.III.2011 (*B.L. Fisher et al.*); Antsiranana, Forêt de Binara, 9.4 km 235° SW Daraina, 13.26333°S, 49.6°E, 1100 m, montane rainforest, 5.XII.2003 (*B.L. Fisher*); Antsiranana, Galoko chain, Mont Galoko, 13.59358°S, 48.73157°E, 1100 m, montane forest, 22.II.2013 (*B.L. Fisher et al.*); Antsiranana, 7 km N Joffreville [camp 2 of Fisher], 12.33333°S, 49.25°E, 360 m, dry forest, 13.–16.V.2001 (*R. Harin’Hala*); Antsiranana, R.S. Manongarivo, 14.5 km 220° SW Antanambao, 13.99833°S, 48.42833°E, 1175 m, montane rainforest, 21.X.1998 (*B.L. Fisher*); Antsiranana, R.S. Manongarivo 17.3 km 218° SW Antanambao, 14.02167°S, 48.41833°E, 1580 m, montane rainforest, 27.X.1998 (*B.L. Fisher*); Antsiranana, Parc National de Marojejy, Manantenina River, 28.0 km 38° NE Andapa, 8.2 km 333° NNW Manantenina, 14.43667°S, 49.775°E, 450 m, rainforest, 12.–15.XI.2003 (*B.L. Fisher et al.*); Mahajanga, Réserve Spéciale Marotandrano, Marotandrano 48.3 km S Mandritsara, 16.28322°S, 48.81443°E, 865 m, transition humid forest, 7.XII.2007 (*B.L. Fisher et al.*); Toamasina, Andranobe, 5.3 km SSE Ambanizana, 15.67133°S, 49.97395°E, 425 m, rainforest, 21.XI.1993 (*B.L. Fisher*); Toliara, Forêt Classée d’Analavelona, 29.2 km 343° NNW Mahaboboka, 22.675°S, 44.19°E, 1100 m, montane rainforest, 18.–22.II.2003 (*B.L. Fisher et al.*); Toliara, FForêt Classée d’Analavelona, 33.2 km 344° NNW Mahaboboka, 22.64333°S, 44.17167°E, 1300 m, montane rainforest, 22.–25.II.2003 (*B.L. Fisher et al.*).

####### Diagnosis.

The following character set discriminates *Tetramorium xanthogaster* from the other species of the *Tetramorium schaufussii* complex: moderate to large eyes (OI 22–25); short antennal scapes (SI 70–75); frontal carinae weakly developed, only faintly raised, usually becoming much weaker around eye level and fading out halfway between posterior eye margin and posterior head margin; propodeal spines/teeth very short to short (PSLI 10–16), propodeal spines and lobes not strongly inclined towards each other; petiolar node in profile around 1.5 to 1.7 times higher than long (LPeI 59–67) and in dorsal view around 1.2 to 1.3 times wider than long (DPeI 119–133); dorsum of promesonotum with numerous pairs of long, standing hairs (10+), propodeum usually with one or two pairs, sometimes with up to five; waist segments with long, standing pilosity.

####### Worker measurements

**(N=25).** HL 0.54–0.75 (0.64); HW 0.59–0.80 (0.70); SL 0.38–0.53 (0.46); EL 0.13–0.18 (0.16); PH 0.27–0.41 (0.32); PW 0.39–0.56 (0.46); WL 0.72–1.05 (0.86); PSL 0.06–0.13 (0.09); PTL 0.12–0.19 (0.15); PTH 0.21–0.29 (0.25); PTW 0.16–0.24 (0.19); PPL 0.14–0.21 (0.18); PPH 0.19–0.29 (0.24); PPW 0.19–0.29 (0.24); CI 92–94 (93); SI 70–75 (72); OI 22–25 (24); DMI 51–55 (54); LMI 36–39 (38); PSLI 10–16 (13); PeNI 37–46 (41); LPeI 59–67 (62); DPeI 119–133 (126); PpNI 48–56 (51); LPpI 68–80 (75); DPpI 125–140 (135); PPI 117–129 (125).

####### Worker description.

Head clearly longer than wide (CI 92–94); posterior head margin weakly concave. Anterior clypeal margin with distinct median impression. Frontal carinae weakly developed, only faintly raised, usually becoming much weaker around eye level and fading out halfway between posterior eye margin and posterior head margin. Antennal scrobes very weak, shallow and without clear and distinct posterior and ventral margins. Antennal scapes short, not reaching posterior head margin (SI 70–75). Eyes moderate to relatively large (OI 22–25). Mesosomal outline in profile flat to weakly convex, comparatively low and long (LMI 36–39), weakly to moderately marginate from lateral to dorsal mesosoma; promesonotal suture absent; metanotal groove weakly developed or absent. Propodeal spines/teeth very short to short, varying from triangular and blunt to elongate-triangular and acute (PSLI 10–16), propodeal lobes short, triangular, and blunt, always much shorter than propodeal spines, spines and lobes not strongly inclined towards each other. Petiolar node in profile high nodiform, nodiform or weakly cuneiform, always with relatively well rounded antero- and posterodorsal margins, around 1.5 to 1.7 times higher than long (LPeI 59–67), anterior and posterior faces often approximately parallel and often not, anterodorsal and posterodorsal margins usually at about same height, sometimes anterodorsal margin situated slightly higher than posterodorsal, petiolar dorsum weakly to moderately convex; petiolar node in dorsal view around 1.2 to 1.3 times wider than long (DPeI 119–133), in dorsal view pronotum between 2.2 to 2.7 times wider than petiolar node (PeNI 37–46). Postpetiole in profile globular to subglobular, approximately 1.3 to 1.5 times higher than long (LPpI 68–80); in dorsal view around 1.3 to 1.4 times wider than long (DPpI 125–140), pronotum between 1.8 to 2.1 times wider than postpetiole (PpNI 48–56). Postpetiole in profile appearing more or less as voluminous as petiolar node, postpetiole in dorsal view around 1.2 to 1.3 times wider than petiolar node (PPI 117–129). Mandibles unsculptured, smooth, and shining; generally sculpture on clypeus very much reduced, median area usually unsculptured with one or two weak, irregular, and broken rugulae laterally, very rarely median rugula present but then weak and broken; cephalic dorsum between frontal carinae irregularly longitudinally rugulose with six to eight widely separated rugulae, rugulae running from posterior clypeal margin to posterior head margin, but irregularly shaped, often broken or with cross-meshes, and becoming weaker or fading out towards posterior head margin; scrobal area partly unsculptured, but mostly merging laterally with surrounding reticulate-rugose to longitudinally rugose sculpture present around eyes, most of lateral head predominantly unsculptured, smooth, and shiny; ground sculpture on head weakly to moderately punctate. Dorsum of mesosoma weakly to moderately longitudinally rugulose, sometimes irregularly so, often rugulae very weak and parts of dorsal mesosoma smooth; lateral mesosoma anteriorly often only weakly sculptured and shiny, katepisternum and lateral propodeum usually irregularly longitudinally rugulose to reticulate-rugose, sometimes almost completely unsculptured, smooth, and shiny; ground sculpture on mesosoma usually only weakly developed, mostly absent. Forecoxae unsculptured, smooth, and shining. Waist segments and gaster unsculptured, smooth, and shining. Dorsum of head with several pairs of long, fine, standing hairs; dorsum of promesonotum always with more than ten pairs of long, standing hairs, propodeum usually with one or two pairs, sometimes with up to five, rarely without any standing pilosity; waist segments each with several pairs; first gastral tergite with short, scarce to moderately abundant, appressed to decumbent pubescence in combination with scattered, long, standing hairs. Anterior edges of antennal scapes and dorsal (outer) surfaces of hind tibiae with decumbent to subdecumbent hairs. Body colour variable, usually bicoloured with head and mesosoma of dark brown to blackish colour contrasting with yellowish or light brown appendages, waist segments, and gaster; very rarely only head of dark brown contrasting with yellowish remainder of body, sometimes body uniformly coloured, ranging from yellow to dark brown.

####### Distribution and biology.

*Tetramorium xanthogaster* has a relatively patchy distribution range (Fig. 66). The southernmost locality is Analavelona in the southwest. The next known localities are located in the Central Highlands much further north and east (Ambohitantely, Ankokoy, and Marotandrano). The remaining localities are situated in the northern part of Madagascar (Andranobe, Marojejy, Binara, Montagne d’Ambre, and Manongarivo). Many of these localities are widely separated from each other. Furthermore, it seems that *Tetramorium xanthogaster* prefers montane rainforests since most collections are from elevations around or above 1000 m, whereas it was only occasionally collected from lower elevations. An explanation for the currently patchy distribution records could be that *Tetramorium xanthogaster* nests and/or forages in the vegetation since almost all of the available material was collected either from beating low vegetation or Malaise traps. Consequently, we expect that more collecting in the lower vegetation stratum will likely yield more material of this species.

####### Discussion.

Despite its pronounced variability in colouration and petiolar node shape, the determination of *Tetramorium xanthogaster* is fairly straightforward. The species was described by Santschi (1911) and later redescribed by Bolton (1979) as a bicoloured species with dark head and mesosoma contrasting with the yellowish to light brown remainder of the body. Indeed, the material from the southwest and the Central Highlands is consistently strongly bicoloured, and even though one should not overly rely too heavily on body colouration, no other species of the *Tetramorium schaufussii* species group is similarly bicoloured. However, material from the northern localities shows remarkable diversity in colouration. The populations from Andranobe, Marojejy, and Binara are of a fairly bright, yellowish brown. By contrast, the material from Manongarivo and Montagne d’Ambre is in parts bicoloured like the southern populations, partly bicoloured with only the head or mesosoma darker than the rest of the body, or just uniformly light brown to dark brown. But even misregarding colouration, *Tetramorium xanthogaster* cannot be misidentified with another member of the complex. Due to the presence of long, standing pilosity on its waist segments it cannot be mistaken for *Tetramorium obiwan* (partly), *Tetramorium pseudogladius*, *Tetramorium sikorae*, or *Tetramorium rala*, and the broader than long petiolar node (DPeI 119–133) distinguishes it from *Tetramorium nassonowii* (DPeI 87–98). In addition, the petiolar node of *Tetramorium xanthogaster* in profile is around 1.5 to 1.7 times higher than long (LPeI 59–67), which separates it from *Tetramorium rala* and *Tetramorium scutum* that have nodes which are 2.0 to 2.2 times higher than long (LPeI 45–50). The petiolar nodes of the latter two are also thinly cuneiform to weakly squamiform while the node of *Tetramorium xanthogaster* is variably shaped, but lower and less angled. *Tetramorium obiwan* possesses well-developed frontal carinae and cephalic sculpture, as well as relatively long antennal scapes (SI 77–82), whereas *Tetramorium xanthogaster* has much weaker frontal carinae and cephalic sculpture and shorter antennal scapes (SI 70–75). The remaining three species, *Tetramorium merina*, *Tetramorium monticola*, and *Tetramorium schaufussii*, are more difficult to separate from *Tetramorium xanthogaster*. *Tetramorium merina* is only found in the central Highlands and co-occurs there with *Tetramorium xanthogaster*. In this region *Tetramorium xanthogaster* is always strongly bicoloured with head and mesosoma very dark brown to black, which contrasts with the yellow to light brown waist segments and gaster. In addition, *Tetramorium merina* lacks any standing pilosity on the propodeum while *Tetramorium xanthogaster* usually has long, fine, standing pilosity on the propodeal dorsum. As mentioned above, there are not too many other diagnostic characters to separate these species, but the fact that they co-occur in sympatry in several localities without any intermediate forms supports their heterospecificity. *Tetramorium monticola* is also sympatric with *Tetramorium xanthogaster*, this time in the northern part of Madagascar, where the colouration of the latter species is not reliable. However, in *Tetramorium monticola* the frontal carinae are more strongly developed and the cephalic dorsum between the frontal carinae has nine to thirteen relatively regularly shaped, mostly unbroken rugae while the frontal carinae of *Tetramorium xanthogaster* are weakly developed and the cephalic dorsum has only six to eight widely separated, often irregularly shaped rugulae. Also, the propodeal spines of the latter are shorter (PSLI 10–16) than in *Tetramorium monticola* (PSLI 18–22). The last and most common and abundant species of the complex, *Tetramorium schaufussii*, is also usually found in sympatry with *Tetramorium xanthogaster*, but both species can be well separated by head shape. The head of *Tetramorium schaufussii* is much longer and thinner (CI 86–90) than in *Tetramorium xanthogaster* (CI 92–94). Additional differentiating characters are the long, standing pilosity on the propodeal dorsum (usually present in *Tetramorium xanthogaster* but absent in *Tetramorium schaufussii*) and the median clypeal ruga (present in *Tetramorium schaufussii* but absent in *Tetramorium xanthogaster*).

It has to be noted that *Tetramorium xanthogaster* is relatively variable in the shape of the petiolar node, which can be high nodiform, nodiform, cuneiform, or in intermediate stages. The node shape varies strongly even within material from the same collection event. Another noteworthy variation can be seen in the material from Analavelona. In contrast to the rest of the material from all other localities it seems that the sculpture on the cephalic dorsum and the mesosomal dorsum is much better developed, and does not differ significantly from other species like *Tetramorium schaufussii*. However, the frontal carinae are still weaker than in the latter.

### Revision of the *Tetramorium severini* species group

#### *Tetramorium severini* species group

**Diagnosis.** Eleven-segmented antennae; anterior clypeal margin with distinct median impression; frontal carinae strongly developed and long, usually ending shortly before posterior head margin; antennal scrobes present, but weak and without well-developed posterior and ventral margins; anterior face of mesosoma weakly developed; mesosomal outline in profile flat and relatively elongated, only very weakly marginate from lateral to dorsal mesosoma, sides usually rounding onto dorsum; mesosoma relatively low (LMI 35–37); propodeal spines long to very long, spinose and acute (PSLI 38–43); propodeal lobes triangular and short; petiolar node in profile high rounded nodiform, in profile around 1.5 to 1.7 times higher than long (LPeI 57–69), node in dorsal view around 1.1 to 1.2 times wider than long (DPeI 104–121), anterior and posterior faces approximately parallel; postpetiole in profile globular to subglobular; mandibles unsculptured, smooth, and shining; cephalic dorsum with distinct longitudinally rugose sculpture; mesosoma laterally, distinctly, irregularly, and longitudinally rugose to reticulate-rugose, sculpture on mesosomal dorsum relatively weak, usually consisting of feeble, irregular longitudinal rugulae; waist segments and gaster unsculptured, smooth, and shiny; head with numerous standing, long hairs, mesosoma with one or two long hairs, waist segments and first gastral tergite without any standing pilosity; first gastral tergite with very short and appressed pubescence; sting appendage spatulate.

**Comments.** This group only holds the species *Tetramorium severini*, which is a common faunal element in most rainforests in eastern Madagascar. Previous to Hita Garcia and Fisher (2011), *Tetramorium severini* was placed in the *Tetramorium schaufussii* group by Bolton (1979). Despite strong similarities in the shape of the mesosoma and waist segments, we still consider *Tetramorium severini* distinct enough from all members of the *Tetramorium schaufussii* group to justify its placement in its own species group. All species of the *Tetramorium schaufussii* group are comparatively small species with very short to moderately long propodeal spines, whereas *Tetramorium severini* is one of the largest species found in Madagascar outside the *Tetramorium tortuosum*, *Tetramorium kelleri*, and *Tetramorium tosii* species groups, and possesses very long propodeal spines. Admittedly few more factors argue for the separation, and future reexaminations may come to a different conclusion. However, at present, we believe the similarities between *Tetramorium severini* and the *Tetramorium schaufussii* group are convergent in nature, an idea also supported by unpublished mtDNA data (FHG & BLF, unpublished data).

Furthermore, the *Tetramorium severini* group can be easily distinguished from all other Malagasy *Tetramorium* species and groups by its 11-segmented antennae, long and slender mesosoma (LMI 35–37) without distinct margination between lateral and dorsal mesosoma, very long propodeal spines (PSLI 38–43), rounded high nodiform petiolar node, and completely unsculptured waist segments.

##### Tetramorium severini

(Emery, 1895)

http://species-id.net/wiki/Tetramorium_severini

Figs 60 , 66

Xiphomyrmex severini Emery, 1895: 343. [Combination in *Tetramorium* by Bolton 1979: 138]

###### Type material.

**Holotype**, pinned worker, MADAGASCAR, Diego Suarez, 1893 (*C. Allaud*) (MCSN: CASENT0102078) [examined].

[Note: Emery (1895) did not note the number of specimens in the primary description of the species. He only stated the type locality to be Diego Suarez. Bolton (1979) noted in his redescription that the species was described based on a syntype series located in MCSN and MHNG. However, examination of the material from both collections indicates that only the single type from MCSN is a primary name-bearing type from the type locality mentioned in the primary description (Emery 1895). The material from MHNG consists of one cotype only, which was collected from the Bay of Antongil, and is thus neither part of the type series nor a valid type at all. To increase the confusion, the type from MCSN was labelled as type and also as syntype. Nevertheless, since there does not seem to be another primary type in either MCSN or MHNG, we consider the single type from MCSN as holotype.]

**Figure 60.**
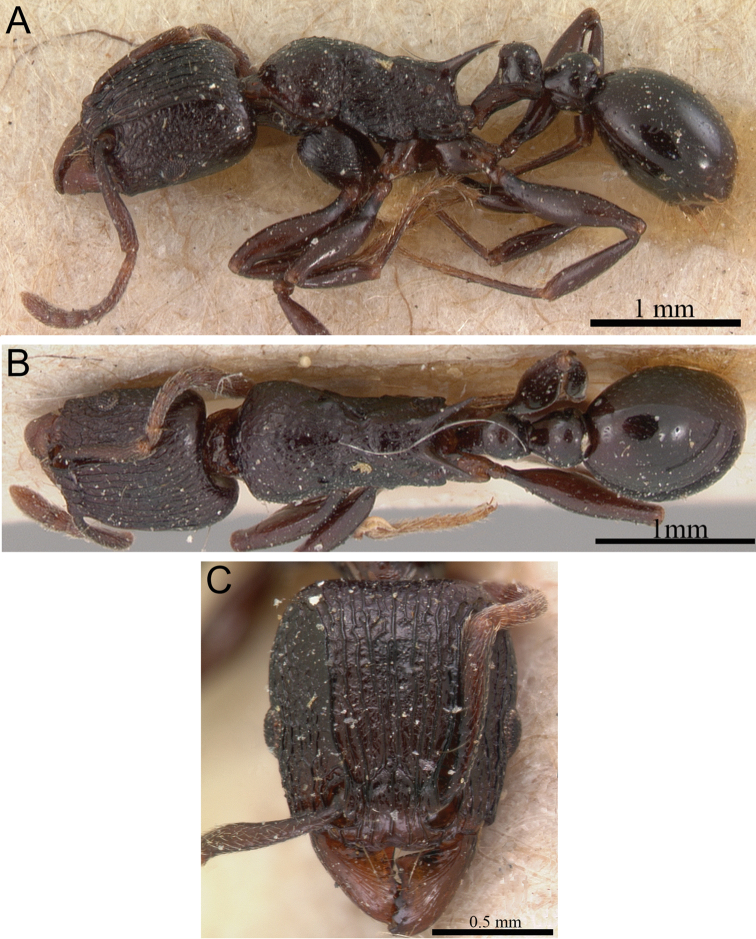
*Tetramorium severini* holotype worker (CASENT0102078). **A** Body in profile **B** Body in dorsal view **C** Head in full-face view.

###### Non-type material.

MADAGASCAR: Antsiranana, Ambato, 14 km W Cap Est, 15°17'28.6"S, 50°20'16.9"E, 150 m, secondary rainforest, (*G.D. Alpert et al.*); Antsiranana, Parc National Montagne d’Ambre, 3.6 km 235° SW Joffreville, 12.5344°S, 49.1795°E, 925 m, montane rainforest, 20.–26.I.2001 (*B.L. Fisher et al.*); Antsiranana, Réserve Spéciale d’Ambre, 3.5 km 235° SW Sakaramy, 12.4689°S, 49.2422°E, 325 m, tropical dry forest, 26.–31.I.2001 (*B.L. Fisher et al.*); Antsiranana, Parc National Montagne d’Ambre (Petit Lac road), 12.5203°S, 49.1792°E, 1125, rainforest, 12.–14.V.2001 (R. Harin’Hala); Antsiranana, Parc National Montagne d’Ambre, Crête, 12.5813°S, 49.1337°E, 1110 m, 13.XI.2007 (*B.L. Fisher et al.*); Antsiranana, Parc National Montagne d’Ambre, Roussettes, 12.5257°S, 49.1724°E, 1025 m, montane rainforest, 15.XI.2007 (*B.L. Fisher et al.*); Antsiranana, Parc National Montagne d’Ambre, Antomboka, 12.5003°S, 49.175°E, 885 m, montane rainforest, 16.XI.2007 (*B.L. Fisher et al.*); Antsiranana, Parc National Montagne d’Ambre, Antomboka, 12.5127°S, 49.1781°E, 970 m, montane rainforest, 16.–17.XI.2007 (*B.L. Fisher et al.*); Antsiranana, Parc National Montagne d’Ambre, Pic Bades, 12.5186°S, 49.1862°E, 900 m, 20.XI.2007 (*B.L. Fisher et al.*); Antsiranana, Parc National Montagne d’Ambre, 12.5139°S, 49.1778°E, 984 m, montane rainforest, 23.–27.II.2011 (*B.L. Fisher et al.*); Antsiranana, 1 km W Andampibe, Cap Masoala, 15°41'37"S, 50°10'53.4"E, 125 m, rainforest, 30.XI.1993 (*G.D. Alpert*); Antsiranana, 2.0 km S Andrakata, 14.65°S, 49.7167°E, 520 m, disturbed rainforest, 2.XII.1994 (*B.L. Fisher*); Antsiranana, Rés. Anjanaharibe-Sud, 14°45'27.9"S, 49°30'36.7"E, 875 m, rainforest, 4.XI.1994 (*G.D. Alpert*); Antsiranana, Rés. Anjanaharibe-Sud, 6.5 km SSW Befingotra, 14.75°S, 49.5°E, 875 m, rainforest, 20.X.1994 (*B.L. Fisher*); Antsiranana, Betaolana Forest, along Bekona River, 14.53°S, 49.4404°E, 880 m, rainforest, 4.–5.III.2009 (*B.L. Fisher*); Antsiranana, Betaolana forest, Ambodihazovolabe village along Ambolokopatrika river, 14.5448°S, 49.4516°E, 740 m, disturbed forest patch, 6.III.2009 (*B.L. Fisher*); Antsiranana, Forêt de Binara, 9.1 km 233° SW Daraina, 13.2633°S, 49.6033°E, 800 m, rainforest, 3.XII.2003 (*B.L. Fisher*); Antsiranana, Forêt de Binara, 9.1 km 233° SW Daraina, 13.2633°S, 49.6033°E, 725 m, rainforest, 4.XII.2003 (*B.L. Fisher*); Antsiranana, Forêt de Binara, 9.4 km 235° SW Daraina, 13.2633°S, 49.6°E, 1100 m, montane rainforest, 5.–6.XII.2003 (*B.L. Fisher*); Antsiranana, Forêt de Binara, 9.1 km 233° SW Daraina, 13.2633°S, 49.6033°E, 725 m, rainforest, 19.IV.2004 (*B.L. Fisher*); Antsiranana, Forêt de Binara, 9.1 km 233° SW Daraina, 13.2633°S, 49.6033°E, 800 m, rainforest, 20-21.IV.2004 (*B.L. Fisher*); Antsiranana, Makirovana forest, 14.1707°S, 49.9541°E, 415 m, rainforest, 28.–29.IV.2011 (*B.L. Fisher et al.*); Antsiranana, R.S. Manongarivo, 10.8 km 229° SW Antanambao, 13.9617°S, 48.4333°E, 400 m, rainforest, 8.–13.XI.1998 (*B.L. Fisher*); Antsiranana, R.S. Manongarivo, 12.8 km 228° SW Antanambao, 13.9767°S, 48.4233°E, 760–780 m, rainforest, 11.–17.X.1998 (*B.L. Fisher*); Antsiranana, Marojejy R.N.I. #12, 14°26'43.2"S, 49°47'8.3"E, 375 m, rainforest, 23.XI.1993 (*G.D. Alpert*); Antsiranana, Parc National de Marojejy, Manantenina River, 28.0 km 38° NE Andapa, 8.2 km 333° NNW Manantenina, 14.4367°S, 49.775°E, 450 m, rainforest, 12.XI.–12.XII.2003 (*B.L. Fisher et al.*); Antsiranana, Marojejy National Park, 5 km W Manantenina village, 1st Camp site (Mantella), 14.4382°S, 49.774°E, 487 m, rainforest, 20.XII.2004–7.V.2005 (*M. Rin’Ha*); Antsiranana, Parc National de Marojejy, Manantenina River, 27.6 km 35° NE Andapa, 9.6 km 327° NNW Manantenina, 14.435°S, 49.76°E, 775 m, rainforest, 11.XII.2005 (*B.L. Fisher et al.*); Antsiranana, Nosy Be, 4 km ESE Andoany (=Hellville), 13.4167°S, 48.3°E, 100 m, rainforest, 1.V.1989 (*P.S. Ward*); Antsiranana, Ampasindava, Forêt d’Ambilanivy, 3.9 km 181° S Ambaliha, 13.7986°S, 48.1617°E, 600 m, rainforest, 4.–9.III.2001 (*B.L. Fisher et al.*); Antsiranana, Nosy Be, Réserve Naturelle Intégrale de Lokobe, 6.3 km 112° ESE Hellville, 13.4193°S, 48.3312°E, 30 m, rainforest, 19.–24.III.2001 (*B.L. Fisher et al.*); Antsiranana, 84 km SW Sambava on road to Andapa, degraded forest, 115 m, 17.II.1977 (*W.L. & D.E. Brown*); Antsiranana, 84 km SW Sambava, secondary forest, 26.I.1991 (*G.D. Alpert*); Fianarantsoa, Réserve Speciale Manombo 24.5 km 228° Farafangana, 23.0158°S, 47.719°E, 30 m, rainforest, 21.IV.2006 (*B.L. Fisher et al.*); Fianarantsoa, Ranomafana Nat. Park, Miaranony Forest, 21.25°S, 47.41667°E, 1050 m, montane rainforest, 24.X.1992 (*E. Rajeriarison*); Fianarantsoa, Forêt Classée Vatovavy, 7.6 km 122° Kianjavato, 21.4°S, 47.94°E, 175 m, rainforest, 6.–8.VI.2005 (*B.L. Fisher et al.*); Fianarantsoa, Forêt de Vevembe, 66.6 km 293° Farafangana, 22.791°S, 47.1818°E, 600 m, rainforest, transition to montane forest, 23.–24.IV.2006 (*B.L. Fisher et al.*); Toamasina, Montagne d’Akirindro 7.6 km 341° NNW Ambinanitelo, 15.2883°S, 49.5483°E, 600 m, rainforest, 17.–21.III.2003 (*B.L. Fisher et al.*); Toamasina, Andranobe, 6.3 km S Ambanizana, 15.6813°S, 49.958°E, 25 m, rainforest, 14.1993 (*B.L. Fisher*); Toamasina, Andranobe, 6.3 km S Ambanizana, 15.6813°S, 49.958°E, 150 m, 15.1993 (*B.L. Fisher*); Toamasina, F.C. Andriantantely, 530 m, 18.695°S, 48.8133°E, rainforest, 4.–7.XII.1998 (*H.J. Ratsirarson*); Toamasina, Montagne d’Anjanaharibe, 18.0 km 21° NNE Ambinanitelo, 15.1883°S, 49.615°E, 470 m, rainforest, 8.–12.III.2003 (*B.L. Fisher et al.*); Toamasina, Baie d’Antongil, no additional collection data; Toamasina, P.N. Mantadia, 18.7917°S, 48.4267°E, 895 m, rainforest, 25.XI.–1.XII.1998 (*H.J. Ratsirarson*); Toamasina, Sahafina forest, 11.4 km W Brickaville, 18.8144°S, 48.9621°E, 140 m, rainforest, 14.XII.2007 (*B.L. Fisher et al.*); Toamasina, Parc National de Zahamena, Tetezambatana forest, near junction of Nosivola and Manakambahiny Rivers, 17.743°S, 48.7294°E, 860 m, rainforest, 18.II.2009 (*B.L. Fisher et al.*); Toamasina, Parc National de Zahamena, 17.7336°S, 48.7262°E, 950 m, rainforest, 19.II.2009 (*B.L. Fisher et al.*); Toamasina, Parc National de Zahamena, Sahavorondrano River, 17.7526°S, 48.8573°E, 765 m, rainforest, 23.II.2009 (*B.L. Fisher et al.*); Toliara, Res. Andohahela, 5 km NNW Isaka-Ivondro, 24°45'32"S, 46°51'15.2"E, 235 m, 3.II.1992 (*E. Rajeriarison*); Toliara, Res. Andohahela, 6 km SSW Eminimimy, 24°44'35"S, 46°48'19"E, 330 m, 4.II.1992 (*E. Rajeriarison*); Toliara, Res. Andohahela, 10 km NW Enakara, 24.5667°S, 46.8167°E, 400 m, rainforest, 25.XI.1992 (*B.L. Fisher*); Toliara, Parc National d’Andohahela, Manampanihy River, 5.4 km 113° ESE Mahamavo, 36.7 km 343° NNW Tolagnaro, 24.7639°S, 46.7668°E, 650 m, rainforest, 24.I.2004 (*B.L. Fisher et al.*); Toliara, Parc National Andohahela, Col de Tanatana, 33.3 km NW Tolagnaro, 24.7585°S, 46.8537°E, 275 m, rainforest, 23.XI.2006 (*B.L. Fisher et al.*); Toliara, Forêt Ivohibe 55.0 km N Tolagnaro, 24.569°S, 47.204°E, 200 m, rainforest, 3.XII.2006 (*B.L. Fisher et al.*).

###### Diagnosis.

*Tetramorium severini* can be easily distinguished from all other Malagasy *Tetramorium* by the following character combination: 11-segmented antennae; long and slender mesosoma (LMI 35–37) without distinct margination between lateral and dorsal mesosoma; very long propodeal spines (PSLI 38–43); rounded high nodiform petiolar node; weakly sculptured mesosomal dorsum; completely unsculptured waist segments; very dark brown to black colouration.

###### Worker measurements

**(N=12).** HL 0.87–1.08 (0.95); HW 0.82–1.03 (0.89); SL 0.58–0.80 (0.66); EL 0.19–0.24 (0.21); PH 0.40–0.55 (0.45); PW 0.61–0.75 (0.65); WL 1.14–1.48 (1.24); PSL 0.35–0.47 (0.39); PTL 0.20–0.24 (0.22); PTH 0.34–0.42 (0.36); PTW 0.23–0.29 (0.25); PPL 0.27–0.35 (0.30); PPH 0.33–0.42 (0.36); PPW 0.30–0.39 (0.33); CI 92–96 (94); SI 69–78 (73); OI 22–24 (23); DMI 51–54 (52); LMI 35–37 (36); PSLI 38–43 (41); PeNI 35–41 (38); LPeI 57–69 (62); DPeI 104–121 (111); PpNI 47–54 (50); LPpI 79–91 (83); DPpI 103–117 (110); PPI 125–143 (132).

###### Worker description.

Head distinctly longer than wide (CI 92–96); posterior head margin weakly concave. Anterior clypeal margin with distinct median impression. Frontal carinae strongly developed, diverging posteriorly, and usually ending shortly before posterior head margin; antennal scrobe present, but weak, shallow, and without defined posterior or ventral margins. Antennal scapes short, not reaching posterior head margin (SI 69–78). Eyes of moderate size (OI 22–24). Mesosomal outline in profile flat, relatively low and elongated (LMI 35–37), only weakly marginate from lateral to dorsal mesosoma; promesonotal suture and metanotal groove usually present, but relatively weak. Propodeal spines very long, spinose, and acute (PSLI 38–43); propodeal lobes short, triangular, and blunt, always much shorter than propodeal spines. Petiolar node in profile high, rounded nodiform, with well-rounded antero- and posterodorsal margins, around 1.5 to 1.7 times higher than long (LPeI 57–69), anterior and posterior faces approximately parallel, generally anterodorsal and posterodorsal margins situated at about same height (sometimes anterodorsal margin higher than posterodorsal margin), petiolar dorsum always noticeably rounded and convex; node in dorsal view around 1.1 to 1.2 times wider than long (DPeI 104–121), in dorsal view pronotum between 2.4 to 2.8 times wider than petiolar node (PeNI 35–41). Postpetiole in profile globular to subglobular, approximately 1.1 to 1.3 times higher than long (LPpI 79–91); in dorsal view between 1.0 to 1.2 times wider than long (DPpI 103–117), pronotum around 1.8 to 2.1 times wider than postpetiole (PpNI 47–54). Postpetiole in profile usually appearing thicker and slightly lower than petiolar node, postpetiole in dorsal view between 1.2 to 1.4 times wider than petiolar node (PPI 125–143). Mandibles variably sculptured, ranging from fully unsculptured, smooth, and shining, through partially striate, especially basally, to fully covered by fine striae; clypeus longitudinally rugose/rugulose, with four to eight rugae/rugulae, median ruga always very well developed and distinct, lateral rugulae usually much weaker and usually broken; cephalic dorsum between frontal carinae with seven to nine thick, longitudinal rugae, rugae running mostly unbroken from posterior clypeal margin to posterior head margin, rarely interrupted or with cross-meshes; scrobal area usually mostly unsculptured, rarely longitudinally rugulose to reticulate- rugulose; lateral head longitudinally rugulose to reticulate- rugulose, often with unsculptured and smooth areas. Mesosoma laterally irregularly longitudinally rugose to reticulate-rugose; sculpture on mesosomal dorsum relatively weak, usually consisting of feeble, irregular longitudinal rugulae, often with unsculptured and smoother areas. Ground sculpture on head and mesosoma weakly to moderately punctate Forecoxae rugulose to reticulate-rugulose. Waist segments and gaster completely unsculptured, smooth. and shining. Head with numerous long, standing hairs, mesosoma with one or two long hairs, waist segments and first gastral tergite without any standing hairs at all; first gastral tergite with very short, moderately scattered, and appressed pubescence. Anterior edges of antennal scapes and dorsal (outer) surfaces of hind tibiae with appressed hairs only. Body very dark brown to black, appendages often slightly lighter.

###### Distribution and biology.

*Tetramorium severini* is widely distributed in the rainforests and montane rainforests of eastern and northern Madagascar (Fig. 66). The distribution ranges from Andohahela in the southeast to Montagne d’Ambre on the northern tip of the island, and from there south to Nosy Be, Ampasindava, and Manongarivo on the northwestern side. Throughout its distribution, *Tetramorium severini* is found at altitudes ranging from 25 to 1125 m. In addition, it seems that the species lives in leaf litter and/or the ground.

###### Discussion.

As mentioned above, *Tetramorium severini* is the only member of its species group, and a prominent element of the Malagasy *Tetramorium* fauna. The large body size, very dark colour, very long propodeal spines, reduced sculpture on the mesosoma, and complete lack of sculpture on the waist segments render it immediately recognisable. Considering its wide distribution and large number of specimens examined, *Tetramorium severini* displays very little intraspecific variation.

**Figure 61.**
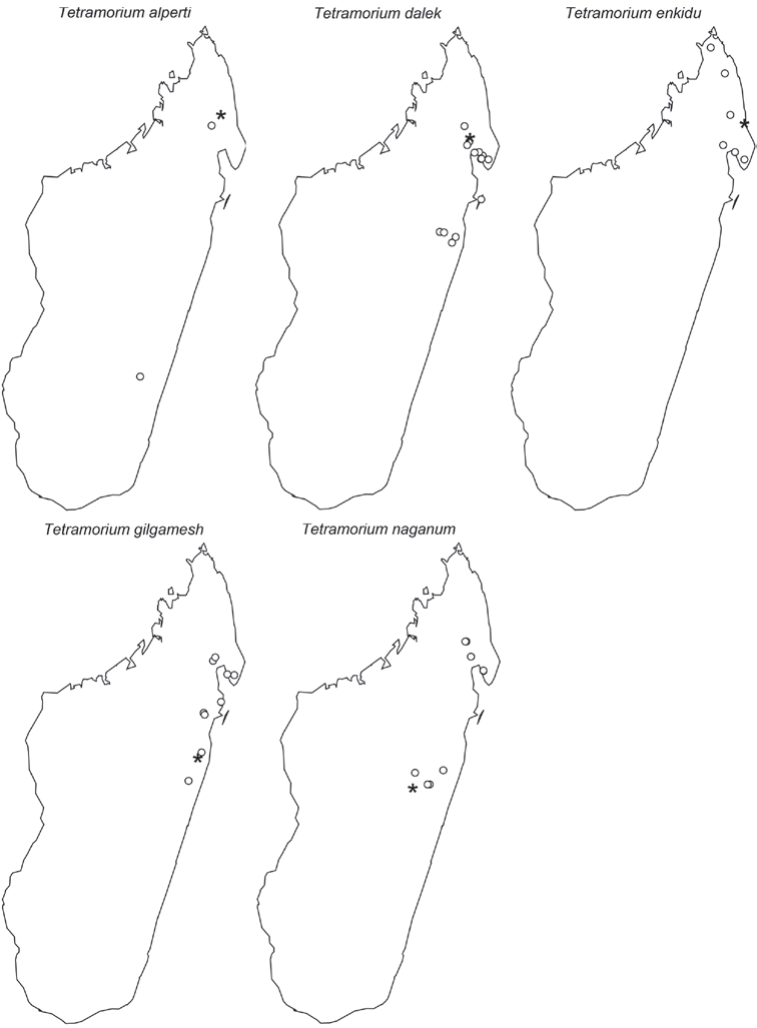
Geographic distribution maps for the species of the *Tetramorium naganum* species group. Star symbols represent type localities while circles represent non-type localities.

**Figure 62.**
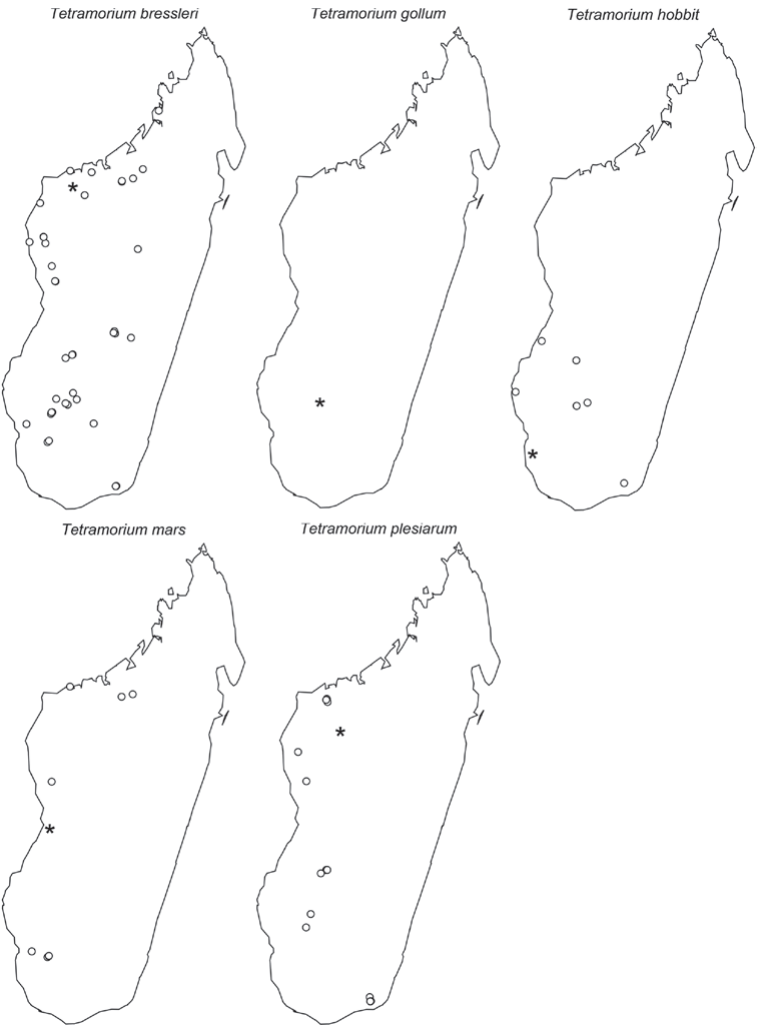
Geographic distribution maps for the species of the *Tetramorium plesiarum* species group. Star symbols represent type localities while circles represent non-type localities.

**Figure 63.**
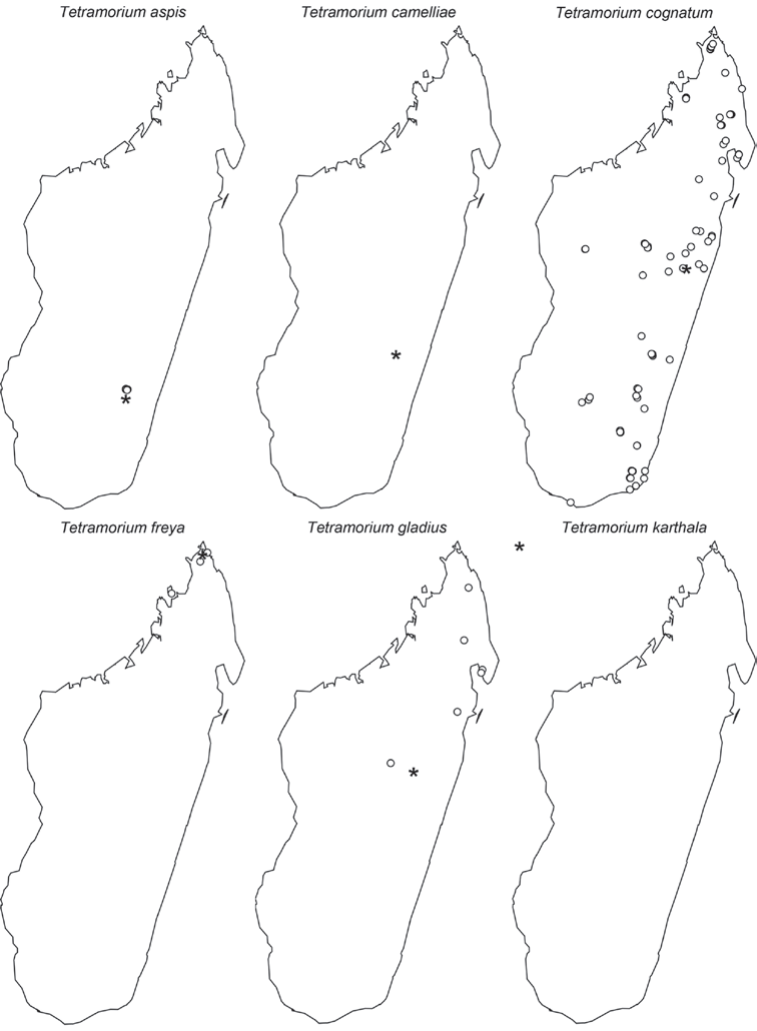
Geographic distribution maps for the species of the *Tetramorium cognatum* species complex I. Star symbols represent type localities while circles represent non-type localities. Note that *Tetramorium karthala* is endemic to the island of Grand Comore and not found on Madagascar.

**Figure 64.**
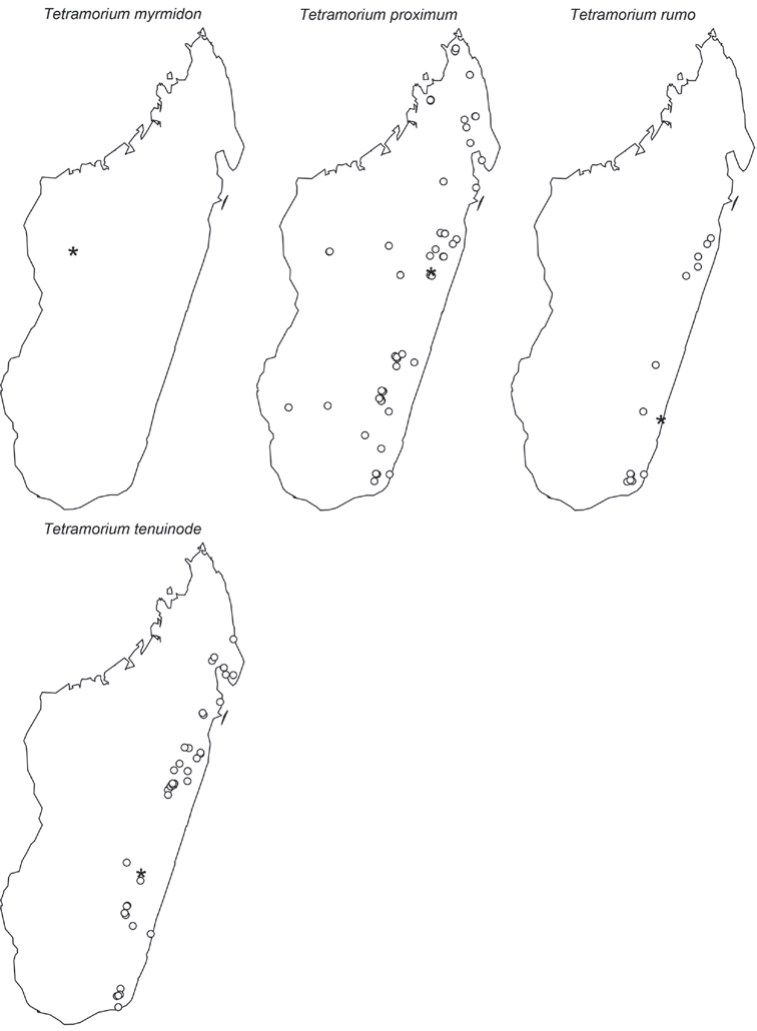
Geographic distribution maps for the species of the *Tetramorium cognatum* species complex II. Star symbols represent type localities while circles represent non-type localities.

**Figure 65.**
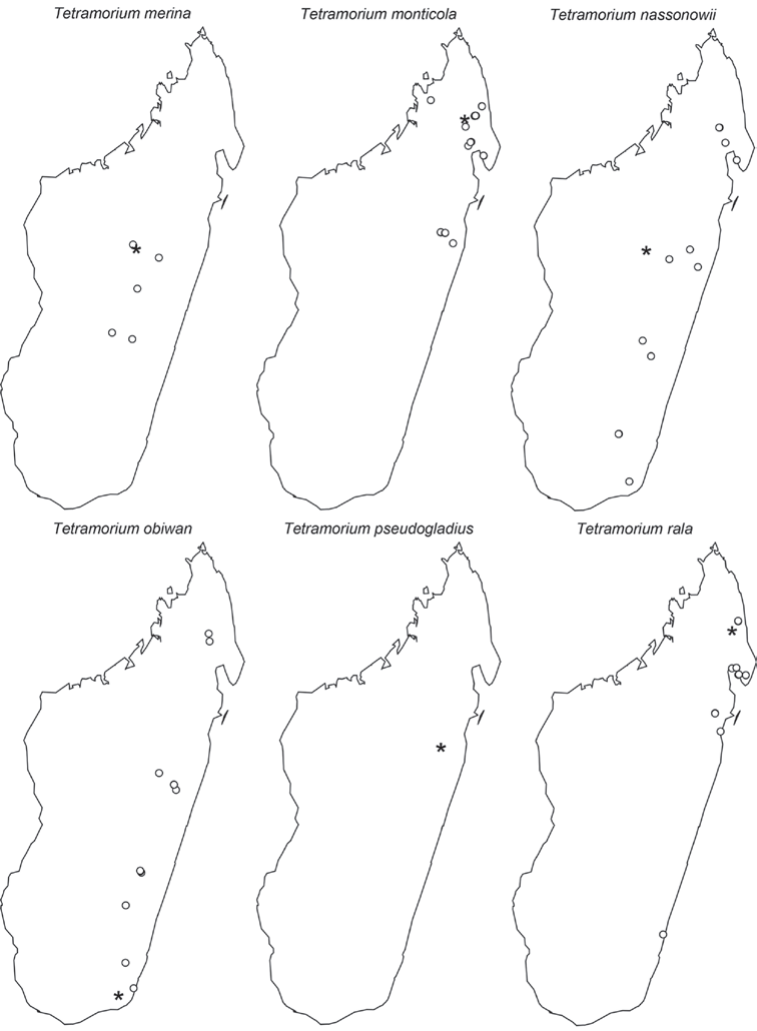
Geographic distribution maps for the species of the *Tetramorium schaufussii* species complex I. Star symbols represent type localities while circles represent non-type localities.

**Figure 66.**
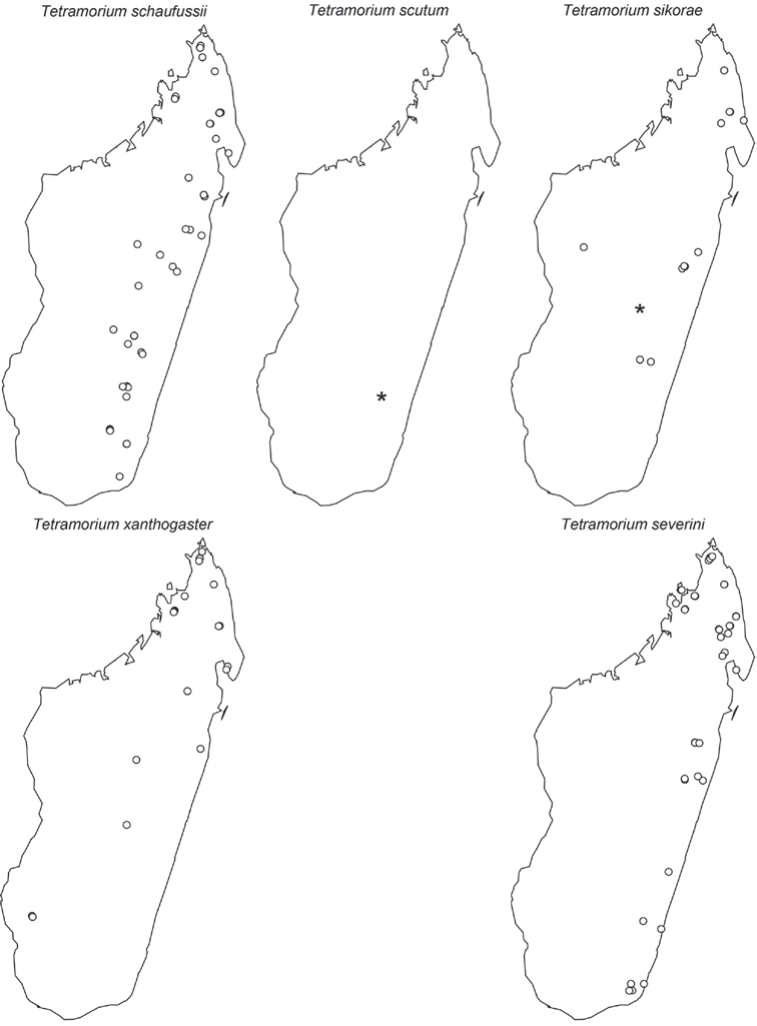
Geographic distribution maps for the species of the *Tetramorium schaufussii* species complex II and the *Tetramorium severini* species group. Star symbols represent type localities while circles represent non-type localities. Note that *Tetramorium schaufussii* is also recorded from the island of Reunion, but we omit it from the map due to questionable locality data.

## Supplementary Material

XML Treatment for Tetramorium alperti

XML Treatment for Tetramorium dalek

XML Treatment for Tetramorium enkidu

XML Treatment for Tetramorium gilgamesh

XML Treatment for Tetramorium naganum

XML Treatment for Tetramorium bressleri

XML Treatment for Tetramorium gollum

XML Treatment for Tetramorium hobbit

XML Treatment for Tetramorium mars

XML Treatment for Tetramorium plesiarum

XML Treatment for Tetramorium aspis

XML Treatment for Tetramorium camelliae

XML Treatment for Tetramorium cognatum

XML Treatment for Tetramorium freya

XML Treatment for Tetramorium gladius

XML Treatment for Tetramorium karthala

XML Treatment for Tetramorium myrmidon

XML Treatment for Tetramorium proximum

XML Treatment for Tetramorium rumo

XML Treatment for Tetramorium tenuinode

XML Treatment for Tetramorium merina

XML Treatment for Tetramorium monticola

XML Treatment for Tetramorium nassonowii

XML Treatment for Tetramorium obiwan

XML Treatment for Tetramorium pseudogladius

XML Treatment for Tetramorium rala

XML Treatment for Tetramorium schaufussii

XML Treatment for Tetramorium scutum

XML Treatment for Tetramorium sikorae

XML Treatment for Tetramorium xanthogaster

XML Treatment for Tetramorium severini
